# Controlling the Redox Speciation of N,C,N–Bi Complexes Using the Anion Coordination Index

**DOI:** 10.1021/acs.organomet.6c00069

**Published:** 2026-03-24

**Authors:** Vanessa A. Béland, Alexios G. Stamoulis, Nils Nöthling, Josep Cornella

**Affiliations:** Max-Planck-Institut für Kohlenforschung, Kaiser-Wilhelm-Platz 1, Mülheim an der Ruhr 45470, Germany

## Abstract

Recently, N,C,N-bismuth pincer complexes have been shown to engage in a rich repertoire of redox chemistry, which has been harnessed in a myriad of catalytic organic reactions. Despite their ability to maneuver between various oxidation states, the thermodynamic landscape of the redox speciation has never been systematically studied, hampering a deeper understanding of low-valent bismuth redox manifolds. In this work, we probed the stability of a dimeric N,C,N–Bi­(II) pincer (**2OTf**) toward disproportionation in the presence of strongly and weakly coordinating anions, categorized by their coordinating ability index (α). While the complex persists in the presence of noncoordinating anions (α ≤ −0.4), it undergoes disproportionation to the corresponding Bi­(I) (**1**) and Bi­(III) (**3X**) complexes in the presence of coordinating anions (α ≥ 1). When the α falls between −0.4 and 1, excess electrolyte is required for disproportionation. These findings were guided by cyclic voltammetry experiments and further corroborated by spectroscopic and crystallographic characterization of the reaction products.

## Graphical abstract

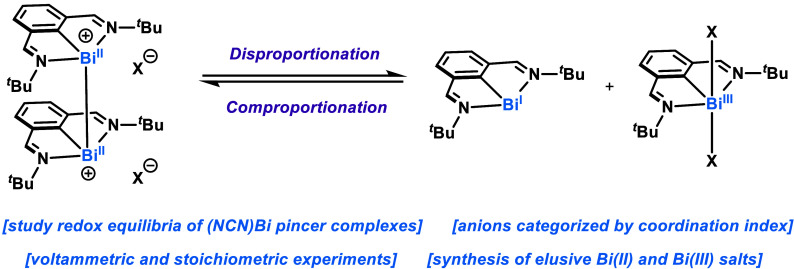

## Introduction

Redox comproportionation and disproportionation events are well established across the p-,^1–16^ d- ^17,18^ and f-blocks^19–27^ of the periodic table. Among their applications,^28–41^ these redox fluctuations have been instrumental in catalysis by controlling fundamental organometallic steps, determining the metal redox speciation under certain conditions, and affording access to rare oxidation states (Figure 1A).^42–46^ Comproportionation/disproportionation equilibria are governed by thermodynamics, and can thus be modulated by numerous variables such as the ligand,^47–50^ additives,^51–54^ nature of the solvent,^51^ and others.^55–65^ While the formation of insoluble, paramagnetic or fleeting species can make the study of comproportionation and disproportionation challenging,^66^ studies geared toward understanding catalyst redox speciation have appeared in the literature.^48,67–76^ These studies have yielded critical insights into stoichiometric and catalytic processes alike, especially in first-row transition metal catalysis.^77–84^ In recent years, main group complexes have shown transition metal-like behavior as redox catalysts.^85–92^ Recent work by Powers et al. has shown how controlling the stability of anodically generated iodanyl radicals toward redox disproportionation via modulation of the electrolyte or the ligand can lead to divergent selectivity. Acetate ions or neighboring iodine atoms can stabilize the incipient iodanyl radical, which is on-path for substrate oxidation, while also promoting its formation at more modest potentials. Conversely, the introduction of perchlorate salts can engender sequential disproportionation events from the anodically generated iodanyl radical to generate high-potential iodoxybenzene species at lower overpotentials (Figure 1B).^16,75,76^ This work showcases the importance of understanding redox disproportionation equilibria in the context of well-defined main group species. In this vein, our development of N,C,N–Bi pincer redox catalysis,^93–99^ where radical pathways play prominent roles,^84,100–105^ has revealed disproportionation of Bi­(II) as a fundamental step in catalysis.^84^ While disproportionation of Bi­(I) to Bi(0) and Bi­(III) is understood for an alternative ligand framework,^31^ it is of capital importance to understand this Bi­(II) to Bi­(I)/Bi­(III) redox equilibrium which occurs in the pincer framework to establish a blueprint for controlling the speciation of the Bi catalyst.^93–105^ This article provides a comprehensive study on the effect of anionic ligands on the redox speciation of the Bi center, with the intention of setting a thermodynamic backdrop that can inform future reaction development and mechanistic studies (Figure 1C).

**Figure 1:**
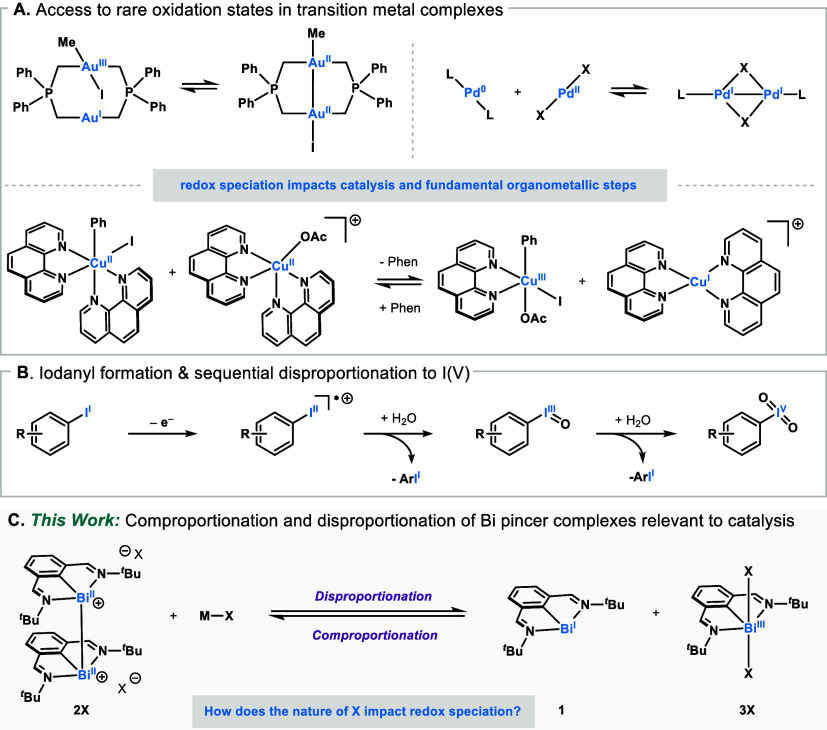
(A) Access to rare oxidation states in transition metal complexes enabled by comproportionation/disproportionation events. (B) Access to high-valent I­(V) enabled by sequential disproportionation at main group centers. (C) Studying the redox speciation of a Bi pincer complex relevant to catalysis.

## Results and Discussion

We commenced by performing voltammetric studies to gauge the counteranion-dependent redox speciation of the Bi complex, comparing the cyclic voltammograms (CVs) of **1** in MeCN using tetrabutylammonium electrolytes bearing different anions. Both the CVs of **1** and **2OTf** in [*n*Bu_4_N]­[OTf] showed an electrochemically reversible feature with a half-wave potential (*E*_1/2_) of −0.830 V vs Fc^+/0^, corresponding to a well-defined Bi^I/II^ redox couple (Figures 2A, S25, and S26). This confirms that the corresponding Bi­(II) from **1** is stable toward disproportionation in the presence of [OTf]^−^ salts. Notably, the CV of **1** collected in [*n*Bu_4_N]­[PF_6_] electrolyte gave a quasireversible redox feature (Figures 2B and S24). In more coordinating electrolytes, the CV of **1** features an electrochemically irreversible pair of redox events, separated by *ca*. 700 mV (Figure 2C). These voltammograms are characteristic of species that, upon 1e^–^ oxidation or reduction at an electrode, transiently generate oxidation states that are unstable toward bimolecular disproportionation, or a rapid second electron transfer at the electrode. This was confirmed to be the case by collecting the CV of **3Cl** in [*n*Bu_4_N]­[PF_6_], which matched the irreversible redox events of **1** collected in [*n*Bu_4_N]­[Cl] (see SI Figure S27). Similar irreversible redox of **1** was observed in the presence of bromide, iodide, tetrafluoroborate, and tosylate anions, indicative of two separate 2e^–^ redox features where Bi­(I) is oxidized to Bi­(III) in the anodic wave, while Bi­(III) is reduced to Bi­(I) in the cathodic wave. It is worth noting that any Bi­(II) generated during the anodic scans was no longer present during the return waves, and Bi^II/I^ redox couples could not be captured even at high scan rates (3200 mV/s, 100 mM [*n*Bu_4_N]­[I], see SI Figure S29). In order to probe whether the mechanism for disproportionation featured an obligate intermediate with a Bi–Bi bond, we recorded the CV of N,C,N–Bi­(I) complex **4** that can support a monomeric Bi­(II) radical cation, owing to the steric bulk imparted by the terphenyl substituents (see SI Figures S32 and S33).^106^ The irreversible redox event of **4Cl** speaks to the instability of the Bi­(II) in the presence of coordinating anions, even when formation of a dimer in solution (akin to **2OTf**) is not feasible. This result strongly suggests that disproportionation and comproportionation events can proceed via outer-sphere electron transfer steps, and do not require formation of a Bi–Bi bond.

**Figure 2:**
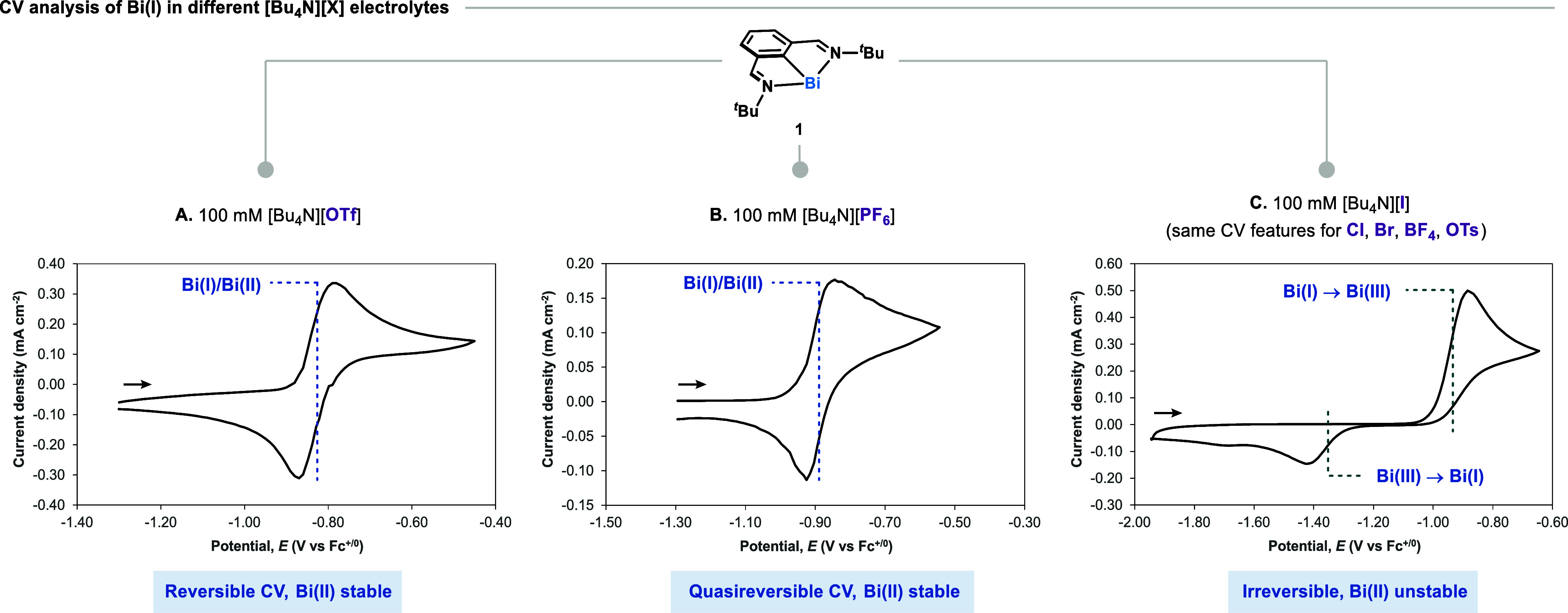
Cyclic voltammograms of **1** in MeCN using 100 mM [*n*Bu_4_N]­[X] as the supporting electrolyte at ambient temperature; scan rate: 100 mV/s; 3-electrode setup composed of glassy carbon disk working electrode, silver wire pseudoreference electrode, and platinum wire counter-electrode. Potential in V vs Fc^+/0^. Black arrow indicates the direction of the potential sweep. (A) Reversible redox event of **1** in a solution of 100 mM [*n*Bu_4_N]­[OTf] in MeCN. The dotted line is used to denote the half-wave potential, *E*_1/2_. (B) Quasi-reversible redox event of **1** in a solution of 100 mM [*n*Bu_4_N]­[PF_6_] in MeCN. The dotted line is used to denote the half-wave potential, *E*_1/2_. (C) Irreversible redox event of **1** in a solution of 100 mM [*n*Bu_4_N]­[I], featuring two separated 2-electron features corresponding to Bi­(I) to Bi­(III) in the anodic wave and Bi­(III) to Bi­(I) in the cathodic wave. The dotted line is used to denote the half-peak potential, *E*_p/2_.

The data from the CV studies already pointed toward the ability of weakly coordinating anions (such as [OTf]^−^ and [PF_6_]^−^) to stabilize the respective Bi­(II) species generated electrochemically. This implies that disproportionation of Bi­(II) to Bi­(I) and Bi­(III) should be favored for more coordinating anions, while comproportionation should be favored for less coordinating anions. We sought to validate these hypotheses through a series of stoichiometric studies with the Bi­(II) complex **2OTf** and a series of anions (Scheme 1A). When 2 equiv of the naked fluoride source, [Cp_2_Co]­[F][Bibr ref107] was reacted with **2OTf**, **1** and **3F** were obtained in 95% and >95% NMR yield, respectively. Similarly, addition of the commercially available fluoride source [*n*Bu_4_N]­[Ph_3_SiF_2_] resulted in disproportionation of **2OTf** into **1** and **3F**. In agreement with the CV data for **1** collected in tetrabutylammonium chloride, bromide and iodide electrolytes, it was found that 2.0 equiv of salts bearing coordinating anions (X) including chloride, bromide, iodide, acetate, benzoate, phthalimide, phenoxide and thiophenolate could disproportionate **2OTf** to give quantitative yields for **1** and the corresponding N,C,N–Bi^III^X_2_ complexes (**3X**, Scheme 1B, SI Section 5). While addition of CO_3_^2–^ and PO_4_^3–^ salts resulted in the formation of insoluble species, which complicated characterization of any Bi­(III) species, quantitative formation of **1** is circumstantial evidence pointing to disproportionation of **2OTf** (see SI Section 6). It is noteworthy that all of the **3X** disproportionation products exclusively exhibit homoleptic substitution (i.e., no mixed [X]^−^ [OTf]^−^ Bi­(III) complexes are observed), even when substoichiometric loadings of salts are used (see SI Section 8).

**Scheme 1:**
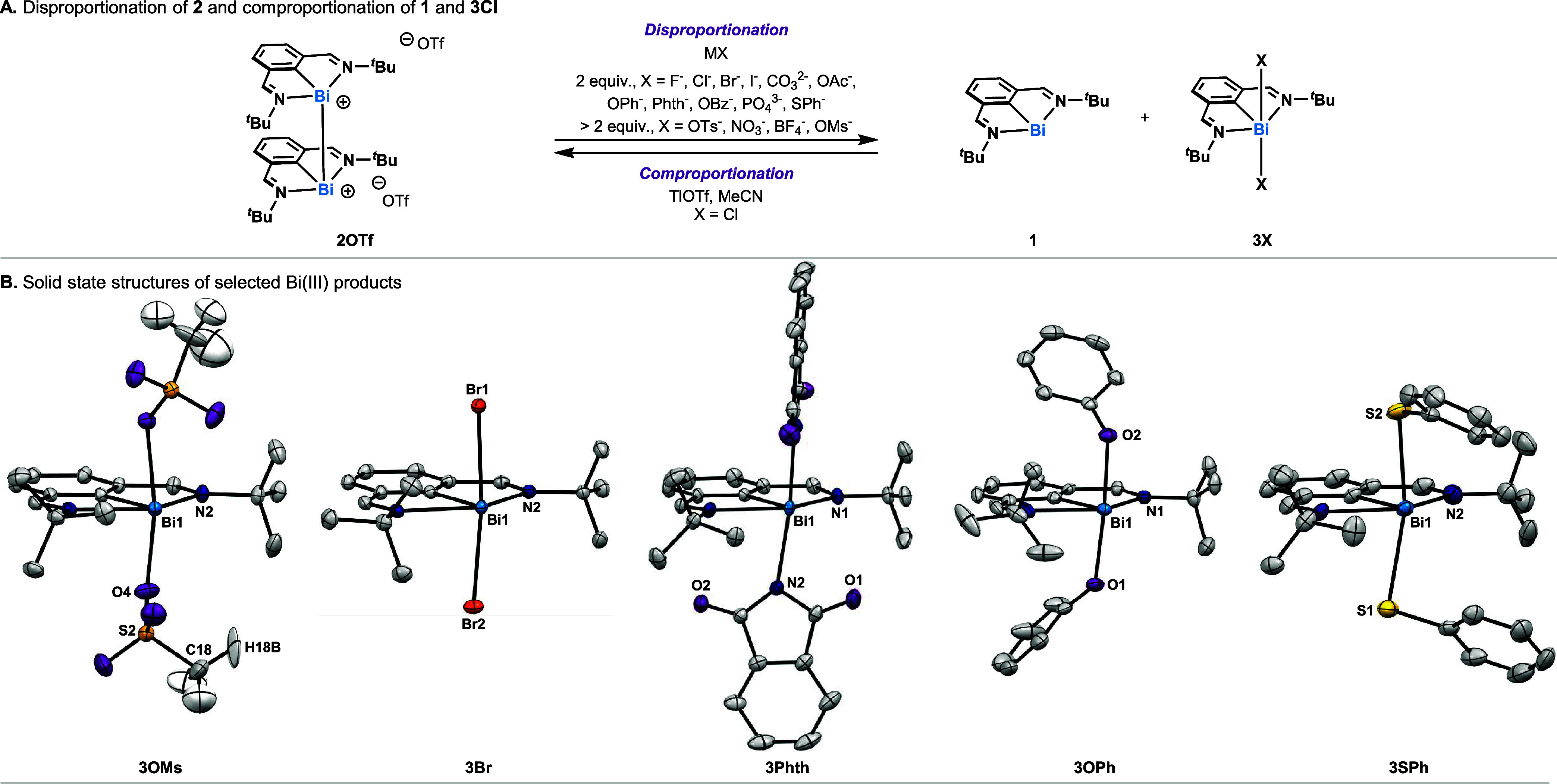
(A) Disproportionation of **2OTf** and Comproportionation of **1** and **3Cl**. (B) Solid State Structures of **3OMs**, **3Br**, **3Phth**, **3OPh** and **3SPh** Visualized with 50% Probability Ellipsoids. For the Sake of Clarity, H Atoms, Disorder Parts and Solute Molecules have been Omitted. Relevant Lengths and Angles: **3OMs**: C(1)–Bi(1) 2.1800(16)­Å, N(1)–Bi(1) 2.4787(15) Å, N(2)–Bi(1) 2.4782(14) Å, Bi(1)–O(1) 2.3534(13) Å, Bi(1)–O(4) 2.3740(14)­Å, C(1)–Bi(1)–N(1) 72.08(6)°, C(1)–Bi(1)–N(2) 71.72(5)°, O(1)–Bi(1)–O(4) 157.66(5)°; **3Br**: C(1)–Bi(1) 2.1982(16) Å, N(1)–Bi(1) 2.5092(13) Å, N(2)–Bi(1) 2.4909(13) Å, Bi(1)–Br(1) 2.82495(17) Å, Bi(1)–Br(2) 2.83243(17) Å, C(1)–Bi(1)–N(1) 71.09(5)°, C(1)–Bi(1)–N(2) 71.48(5)°, Br(1)–Bi(1)–Br(2) 172.450(5)°; **3Phth**: C(1)–Bi(1) 2.1952(12)­Å, N(1)–Bi(1) 2.4928(8) Å, N(2)–Bi(1) 2.4408(9) Å, C(1)–Bi(1)–N(1) 71.51(2)°, N(2)–Bi(1)–N(2) 171.18(4)°; **3OPh**: C(1)–Bi(1) 2.193(5) Å, N(1)–Bi(1) 2.487(4) Å, N(2)–Bi(1) 2.538(4) Å, Bi(1)–O(1) 2.318(3)­Å, Bi(1)–O(2) 2.243(3) Å, C(1)–Bi(1)–N(1) 71.39(15)°, C(1)–Bi(1)–N(2) 70.51(16)°, O(1)–Bi(1)–O(2) 175.46(12)°; **3SPh**: C(1)–Bi(1) 2.211(6) Å, N(1)–Bi(1) 2.461(5) Å, N(2)–Bi(1) 2.576(8) Å, Bi(1)–S(1) 2.7876(19) Å, Bi(1)–S(2) 2.714(2) Å, C(1)–Bi(1)–N(1) 71.3(2)°, C(1)–Bi(1)–N(2) 70.3(2)°, S(1)–Bi(1)–S(2) 165.43(8)°

This suggests that two exogenous anions are required to drive the disproportionation to completion, speaking to the greater stability of the homoleptic (N,C,N–Bi^III^X_2_) over the heteroleptic [N,C,N–Bi^III^(OTf)­(X)] complexes.

This finding also shows that disproportionation of **2OTf** can be used as a tool to access Bi­(III) complexes substituted with two electron-withdrawing X-type ligands (as in **3Phth** and **3OAc**), which cannot be easily accessed by salt metathesis routes (See SI Section 8). In an attempt to gauge the proclivity toward disproportionation with organic X-type ligands, **2OTf** was reacted with MeLi and PhLi. However, **1** and **3X** could not be assigned in the reaction mixture. This can be rationalized by previous reports featuring complex **1**, which show that these types of organometallic reagents can react directly with the imines on the ligand scaffold.^84^

Given that **2OTf** can disproportionate in the presence of coordinating ions, we speculated that the reverse could be true for comproportionation. To test this, **1** and **3Cl** were combined in the presence of the redox-innocent thallium triflate chloride scavenger. The teal solution of **1** immediately became orange with yellow precipitate on addition of **3Cl** and thallium triflate, affording **2OTf** in >95% NMR yield. Scavenging the coordinating chloride ions by precipitation of TlCl allows for reduction of **3Cl** by **1** to form stable **2OTf** (Scheme 1). In addition, **2OTf** could be accessed in >95% NMR yield by reduction of **3Cl** with 1 equiv of cobaltocene in the presence of thallium triflate (see SI Section 3). This finding supports the CV data, which suggest that the stability of Bi­(II) hinges on the presence of noncoordinating anions.

Since the voltammetric data of **1** revealed the presence of two distinct Bi­(I/III) redox events when conducted in [*n*Bu_4_N]­[BF_4_] and [*n*Bu_4_N]­[OTs], we predicted that these anions could also be used to disproportionate **2OTf** (see SI Figures S30 and S31). However, adding 2 equiv of [*n*Bu_4_N]­[BF_4_] and [*n*Bu_4_N]­[OTs] to **2OTf** did not show any sign of disproportionation. To mimic the large excess of anions present in the electrochemical conditions, excess salt equivalents were titrated into solutions of **2OTf**. The yield of **1** leveled off at 77% with 23 equiv of [*n*Bu_4_N]­[BF_4_] and at 88% with 223 equiv of [*n*Bu_4_N]­[OTs] (see SI Section 7). Similarly, [OMs]^−^ and [NO_3_]^−^ only engendered disproportionation when excess equivalents were added to **2OTf**. Based on this finding, we speculated that Bi­(II) salts with [BF_4_]^−^, [OMs]^−^, [OTs]^−^ and [NO_3_]^−^ counterions could be prepared to glean insights into the coordination-induced disproportionation. Oxidizing **1** with the corresponding Ag­(I) salts of the above anions gave spectra consistent with **2X** complexes by ^1^H NMR spectroscopy (Scheme 2A). Although **2OMs**, **2OTs** and **2NO**_**3**_ disproportionated during isolation attempts (SI Section 4), single crystals suitable for SC-XRD could be obtained for **2BF**_**4**_. The solid state structure of **2BF**_**4**_ exhibits a slightly contracted Bi–Bi bond length (3.0794(3) Å) relative to that in **2OTf** (3.09439(19) Å) (Scheme 2B).^83^ Similar to **2OTf**, the C(1)–Bi(1)–Bi(1) angle of 100.94° in **2BF**_**4**_ is a structural representation of the involvement of the 6p_*z*_ orbital in the Bi–Bi bond.

**Scheme 2:**
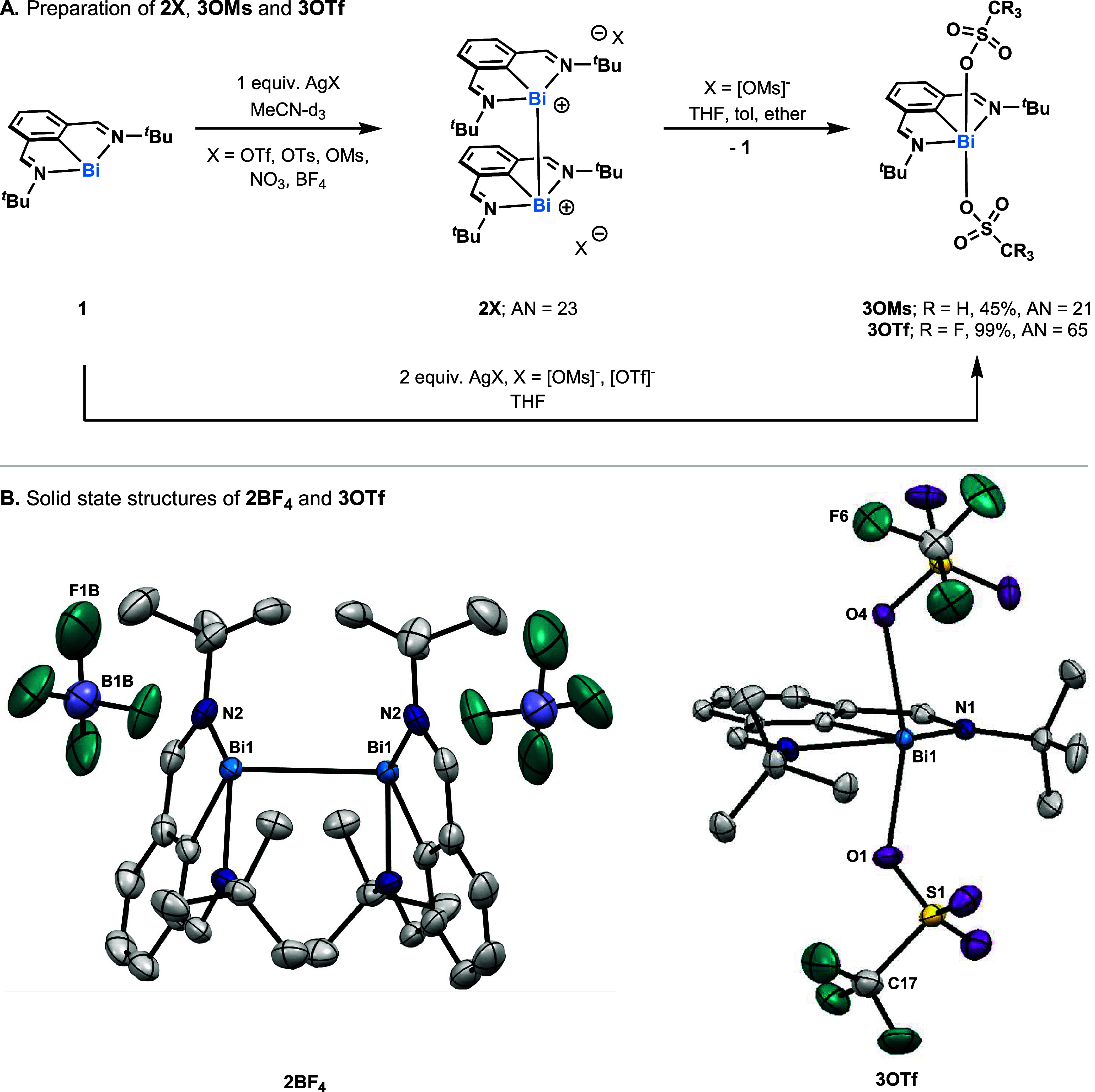
(A) Preparation of Bi­(II) Salts (**2X**, NMR Yields **2OTf**, 82%; **2OTs**, 94%; **2OMs**, >95%; **2NO**_**3**_, 94%; **2BF**_**4**_, >95% (Isolated Yield, 63%)) Showing Synthesis of **3OMs** and **3OTf**. (B) Solid State Structures of **2BF**_**4**_, and **3OTf** Visualized with 50% Probability Ellipsoids. For the Sake of Clarity, H Atoms, Disordered Parts, and Solvent Molecules Have Been Omitted. Relevant Lengths and Angles: **2BF**_**4**_: Bi(1)–Bi(1) 3.0794(2) Å, C(1)–Bi(1) 2.193(4) Å, N(1)–Bi(1) 2.461(3) Å, N(2)–Bi(1) 2.542(3) Å, C(1)–Bi(1)–N(1) 71.93(13)°, C(1)–Bi(1)–N(2) 70.75(13)°, C(1)–Bi(1)–Bi(1) 100.88(10)°; **3OTf**: C(1)–Bi(1) 2.1818(19) Å, N(1)–Bi(1) 2.4616(17) Å, N(2)–Bi(1) 2.4736(17)­Å, Bi(1)–O(1) 2.3820(15) Å, Bi(1)–O(4) 2.4158(15) Å, C(1)–Bi(1)–N(1) 72.16(6)°, C(1)–Bi(1)–N(2) 71.79(6)°, O(1)–Bi(1)–O(4) 160.03(5)°

In order to further probe the factors underlying the stability of **2OTf**, we sought to prepare its Bi­(III) congener (**3OTf**) with the hopes of quantifying its Lewis acidity by the Gutmann-Beckett method.^108–110^ While the OMs analogue (**3OMs**) could be accessed by disproportionation of **2OMs**, **3OTf** could only be accessed by oxidation of **1** with 2.0 equiv AgOTf (Scheme 2A).^111–113^ Upon addition of 1.0 equiv OPEt_3_ to each complex, acceptor numbers (AN) of 65, 23, 21, and 22 were calculated for **3OTf**, **2OTf**, **3OMs** and **3Cl**, respectively. Interestingly, the average Bi–O bond lengths decrease from **3OTf** > **3OMs** > **3OPh** (2.3988(16), 2.3637(14) and 2.281(3) Å, respectively). The longer Bi–O bonds in **3OTf** could indicate a weaker metal–ligand interaction, which contributes to the highly Lewis-acidic nature of the complex. Compound **3OTf** is a significantly stronger Lewis acid than its Bi­(III) analogues, which could allow for comproportionation with **1** ^93–105,114–116^ to make dimeric **2OTf**. This dramatic difference in Lewis acidity between **3OTf** and the other **3X** congeners could offer a clue as to why disproportionation is favored for more coordinating anions. Since the anionic ligands should not affect the stability of the Bi­(I) formed after disproportionation of Bi­(II), the thermodynamics of the redox equilibrium will be determined by the relative stabilization of the Bi­(II) and Bi­(III) complexes. While anions have a large effect on the positive charge buildup at Bi­(III), the small difference in Bi–Bi bond length (0.015 Å) between **2BF**_**4**_ and **2OTf** suggests a minimal effect on the charge buildup at the Bi­(II) centers as a function of the anionic ligand identity. We therefore hypothesize that the ability of more coordinating anions to quench the positive charge concentrated at the Bi­(III) center rationalizes their ability to drive the disproportionation reaction. This principle rationalizes the stability of **2OTf**, which is thermodynamically favored over the highly unstable, Lewis-acidic **3OTf** congener.

In an attempt to develop a semiquantitative method to rationalize the stability of Bi­(II) in the presence of various anions, we categorized each anion according to its coordination index. The Alvarez parameter (α)^117,118^ was derived for individual anions in solid state structures which contain transition metals: it represents the ratio of coordinated to uncoordinated anion in solid-state structures found in the Cambridge Crystallographic Data Centre (CCDC).^117,118^ It should be noted that although p*K*_a_ values can be used as a semiquantitative gauge for Lewis-basicity of conjugate bases, these values are ill-defined for conjugate acids of weakly coordinating anions (e.g., HBAr^F^) in organic solvents. Three distinct categories emerged after placing each anion along the α scale (Figure 3). Strongly coordinating anions (α ≥ 1) engender disproportionation of the Bi­(II) even with stoichiometric loadings (2 equiv). Weakly- and noncoordinating anions (α ≤ – 0.4) do not disproportionate **2OTf** even when excess equivalents are used. While the CVs of **1** and **2** conducted in [*n*Bu_4_N]­[PF_6_] are quasireversible, neither stoichiometric (2.0 equiv) nor excess (100.0 equiv) loadings resulted in disproportionation. To probe this further, tetrakis­(3,5-bis­(trifluoromethyl)­phenyl)­borane ([BAr^F^]^−^, α = −3.4), [SbF_6_]^−^ (α = −1.0) and [Al­(OC­(CF_3_)_3_)_4_]^−^ (α = −1.4)^119,120^ were tested, none of which resulted in disproportionation of **2OTf**. The last category falls in the range of −0.4 < α < 1, where excess equivalents of electrolyte are required to disproportionate **2OTf**. Anions in this region include [*n*Bu_4_N]­[NO_3_] (α = 0)^118^ and [*n*Bu_4_N]­[OMs] (α = 0.4),^118^ which were both found to require excess equivalents for disproportionation.

**Figure 3:**
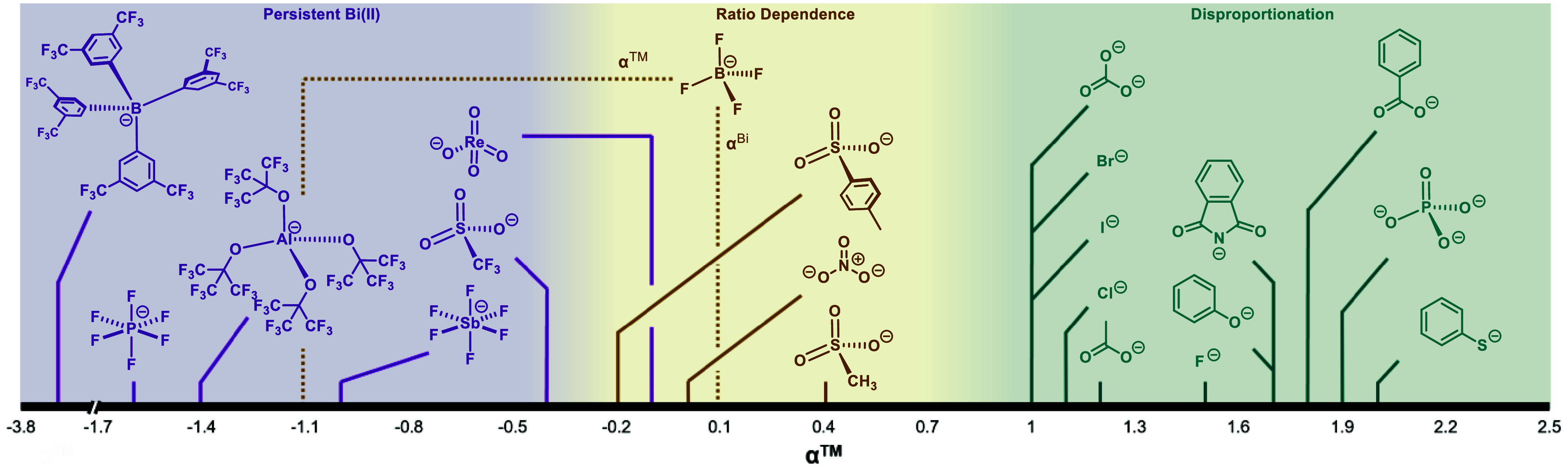
Scope of ions plotted against ion coordination index, α.

Although the above data validate the use of the α parameter as a semiquantitative method to rationalize and predict stability of Bi­(II) toward various anions, some exceptions can arise. When employing stoichiometric (2.0 equiv) or excess (100.0 equiv) tetrabutylammonium perrhenate ([ReO_4_]^−^ α = −0.1),^118^ no disproportionation was observed, despite being in the range of α values that should feature a ratio-dependent stability of the Bi­(II) toward disproportionation. Furthermore, while [BF_4_]^−^, with an α value of −1.1,^118^ is expected to lead to stable Bi­(II), it engenders disproportionation with excess equivalents (in keeping with the CV data). It is important to note that α is a statistical value based on anion coordination to transition metals in the solid state, under a range of different conditions (e.g., solvent, antisolvent, temperature) and therefore exceptions might occur. However, following the Alvarez approach,^117,118^ we were able to calculate a Bi-specific value (α^Bi^) for [BF_4_]^−^ of 0.1, from 24 structures in the CCDC (see SI Section 12). This would place [BF_4_]^−^ in the range of anions featuring a loading-dependent stability of the Bi­(II), which now matches with the experimental data. Therefore, although a general trend holds with tabulated α values, exceptions to this trend are expected and can be corrected by considering the crystallographic data for bismuth, when possible.

## Conclusions

The work outlined herein gives insights into the effect of a broad range of anions on the redox speciation of a catalytically salient class of N,C,N–Bi pincer complexes. Initial CV studies suggest that the stability of the electrochemically generated Bi­(II) at the electrode has an inverse relationship with the coordinating ability of the anion used in the electrolyte solution. This hypothesis is validated by stoichiometric studies conducted by mixing a Bi­(II) model compound, **2OTf**, with stoichiometric quantities of tetrabutylammonium salts bearing different anions. The insights obtained allowed for the synthesis of several new Bi­(II) and Bi­(III) salts, including the elusive and highly Lewis-acidic **3OTf**, which is not accessible via disproportionation. The high Lewis acidity of **3OTf** suggests that the redox equilibrium is determined by the relative ability of anions to stabilize Bi­(III) over Bi­(II), with more coordinating anions stabilizing the former species to a larger extent. While **2OTf** persists in the presence of weakly- and noncoordinating anions (α ≤ −0.4), rapid and quantitative disproportionation is observed with more coordinating anions (α ≥ 1). Within the range of −0.4 < α < 1, it is found that disproportionation only occurs when an excess of the anion is employed. While the predictive ability of α is largely validated, Bi-specific values (α^Bi^) can be calculated to account for outliers. The results in this study have implications for the growing repertoire of reactions where bismuth complexes engage in catalytic redox manifolds that feature both 1- and 2-electron elementary steps. For example, the insights provided herein can be used to curb unwanted formation of off-cycle species through control of the anions in solution. This study is therefore expected to inform the design of future bismuth-catalyzed reactions and guide their mechanistic interrogation.

## Supplementary Material



## Notes

Experimental and characterization details (PDF)

## Abbreviations

α: coordination index to transition metals (Alvarez parameter)
α^Bi^: coordination index specific to bismuth
AN: acceptor number
BAr^F^: tetrakis­(3,5-bis­(trifluoromethyl)­phenyl)­borane
CCDC: Cambridge Crystallographic Data Centre
CV(s): cyclic voltammogram(s)
(*E*_1/2_): half-wave potential
Fc^+/0^: ferrocene redox couple
MeCN: acetonitrile
NMR: nuclear magnetic resonance
OMs: mesylate
OTf: triflate
OTs: tosylate
Phth: phthalimide
SC-XRD: single-crystal X-ray diffraction
SET: single-electron transfer.
