# Supplementary material for: Controlling the Redox Speciation of N,C,N–Bi Complexes Using the Anion Coordination Index
Source: Organometallics. 2026 Mar 24;45(7):902–11. doi: 10.1021/acs.organomet.6c00069 (PMC13081114; doi:10.1021/acs.organomet.6c00069)
Supplement: Supplementary file 1 [file om6c00069_si_001.pdf]

Supporting Information

Controlling the Redox Speciation of N,C,N-Bi Complexes Using the Anion Coordination Index

Vanessa A. Béland[+], Alexios G. Stamoulis[+], Nils Nöthling and Josep Cornella*

Max Planck Institut für Kohlenforschung, Kaiser-Wilhelm-Platz 1, Mülheim an der Ruhr, 45470, Germany

cornella@kofo.mpg.de

Contents

1. General Experimental Notes ... 3
2. Preparation of Model Bi(III) Complexes ... 5
   2.1 Synthesis of bromobismuthine (**3Br**) ... 5
   2.2 Synthesis of benzoylbismuthine (**3OBz**) ... 6
   2.3 Synthesis of phenoxybismuthine (**3OPh**) ... 7
   2.4 Synthesis of thiophenolatebismuthine (**3SPh**) ... 8
   2.5 Synthesis of phthalimide-bismuthine (**3Phth**) ... 9
   2.6 Synthesis of phthalimide-triflate-bismuthine (**3Phth-OTf**) ... 10
   2.7 Synthesis of mesylate-bismuthine (**3OMs**) ... 11
   2.8 Synthesis of triflate-bismuthine (**3OTf**) ... 12
   2.9 Tabulated Diagnostic NMR data ... 13
3. Reduction and Comproportionation Routes to 2OTf ... 14
4. Preparation of Bi(II) Salts ... 16
   4.1 General Synthesis of Bi(II) salts (**2X**) ... 16
   4.2 Synthesis of tetrafluoroborate Bi(II) salt (**$2BF_4$**) ... 18
5. Anion Screening for Disproportionation of 2OTf ... 19
6. Molar Absorptivity Calculations ... 25
   6.1 Molar Absorptivity of **1** ... 25
   6.2 Molar Absorptivity of **2OTf** ... 27
   6.3 Calculations for concentration of **1** and **2OTf** in titrations ... 28
7. Ratio Dependent Disproportionation Reactions ... 29
8. Substoichiometric Disproportionation Reactions ... 34
9. Solvent Dependence in 2OMs ... 42

10. Gutmann-Beckett Method ... 47
11. Cyclic Voltammetry ... 53
12. α Calculations ... 59
11.1 Calculation for phthalimide α^{TM} ... 59
11.2 Calculation for tetrafluoroborate α^{Bi} ... 60
13. NMR Spectra ... 61
14. X-Ray Crystallography ... 95
14.1 Single crystal structure analysis of **3Br** acetonitrile solvate ... 95
14.2 Single crystal structure analysis of **3OPh** diethyl ether solvate ... 102
14.3 Single crystal structure analysis of **3SPh** ... 108
14.4 Single crystal structure analysis of **3Phth** tetrahydrofuran solvate ... 118
14.5 Single crystal structure analysis of **3Phth-OTf** ... 125
14.6 Single crystal structure analysis of **3OMs** ... 131
14.7 Single crystal structure analysis of **3OTf** ... 138
14.8 Single crystal structure analysis of **2BF$_4$** ... 147
15. References ... 155

1. General Experimental Notes

Instrumentation: Manipulations were conducted using standard Schlenk line techniques or in a glove box under an atmosphere of dry Ar gas unless otherwise noted. ^{1}H, $^{13}C\{^{1}H\}$, $^{11}B\{^{1}H\}$, ^{19}F and $^{31}P\{^{1}H\}$ NMR spectra were recorded with Bruker Avance III HD nanobay 300 MHz, Bruker Avance III HD 400 MHz, Bruker Avance III 500 MHz, or Bruker Avance NEO 600 MHz NMR spectrometers, and processed using MestReNova. ^{1}H NMR chemical shifts are given in ppm with respect to the residual solvent peak (C_6D_6, δ 7.16 ppm; THF-d_8, δ 1.73 ppm; CD_3CN, δ 1.94 ppm). $^{13}C\{^{1}H\}$ NMR chemical shifts are given in ppm with respect to solvent peak CD_3CN, δ 118.26 ppm). $^{11}B\{^{1}H\}$ NMR chemical shifts are given in ppm relative to 15% BF_3•OEt_2 in $CDCl_3$. ^{19}F NMR chemical shifts are given in ppm relative to $CFCl_3$. $^{31}P\{^{1}H\}$ NMR chemical shifts are given in ppm relative to phosphoric acid. Multiplicities are described as s = singlet, br s = broad singlet, d = doublet, br d = broad doublet, t = triplet, q = quartet, dd = doublet of doublets, m = multiplet. Coupling constants are reported as J-values in Hz. High-resolution mass spectra were obtained using Bruker APEX III FT-MS with ESI ionization source or Finnigan MAT 95 with EI ionization source. Electrochemical experiments were conducted using Gamry Interface 1010E potentiostat. UV-vis spectra were collected using a Cary 6000i UV-Vis-NIR spectrophotometer from Agilent Technologies. Mass spectra were collected using a Q Exactive Plus Hybrid Quadrupole-Orbitrap mass spectrometer from Thermo Fisher.

Reagents: Acetonitrile (MeCN and MeCN-d_3), diethyl ether (Et_2O), toluene (MePh), pentane, dimethylformamide (DMF and DMF-d_7), dichloromethane (CH_2Cl_2 and CD_2Cl_2), and tetrahydrofuran (THF and THF-d_8) were dried over calcium hydride, distilled and stored under argon over sieves (3 Å for MeCN and MeCN-d_3 and 4 Å for all other solvents). Anhydrous chlorobenzene was purchased from Aldrich and used as received. Thallium triflate (TlOTf) was used as received from STREM. **CAUTION**: TlOTf is highly toxic and should be handled accordingly. The following chemicals were used as received; tetrabutylammonium difluorotriphenylsilicate ([NBu_4][Ph_3SiF_2], Aldrich), sodium hexafluoroantimonate ($NaSbF_6$, Aldrich), sodium methanesulfonate (NaOMs, Aldrich), sodium thiophenolate (NaSPh, Aldrich), potassium phthalimide ([K][$C_8H_4NO_2$], Aldrich), silver triflate (AgOTf, Aldrich), cobaltocene (Cp_2Fe, Strem), silver tetrafluoroborate ($AgBF_4$, Aldrich), silver tosylate (AgOTs, Aldrich), silver methanesulfonate (AgOMs, Aldrich), silver nitrate ($AgNO_3$), triethylphosphineoxide ($OPEt_3$, Aldrich), tetrabutylammonium perrhenate ([NBu_4][ReO_4], Aldrich) potassium carbonate (K_2CO_3, Acros Organics) and potassium phosphate (K_3PO_4). The following chemicals were purchased and purified; tetrabutylammonium chloride ([NBu_4][Cl], Aldrich, precipitated from acetone via addition of diethyl ether, then dried under high vacuum by slowly ramping the temperature up to 50 °C, and leaving the material at this temperature for 18 hours), tetrabutylammonium bromide ([NBu_4][Br], Aldrich, was dried under high vacuum at 75 °C overnight), tetrabutylammonium iodide ([NBu_4][I], Aldrich, crystallized from a saturated solution in toluene by layering with pentane, then dried under high vacuum at 90 °C overnight), tetrabutylammonium hexafluorophosphate ([NBu_4][PF_6], Aldrich, recrystallized from hot ethanol, dried under high vacuum at 80 °C overnight), tetrabutylammonium tetrafluoroborate ([NBu_4][BF_4], Aldrich, recrystallized from a saturated solution in ethyl acetate by layering with pentane, then dried under high vacuum at 80 °C overnight), tetrabutylammonium triflate ([NBu_4][OTf], Aldrich, dried under high vacuum at 90 °C overnight), tetrabutylammonium tosylate ([NBu_4][OTs], Aldrich, dried *in vacuo* at 25 °C), tetrabutylammonium acetate ([NBu_4][OAc], Aldrich, dried *in vacuo* at 25 °C),

tetrabutylammonium benzoate ($[NBu_4][OBz]$, Aldrich, dried *in vacuo* at 25 °C), tetrabutylammonium nitrate ($[NBu_4][NO_3]$, Aldrich, dried *in vacuo* at 25 °C) and tetrabutylammonium methanesulfonate ($[NBu_4][OMs]$, Aldrich, dried *in vacuo* at 25 °C).

2. Preparation of Model Bi(III) Complexes

The preparation for compounds **3F**, **3Cl**, **3I** and **3OAc** are reported in the literature and these compounds could be synthesized for comparison to disproportionation products when appropriate.[1–4]

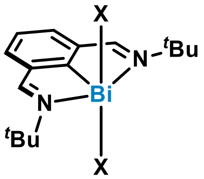

3F, X = F
3Cl, X = Cl
3I, X = I
3OAc, X = OAc

2.1 Synthesis of bromobismuthine (**3Br**)

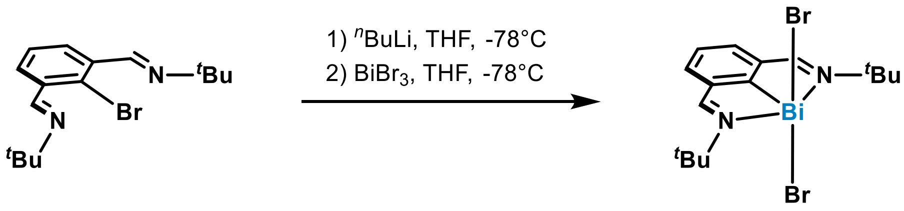

3Br

Compound **3Br** was prepared in a similar manner to **3Cl**.[2] A hexane solution of ^{n}BuLi (0.9 mL, 2.5 M, 2 mmol, 1 equiv.) was added to a precooled (-78°C) solution of 2,6-(^{t}BuN=CH)$_2$C$_6$H$_3$Br (0.698 g, 2.16 mmol, 1.0 equiv.) in THF (20 mL) and stirred for 1 hour. The resulting yellow-brown suspension was added to a solution of $BiBr_3$ (0.974 g, 2.17 mmol, 1.0 equiv.) in THF (15 mL) at -78°C. The solution was allowed to warm to room temperature and was stirred for 18 h to give a yellow suspension. The workup was performed in air. Volatiles were removed *in vacuo* to give a pale orange solid, which was extracted with dichloromethane (3x60 mL). The extract portions were combined and the volume reduced (ca. 10 mL), then the solution was stirred and cyclohexane (250 mL) was added to give a pale orange precipitate. The solids were collected on a frit and dried *in vacuo* to give 1.298 g, 98 % yield of **3Br**.

Crystals suitable for single crystal X-ray diffraction studies were grown by vapor diffusion of diethyl ether into an acetonitrile solution of **3Br** at -10 °C.
^{1}H NMR (600.22 MHz, MeCN-*d*$_3$): δ = 9.76 (s, 2H, aldimine), 8.31 (d, $^{3}J_{HH}$ = 6 Hz, 2H, pincer *meta*), 7.96 (t, $^{3}J_{HH}$ = 6 Hz, 1H, pincer *para*), 1.55 (s, 18H, ^{t}Bu) ppm.
^{13}C{^{1}H} NMR (150.94 MHz, MeCN-*d*$_3$): δ = 169.4, 150.2, 137.4, 130.6, 118.3, 62.7, 31.2 ppm.
Positive ion ESI-MS found (calculated): $[C_{16}H_{23}N_2BrBi]^+$ ($[M\text{-}Br]^+$) m/z 531.08426 (531.08431).
M.p. The compound does not melt, or visibly decompose below 250 °C.

2.2 Synthesis of benzoylbismuthine (**3OBz**)

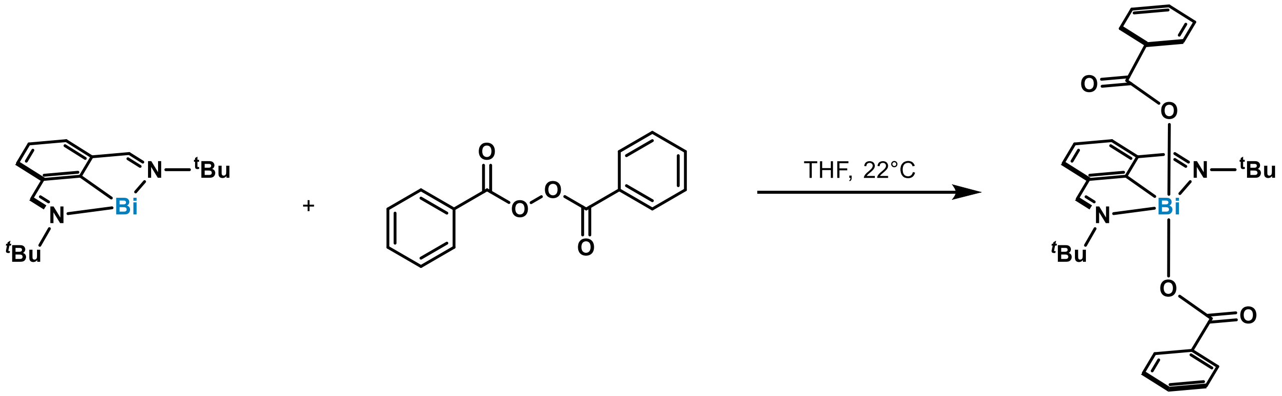

1 **3OBz**

A THF (4 mL) solution of benzoyl peroxide (34 mg, Luperox A75, 75%, remainder water, 0.12 mmol, 1.3 equiv.) was added to a THF (2 mL) solution of **1** (41 mg, 0.092 mmol, 1.0 equiv.), resulting in an immediate color change from dark teal to colorless. The solvent was removed *in vacuo* to give a white solid, which was extracted with toluene (4 mL), filtered over Celite, then layered with pentane (6 mL) and cooled to -35°C for four days to give colorless crystals. The crystals were decanted and dried *in vacuo* to give 30 mg, 53 % yield of **3OBz**.

^{1}H NMR (600.20 MHz, MeCN-d_3): δ = 9.83 (s, 2H, aldimine), 8.23 (d, $^{3}J_{HH}$ = 8 Hz, 2H, pincer *meta*), 7.86 (t, $^{3}J_{HH}$ = 8 Hz, 1H, pincer *para*), 7.73 (m, 4H, OBz *ortho*), 7.40 (m, 2H, OBz *para*), 7.31 (m, 4H, OBz *meta*), 1.52 (s, 18H, ^{t}Bu) ppm.
^{13}C{^{1}H} NMR (150.94 MHz, MeCN-d_3): δ = 212.6, 174.0, 167.3, 151.7, 137.3, 135.8, 131.8, 130.5, 130.0, 128.8, 61.3, 30.7 ppm.
Positive ion ESI-MS found (calculated): $[LBiOBz]^+$ = $[C_{23}H_{28}N_2O_2Bi]^+$ ($[M\text{-}OBz]^+$) = m/z 573.19459 (573.19493).
M.p. The compound does not melt, or visibly decompose below 250 °C.

2.3 Synthesis of phenoxybismuthine (**3OPh**)

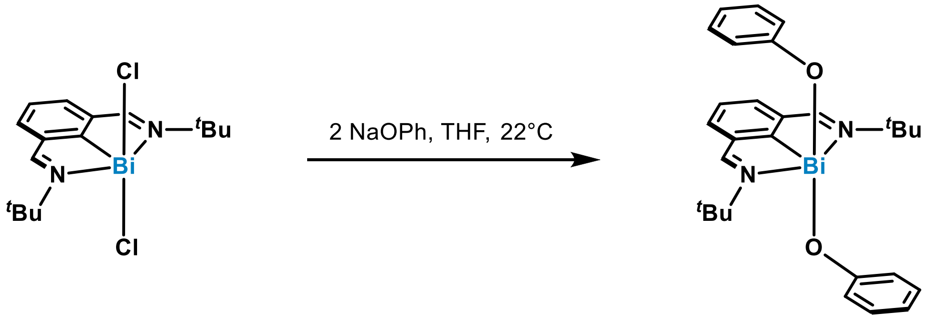

3Cl **3OPh**

A THF stock solution of phenol (2 mL, 0.15 M, 0.30 mmol) was added to solid NaH (16 mg, 0.68 mmol). After effervescence ceased, the solution was stirred for 10 minutes before filtering over Celite into a clean vial. The resulting sodium phenoxide solution (1274 μL, 0.19 mmol, 2.1 equiv.) was added to a THF (1 mL) suspension of **3Cl** (47 mg, 0.089 mmol, 1.0 equiv.) resulting in an immediate color change to Aperol orange and dissolution of all insoluble components. The solution was stirred for 10 minutes, then the solvent was removed *in vacuo* to give a yellow solid, which was extracted with toluene (2x2 mL), filtered over Celite, then layered with pentane (6 mL) and stored at -35°C for four days to give yellow needle-shaped crystals. The crystals were decanted and dried *in vacuo* to give 33 mg, 53 % yield of **3OPh**.

Crystals suitable for single crystal X-ray diffraction studies were grown by vapor diffusion of pentane into a THF solution of **3OPh** at -35°C.

^{1}H NMR (600.20 MHz, MeCN-d_3): δ = 9.73 (s, 2H, aldimine), 8.06 (d, $^3J_{HH}$ = 8 Hz, 2H, pincer meta), 7.72 (t, $^3J_{HH}$ = 8 Hz, 1H, pincer para), 6.83 (m, 4H, OPh meta), 6.25 (m, 2H, OPh para), 6.06 (m, 4H, OPh ortho), 1.47 (s, 18H, ^{t}Bu) ppm.

^{13}C{^{1}H} NMR (150.94 MHz, MeCN-d_3): δ = 212.1, 168.0, 167.0, 149.7, 136.4, 130.0, 129.3,120.9,115.7, 61.3, 30.9 ppm.

Positive ion ESI-MS found (calculated): [LBiOPh]$^+$ = [$C_{22}H_{28}N_2OBi$]$^+$ ([M-OPh]$^+$) = m/z 545.20037 (545.20001).

M.p. The compound does not melt, but begins to decompose at 197 °C.

2.4 Synthesis of thiophenolatebismuthine (**3SPh**)

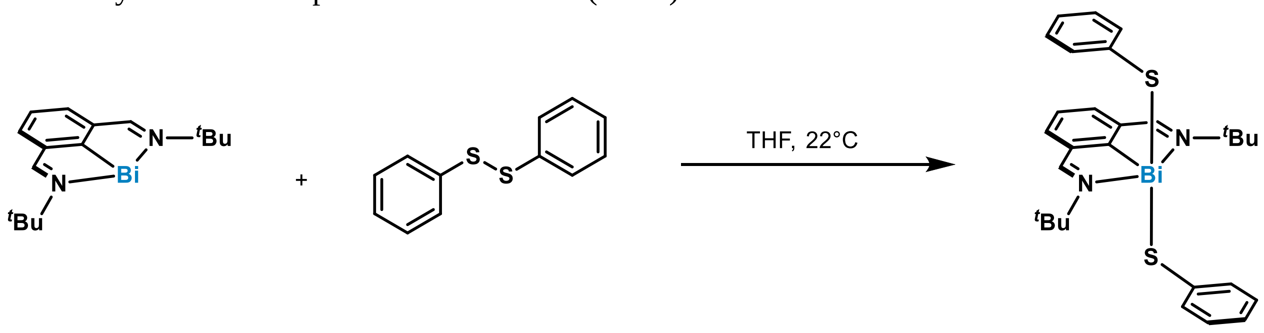

1 **3SPh**

Compound **3SPh** was prepared following a modified procedure to make the Sb analogue reported by Schulz and Dostál.[5] Briefly, a THF solution of phenyl disulfide (20 mg, 0.092 mmol, 1.0 equiv.) was added to a THF solution of **1** (42 mg, 0.092 mmol, 1.0 equiv.) to give a cherry red transparent solution. The volatiles were removed *in vacuo* to give a red solid, which was extracted with toluene (2x2 mL), filtered over Celite, then the solution layered with pentane (6 mL) and placed cooled to -35°C for 5 days to give red-orange crystals, which were decanted. The crystals were dried *in vacuo* to give 49 mg, 80 % yield of **3SPh**.

Crystals suitable for single crystal X-ray diffraction studies were grown by layering a toluene solution of **3SPh** with pentane and holding the vial at -35°C for 1 day.
^{1}H NMR (600.20 MHz, MeCN-d_3): δ = 9.52 (s, 2H, aldimine), 7.97 (d, $^3J_{HH}$ = 12 Hz, 2H, pincer *meta*), 7.71 (t, $^3J_{HH}$ = 12 Hz, 1H, pincer *para*), 6.88 (m, 8H, SPh *ortho* and *meta*), 6.73 (m, 2H, SPh *para*), 1.44 (s, 18H, ^{t}Bu) ppm.
^{13}C{^{1}H} NMR (150.94 MHz, MeCN-d_3): δ = 196.7, 167.8, 149.2, 144.6, 136.7, 134.9, 129.6, 128.3, 123.7, 61.8, 31.5 ppm.
Positive ion ESI-MS found (calculated): $[LBiSPh]^+$ = $[C_{22}H_{28}N_2SBi]^+$ ($[M\text{-}SPh]^+$) = m/z 561.17706 (561.17717).
M.p. The compound does not melt, but begins to visibly decompose at 150 °C.

2.5 Synthesis of phthalimide-bismuthine (**3Phth**)

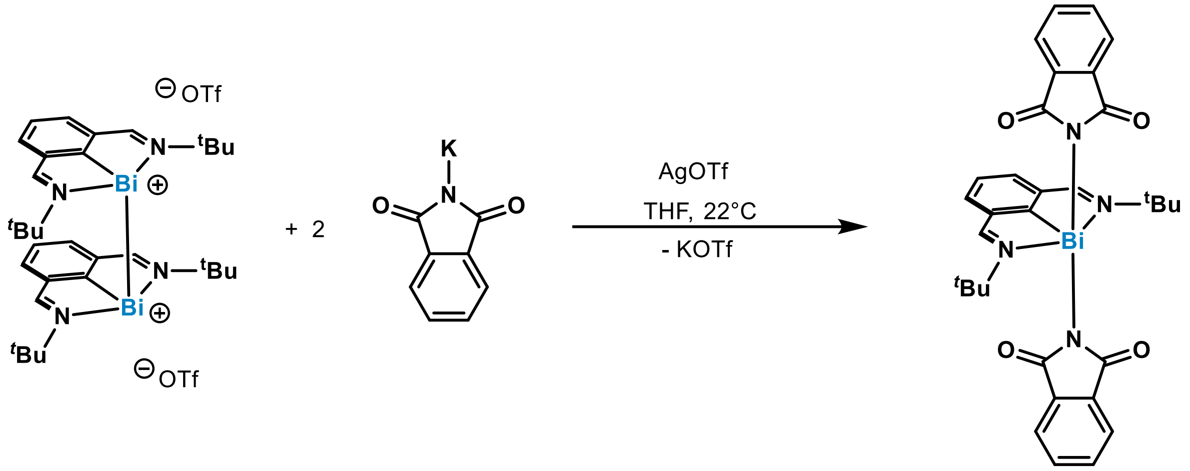

2 **[K][C$_8$H$_4$NO$_2$]** **3Phth**

THF (5 mL) was added to solid **2** (98 mg, 0.082 mmol, 1.0 equiv.), potassium phthalimide (63 mg, 0.34 mmol, 4.1 equiv.) and silver triflate (25 mg, 0.099 mmol, 1.2 equiv.) in a vial covered in Al foil and stirred for 3 days. The resulting reaction mixture was green with colorless precipitate. The solution was filtered over Celite, then the solvent was removed *in vacuo* and the residue was washed with pentane (3 x 5 mL), then redissolved in chlorobenzene (3 mL) and filtered over Celite. The solvent was removed *in vacuo* and the residue was redissolved in THF (3 mL), then this solution was layered with pentane (15 mL) and the vial cooled to -35°C for one week to give colorless crystals. The crystals were decanted and dried *in vacuo* to give 17 mg, 14% yield of **3Phth**.

Crystals suitable for single crystal X-ray diffraction studies were grown by layering a THF solution of **3Phth** with pentane at 22°C for 3 days.

^{1}H NMR (600.22 MHz, DMF-*d$_7$*): δ = 10.19 (s, 2H), 8.41 (d, $^3J_{HH}$ = 7.8 Hz, 2H, pincer *meta*), 7.96 (t, $^3J_{HH}$ = 7.8 Hz, 1H, pincer *para*), 7.61 (m, 4H, phthalimide), 7.55 (m, 4H, phthalimide), 1.46 (s, 18H, ^{t}Bu) ppm.

^{13}C{^{1}H} NMR (300.15 MHz, DMF-*d$_7$*): δ = 199.1, 180.0, 171.0, 152.5, 137.0, 135.5, 132.8, 129.6, 121.6, 60.7, 30.1 ppm.

Positive ion ESI-MS found (calculated): $[C_{24}H_{27}N_3O_2Bi]^+$ ($[M-C_8H_2NO_2]^+$) m/z 598.18978 (598.19018).

M.p. The compound does not melt, or visibly decompose below 250 °C.

2.6 Synthesis of phthalimide-triflate-bismuthine (**3Phth-OTf**)

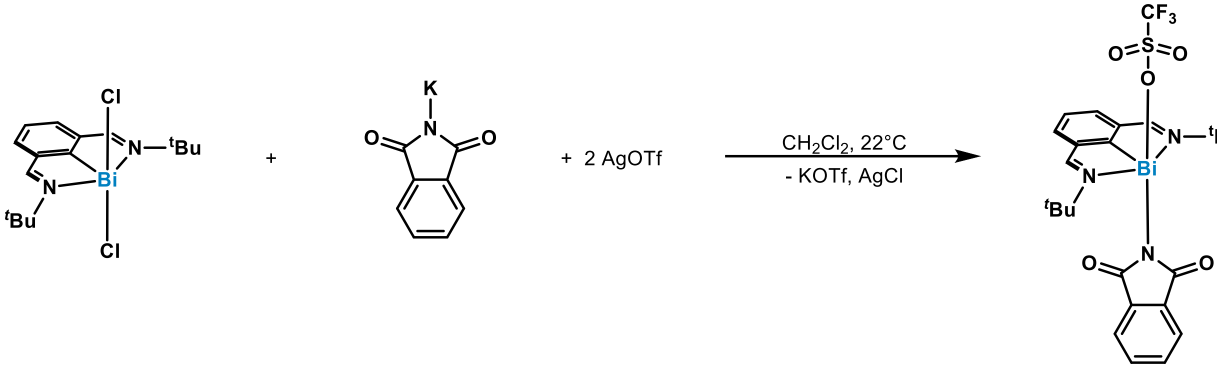

3Cl **[K][$C_8H_4NO_2$]** **3-Phth-OTf**

Compound **3Cl** (100 mg, 0.190 mmol, 1.0 equiv), potassium phthalimide (35 mg, 0.19 mmol, 1.0 equiv) and silver triflate (103 mg, 0.400 mmol, 2.1 equiv) were charged into a 20 mL screw cap vial covered in aluminum foil, then dichloromethane (3 mL) was added and the mixture was stirred for three days. The resulting yellow solution with white precipitate was filtered over Celite, then the solvent was removed *in vacuo*. The residue was redissolved in acetonitrile (2 mL) and the solution layered with diethyl ether (15 mL), then cooled to -35°C for three days to give pale yellow crystals. The crystals were decanted, then dried *in vacuo* to give 84 mg, 59% yield of **3-Phth-OTf**.

Crystals suitable for single crystal X-ray diffraction studies were grown by vapor diffusion of diethyl ether into a MeCN solution of **3-Phth-OTf** at -35°C.
^{1}H NMR (600.20 MHz, MeCN-d_3): δ = 10.01 (s, 2H), 8.42 (d, $^3J_{HH}$ = 7.8 Hz, 2H), 8.05 (t, $^3J_{HH}$ = 7.5 Hz, 1H), 7.70 (m, 2H), 7.65 (m, 2H), 1.41 (s, 18H) ppm.
^{13}C{^{1}H} NMR (150.94 MHz, MeCN-d_3): δ = 196.8, 177.0, 170.7, 152.2, 137.0, 136.0, 135.0, 131.9, 123.7, 121.8 (q, $^1J_{CF}$ = 321.5 Hz), 62.1, 30.5 ppm.
^{19}F NMR (564.72 MHz, MeCN-d_3): δ = -79.3 ppm.
Positive ion ESI-MS found (calculated): $[C_{24}H_{27}N_3O_2Bi]^+$ ($[M-CF_3SO_3]^+$) m/z 598.18992 (598.19018).
Negative ion ESI-MS found: $[CO_3F_3S]^-$ ($[OTf]^-$) m/z 148.952560 (148.952578).
M.p. The compound does not melt, or visibly decompose below 250 °C.

2.7 Synthesis of mesylate-bismuthine (**3OMs**)

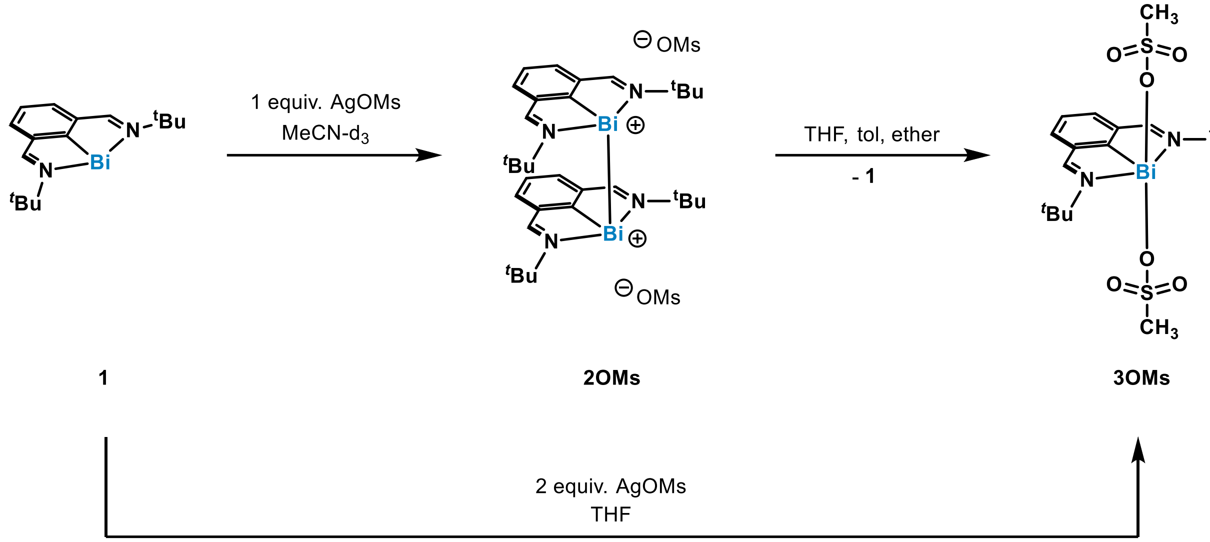

Solid **1** (71 mg, 0.16 mmol, 1.0 equiv.) and silver methanesulfonate (60 mg, 0.30 mmol, 1.9) were combined and dissolved in THF (3 mL) and MeCN (2 mL) to give a dark teal solution, which gradually changed to pale green, accompanied by a black precipitate after stirring for 18 h at 22°C. Diethyl ether (20 mL) was added and the mixture was cooled to -35°C for 18 h to precipitate the product. The supernatant was decanted and the solids were redissolved in MeCN (3 mL), the solution filtered over Celite, then layered with diethyl ether (8 mL) and cooled to -35°C for 3 days. A beige precipitate formed, the supernatant was decanted, and the solids dried *in vacuo* to give 42 mg, 45% yield of **3OMs**.

Crystals suitable for single crystal X-ray diffraction studies were grown by vapor diffusion of a THF solution of **3OMs** into toluene at 22°C.
^{1}H NMR (600.20 MHz, MeCN-*d*$_3$): δ = 10.13 (s, 2H, aldimie), 8.43 (d, $^3J_{HH}$ = 7.2 Hz, 2H, pincer *meta*), 8.01 (t, $^3J_{HH}$ = 7.5 Hz, 1H, pincer *para*), 2.47 (s, 6H, methanesulfonate), 1.57 (s, 18H, ^{t}Bu) ppm.
^{13}C{^{1}H} NMR (150.94 MHz, MeCN-*d*$_3$): δ = 170.5, 152.1, 137.3, 131.8, 62.0, 39.2, 30.9 ppm.
Positive ion ESI-MS found (calculated): $[C_{16}H_{23}N_2Bi]^{2+}$ ($[M\text{-}2(MeSO_3)]^{2+}$) m/z 226.08255 (226.08271) (trace) and $[C_{17}H_{26}N_2O_3SBi]^+$ ($[M\text{-}MeSO_3]^+$) m/z 547.14610 (547.14626).
Negative ion ESI-MS found: $[CO_3H_3S]^-$ ($[M]^-$) m/z 94.98.
M.p. The compound does not melt, or visibly decompose below 250 °C.

2.8 Synthesis of triflate-bismuthine (**3OTf**)

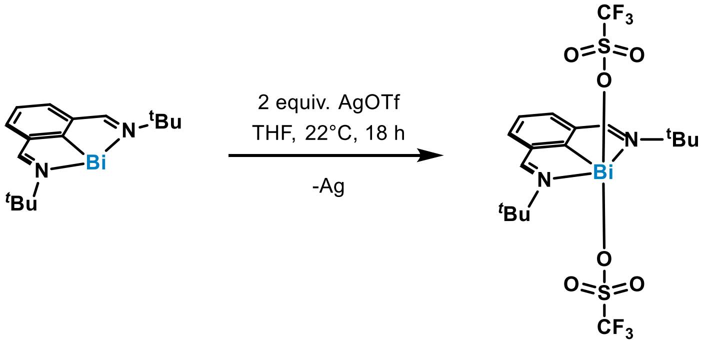

1 3OTf

THF (3 mL) was added to solid **1** (42 mg, 0.092 mmol, 1.0 equiv) and silver triflate (47 mg, 0.18 mmol, 2.0 equiv) to give a red and black suspension, which was stirred overnight to give a pale yellow solution with black precipitate. The solution was filtered over Celite, then pentane (15 mL) was added to precipitate the product. The suspension was cooled to -35°C for 24 hours, then the supernatant was decanted and the solids dried *in vacuo* to give 69 mg, 99% yield of **3OTf** as a beige solid.

Crystals suitable for single crystal X-ray diffraction studies were grown by vapor diffusion of a dichloromethane solution of **3OTf** into THF at -35°C.
^{1}H NMR (600.20 MHz, MeCN-d_3): δ = 10.40 (s, 2H), 8.62 (d, $^{3}J_{HH}$ = 7.2 Hz, 2H), 8.14 (t, $^{3}J_{HH}$ = 7.2 Hz, 1H), 1.55 (s, 18H) ppm.
^{13}C{^{1}H} NMR (150.94 MHz, MeCN-d_3): δ = 215.8, 171.9, 153.2, 138.2, 133.2, 120.6 (quartet, $^{1}J_{FC}$ = 312.8 Hz), 62.6, 30.5 ppm.
^{19}F NMR (564.72 MHz, MeCN-d_3): δ = -79.0 ppm.
Positive ion ESI-MS found (calculated): $[C_{16}H_{23}N_2Bi]^{2+}$ ($[M]^{2+}$) m/z 226.08263 (226.08271).
Negative ion ESI-MS found: $[CO_3F_3S]^{-}$ ($[M]^{-}$) m/z 148.95.
M.p. The compound does not melt, or visibly decompose below 250 °C.

2.9 Tabulated Diagnostic NMR data

Table S1: Pincer ligand ^{1}H NMR chemical shifts and coupling constants for Bi(III) complexes (**3X**) bearing different X-type ligands in MeCN-d_3.

Compound	δ_H *aldimine (ppm)*	δ_H *meta (ppm)*	$^3J_{HH}$ *(Hz)*	δ_H *para (ppm)*	$^3J_{HH}$ *(Hz)*	δ_H ^{t}*Bu (ppm)*
**3SPh**	9.52	7.97	12	7.71	12	1.44
**3OAc**	9.66	8.12	8	7.78	8	1.33
**3F**	9.69	8.20	8	7.87	8	1.45
**3OPh**	9.73	8.06	8	7.72	8	1.47
**3Br**	9.76	8.31	6	7.96	6	1.55
**3Cl**	9.81	8.31	8	7.94	8	1.53
**3OBz**	9.83	8.23	8	7.86	8	1.52
**3Phth-OTf**	10.01	8.42	8	8.05	8	1.41
**3OMs**	10.13	8.43	7	8.01	8	1.57
**3Phth**	10.19	8.41	8	7.96	8	1.46
**3OTf**	10.40	8.62	7	8.14	7	1.55

Table S2: ^{1}H NMR chemical shifts and coupling constants for **1** in benzene-d_6, MeCN-d_3, THF-d_8, toluene-d_8 and DMF-d_7. *Overlaps with residual solvent signal.

Solvent	δ_H *aldimine (ppm)*	δ_H *meta (ppm)*	$^3J_{HH}$ *(Hz)*	δ_H *para (ppm)*	$^3J_{HH}$ *(Hz)*	δ_H ^{t}*Bu (ppm)*
benzene-d_6	9.58	7.86	8	7.15*	8	1.40
MeCN-d_3	9.87	8.04	8	7.20	8	1.57
THF-d_8[1]	9.82	7.95	8	7.09	7	1.58
toluene-d_8	9.54	7.79	7	7.05*	-*	1.40
DMF-d_7	10.03	8.13	7	7.20	7	1.58

3. Reduction and Comproportionation Routes to 2OTf

We have previously reported the synthesis of **2OTf** by oxidation of **1** with either SF_6, or ferrocenium triflate [Fc][OTf].[6] Here we demonstrate that **2OTf** can be made by reduction of the Bi(III) species (**3Cl**) using either cobaltocene or **1** as reducing agent, in the presence of TlOTf to scavenge the coordinating chloride anions from solution. This reductive method streamlines the synthetic process by avoiding reduction from **3Cl** to **1**, then oxidation to **2OTf**. The drawbacks of these routes is the use of toxic thallium and the mixture of [Cp_2Co][OTf] and **2OTf** is difficult to separate from the reaction mixture. These reactions are however a good proof of principle for this work.

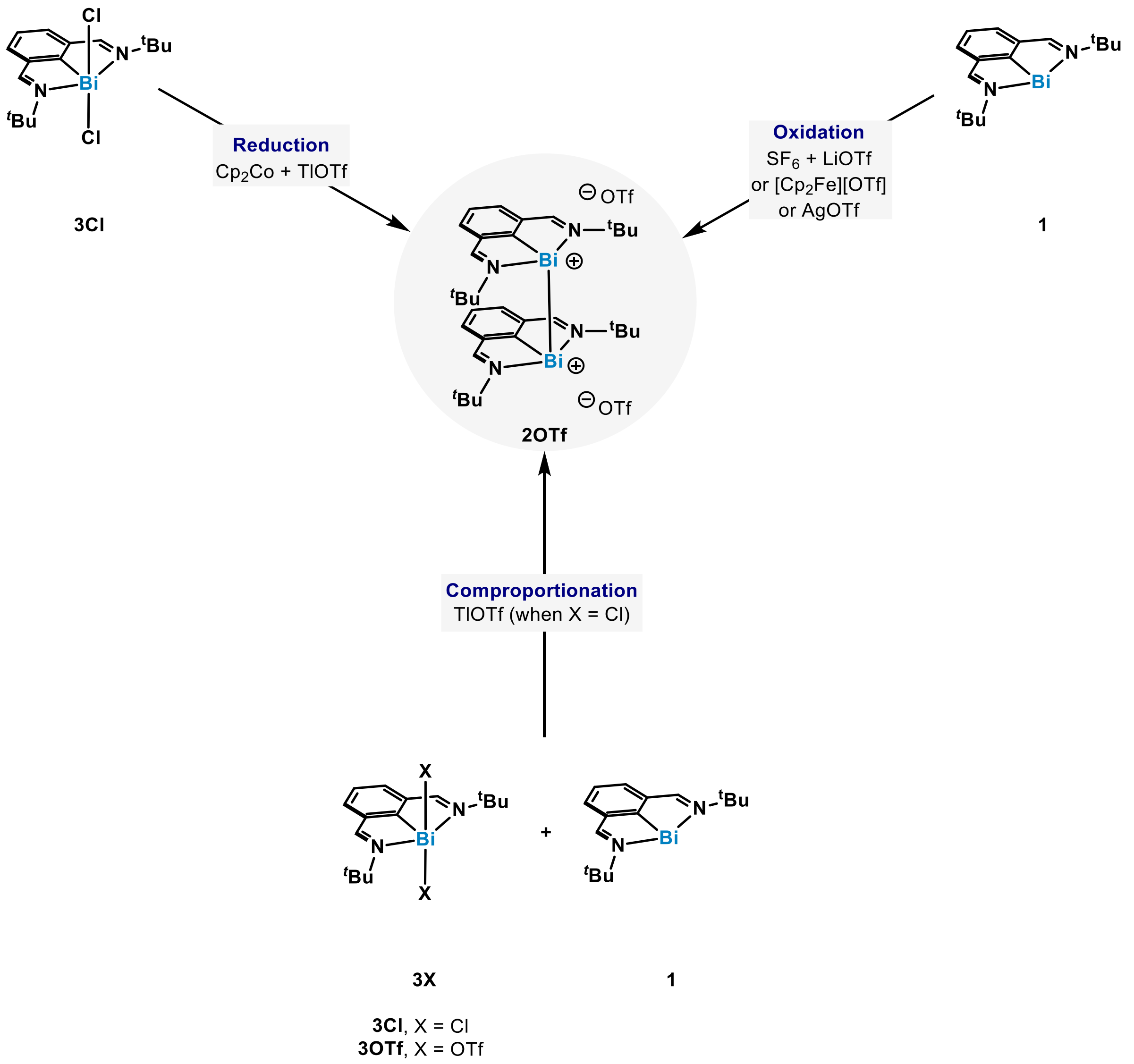

Reductive method: MeCN-d_3 (1 mL) was added to a mixture of solid cobaltocene (9 mg, 0.05 mmol, 1 equiv.), **3Cl** (23 mg, 0.043 mmol, 1.0 equiv.), thallium triflate (44 mg, 0.12 mmol, 2.8 equiv.) and 1,3,5-trimethoxybenzene (15 mg, 0.091 mmol, 2.1 equiv.). The mixture was shaken to

give a yellow-brown solution with yellow precipitate, then filtered over Celite into an NMR tube for analysis. The NMR yield of **2OTf** was determined to be >95% relative to the internal standard.

Comproportionation and salt metathesis method: Solid **1Cl** (29 mg, 0.055 mmol, 1.3 equiv.), **1** (18 mg, 0.041 mmol, 1.0 equiv.), thallium triflate (31 mg, 0.089 mmol, 2.2 equiv.) and 1,3,5-trimethoxybenzene (15 mg, 0.087 mmol, 2.1 equiv.) were combined in a vial. MeCN-d_3 (0.7 mL) was added and the contents of the vial stirred to immediately give a red solution and yellow precipitate. An aliquot of the reaction mixture revealed an NMR yield of >95% for **2OTf** relative to the internal standard.

Comproportionation method: A stock solution of **1** (0.062 mmol, 0.031 M, 0.4 mL, 1 equiv.) and 1,3,5-trimethoxybenzene (0.06 mmol, 0.03 M, 1 equiv.) in MeCN-d_3 was added to a stock solution of **3OTf** (0.062 mmol, 0.031 M, 0.4 mL, 1 equiv.) and 1,3,5-trimethoxybenzene (0.06 mmol, 0.03 M, 1 equiv.) in MeCN-d_3 in an NMR tube to give a yellow solution. Inspection of the ^{1}H NMR spectrum revealed the presence of **2OTf** in 90% NMR yield relative to the internal standard.

Oxidative method: A MeCN-d_3 solution of **1** (7 mg, 0.1 mmol, 1 equiv.) was added to solid silver triflate (4 mg, 0.1 mmol, 1 equiv.) to give a yellow-brown solution and a mirror. A MeCN solution of 1,3,5-trimethoxybenzene was added (0.015 mmol, 0.021 M, 693 μL) and the mixture was filtered into an NMR tube for analysis. The NMR yield of **2OTf** was determined to be 82% relative to the internal standard.

4. Preparation of Bi(II) Salts

In this study, we found that excess equivalents of $[BF_4]^-$, or $[OTs]^-$, $[OMs]^-$ and $[NO_3]^-$ salts were required to disproportionate **2OTf**. We suspected that the Bi(II) salts could be accessible via oxidation of **1** with the corresponding silver salts.

4.1 General Synthesis of Bi(II) salts (**2X**)

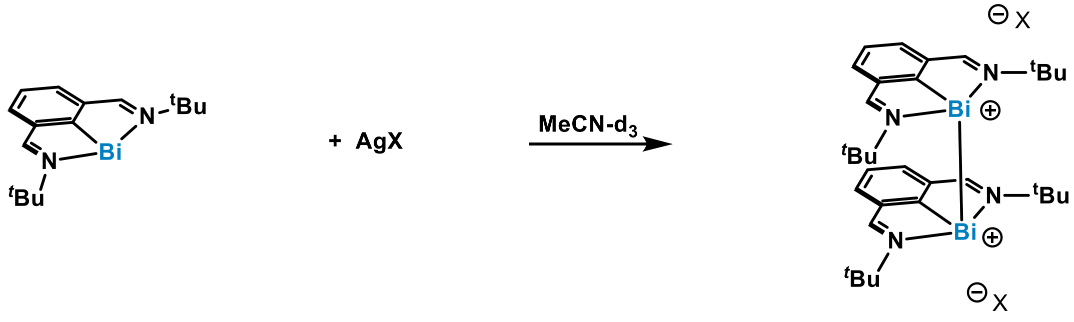

1

2X

2OTs; X = $[OTs]^-$, 94 % (NMR yield)
2OMs; X = $[OMs]^-$, >95 % (NMR yield)
2NO$_3$; X = $[NO_3]^-$, 94 % (NMR yield)
2BF$_4$; X = $[BF_4]^-$, >95 % (NMR yield)
2PF$_6$; X = $[PF_6]^-$, >95 % (NMR yield)
2SbF$_6$; X = $[SbF_6]^-$, >95 % (NMR yield)

An MeCN-d_3 dark teal stock solution of **1** (0.7 mL, 0.01 mmol, 0.016 M, 1 equiv) and 1,3,5-trimethoxybenzene (0.1 mmol, 0.017 M, 1 equiv) was added to four different tubes containing AgX (0.01 mmol, 1 equiv). Black precipitate formed and for $AgBF_4$, $AgPF_6$, $AgSbF_6$, AgOMs and AgOTf, there was a color change to yellow-brown, while $AgNO_3$ gave a color change to green-brown. An NMR spectrum was collected for an aliquot of the reaction mixture and the NMR yields were determined by integration relative to the internal standard.

Compounds **2OTs**, **2OMs** and **2NO$_3$** were unstable to anti-solvents and could not be isolated by recrystallization.

Figure S1: Stacked ^{1}H NMR spectra (MeCN-d_3) showing the aromatic region for reaction mixture aliquots where **1** was combined with AgOTf (300 MHz, 25°C), $AgPF_6$ (300 MHz, 25°C), $AgSbF_6$ (300 MHz, 25°C), $AgBF_4$ (600 MHz, -40°C), $AgNO_3$ (600 MHz, -40°C), AgOTs (300 MHz, 25°C), AgOMs (300 MHz, 25°C).

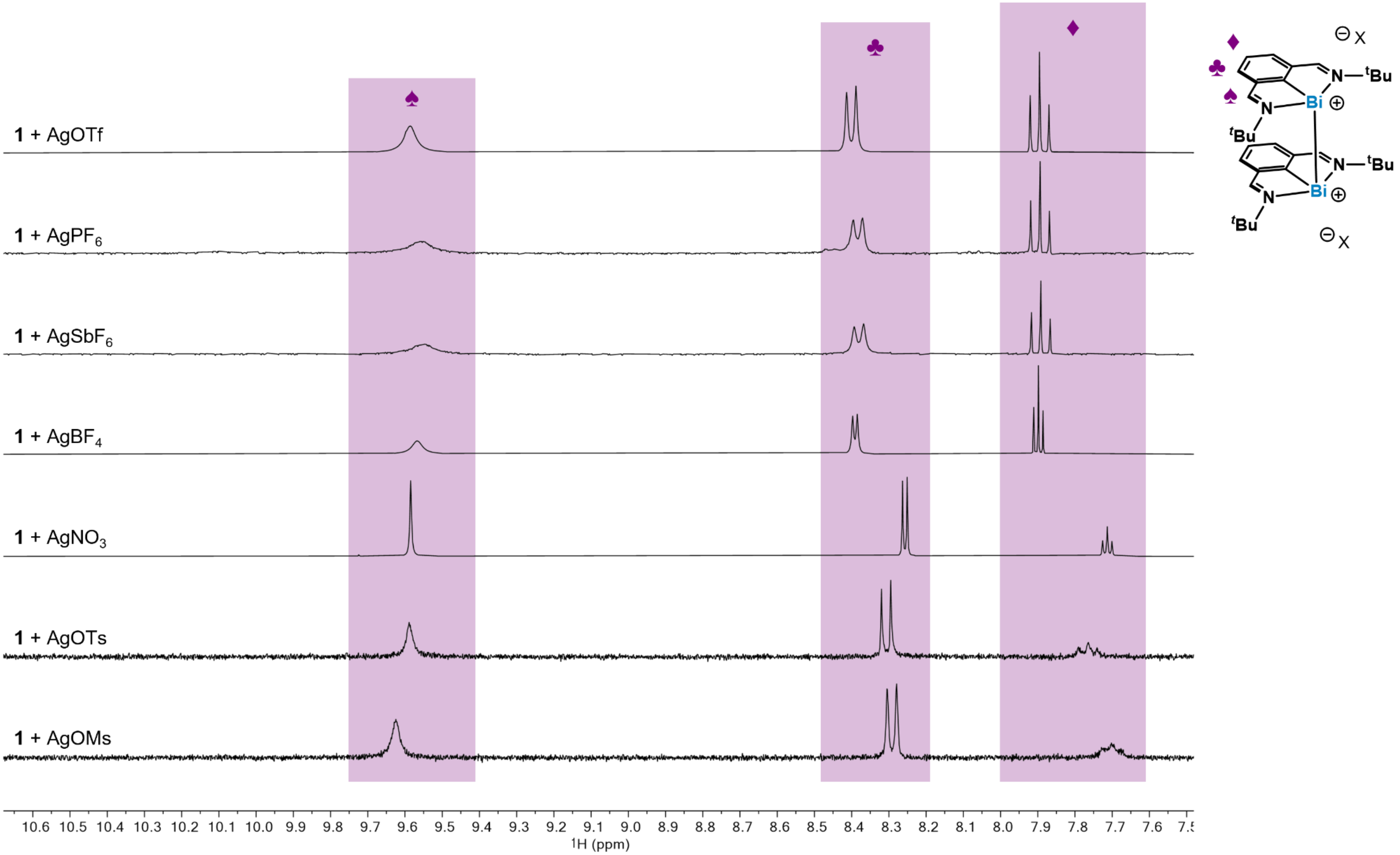

4.2 Synthesis of tetrafluoroborate Bi(II) salt (**2BF4**)

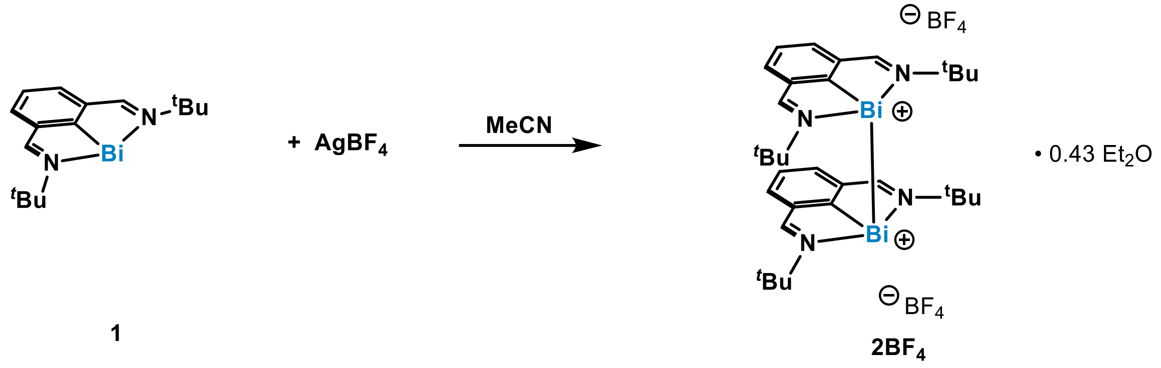

An MeCN solution (3 mL) of silver tetrafluoroborate (40 mg, 0.21 mmol, 1.1 equiv.) was added to solid **1** (87 mg, 0.19 mmol, 1.0 equiv.) to give a yellow-brown solution with black precipitate. The solution was stirred for 18 hours before being filtered over Celite. The solution was then layered with diethyl ether (15 mL) and cooled to -35°C for 4 days to give red crystals. The crystals were decanted and dried *in vacuo* at 60°C for 25 h to give 65 mg, 63% yield **2BF4**. Even after drying, the sample contained 43% Et_2O as determined by ^{1}H NMR spectroscopy and this was accounted for in the elemental analysis.

Crystals suitable for single crystal X-ray diffraction studies were grown by layering an acetonitrile solution of **2BF4** with THF and allowing to stand at room temperature for one week.
^{1}H NMR (600.20 MHz): δ = 9.56 (br s, 4H), 8.39 (d, $^3J_{HH}$ = 7.2 Hz, 4H), 7.90 (t, $^3J_{HH}$ = 7.5 Hz, 2H), 1.28 (br s, 36H) ppm.
^{11}B NMR (192.57 MHz): δ = -1.2 ppm.
^{13}C{^{1}H} NMR (150.94 MHz): δ = 185.4, 171.0, 150.7, 138.5, 132.0, 62.5, 31.6 ppm.
^{19}F NMR (564.72 MHz): δ = -151.6 ([$^{10}BF_4$]$^-$), -151.7 ([$^{11}BF_4$]$^-$) ppm.
Elemental Analysis found (calculated) for $C_{32}H_{46}B_2N_4F_8Bi_2$•0.43($C_4H_{10}O$): C 36.57% (36.48%), H 4.55% (4.57%), N 5.04% (5.05%).
M.p. The compound does not melt, but begins to visibly decompose at 214 °C.

5. Anion Screening for Disproportionation of 2OTf

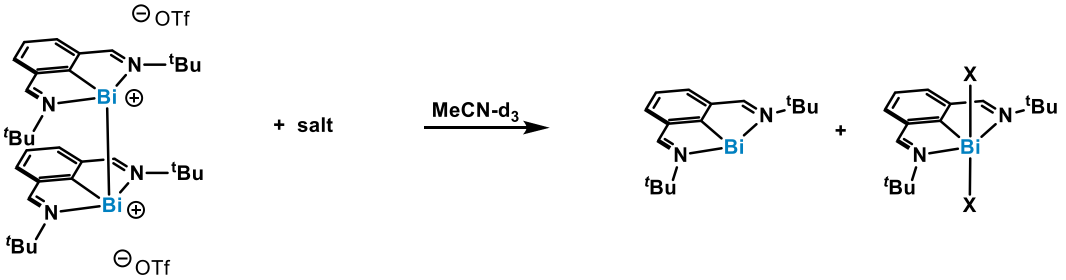

2OTf **1** **3X**

Entry	salt	equiv.	Colour	Disproportionation?	NMR yield 1	NMR yield 3X
1	$[NBu_4][Cl]$	0.5	green	yes	-	26%
2	$[NBu_4][Cl]$	1	dark brown	yes	-	41%
3	$[NBu_4][Cl]$	2	dark teal	yes	>95%	>95%
4	$[NBu_4][Cl]$	10	dark teal	yes	95%	>95%
5	$[Cp_2Co][F]$	2	dark teal	yes	95%	>95%
6	$[NBu_4][Br]$	2	dark teal	yes	89%	>95%
7	$[NBu_4][I]$	2	dark teal	yes	93%	-
8	$[NBu_4][Ph_3SiF_2]$	2	dark teal	yes	-	-
9	$[NBu_4][PF_6]$	2	yellow	no	-	-
10	$[NBu_4][PF_6]$	100	yellow	no	-	-
11	$[NBu_4][BF_4]$	2	dark brown	no	-	-
12	$[NBu_4][BF_4]$	30	dark teal	yes	81%	80%
13	$[NBu_4][BArF]$	2	yellow	no	-	-
14	$[NBu_4][BArF]$	100	yellow	no	-	-
15	$[NBu_4][OTs]$	2	olive green	no	-	-
16	$[NBu_4][OAc]$	2	dark teal	yes	84%	>95%
17	$[NBu_4][OBz]$	2	dark teal	yes	>95%	>95%
18	$[Na][SbF_6]$	2	yellow	no	-	-
19	[Na][BArF]	2	pale brown	no	-	-
20	[Na][OMs]	2	yellow	no	-	-
21	[Na][OPh]	2	dark teal	yes	83%	90%
22	[Na][SPh]	2	dark teal	yes	>95%	>95%
23	K_2CO_3	2	dark teal	yes	81%	-
24	K_3PO_4	2	dark teal	yes	>95%	-
25	$[K][C_8H_4NO_2]$	2	dark teal	yes	89%	>95%
26	$[NBu_4][NO_3]$	2	olive green	no	-	-
27	$[NBu_4][NO_3]$	66	dark teal	yes	82%	90%
28	$[Li][Al(OC(CF_3)_3)_4]$	2	yellow	no	-	-
29	$[NBu_4][ReO_4]$	2	yellow	no	-	-
30	$[NBu_4][ReO_4]$	100	yellow-green	no	-	-
31	$[NBu_4][OMs]$	2	yellow	no	-	-
32	$[NBu_4][OMs]$	18	dark teal	yes	-	-

NMR yiels were determined relative to 1,3,5-trimethoxybenzene internal standard, which was added to the reaction mixture after completion and initial NMR monitoring to ensure that the standard does not affect the outcome.

General Procedure: NMR yields were calculated relative to an internal standard (1,3,5-trimethoxybenzene, or 1,4-difluorobenzene) by ^{1}H NMR spectroscopy using 30 s relaxation time.

[NBu4][Cl]: A stock solution of [NBu4][Cl] in MeCN (100 µL, 0.090 M, 0.0090 mmol, 2.1 equiv.) was added to the stock solution of **2OTf** (0.0043 mmol, 0.007 M, 0.6 mL, 1.0 equiv.) and the

solution changed from yellow to dark teal. The initial ^{1}H NMR spectrum of the reaction mixture revealed sharp signals in line with **1** and **3Cl**. The 1,3,5-trimethoxybenzene solution (0.0042 mmol, 0.021 M, 0.2 mL) was then added and the NMR yield was found to be >95% for **1** and >95% for **3Cl**.

Excess [NBu$_4$][Cl]: A MeCN-*d$_3$* stock solution of **2OTf** (0.0043 mmol, 0.007 M, 0.6 mL, 1.0 equiv.) was added to solid [NBu$_4$][Cl] (11.1 mg, 0.0399 mmol, 9.5 equiv.) and the solution immediately changed from yellow to dark teal. The initial ^{1}H NMR spectrum of the reaction mixture revealed sharp signals in line with **1** and **3Cl**. A stock solution of 1,3,5-trimethoxybenzene in MeCN (0.0042 mmol, 0.021 M, 0.2 mL) was then added and the NMR yield was found to be 95% for **1** and >95% for **3Cl**.

[Cp$_2$Co][F]: A MeCN-*d$_3$* solution of **2OTf** (17 mg, 0.014 mmol, 1.0 equiv.) was added to solid cobaltocenium fluoride[7] (6 mg, 0.03 mmol, 2 equiv.) and 1,3,5-trimethoxybenzene (6 mg, 0.03 mmol, 2 equiv.). The solution immediately changed from yellow to dark teal. The NMR yield was determined at 233 K and found to be 95% for **1** and >95% for **3F**. Volatiles were removed from the reaction mixture and ESI-MS of the residue revealed a signal to corroborate the presence of **3F** in the positive ion mode: $[LBiF]^+ = [C_{16}H_{23}N_2FBi]^+$, found (calculated) m/z 471.16453 (471.16436).

[NBu$_4$][Br]: A MeCN-*d$_3$* stock solution of **2OTf** (0.0043 mmol, 0.007 M, 0.6 mL, 1.0 equiv.) was added to [NBu$_4$][Br] (3.1 mg, 0.0096 mmol, 2.2 equiv.) and a color change from yellow to dark brown to dark teal was observed. The initial ^{1}H NMR spectrum of the reaction mixture revealed broad signals in line with **1** and **3Br**. A stock solution of 1,3,5-trimethoxybenzene in MeCN (0.0042 mmol, 0.021 M, 0.2 mL) was then added and an NMR spectrum recorded at 233 K in order to resolve broad signals. The NMR yield was found to be 89% for **1** and >95% for **3Br**. The solvent was removed from the reaction mixture and ESI-MS of the residue revealed a signal to corroborate the presence of **3Br** in the positive ion mode: $[LBiBr]^+ = [C_{16}H_{23}N_2BrBi]^+$, found (calculated) m/z 531.08477 (531.08431).

[NBu$_4$][I]: A MeCN-*d$_3$* stock solution of **2OTf** (0.0043 mmol, 0.007 M, 0.6 mL, 1.0 equiv.) was added to [NBu$_4$][I] (3.1 mg, 0.0084 mmol, 1.95 equiv.) and a color change from yellow to dark brown to dark teal was observed. The initial ^{1}H NMR spectrum of the reaction mixture revealed overlapping sharp and broad signals in line with **1** and **3I**. A stock solution of 1,3,5-trimethoxybenzene in MeCN (0.0042 mmol, 0.021 M, 0.2 mL) was then added and an NMR spectrum recorded at 233 K in order to resolve broad signals. The NMR yield was found to be 93% for **1**. Yellow single crystals crashed out of the reaction mixture and were determined to have the same unit cell parameters for the reported structure of **3I** (monoclinic, $P2_1/c$, a = 8.5025(4) Å, b = 23.4386(10) Å, c = 10.0665(5) Å, α = 90°, β = 96.205(2)°, γ = 90°, Volume = 1994.37(16) Å^3).[3] The NMR yield of **3I** was not determined due its low solubility in acetonitrile. The solvent was removed from the reaction mixture and ESI-MS of the residue revealed a signal to corroborate the presence of **3I** in the positive ion mode: $[LBiI]^+ = [C_{16}H_{23}N_2IBi]^+$, found (calculated) m/z 579.07064 (579.07045).

[NBu$_4$][I]: A DMF-*d$_7$* solution of [NBu$_4$][I] (5 mg, 0.02 mmol, 2 equiv.) and 1,3,5-trimethoxybenzene (4 mg, 0.02 mmol, 2 equiv.) was added to **2OTf** (8 mg, 0.007 mmol, 1 equiv.)

and the solution became dark teal. The initial ^{1}H NMR spectrum of the reaction mixture was recorded at 25°C and revealed broad signals, which could be somewhat resolved at -40°C to give sets of slightly broadened signals in line with **1** and **3I**. The NMR yield was found to be 91% for **1** and 98% for **3I**. The solvent was removed from the reaction mixture and ESI-MS of the residue revealed a signal to corroborate the presence of **3I** in the positive ion mode: $[LBiI]^+$ = $[C_{16}H_{23}N_2IBi]^+$, found (calculated) m/z 579.07053 (579.07045).

$[NBu_4][Ph_3SiF_2]$: A MeCN-*d_3* stock solution of **2OTf** (0.0043 mmol, 0.007 M, 0.6 mL, 1.0 equiv.) was added to $[NBu_4][Ph_3SiF_2]$ (4.8 mg, 0.0089 mmol, 2.1 equiv.) and a color change from yellow to dark teal was observed. Due to overlapping signals NMR yields could not be obtained, however ESI-MS of revealed a signal to corroborate the presence of **3F** in the positive ion mode: $[LBiF]^+$ = $[C_{16}H_{23}N_2FBi]^+$, found (calculated) m/z 471.16463 (471.16438). The reaction was repeated by combining solid **2OTf** (5 mg, 0.004 mmol, 1 equiv.) and solid $[NBu_4][Ph_3SiF_2]$ (5 mg, 0.009 mmol, 2 equiv.) in a 20 mL volumetric flask and filling with MeCN to make a 0.2 mM reaction mixture. By UV-vis spectroscopy of an aliquot of this reaction mixture, the yield of **1** was determined to be 62 % (A = 0.104103 at 633 nm, calculated C = 0.124 mM for **1**).

$[NBu_4][PF_6]$: A MeCN-*d_3* stock solution of **2OTf** (0.0043 mmol, 0.007 M, 0.6 mL, 1.0 equiv.) was added to $[NBu_4][PF_6]$ (3.4 mg, 0.0088 mmol, 2.0 equiv.) and no color change was observed. No change was observed in the ^{1}H NMR spectrum of the reaction mixture.

Excess $[NBu_4][PF_6]$: A MeCN-*d_3* stock solution of **2OTf** (0.0043 mmol, 0.007 M, 0.6 mL, 1.0 equiv.) was added to solid $[NBu_4][PF_6]$ (168 mg, 0.434 mmol, 100 equiv.) and no color change was observed. No change was observed in the ^{1}H NMR spectrum of the reaction mixture.

$[Na][SbF_6]$: A MeCN-*d_3* stock solution of **2OTf** (0.0043 mmol, 0.007 M, 0.6 mL, 1.0 equiv.) was added to $[Na][SbF_6]$ (2.5 mg, 0.0097 mmol, 2.2 equiv.) and no color change was observed. No change was observed in the ^{1}H NMR spectrum of the reaction mixture.

$[NBu_4][BF_4]$: A MeCN-*d_3* stock solution of **2OTf** (0.0043 mmol, 0.007 M, 0.6 mL, 1.0 equiv.) was added to $[NBu_4][BF_4]$ (2.7 mg, 0.0082 mmol, 1.9 equiv.) and a color change from yellow to red-brown was observed. No change was observed in the ^{1}H NMR spectrum of the reaction mixture.

30 equiv. $[NBu_4][BF_4]$: A MeCN-*d_3* solution of **2OTf** (5 mg, 0.004 mmol, 1.0 equiv.) was added to solid $[NBu_4][BF_4]$ (43 mg, 0.13 mmol, 32 equiv.) and a color change from yellow to dark teal was observed. A MeCN stock solution of 1,3,5-trimethoxybezene (294 μL, 0.0062 mmol, 0.021 M, 1 equiv.) and heteronuclear NMR spectra were recorded at -40°C for an aliquot of the reaction mixture. From integrations relative to the internal standard in the ^{1}H NMR spectrum, the NMR yield of **1** was found to be 81%. Another pincer ligand species could be identified (δ_H = 9.78 (*aldimine*, s, 2H), 8.31 (*meta*, d, 7 Hz, 2H), 7.95 (*para*, t, 7 Hz, 1H) ppm) and the NMR yield was found to be 80%. We speculate that this second set of signals could be ascribed to a Bi(III) species with either two $[BF_4]^-$ counterions, or one $[BF_4]^-$ and one triflate. Lewis acidic Bi cations have been reported to abstract fluoride from $[BF_4]^-$.[8,9] However, in this case we have ruled this out, as we would expect to observe **3F** as a byproduct. The ^{1}H NMR signals of the unassigned species do not match with those for **3F** (*cf.* **3F**: δ_H = 9.69 (*aldimine*, s, 2H), 8.20 (*meta*, d, 8 Hz, 2H), 7.87

(*para*, t, 8 Hz, 1H) ppm). Further, the ^{19}F NMR spectrum lacks any evidence for **3F** (δ_F = -133.4 ppm),[1] showing only signals for triflate (δ_F = -79.6 ppm) and $[BF_4]^-$ (δ_F = -150.7 ($[^{10}BF_4]^-$), -150.8 ($[^{11}BF_4]^-$) ppm). The only species observed in the ^{11}B NMR spectrum is $[BF_4]^-$ (δ_B = -1.3 ppm). Volatiles were removed from the reaction mixture and the color changed from dark teal to purple. No signs of the speculated Bi(III) species could be found in the ESI-MS of the residue. We speculate that this species is only stable in solution and possibly decomposes when solvent is removed.

[NBu₄][BArF]: A MeCN-d_3 stock solution of **2OTf** (0.0043 mmol, 0.007 M, 0.6 mL, 1.0 equiv.) was added to $[NBu_4]$[BArF] (9.4 mg, 0.0085 mmol, 2.0 equiv.) and no color change was observed. No change was observed in the ^{1}H NMR spectrum of the reaction mixture.

Excess $[NBu_4]$[BArF]: Solid **2OTf** (4 mg, 0.003 mmol, 1.0 equiv.), $[NBu_4]$[BArF] (353 mg, 0.319 mmol, 100 equiv.) and 1,3,5-trimethoxybenzene (7 mg, 0.04 mmol, 13 equiv.) were combined and dissolved in MeCN-d_3 (0.6 mL) to give a yellow-orange solution. The only pincer-ligand signals observed in the ^{1}H NMR spectrum of the reaction mixture correspond to **2OTf**.

[Na][BArF]: A MeCN-d_3 stock solution of **2OTf** (0.0043 mmol, 0.007 M, 0.6 mL, 1.0 equiv.) was added to [Na][BArF] (7.5 mg, 0.0085 mmol, 2.0 equiv.) and a color change from yellow to pale brown was observed. No change was observed in the ^{1}H NMR spectrum of the reaction mixture.

$[NBu_4]$[OTs]: A MeCN-d_3 stock solution of **2OTf** (0.0043 mmol, 0.007 M, 0.6 mL, 1.0 equiv.) was added to $[NBu_4]$[OTs] (3.4 mg, 0.0082 mmol, 1.9 equiv.) and a color change from yellow to light olive green was observed. No change was observed in the ^{1}H NMR spectrum of the reaction mixture.

[Na][OMs]: A MeCN-d_3 solution of **2OTf** (14 mg, 0.012 mmol, 1.0 equiv.) was added to solid [Na][OMs] (3 mg, 0.02 mmol, 2 equiv.) and stirred for three days. The solution remained yellow and no change could be observed in the ^{1}H NMR spectrum of the reaction mixture.

2.0 equiv. $[NBu_4]$[OMs]: A MeCN-d_3 solution of $[NBu_4]$[OMs] (7 mg, 0.02 mmol, 2 equiv.) was added to solid **2OTf** (12 mg, 0.0096 mmol, 1.0 equiv.) and 1,3,5-trimethoxybenzene (2 mg, 0.01 mmol, 1 equiv.). The yellow-brown solution was analyzed by ^{1}H NMR spectroscopy and only signals for **2OTf** were observed, indicating no reaction.

20 equiv. $[NBu_4]$[OMs]: A MeCN-d_3 solution of $[NBu_4]$[OMs] (37 mg, 0.11 mmol, 18 equiv.) was added to solid **2OTf** (7 mg, 0.006 mmol, 1.0 equiv.) and 1,3,5-trimethoxybenzene (3 mg, 0.02 mmol, 3 equiv.). The dark teal solution was analyzed by ^{1}H NMR spectroscopy which revealed broad signals at room temperature, which could be partially resolved at -40°C. The broad signals could possibly indicate an equilibrium between **1** and **3OMs**. UV-vis spectroscopy of the reaction mixture confirmed the presence of **1** (λ_{max} = 631 nm).

$[NBu_4]$[OAc]: A MeCN-d_3 stock solution of **2OTf** (0.0043 mmol, 0.007 M, 0.6 mL, 1.0 equiv.) was added to $[NBu_4]$[OAc] (3.0 mg, 0.0099 mmol, 2.3 equiv.) and a color change from yellow to dark teal was observed. The initial ^{1}H NMR spectrum of the reaction mixture revealed broad

signals in line with Bi(I) and Bi(III) species. A stock solution of 1,3,5-trimethoxybenzene in MeCN (0.0042 mmol, 0.021 M, 0.2 mL) was then added and an NMR spectrum recorded at 233 K in order to resolve broad signals. The NMR yield was found to be 84% for **1** and >95% for **3OAc**. The solvent was removed from the reaction mixture and ESI-MS of the residue revealed a signal to corroborate the presence of **3OAc** in the positive ion mode: $[LBiOAc]^+ = [C_{18}H_{26}N_2O_2Bi]^+$, found (calculated) m/z 511.17967 (511.17928).

$[NBu_4][OBz]$: A MeCN-*d_3* stock solution of **2OTf** (0.0043 mmol, 0.007 M, 0.6 mL, 1.0 equiv.) was added to $[NBu_4][OBz]$ (2.7 mg, 0.0088 mmol, 2.0 equiv.) and a color change from yellow to dark teal was observed. The initial ^1H NMR spectrum of the reaction mixture revealed sharp signals in line with **1** and **3OBz**. A stock solution of 1,3,5-trimethoxybenzene in MeCN (0.0042 mmol, 0.021 M, 0.2 mL) was then added and the NMR yield was found to be >95% for **1** and >95% for **3OBz**. The solvent was removed from the reaction mixture and ESI-MS of the residue revealed a signal to corroborate the presence of **3OBz** in the positive ion mode: $[LBiOBz]^+ = [C_{23}H_{28}N_2O_2Bi]^+$, found (calculated) m/z 573.19533 (573.19225).

[Na][OPh]: A THF stock solution of sodium phenoxide (82 μL, 0.15 M, 0.012 mmol, 2 equiv.) was added to a THF (1 mL) suspension of **2OTf** (7 mg, 0.006 mmol, 1 equiv.). The solution was stirred for four hours and a gradual color change from pale yellow to dark teal was observed. The solvent was removed *in vacuo,* then a MeCN stock solution of 1,3,5-trimethoxybezene (294 μL, 0.0062 mmol, 0.021 M, 1 equiv.), MeCN-*d_3* (1 mL) were added and the NMR yield was found to be 83% for **1** and 90% for **3OPh**. The solvent was removed from the reaction mixture and ESI-MS of the residue revealed a signal to corroborate the presence of **3OPh** in the positive ion mode: $[LBiOPh]^+ = [C_{22}H_{28}N_2OBi]^+$, found (calculated) m/z 545.20014 (545.20001).

[Na][SPh]: A MeCN-*d_3* stock solution of **2OTf** (0.0043 mmol, 0.007 M, 0.6 mL, 1.0 equiv.) was added to [Na][SPh] (1.3 mg, 0.0098 mmol, 2.3 equiv.) and a color change from yellow to dark teal was observed. The initial ^1H NMR spectrum of the reaction mixture revealed sharp signals in line with **1** and **3SPh**. A stock solution of 1,3,5-trimethoxybenzene in MeCN (0.0042 mmol, 0.021 M, 0.2 mL) was then added and the NMR yield was found to be >95% for **1** and >95% for **3SPh**. The solvent was removed from the reaction mixture and ESI-MS of the residue revealed a signal to corroborate the presence of **3SPh** in the positive ion mode: $[LBiSPh]^+ = [C_{22}H_{28}N_2SBi]^+$, found (calculated) m/z 561.17719 (561.17717).

K_2CO_3: A MeCN-*d_3* solution of **2OTf** (13 mg, 0.011 mmol, 1.0 equiv.) was added to solid potassium carbonate (4 mg, 0.03 mmol, 3 equiv.) and stirred for three days. The solution changed from yellow to dark teal with white precipitate. The 1,3,5-trimethoxybenzene solution (513 μL, 0.011 mmol, 0.021 M, 1 equiv.) was then added and the NMR yield was found to be 81% for **1**.

K_3PO_4: A MeCN-*d_3* solution of **2OTf** (12 mg, 0.0098 mmol, 1.0 equiv.) was added to solid potassium phosphate (4 mg, 0.02 mmol, 2 equiv.) and stirred for three days. The solution changed from yellow to dark teal with white precipitate. The 1,3,5-trimethoxybenzene solution (466 μL, 0.0098 mmol, 0.021 M, 1 equiv.) was then added and the NMR yield was found to be >95% for **1**. A second pincer ligand-bearing species was formed in 58% NMR yield. The $^{31}P\{^1H\}$ NMR spectrum of the reaction mixture was silent and this second species remains unassigned.

[K][$C_8H_4NO_2$]: A THF solution (2 mL) of potassium phthalimide (2 mg, 0.01 mmol, 2 equiv.) was added to solid **2OTf** (5 mg, 0.004 mmol, 1 equiv.). The colorless solution gradually changed to dark teal over the course of 20 minutes. The reaction was stirred for 18 hours to ensure consumption of **2OTf**, which is sparingly soluble in THF. Volatiles were removed *in vacuo*, then a MeCN stock solution of 1,4-difluorobenzene (27 µL, 0.0043 mmol, 0.16 M, 1 equiv.) and DMF-d_7 (0.6 mL) were added to the residue and the NMR yield was found to be 89% for **1** and >95% for **3Phth**. ESI-MS of the reaction mixture residue revealed a signal to corroborate the presence of **3Phth** in the positive ion mode: $[LBiPhth]^+$ = $[C_{24}H_{27}N_3O_2Bi]^+$, found (calculated) m/z 598.189736 (598.19018).

[NBu_4][NO_3]: A MeCN-d_3 stock solution of **2OTf** (0.004 mmol, 0.007 M, 0.6 mL, 1 equiv.) was added to solid [NBu_4][NO_3] (3 mg, 0.01 mmol, 2 equiv.). The color changed from yellow to olive green, but no change could be observed by ^1H NMR spectroscopy. Further equivalents of [NBu_4][NO_3] (88 mg, 0.29 mmol, 66 equiv.) were added to the reaction mixture and a change to dark teal was observed. A stock solution of 1,3,5-trimethoxybenzene in MeCN (0.005 mmol, 0.021 M, 233 µL) was then added and the NMR yield was found to be 82% for **1** and 90% for a second pincer ligand-bearing species, which could not be assigned.

[Li][Al(OC(CF_3)$_3$)$_4$]: A MeCN-d_3 stock solution of **2OTf** (0.004 mmol, 0.007 M, 0.6 mL, 1 equiv.) was added to solid [Li][Al(OC(CF_3)$_3$)$_4$] (10 mg, 0.010 mmol, 2 equiv.). The solution remained yellow and no change could be observed in the ^1H NMR spectrum of the reaction mixture.

[NBu_4][ReO_4]: A MeCN-d_3 solution of [NBu_4][ReO_4] (8 mg, 0.02 mmol, 2 equiv.) was added to solid **2OTf** (10 mg, 0.01 mmol, 1 equiv.) to give a yellow solution. No change could be observed in the ^1H NMR spectrum of the reaction mixture.

100 equiv. [NBu_4][ReO_4]: A MeCN-d_3 solution of [NBu_4][ReO_4] (164 mg, 0.332 mmol, 100 equiv.) was added to solid **2OTf** (4 mg, 0.003 mmol, 1 equiv.) to give a yellow solution. No change could be observed in the ^1H NMR spectrum of the reaction mixture.

6. Molar Absorptivity Calculations

6.1 Molar Absorptivity of **1**

Table S3: Molar absorptivity (ε) values of **1** and **2OTf** at different wavelengths (λ), calculated by plotting absorbance (A) vs. concentration (c) • path length (l), to give the equation,

$$A = \varepsilon cl + \text{intercept.}$$

Compound	*λ (nm)*	*ε* ($L \cdot mol^{-1} \cdot cm^{-1}$)	*intercept*	R^2
**1**	633	4426.7	-0.0057	0.9991
**1**	477	3817.7	-0.0056	0.9989
**1**	411	2776	-0.005	0.9985
**1**	405	2894.2	-0.0052	0.9985
**1**	310	8037.5	-0.0125	0.9989
**1**	255	32531	-0.0253	0.9996
**2OTf**	411	25109	-0.0377	0.9983
**2OTf**	246	39429	+0.0403	0.9984

Figure S2: UV-vis spectra of different concentrations of **1** in MeCN.

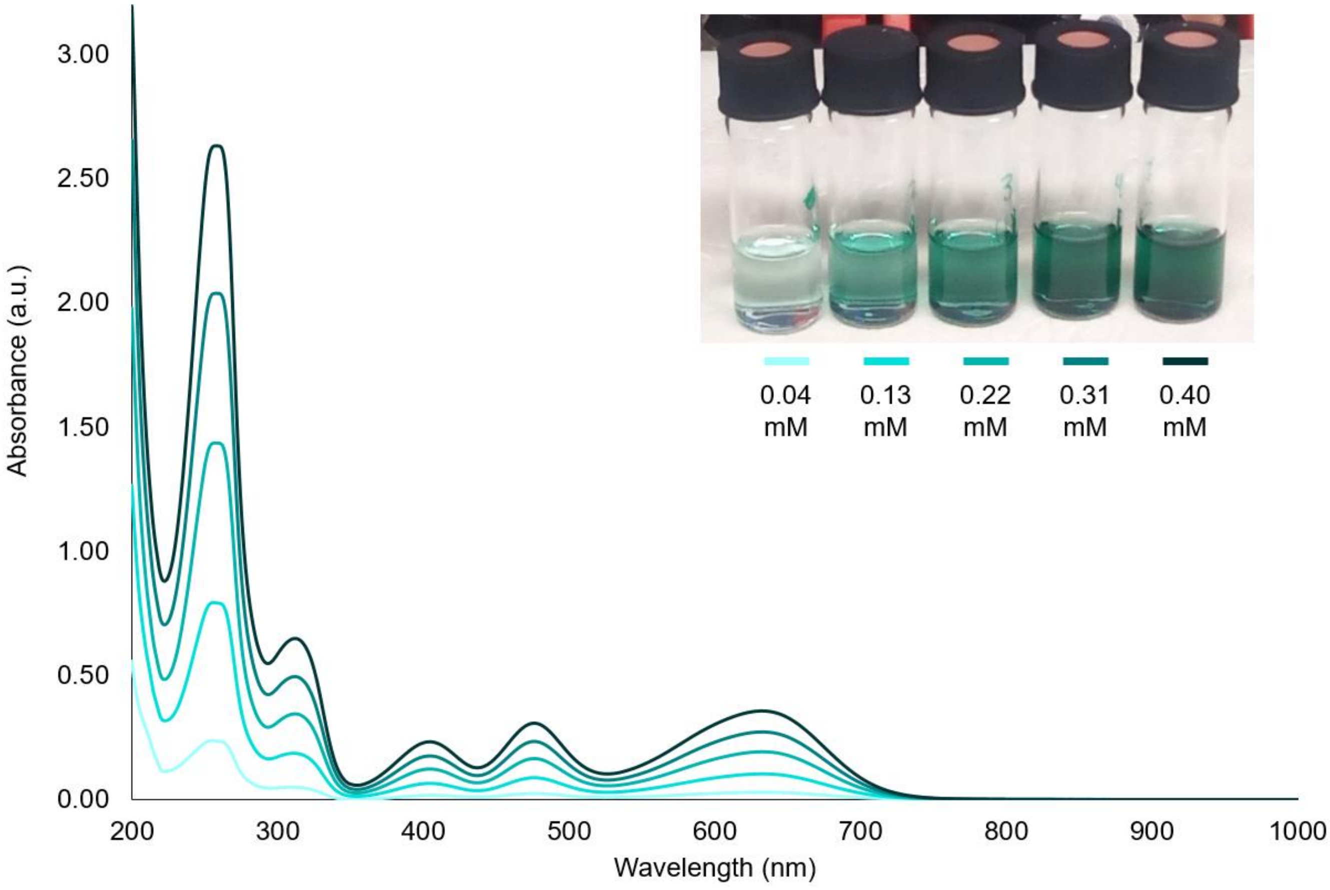

Figure S3: Absorbance vs. concentration • path length plot of **1** to determine molar absorptivity at different wavelengths.

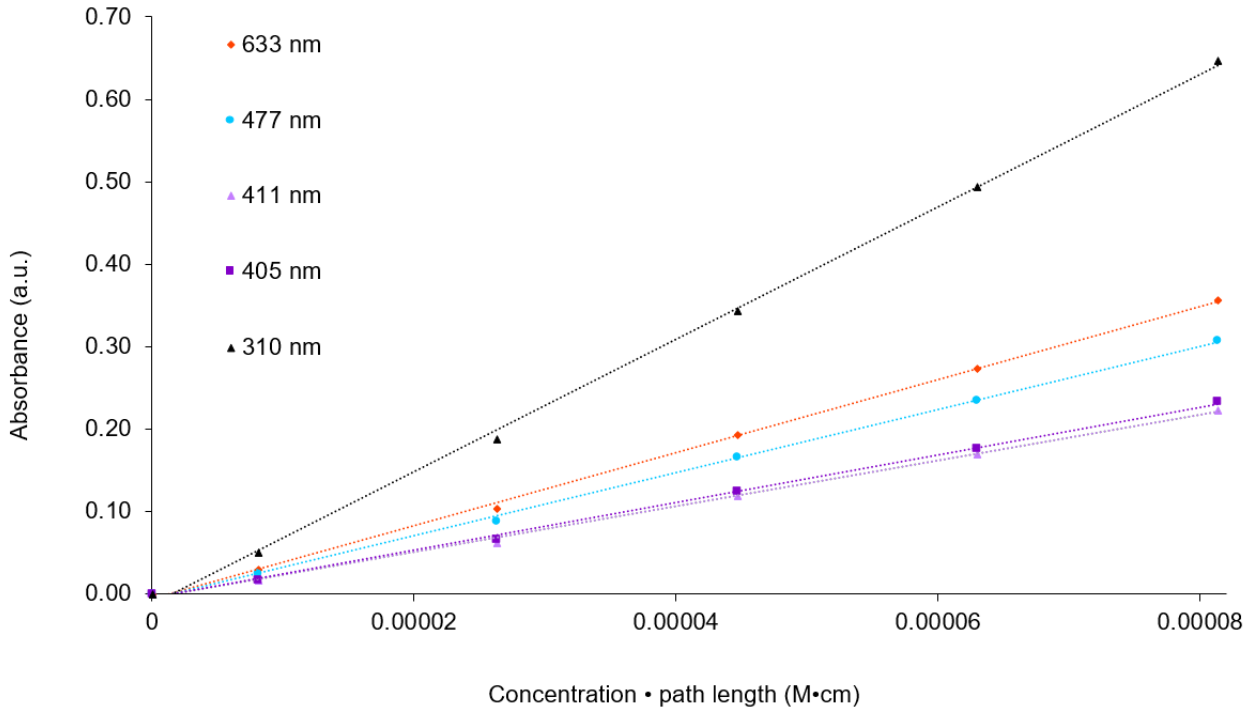

Figure S4: Absorbance vs. concentration • path length plot of **1** to determine molar absorptivity at 255 nm.

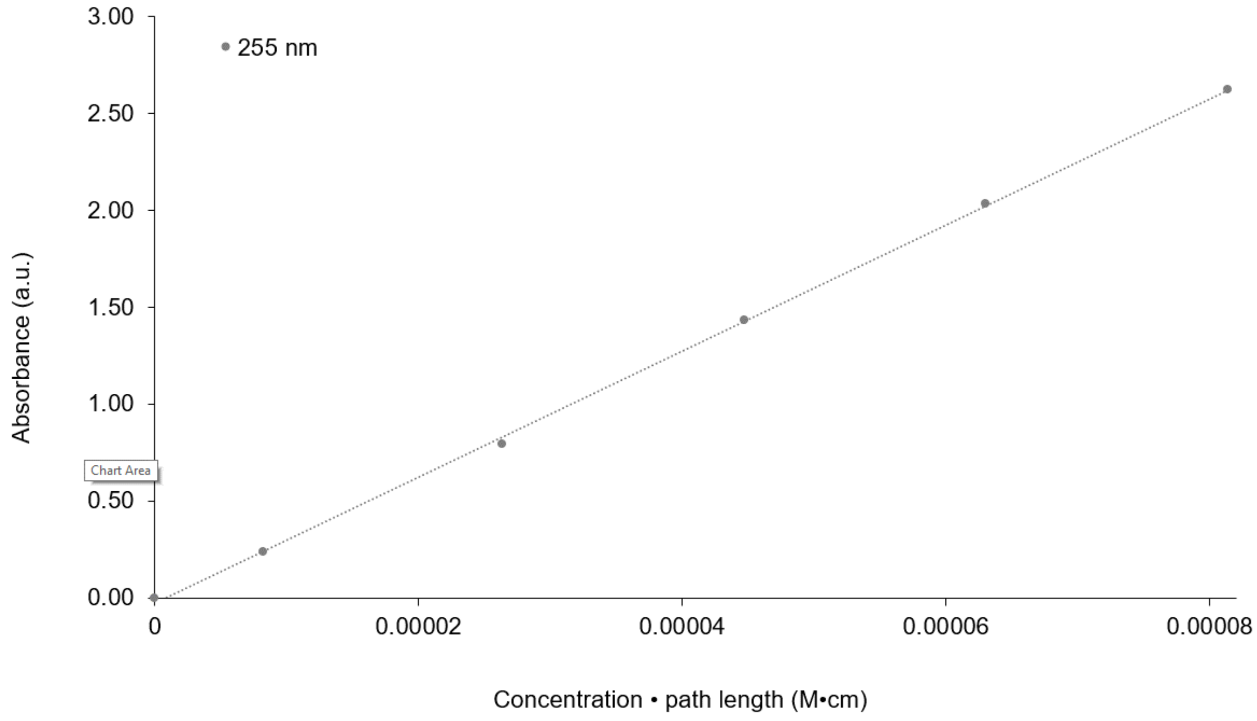

6.2 Molar Absorptivity of **2OTf**

Figure S5: UV-vis spectra of different concentrations of **2OTf** in MeCN.

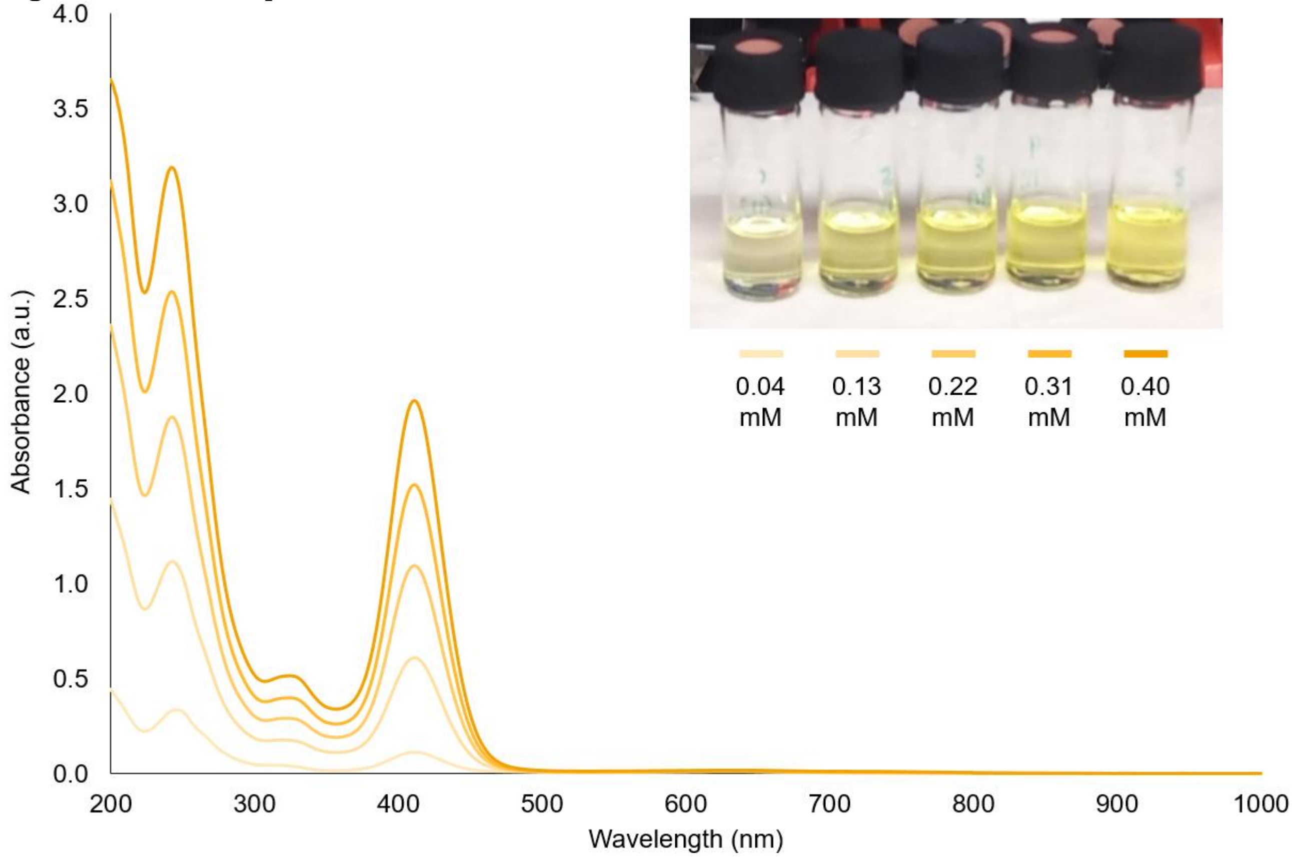

Figure S6: Absorbance vs. concentration • path length plot of **2OTf** to determine molar absorptivity at 411 and 246 nm.

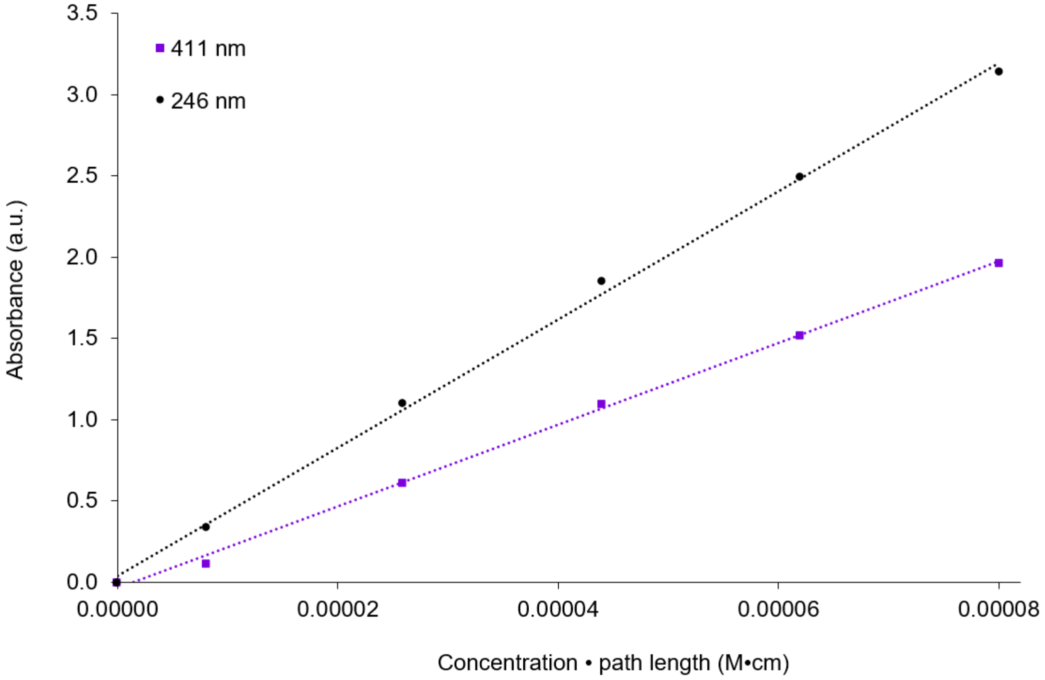

6.3 Calculations for concentration of **1** and **2OTf** in titrations

The absorption at 633 nm ($A_{obs\text{-}633}$) was used to calculate the concentration of **1** ($C_{\mathbf{1}\text{-}633}$), however **1** has multiple absorption bands, including one at 405 nm, which overlaps with the 411 nm band of **2OTf**. This is important to account for in the titrations for the disproportionation of **2OTf**. The concentration of **1** was calculated from the absorption at 633 nm, then the absorption of **1** at 411 nm could be calculated ($A_{\mathbf{1}\text{-}411}$). This absorption could be subtracted from the observed absorption at 411 nm, then the concentration of **2OTf** could be calculated ($C_{\mathbf{2}\text{-}411}$).

$$C_{\mathbf{1}-633} = \frac{A_{obs-633}}{\varepsilon_{\mathbf{1}-633} \bullet l}$$

$$A_{\mathbf{1}-411} = \varepsilon_{\mathbf{1}-411} \bullet C_{\mathbf{1}-633} \bullet l$$

$$C_{\mathbf{2}-411} = \frac{(A_{obs-411} - A_{\mathbf{1}-411})}{\varepsilon_{\mathbf{2}-411} \bullet l}$$

7. Ratio Dependent Disproportionation Reactions

In the stoichiometric studies, we observed that 2.0 equivalents of [NBu_4][BF_4], or [NBu_4][OTs] did not result in disproportionation of **2OTf**, however based on the CV data, we could observe disproportionation. We suspected that the disproportionation for these two salts could be dependent on the number of equivalents of electrolyte used and by titration, we found that an excess of these salts could result in disproportionation. Similar results were found for [NBu_4][OMs] and [NBu_4][NO_3].

Figure S7: UV-vis spectra of **2OTf** (0.26 mM) combined with 0 to 30 equivalents (n) of [NBu_4][BF_4]. Inset photo of colored stock solutions. Two isosbestic points appear at 462 and 351 nm.

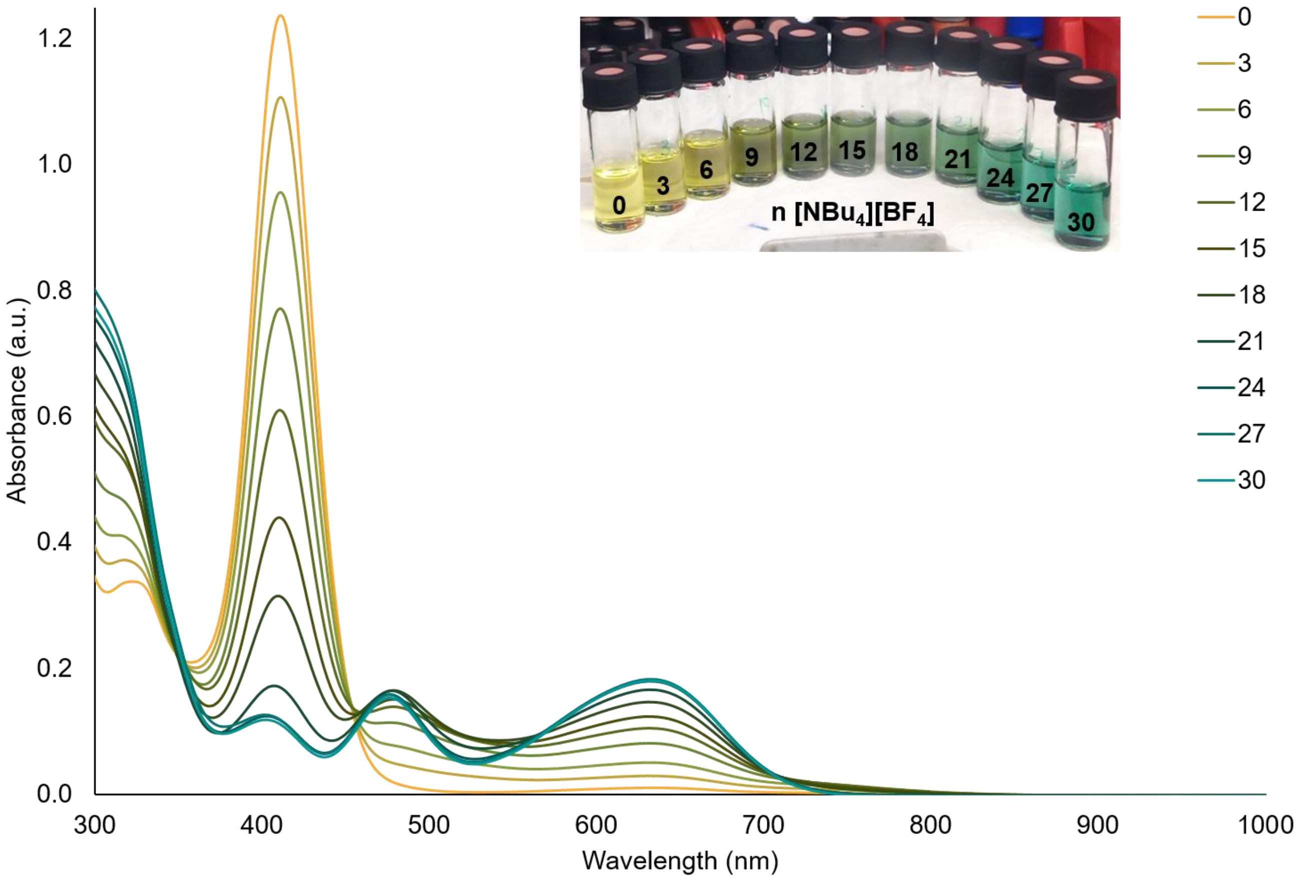

Figure S8: Plot of λ_{max} vs. equivalents $[NBu_4][BF_4]$ showing a hypsochromic shift of the 411 nm absorption of **2OTf** towards the 405 nm absorption of **1** as the species disappear and appear, respectively.

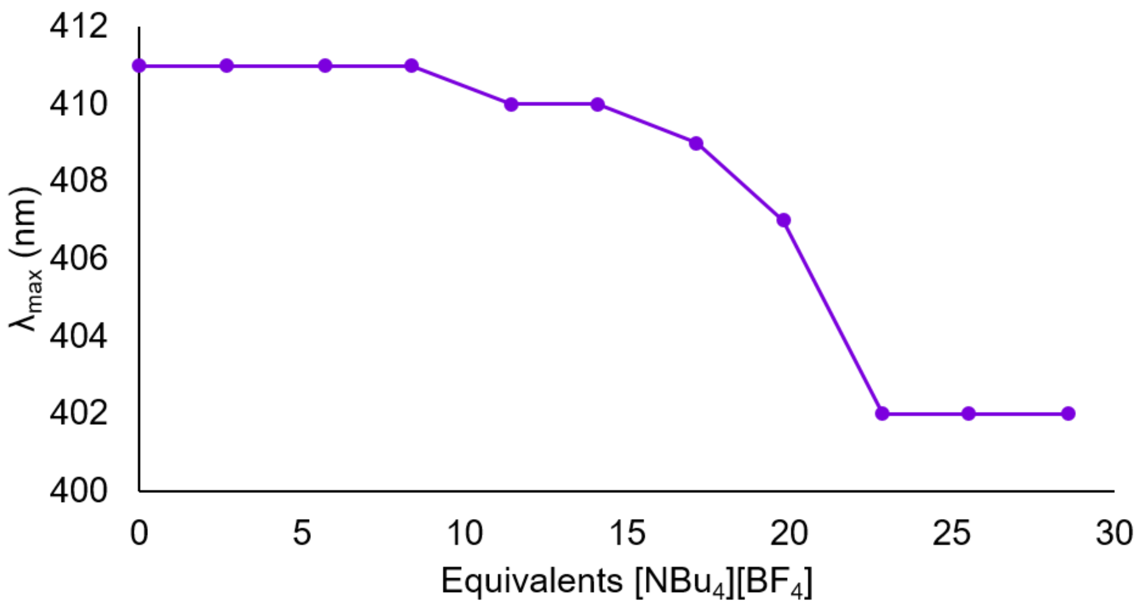

Figure S9: Titration curve for the disproportionation of **2OTf** using $[NBu_4][BF_4]$.

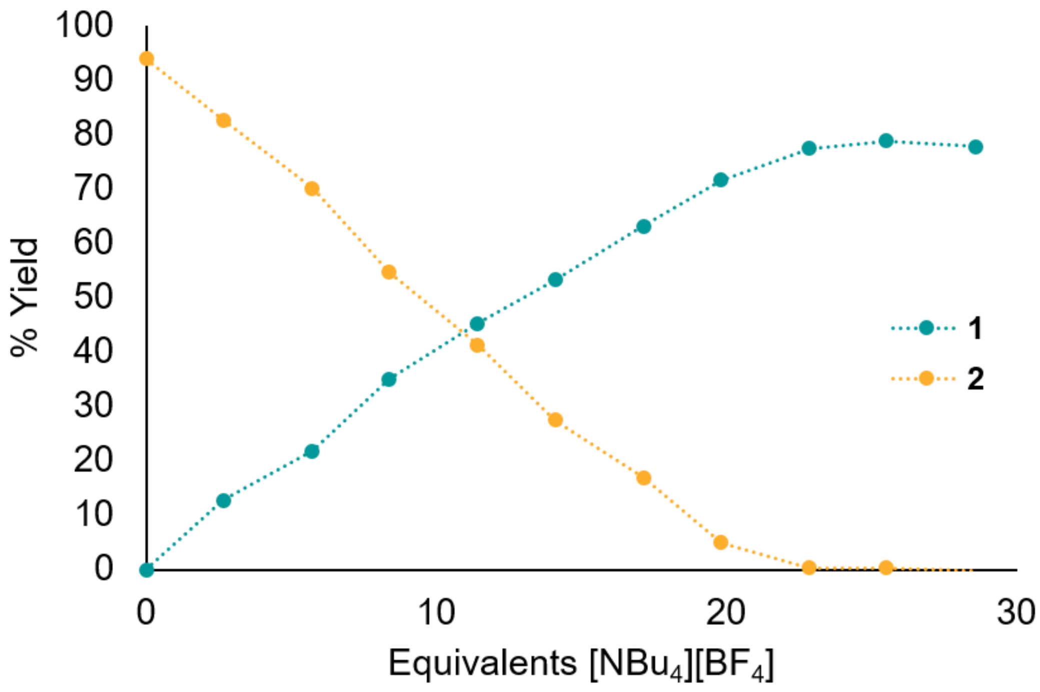

Figure S10: UV-vis spectra of **2OTf** (0.26 mM) combined with 0 to 300 equivalents (n) of $[NBu_4][OTs]$. Inset photo of colored stock solutions. The λ_{max} of **2OTf** (411 nm) undergoes a bathochromic shift to 422 nm with 100 equivalents, indicating the formation of a new species. With the addition of further equivalents of $[NBu_4][OTs]$, the λ_{max} undergoes a hypsochromic shift to 403 nm, in line with **1**.

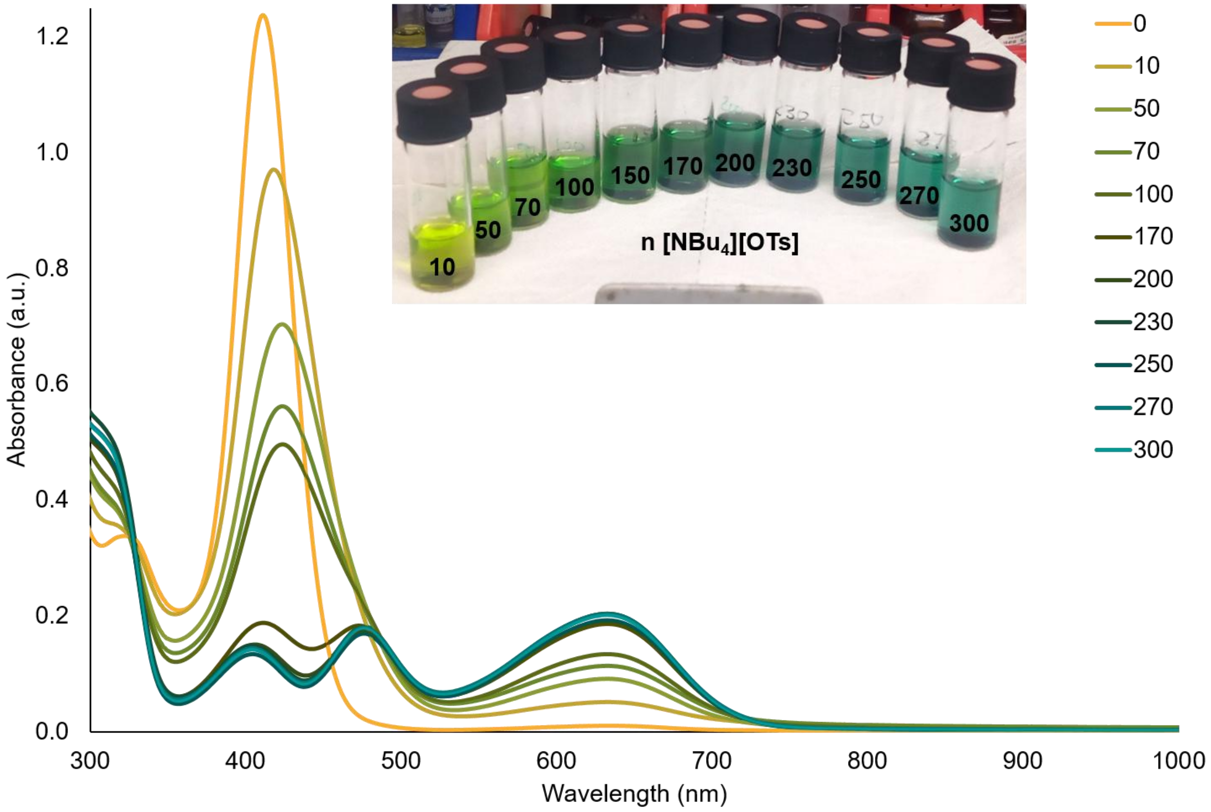

Figure S11: Plot of λ_{max} vs. equivalents $[NBu_4][OTs]$ showing a bathochromic shift of the 411 nm absorption of **2OTf** towards 423 nm at 100 equiv, then a hypsochromic shift to the 205 nm absorption of **1**.

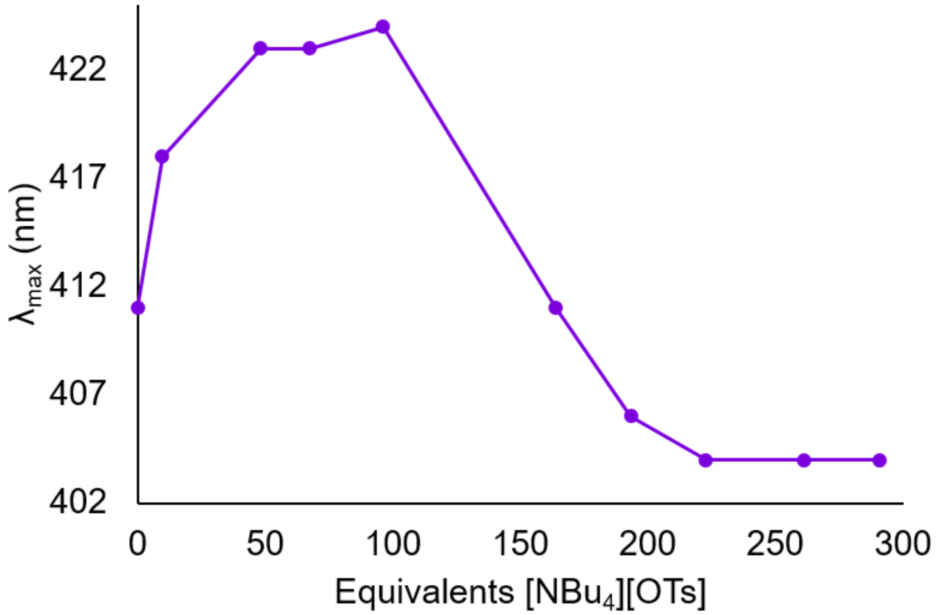

Figure S12: Titration curve for the disproportionation of **2OTf** using $[NBu_4][OTs]$, plotting the yield of **1** vs. equivalents of $[NBu_4][OTs]$.

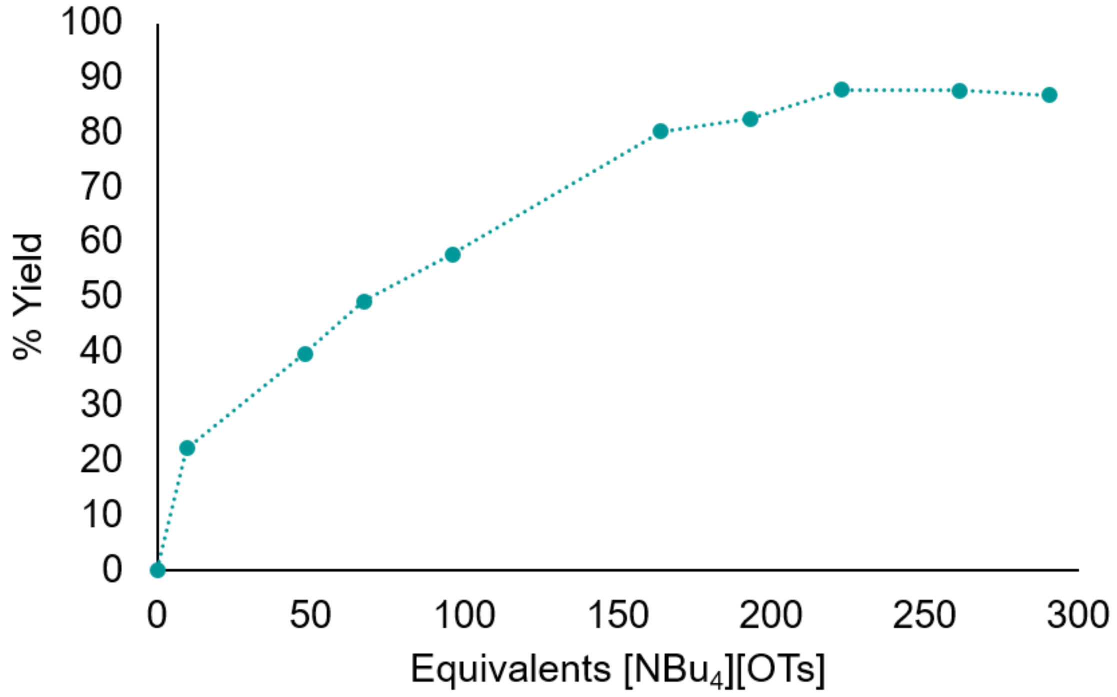

Figure S13: UV-vis spectrum of **$[NBu_4][OTs]$** (4 mM) in MeCN.

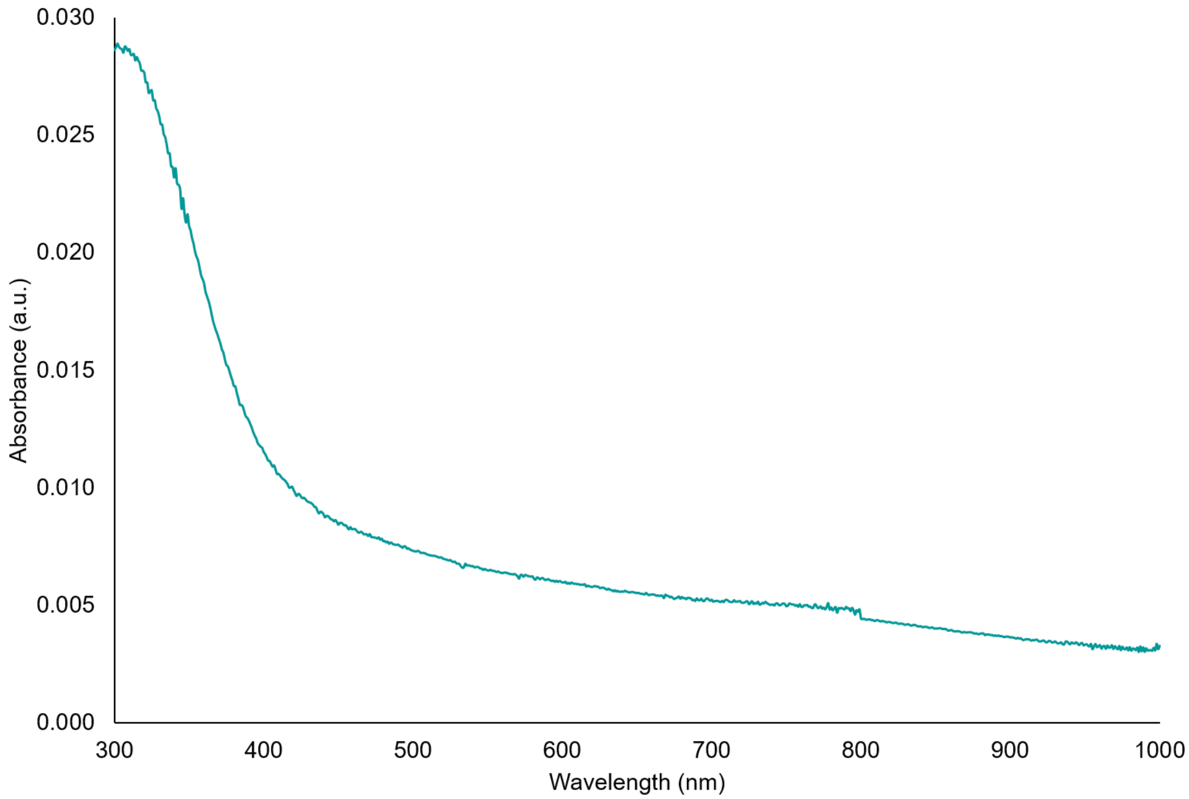

8. Substoichiometric Disproportionation Reactions

Given the low isolated yields of **3Phth** from disproportionation, we sought an alternative synthesis which could be used to make enough compound for characterization. Salt metathesis of 3Cl with potassium pthalimide did not give the desired product. We found that for any salt metathesis to occur, AgOTf was required as chloride scavenger. However, even under these conditions, we were only able to achieve mono-phthalimide substitution, giving **3-Phth-OTf** (Scheme S1C). This stable heteroleptic substitution complex suggested that it could be possible to form **3-Phth-OTf** as a disproportionation product by mixing **2OTf** with 1 equivalent of potassium phthalimide. In practice, this was not the case, the reaction of **2OTf** with 1 equiv. of potassium phthalimide resulted in the formation of **3Phth** and unreacted **2OTf** (Scheme S1B), suggesting the requirement of 2 equivalents of coordinating anion to engender full disproportionation. To confirm this, compound **2OTf** was titrated with [*n*Bu_4N][Cl], giving a linear titration curve for the formation of **1** which plateaued at 2 equivalents of chloride (see Figure S19).

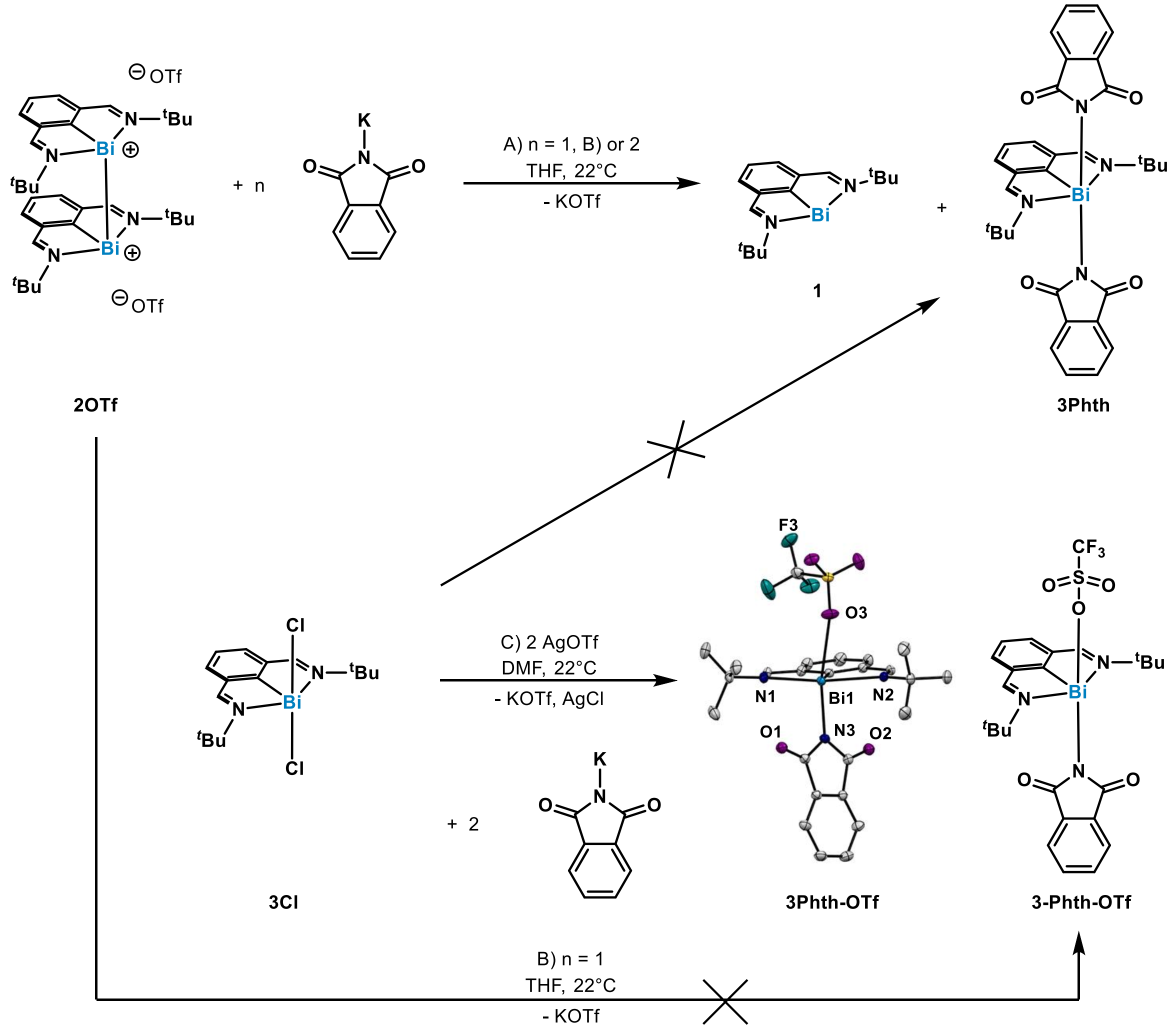

Scheme S1: Disproportionation of **2OTf** with A) 2 and B) 1 equiv. of potassium phthalimide and C) synthesis of **3Phth-OTf**. Inset solid state structure of **3Phth**, visualized with 50% probability ellipsoids. For the sake of clarity, H atoms have been omitted. Relevant XRD lengths and angles: **3Phth-OTf**: C(1)–Bi(1) 2.1940(18) Å, N(1)–Bi(1) 2.5194(16) Å, N(2)–Bi(1) 2.4778(16) Å,

Bi(1)–N(3) 2.2596(15) Å, Bi(1)–O(3) 2.6208(15) Å, C(1)–Bi(1)–N(1) 70.82(6)°, C(1)–Bi(1)–N(2) 71.73(6)°, O(3)–Bi(1)–N(3) 168.28(6)°.

1.0 equiv. [NBu_4][Cl]: A CH_3CN stock solution of [NBu_4][Cl] (0.0042 mmol, 0.083 M, 50 μL, 0.98 equiv.) was added to a yellow CD_3CN stock solution of **2OTf** (0.0043 mmol, 0.007 M, 0.6 mL, 1.0 equiv.) and a MeCN solution of 1,3,5-trimethoxybenzene (0.0042 mmol, 0.021 M, 200 μL, 0.98 equiv.). The solution quickly became brown. The solvent was removed from the reaction mixture and ESI-MS of the residue revealed a signal to corroborate the presence of **3Cl** in the positive ion mode: $[LBiCl]^+ = [C_{16}H_{23}N_2ClBi]^+$, found (calculated) m/z 487.13481 (487.13481).

0.5 equiv. [NBu_4][Cl]: A CH_3CN stock solution of [NBu_4][Cl] (0.0021 mmol, 0.083 M, 25 μL, 0.49 equiv.) was added to a yellow CD_3CN stock solution of **2OTf** (0.0043 mmol, 0.007 M, 0.6 mL, 1.0 equiv.) and a MeCN solution of 1,3,5-trimethoxybenzene (0.0042 mmol, 0.021 M, 200 μL, 0.98 equiv.). The solution quickly became green.

In both cases, at room temperature the ^{1}H NMR spectra of the reaction mixture revealed broad ligand backbone signals, so variable temperature NMR experiments were conducted. At -50 °C, a set of sharp signals were observed and can be assigned to **3Cl**, which was found in 41% and 26% yield in the reactions using 1.0 and 0.5 equiv. [NBu_4][Cl], respectively. Another set of broad signals were observed at chemicals shifts intermediate to where the aldimine (s), *meta* (d) and *para* (t) protons appear at chemical shifts that are intermediate to those for **1** and **2** (see Figure S14 and S17), indicating a possible equilibrium between the two species.

1.0 equiv. potassium phthalimide: THF (3 mL) was added to solid **2OTf** (26 mg, 0.022 mmol, 1 equiv.) and potassium phthalimide (5 mg, 0.02 mmol, 1 equiv.) and the mixture was stirred for 4 days to give a dark teal solution with yellow precipitate. The solution was filtered over Celite, then pentane (15 mL) was added and a yellow precipitate formed. The supernatant was filtered over Celite and cooled to -35°C for 18 hours to give colorless crystals. The supernatant was decanted and the crystals dried and redissolved in DMF-d_7 (0.6 mL). Compound **3Phth** was identified by ^{1}H NMR spectroscopy.

Figure S14: Stacked ^{1}H NMR spectra (300 MHz, MeCN-d$_3$) showing the aromatic region for **1**, **2OTf** and the reaction mixture of **2OTf** with one equivalent of [NBu$_4$][Cl].

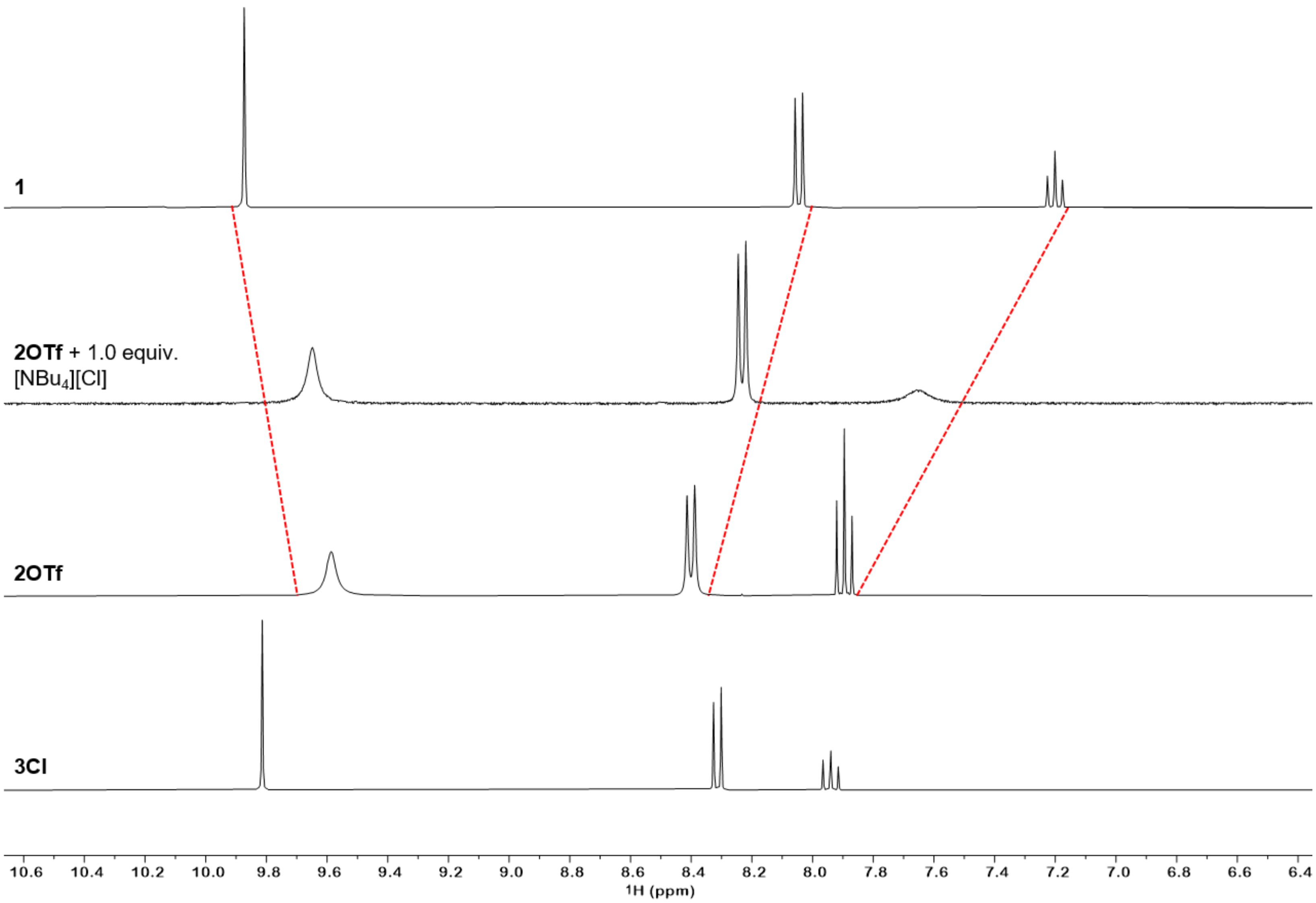

Figure S15: Stacked ^{1}H NMR spectra (500 MHz, $MeCN-d_3$) showing the aromatic region for an aliquot of the reaction mixture of the addition of 1.0 equivalents $[NBu_4][Cl]$ to **2OTf** between -50 and 20 °C.

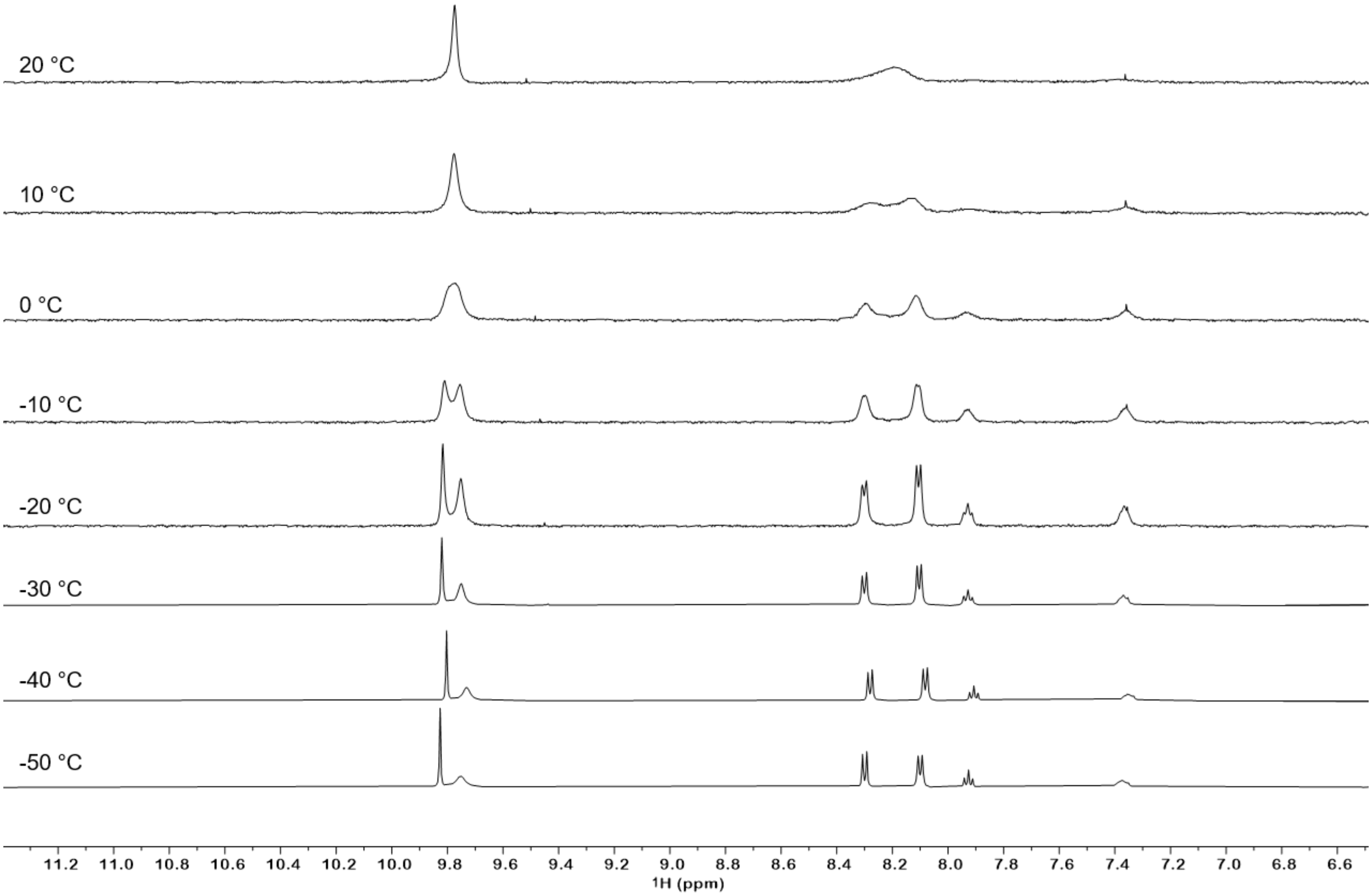

Figure S16: Stacked ^{1}H NMR spectra (500 MHz, MeCN-d_3) showing the aromatic region for an aliquot of the reaction mixture of the addition of 0.5 equivalents [NBu_4][Cl] to **2OTf** between -50 and 20 °C.

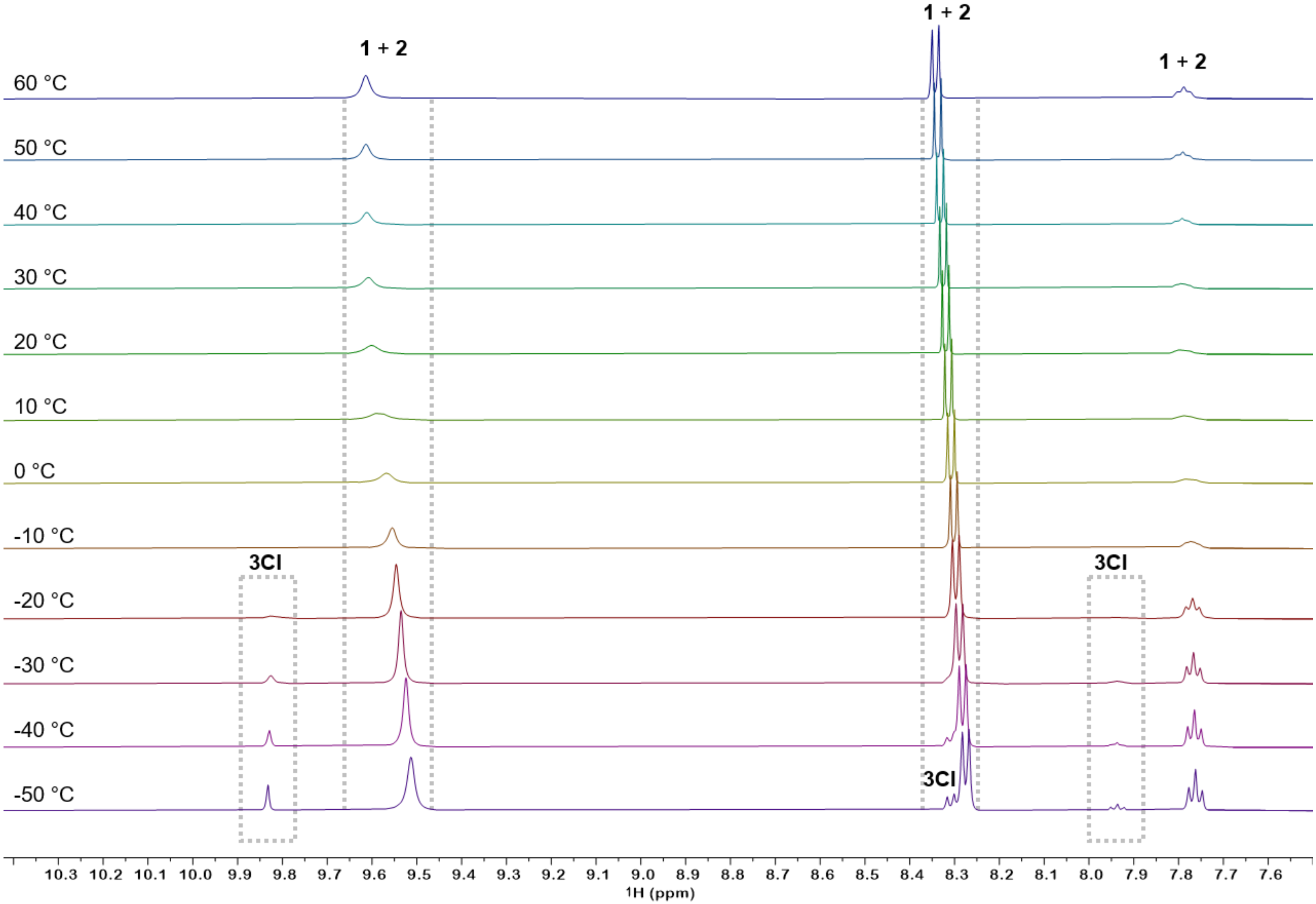

Figure S17: Stacked ^{1}H NMR spectra (MeCN-d3) showing the aromatic region for an aliquot of the reaction mixture in the addition of 0.5 and 1.0 equivalents [NBu4][Cl] to **2OTf** (500 MHz, -50°C) compared to **1**, **2OTf**, and **3Cl** (300 MHz, 25°C). In both reaction mixture spectra, the **3Cl** signals are resolved, while the signals for **1** and **2OTf** appear to coalesce.

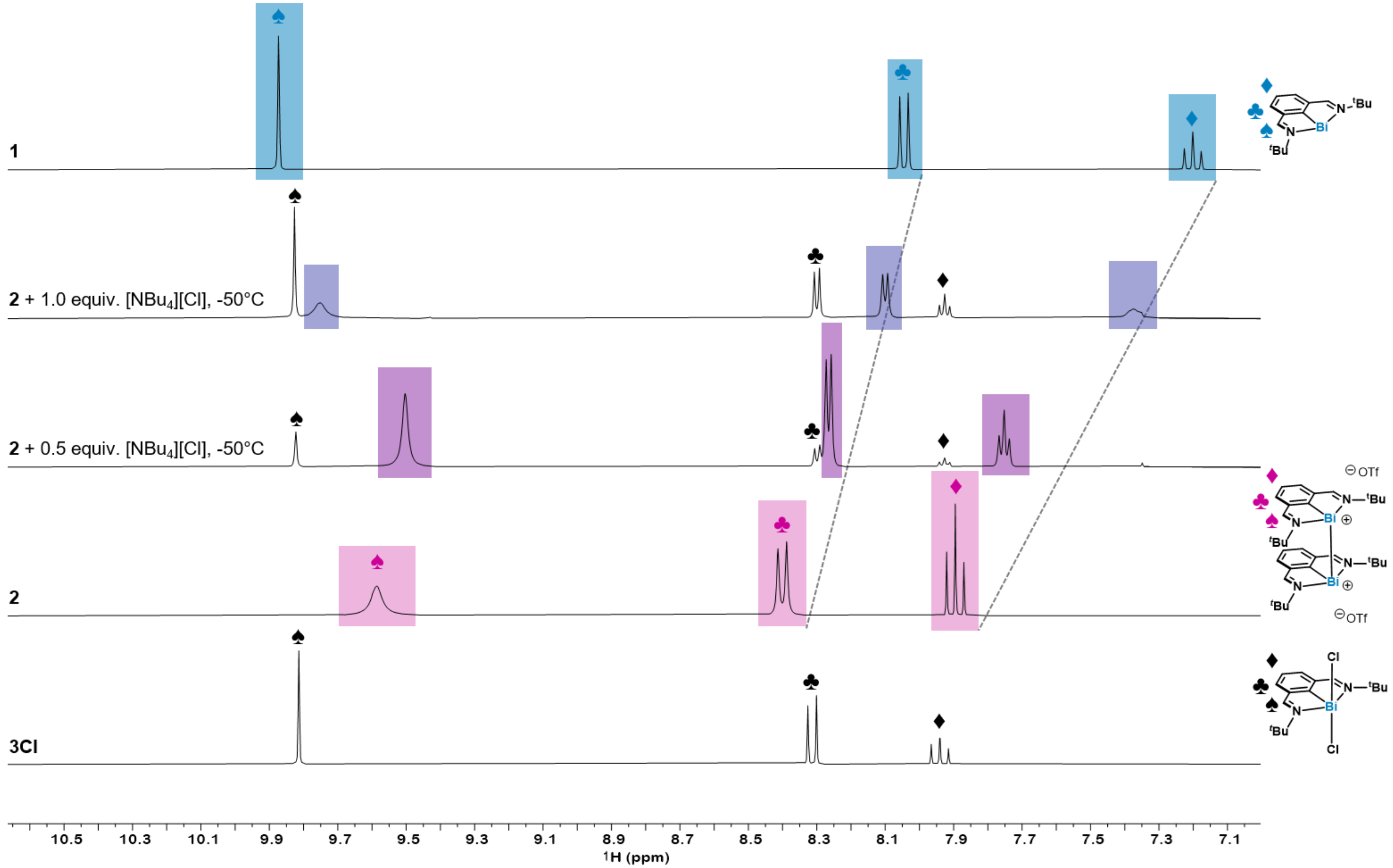

Figure S18: UV-vis spectra of **2OTf** (0.26 mM) combined with 0 to 2.2 equivalents (n) of $[NBu_4][Cl]$. Inset photo of colored stock solutions.

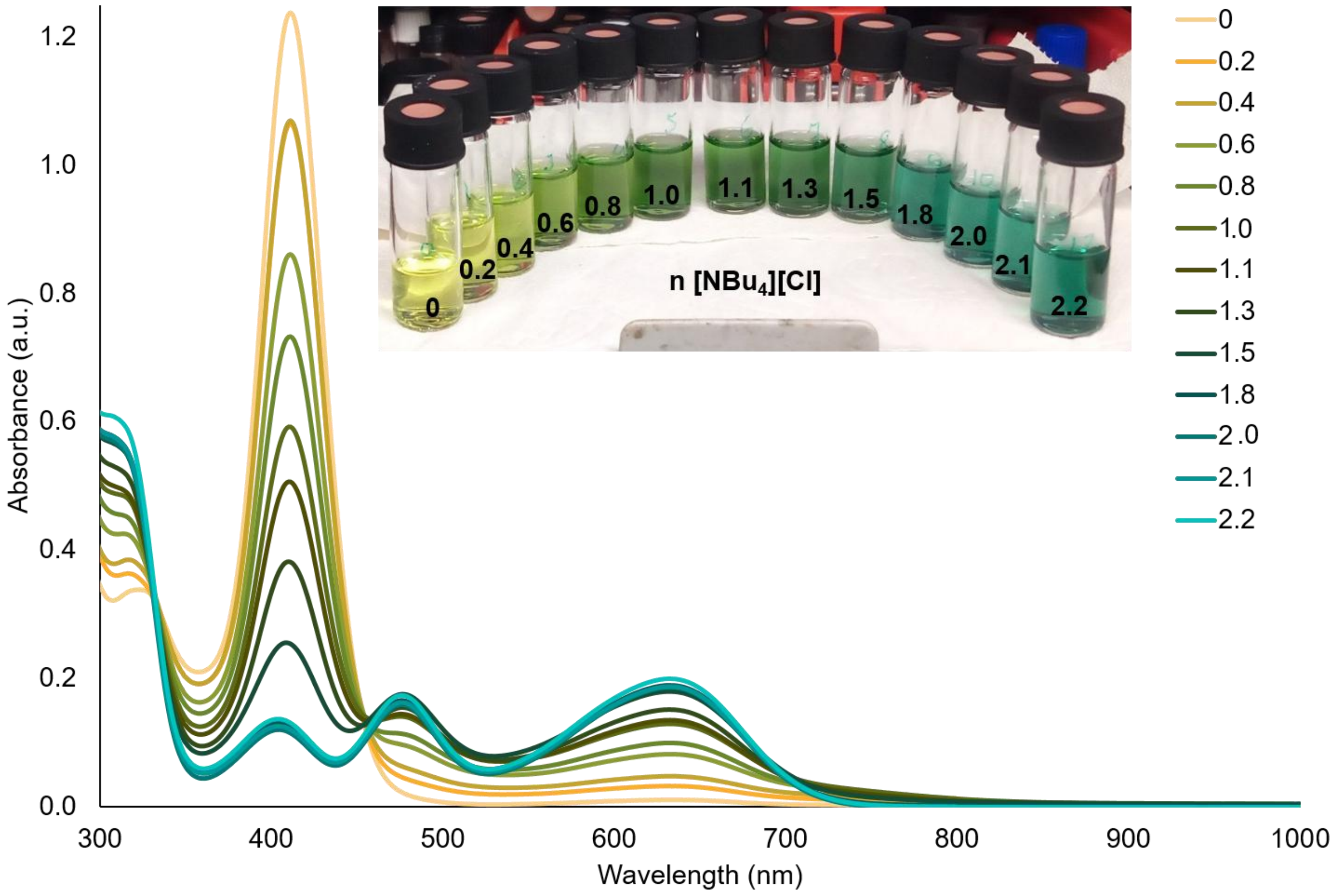

Figure S19: Titration curve for the disproportionation of **2OTf** using $[NBu_4][Cl]$.

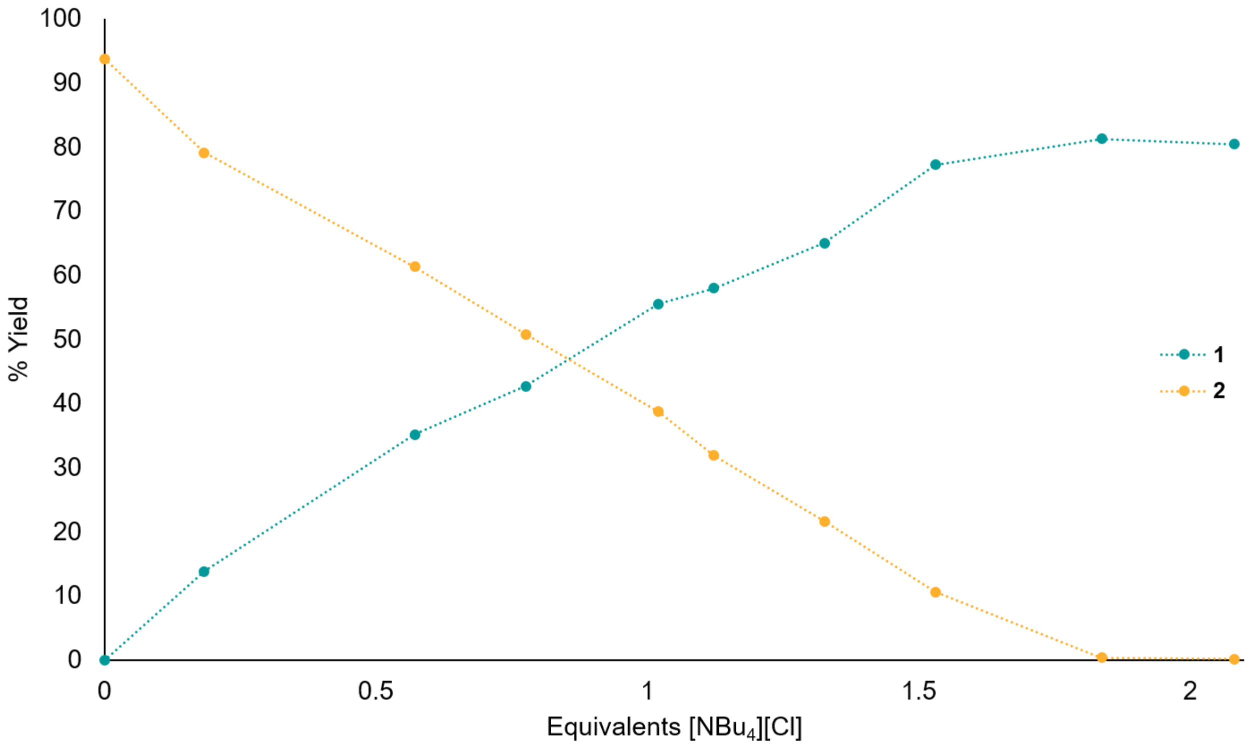

9. Solvent Dependence in 2OMs

To further investigate the effect of solvent, 2.0 equiv. of diethyl ether, THF, toluene, and DMF were added to MeCN-d_3 solutions of **2OMs** and no change was observed by NMR spectroscopy. Then each solvent was added in excess to give a 1:3 solvent:MeCN volume ratio. This resulted in a color change from yellow to green and broad ligand resonances which could not be resolved by VT-NMR spectroscopy. On further inspection, absorbances for **1** (λ_{max} ~ 633 nm) could be observed by UV-vis spectroscopy in all cases, pointing to disproportionation (see Figure S23). The same was conducted for DMSO and no color change was observed and no formation of **1** was observed spectroscopically. To corroborate the above results, solid **2OMs** was dissolved in THF-d_8 to give a dark teal solution, and **1** and **3OMs** could be observed by ^{1}H NMR spectroscopy (see Figure S21).

An MeCN-d_3 stock solution of **2OMs** (0.6 mL, 0.0054 mmol, 0.0090 M) was added to a Schlenk flask. The solvent was removed *in vacuo* to give a yellow-brown solid, then THF-d_8 (0.7 mL) was added and the mixture stirred for 18 hours to give a dark teal solution. A ^{1}H NMR spectrum of this mixture revealed the presence of disproportionation products, **1** and **3OMs** (Figure S21).

An MeCN-d_3 stock solution of **2OMs** (0.6 mL, 0.0054 mmol, 0.0090 M) was added to 5 different vials. The vials were charged with 2 equiv. (0.018 mmol) of either THF, diethyl ether, toluene, DMSO or DMF. In all cases, no color change was observed and no change was observed by NMR spectroscopy. Then, 200 μL of each respective solvent was added to each tube. The tube to which DMSO was added did not display any color, or spectroscopic change. In the case of THF, diethyl ether, toluene and DMF, a color change from yellow to green was observed, however the only change observed by NMR spectroscopy was a broadening, but not shifting of the signals in the ^{1}H NMR spectrum, which could not be resolved at low temperature (Figure S22). The samples were analyzed by UV-vis spectroscopy and revealed the presence of **1**. It has been previously reported that the absorbance for **1** (λ_{max} = 633 nm in MeCN) displays solvatochromism, with a bathochromic shift in non-polar solvents (λ_{max} = 682, 667 and 639 nm in pentane, diethyl ether and DMF, respectively).[10] The 3:1 acetonitile:solvent mixtures also exhibit solvatochromism, which is in line with what is reported for **1** (λ_{max} = 662, 670, 652 and 639 nm in 3:1 MeCN mixtures with toluene, diethyl ether, THF and DMF, respectively).

Figure S20: Left: Photo of NMR tubes containing **2OMs** in MeCN and in MeCN with 200 μL THF, diethyl ether and toluene. Right: Photo of toluene layered with a MeCN solution of **2OMs**, showing a dark teal color at the solvent interface, a qualitative indicator of disproportionation.

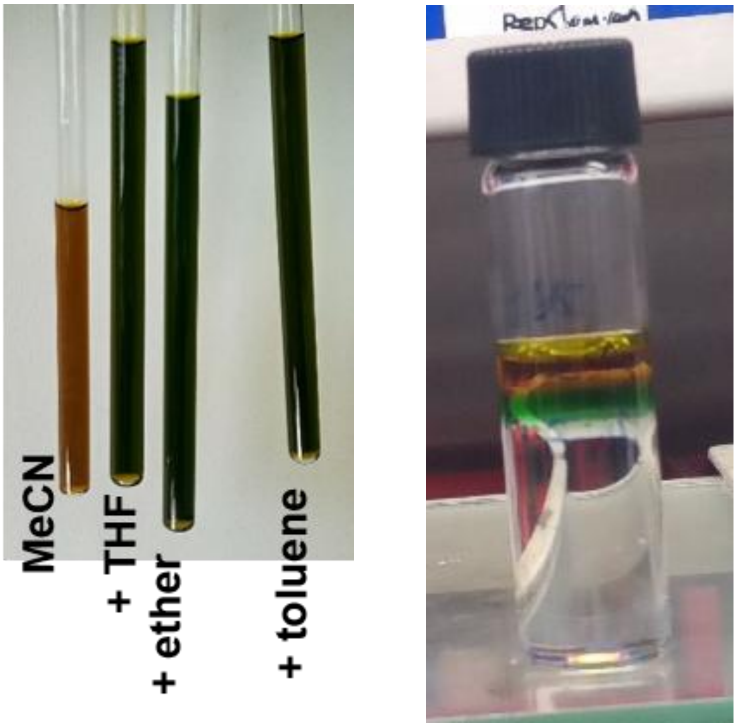

Figure S21: Stacked ^{1}H NMR spectra (300 MHz, 25°C) showing the aromatic region for **2OMs** (MeCN-d_3), and **2OMs** (THF-d_8), showing that the compound disproportionates to **1** and **3OMs** in THF.

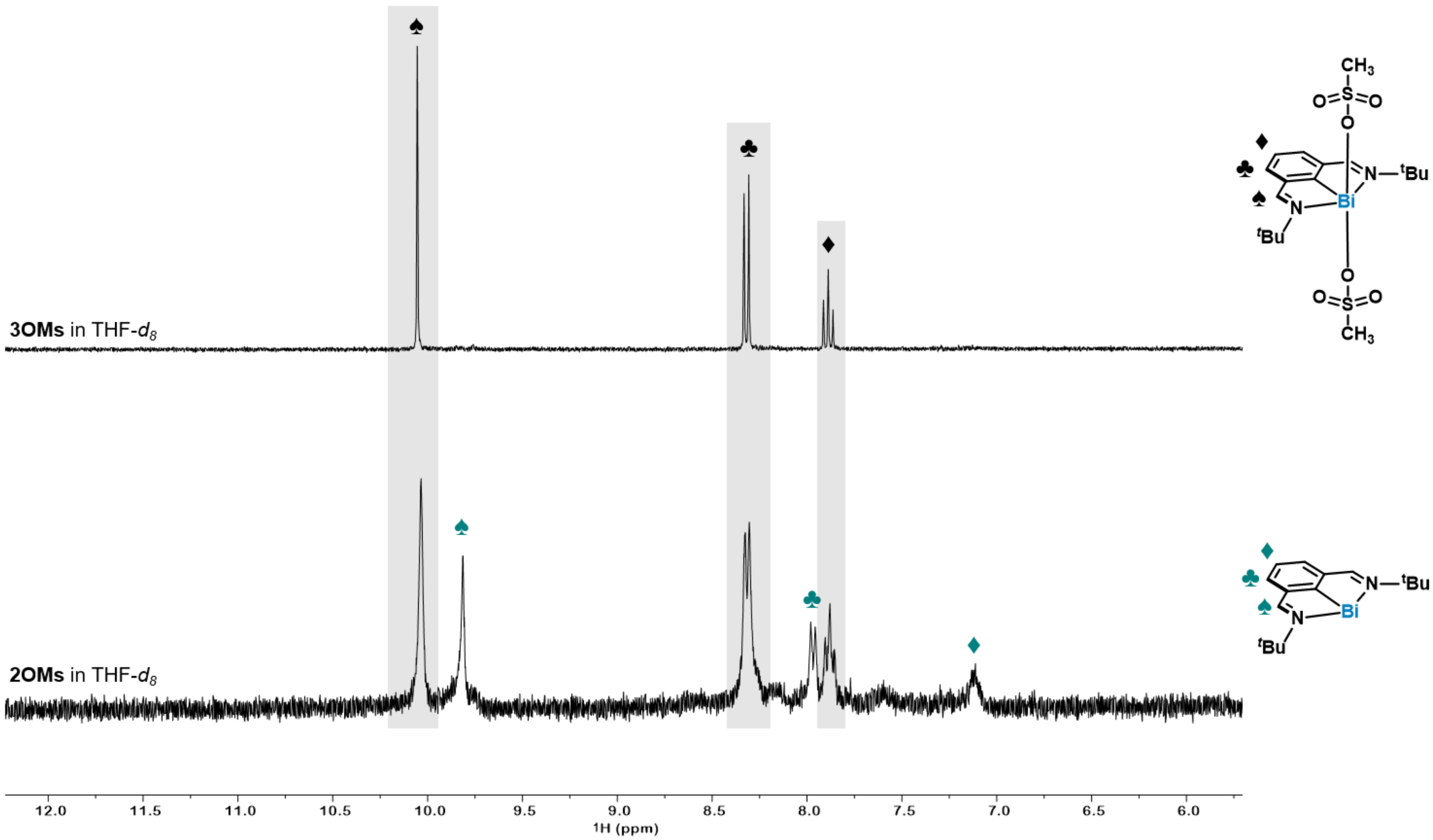

Figure S22: Stacked ^{1}H NMR spectra (MeCN-d_3) showing the aromatic region for **2OMs** (300 MHz, 25°C), and **2OMs** dissolved in MeCN-d_3 with added solvent (200 µL): THF (600 MHz, -40°C), THF (300 MHz, 25°C), diethyl ether (300 MHz, 25°C) and toluene (300 MHz, 25°C).

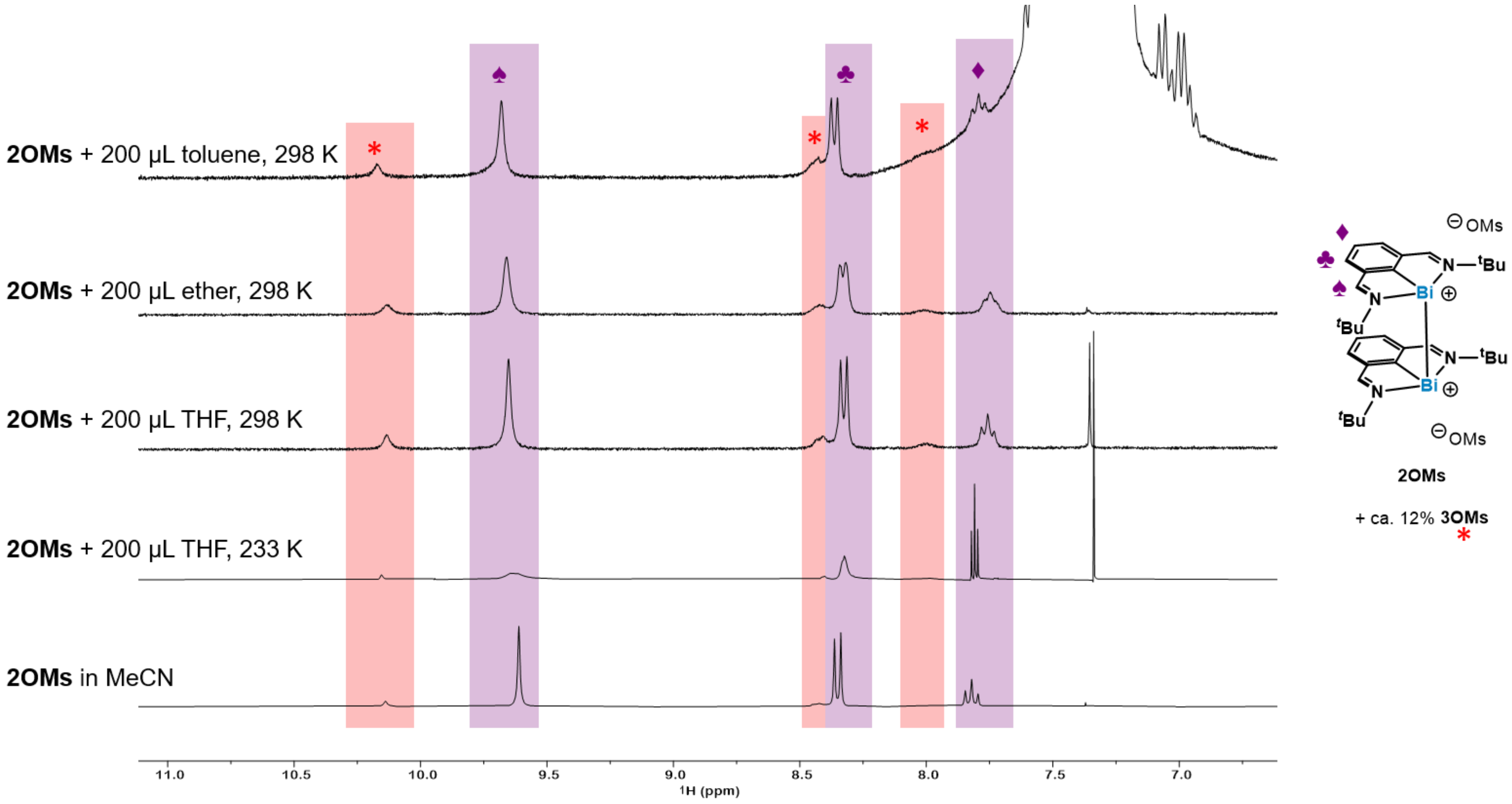

Figure S23: UV-vis spectra of **2OMs** in different solvent mixtures showing the formation of **1** with THF, diethyl ether, toluene and DMF.

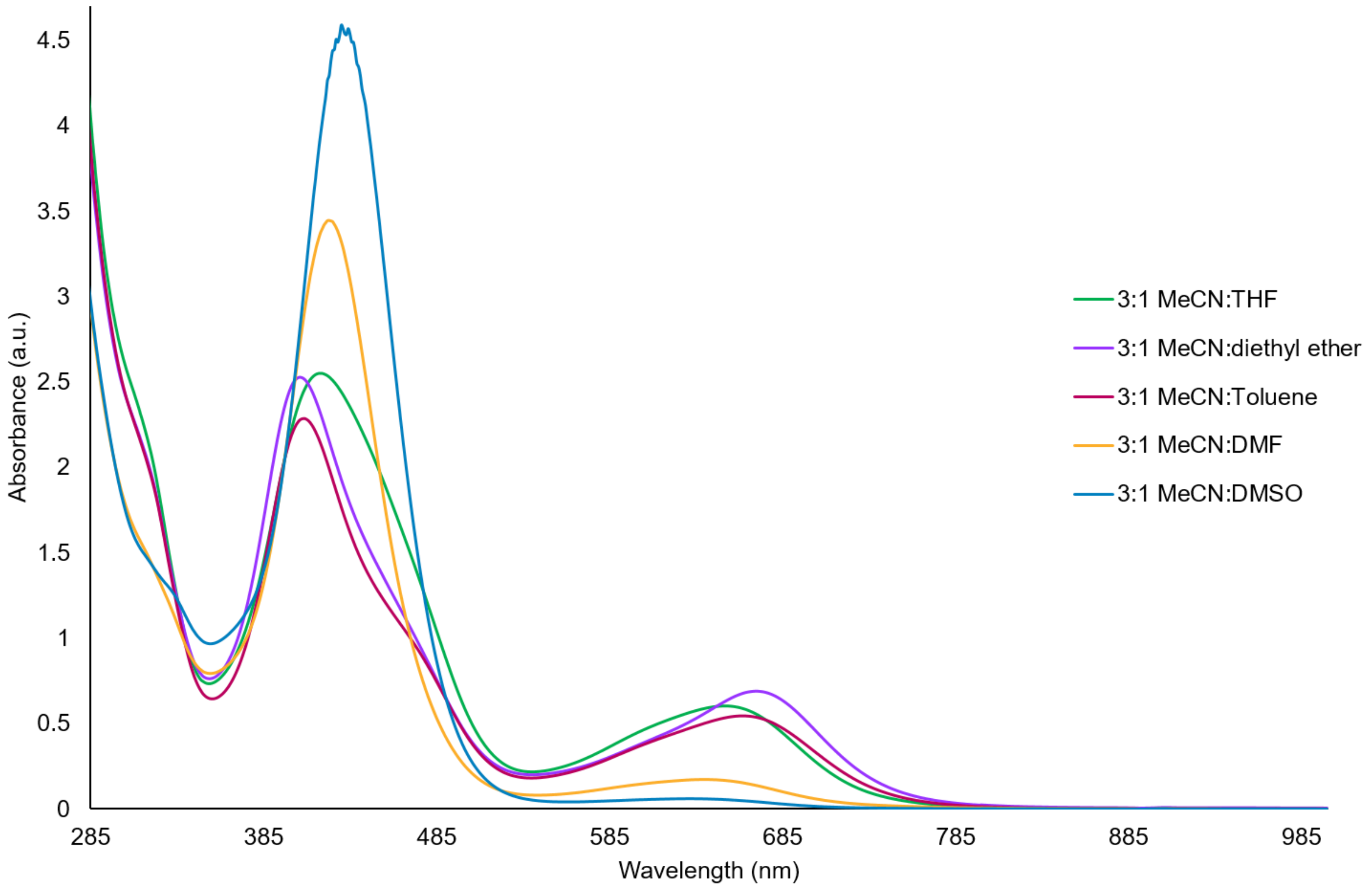

10. Gutmann-Beckett Method

The pincer ligand protons in **3OTf** and **3OMs** are deshielded relative to all other pincer Bi(III) complexes discussed in this work (see Table S1). This, combined with the non-coordinating nature of triflate and mesylate, made us suspect that **3OTf** and **3OMs** might be Lewis acidic. This was probed using the Gutmann-Beckett Method.[11,12] Compounds **3Cl** and **2OTf** were also analyzed for comparison.

The acceptor number (AN) was calculated by plugging the $^{31}P\{^{1}H\}$ NMR chemical shift into the below equation, as reported:[13]

$$AN = 2.21\ x\ (\delta_P - 41.0\ (ppm))$$

*Gutmann-Beckett Measurement for **3OTf**:* A CH_2Cl_2 stock solution of $OPEt_3$ (100 μL, 0.0067 mmol, 0.067 M, 1 equiv.) was added to solid **3OTf** (6 mg, 0.007 mmol, 1 equiv.), then CD_2Cl_2 (0.5 mL) was added and the $^{31}P\{^{1}H\}$ NMR spectrum was recorded (δ_P = 70.4 ppm, AN = 65).

*Gutmann-Beckett Measurement for **2OTf**:* A CH_2Cl_2 stock solution of $OPEt_3$ (186 μL, 0.012 mmol, 0.067 M, 0.9 equiv.) was added to solid **2OTf** (16 mg, 0.014 mmol, 1.0 equiv.), then CD_2Cl_2 (0.5 mL) was added and the $^{31}P\{^{1}H\}$ NMR spectrum was recorded (δ_P = 51.3 ppm, AN = 23).

*Gutmann-Beckett Measurement for **3OMs**:* CD_2Cl_2 (0.6 mL) was added to $OPEt_3$ (1 mg, 0.009 mmol, 1 equiv.) and solid **3OMs** (5 mg, 0.008 mmol, 1 equiv.), then the $^{31}P\{^{1}H\}$ NMR spectrum was recorded (δ_P = 50.5 ppm, AN = 21).

*Gutmann-Beckett Measurement for **3Cl**:* CD_2Cl_2 (0.6 mL) was added to $OPEt_3$ (4 mg, 0.03 mmol, 1 equiv.) and solid **3Cl** (16 mg, 0.30 mmol, 1 equiv.), then the $^{31}P\{^{1}H\}$ NMR spectrum was recorded (δ_P = 51.0 ppm, AN = 22).

*Gutmann-Beckett Measurement for **3Phth-OTf**:* A CH_2Cl_2 stock solution of $OPEt_3$ (300 μL, 0.015 mmol, 0.050 M, 0.94 equiv.) was added to solid **3Phth-OTf** (12 mg, 0.016 mmol, 1.0 equiv.), then CD_2Cl_2 (0.5 mL) was added and the $^{31}P\{^{1}H\}$ NMR spectrum was recorded (δ_P = 52.8 ppm, AN = 26).

Figure S24: $^{31}P\{^{1}H\}$ NMR spectrum of $OPEt_3$ in the presence of one molar equivalent of **3OTf**.

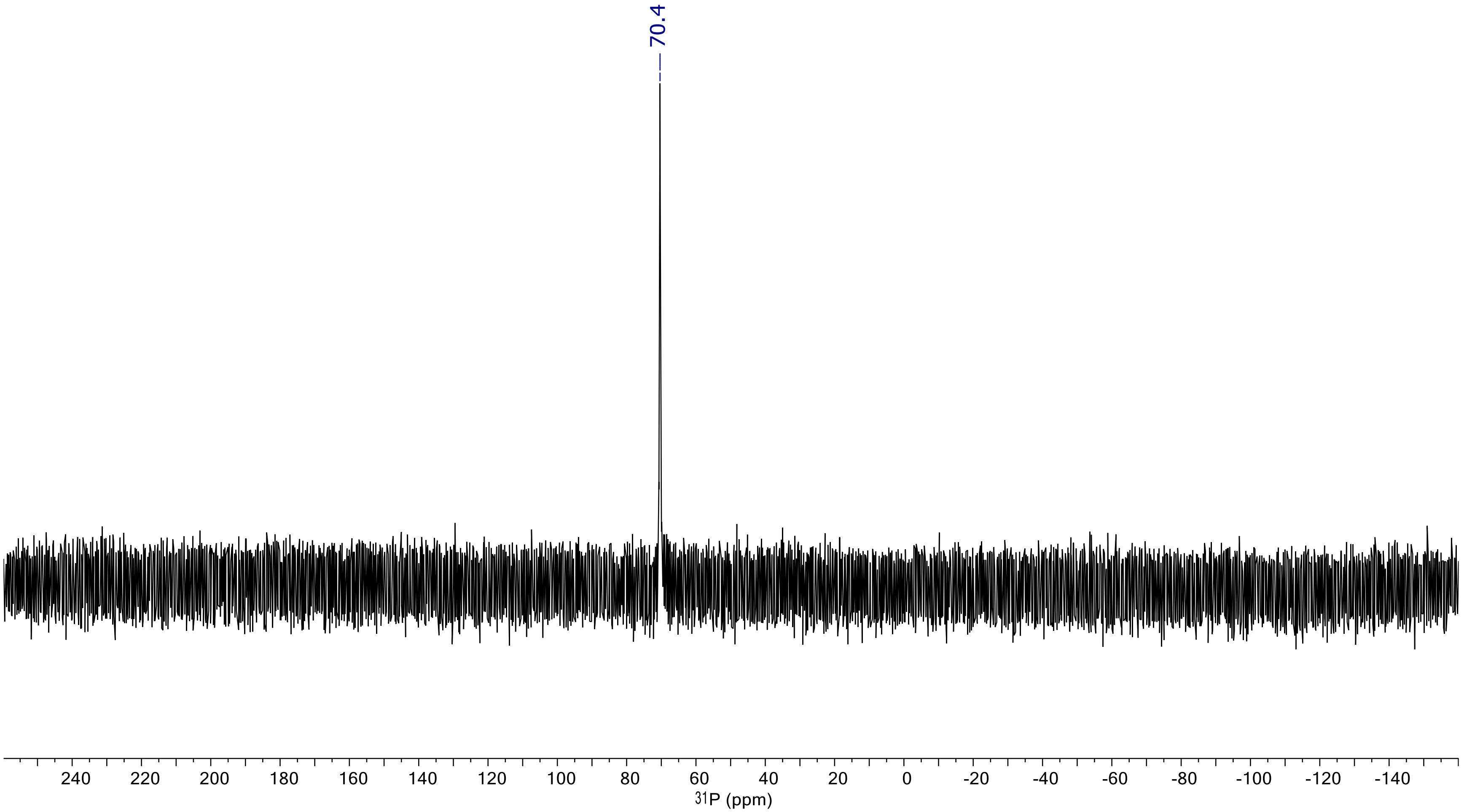

Figure S25: $^{31}P\{^1H\}$ NMR spectrum of $OPEt_3$ in the presence of one molar equivalent of **2OTf**.

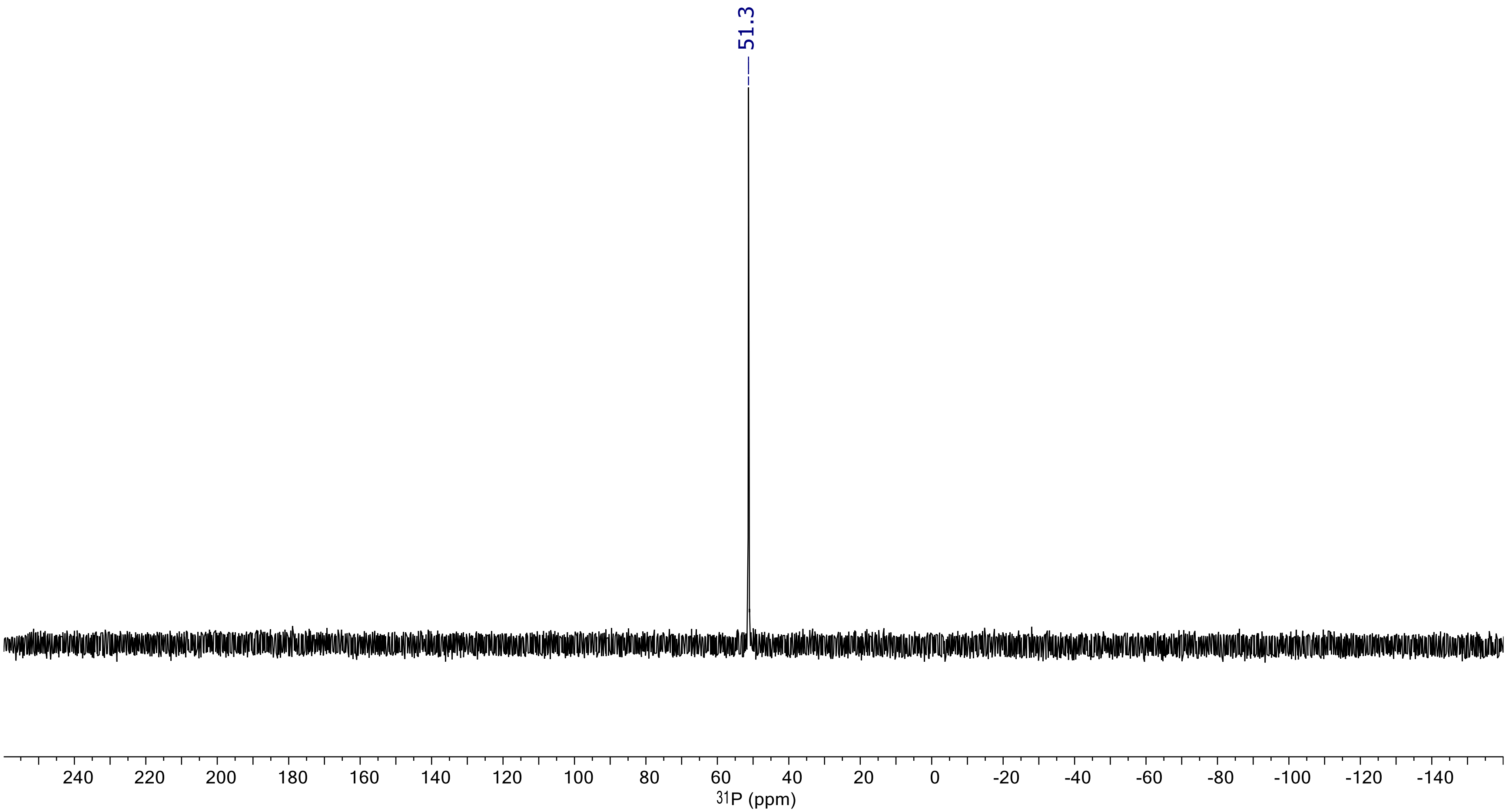

Figure S26: $^{31}P\{^1H\}$ NMR spectrum of $OPEt_3$ in the presence of one molar equivalent of **3OMs**.

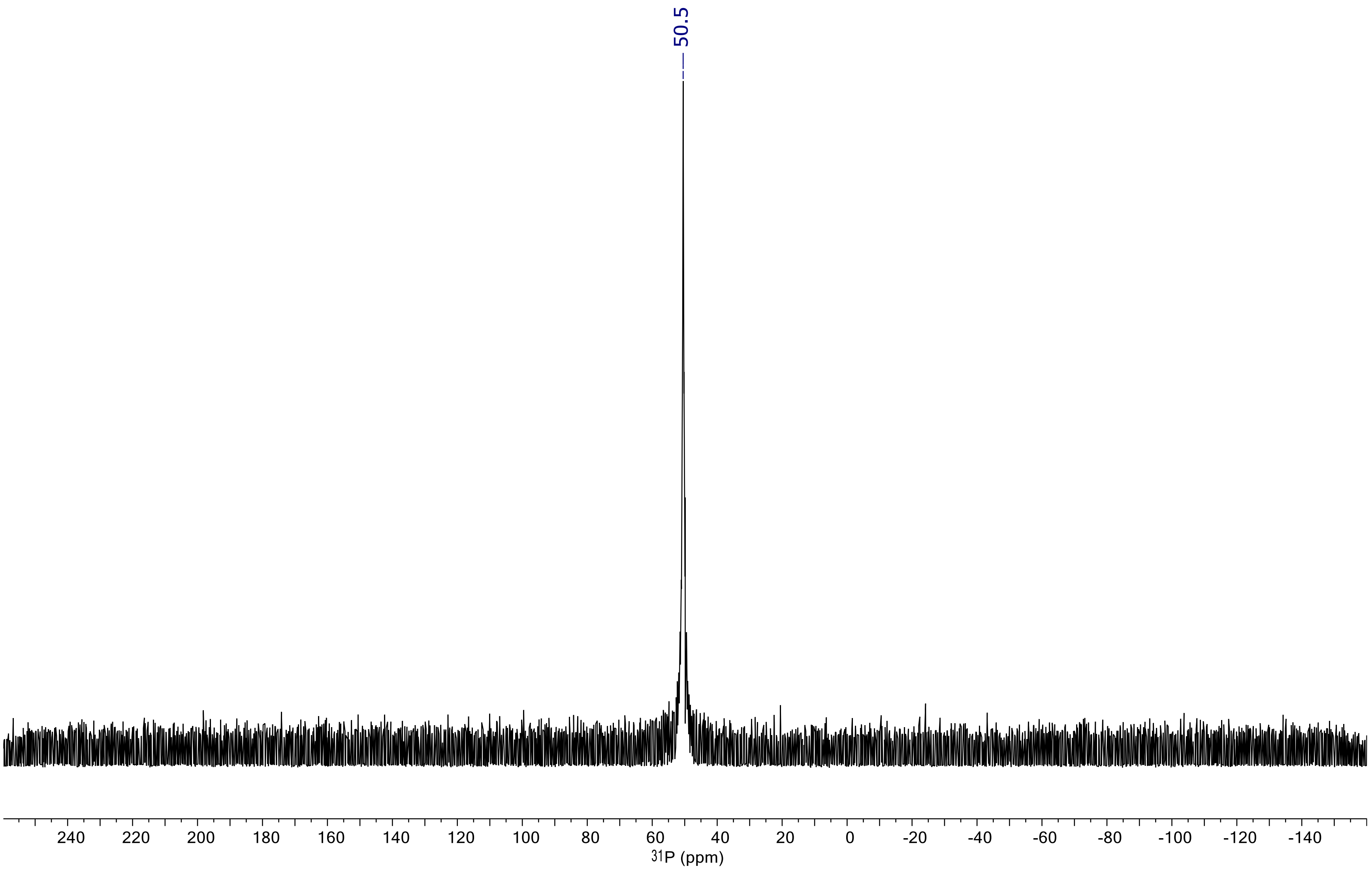

Figure S27: $^{31}P\{^1H\}$ NMR spectrum of $OPEt_3$ in the presence of one molar equivalent of **3Cl**.

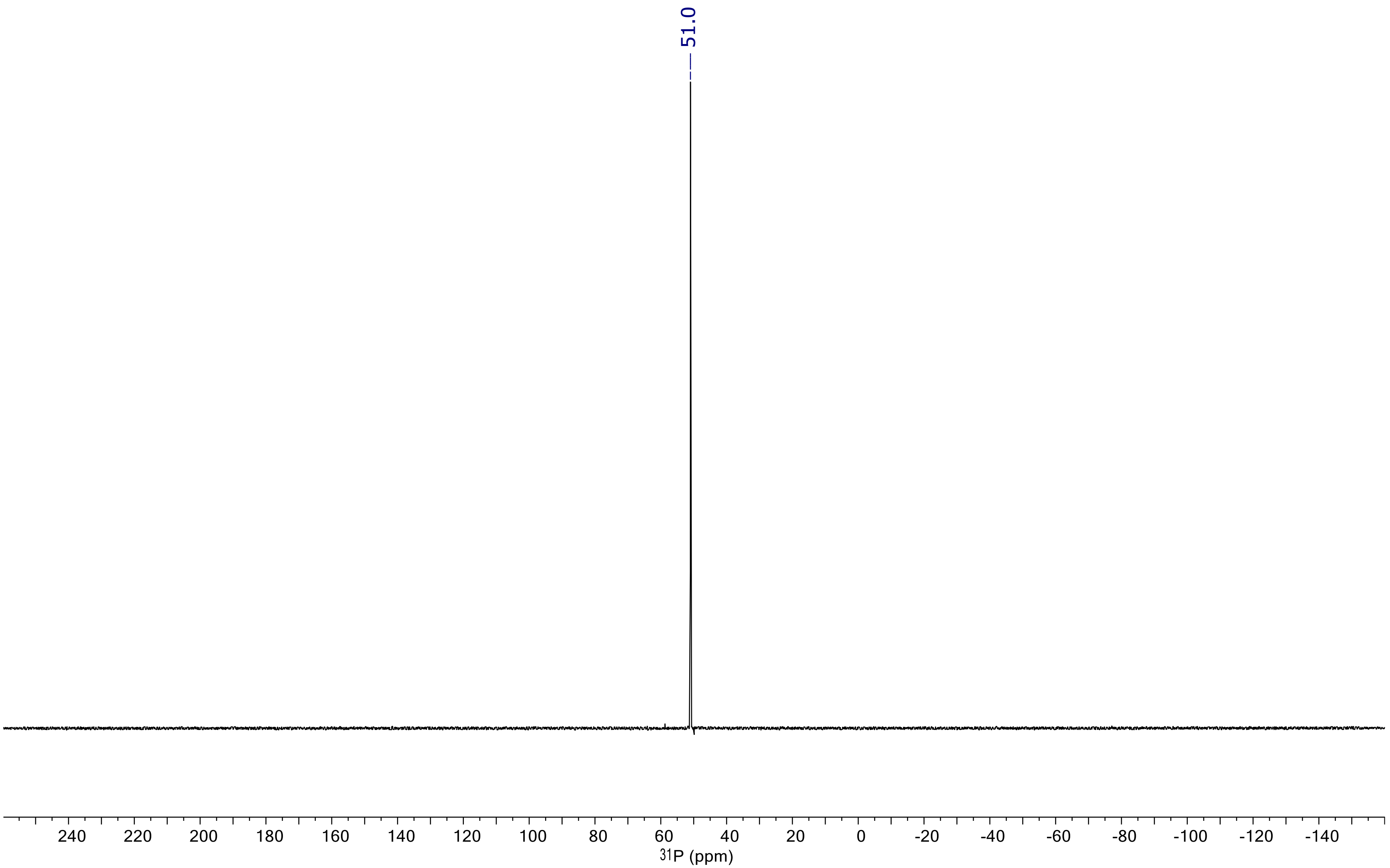

Figure S28: $^{31}P\{^1H\}$ NMR spectrum of $OPEt_3$ in the presence of one molar equivalent of **3Phth-OTf**.

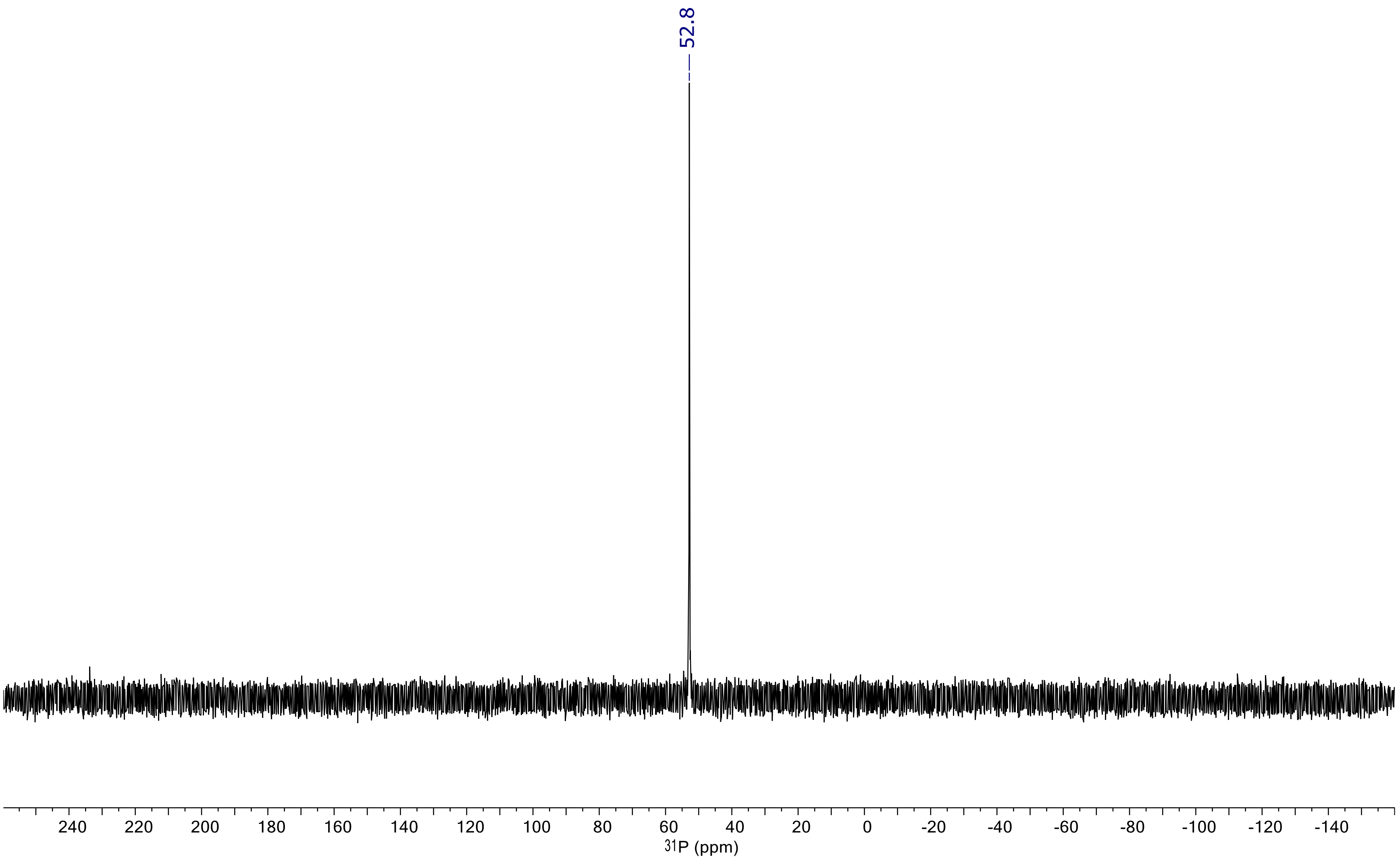

11. Cyclic Voltammetry

General considerations: Electrochemical measurements were performed in an argon-filled glovebox with a Gamry Interface 1010E Potentiostat connected to a 3-electrode cell consisting of a 1.6 mm Ø glassy carbon working electrode, a platinum wire counter electrode, and a bare silver wire as a pseudoreference electrode at ambient temperature in an argon-filled glovebox equipped with electrochemical outlets. A 20 mL scintillation vial used as the cell was fitted with a Teflon cap with openings for the three electrodes. Sublimed ferrocene was added as the internal reference, and all potentials are referenced to the well-defined, reversible $Fc^{0/+}$ couple. Ohmic drop was minimized by minimizing the distance between working and reference electrodes, and by employing a preset current-interrupt iR correction on the Gamry software. Glassy carbon electrodes were polished with an alumina suspension (0.05 μm) in deionized water on a pre-wetted polishing pad using figure-eight motions, rinsed with deionized water, rinsed with ethanol, and then sonicated in ethanol for 20 seconds to remove additional polishing powder, dabbing (not rubbing) any excess solvent from the electrode surface with lint-free paper towels. Silver pseudoreference electrodes were lightly polished with fine grit sandpaper, washed with ethanol, dabbed with lint-free paper towels, and sonicated in ethanol for 30 seconds to remove any particulate. Each voltammogram reported herein was recorded with freshly polished electrodes. Before collecting data in the presence of the analyte of interest, conditioning cycles were performed by cycling the potential between 1.8 V and –2.0 V four times at 100 mV/s in the supporting electrolyte solution. In all cases, the third and fourth cycles were superimposable, indicating sufficient conditioning of the electrodes.

Figure S29: Cyclic voltammograms at different scan rates (10 – 400 mV/s) of a 1 mM solution of **1** in a 100 mM solution of [^{n}Bu$_4$N][PF$_6$] in anhydrous degassed MeCN. Black arrow indicates the direction of the potential sweep. Dashed gray line represents the half-wave potential, $E_{1/2} = -0.884$ V vs Fc$^{+/0}$ (averaged across all scan rates).

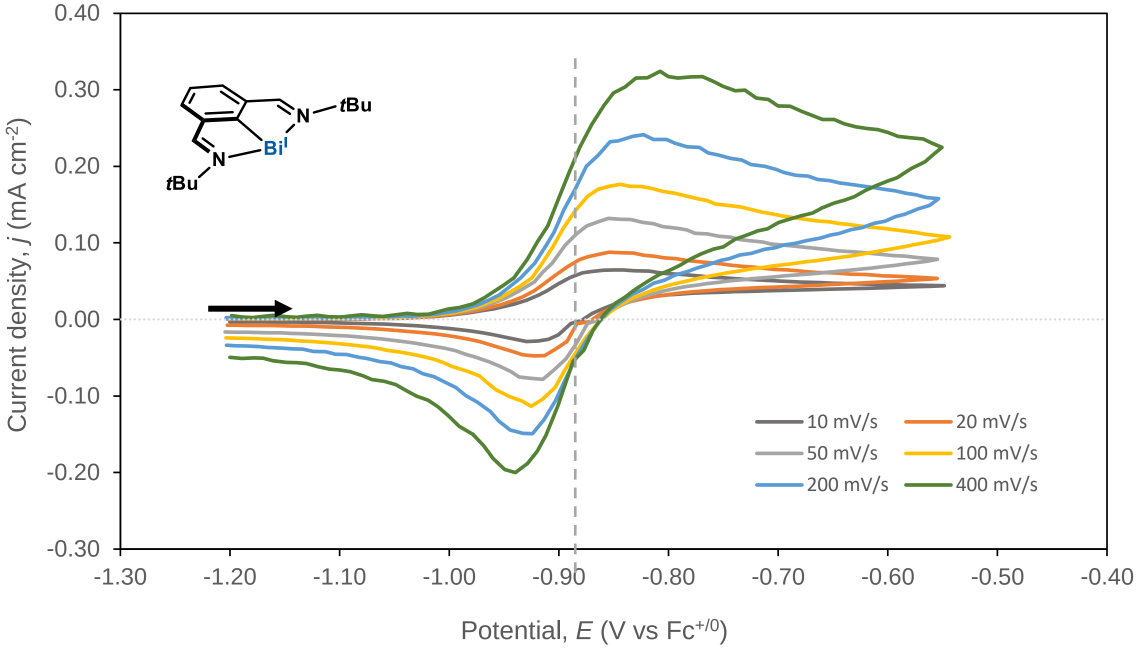

Figure S30: Cyclic voltammograms at different scan rates (20 – 800 mV/s) of a 1 mM solution of **1** in a 100 mM solution of [^{n}Bu$_4$N][OTf] in anhydrous degassed MeCN. Black arrow indicates the direction of the potential sweep. Dashed gray line represents the half-wave potential, $E_{1/2} = -0.830$ V vs Fc$^{+/0}$ (averaged across all scan rates).

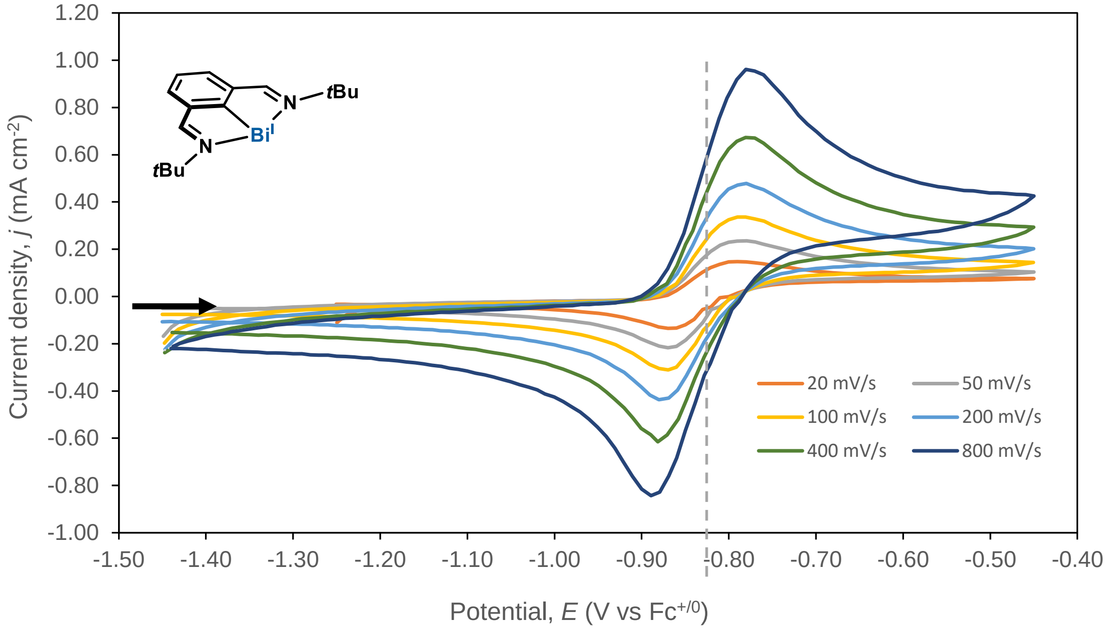

Figure S31: Randles-Sevcik plot showing a linear relationship between the peak current density and the square root of the scan rate. The data indicate that redox events in Figure S25 are occurring between the electrode and a freely-diffusing species in solution.

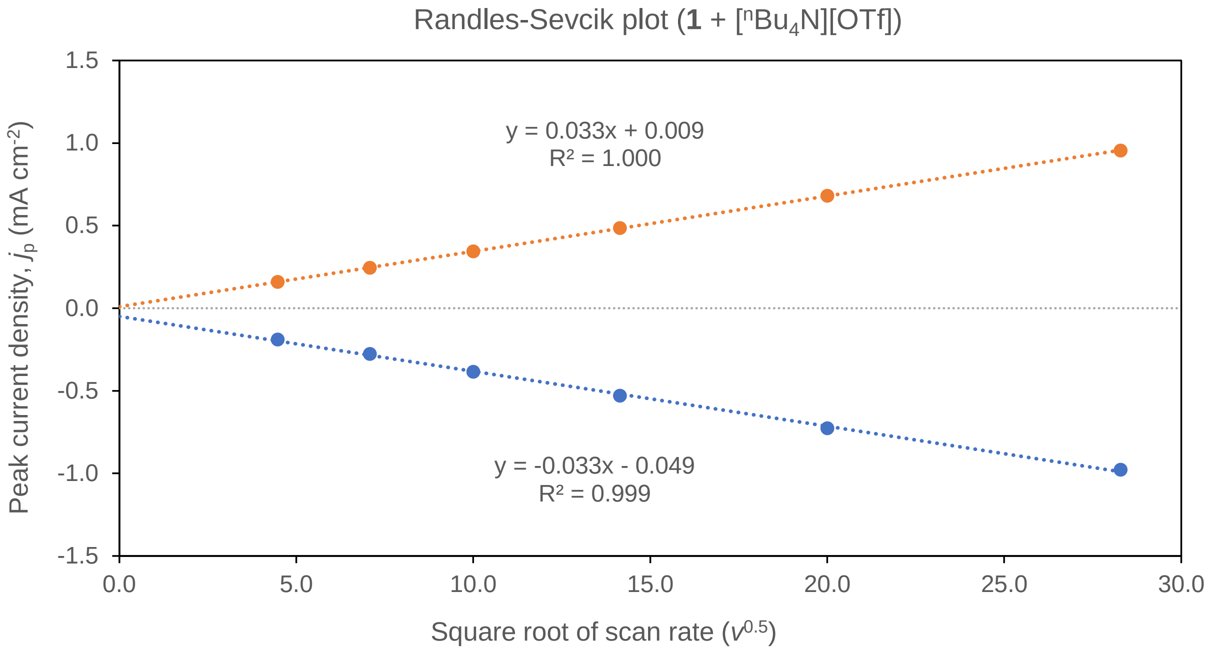

Figure S32: Cyclic voltammograms of a 1 mM solution of **1** (blue trace), **3Cl** (orange trace), and **1** + 10 equivalents of [^{n}Bu$_4$N][Cl] (grey trace) in a 100 mM solution of [^{n}Bu$_4$N][PF$_6$] in anhydrous degassed MeCN. Blue, grey, and orange arrows indicate the direction of the potential sweep for the blue, grey, and orange traces, respectively.

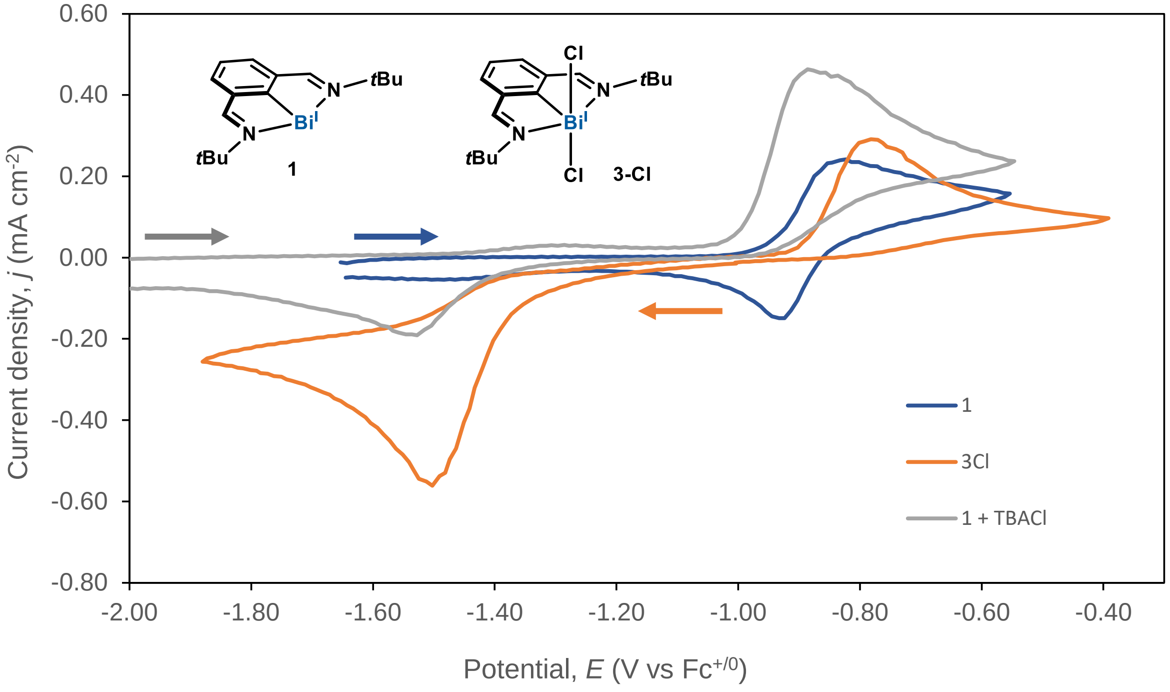

Figure S33: Cyclic voltammograms at different scan rates (20 – 800 mV/s) of a 1 mM solution of **1** in a 100 mM solution of [$^{n}Bu_4N$][Br] in anhydrous degassed MeCN. Black arrow indicates the direction of the potential sweep. Anodic and cathodic peak potentials located at *ca.* −0.872 V and −1.428 V vs $Fc^{+/0}$ (averaged across all scan rates).

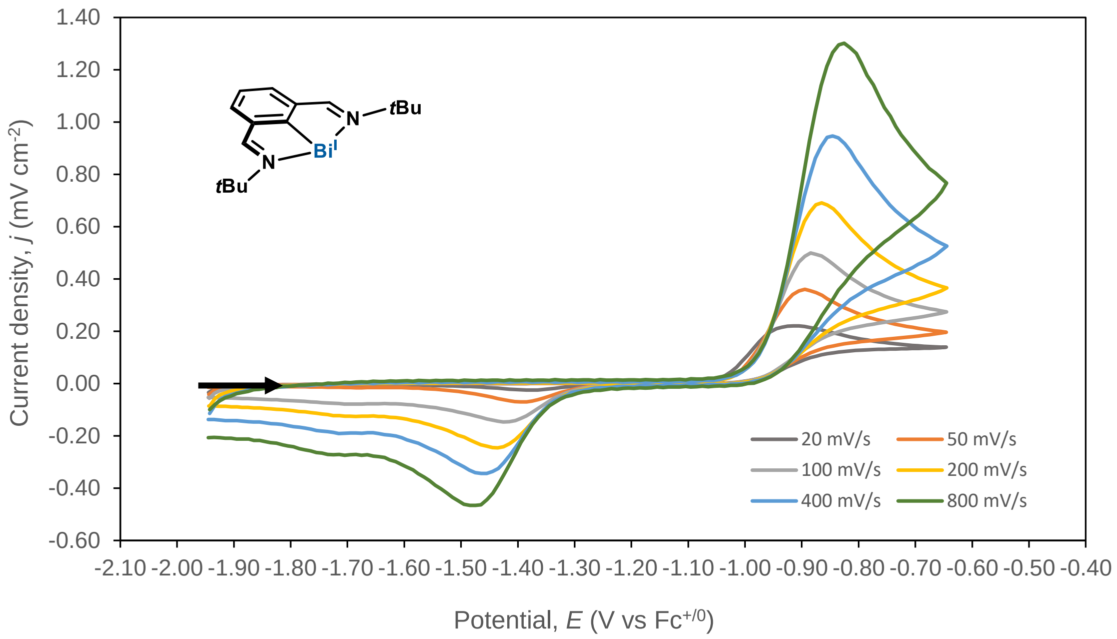

Figure S34: Cyclic voltammograms at different scan rates (20 – 3200 mV/s) of a 1 mM solution of **1** in a 100 mM solution of [$^{n}Bu_4N$][I] in anhydrous degassed MeCN. Black arrow indicates the direction of the potential sweep. Anodic and cathodic peak potentials located at *ca.* −0.587 V and −1.076 V vs $Fc^{+/0}$ (averaged across all scan rates).

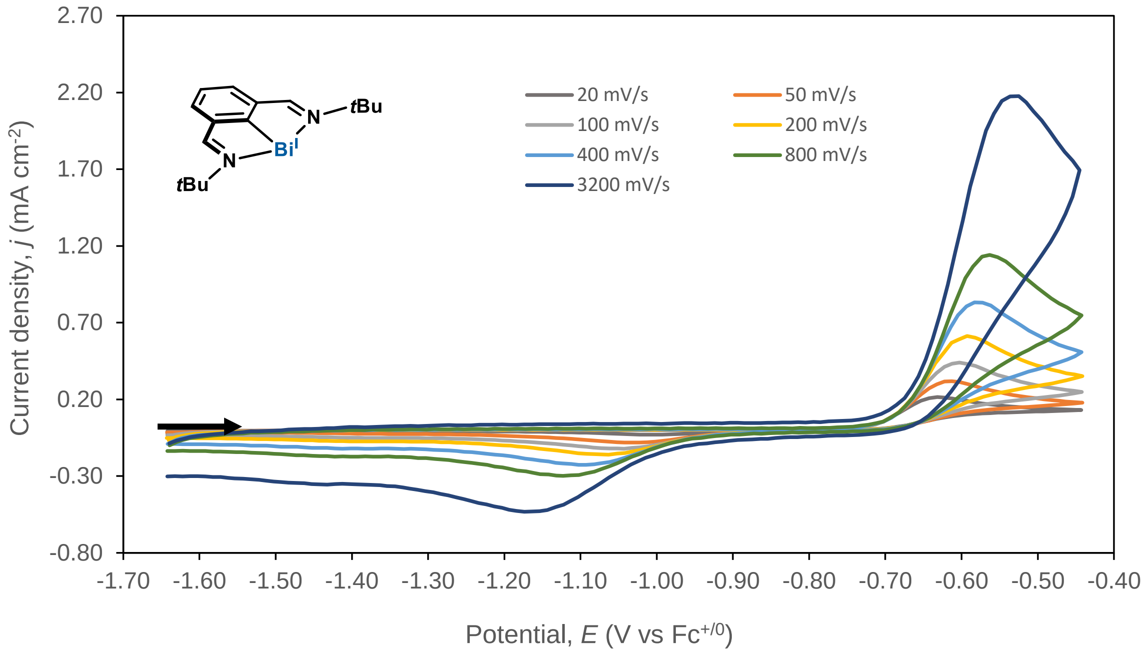

Figure S35: Cyclic voltammograms at different scan rates (20 – 800 mV/s) of a 1 mM solution of **1** in a 100 mM solution of [$^{n}Bu_4N$][BF_4] in anhydrous degassed MeCN. Black arrow indicates the direction of the potential sweep. Anodic and cathodic peak potentials located at *ca.* −0.857 V and −1.515 V vs $Fc^{+/0}$ (averaged across all scan rates).

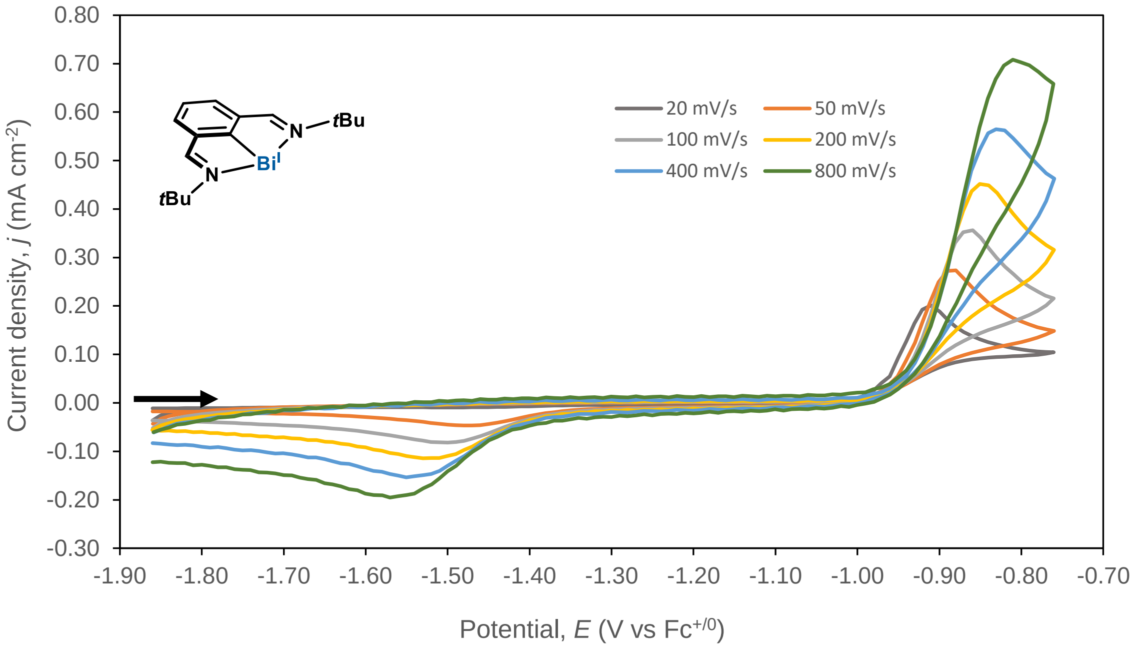

Figure S36: Cyclic voltammograms at different scan rates (20 – 400 mV/s) of a 1 mM solution of **1** in a 100 mM solution of [$^{n}Bu_4N$][OTs] in anhydrous degassed MeCN. Black arrow indicates the direction of the potential sweep. Anodic and cathodic peak potentials located at *ca.* −0.857 V and −1.515 V vs $Fc^{+/0}$ (averaged across all scan rates).

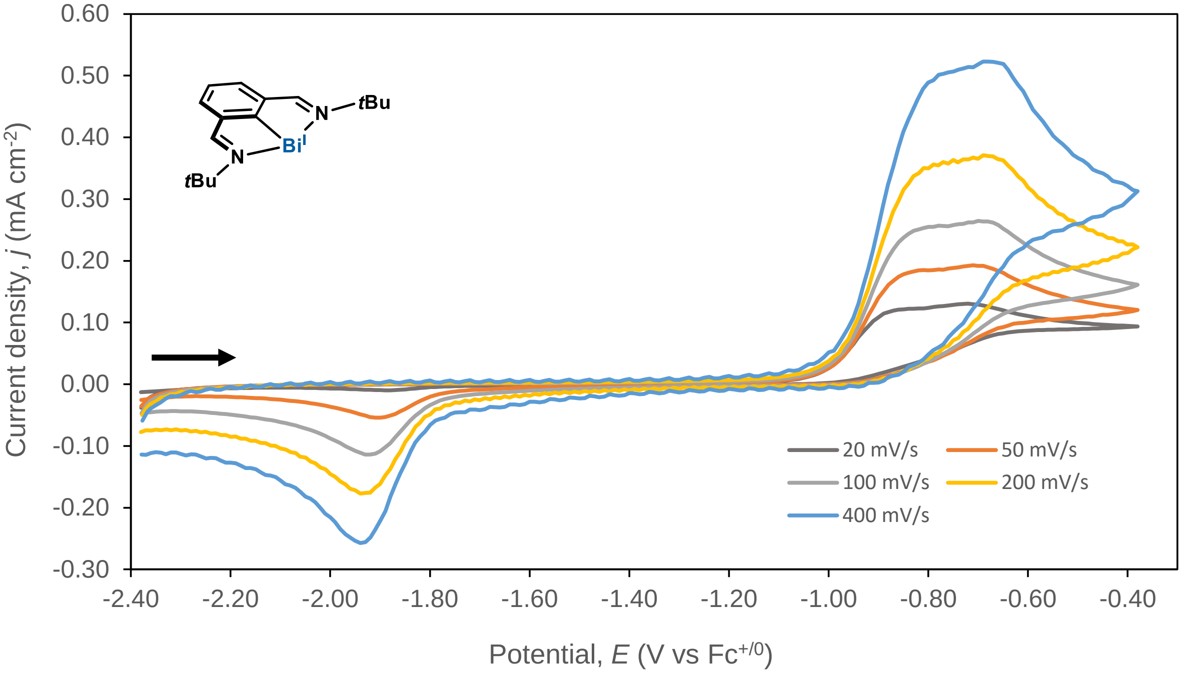

Figure S37: Cyclic voltammograms at different scan rates (10 – 800 mV/s) of a 1 mM solution of **4** in a 100 mM solution of [^{n}Bu$_4$N][PF$_6$] in anhydrous degassed MeCN. Black arrow indicates the direction of the potential sweep. Dashed gray line represents the half-wave potential, $E_{1/2} = -0.514$ V vs Fc$^{+/0}$ (averaged across all scan rates).

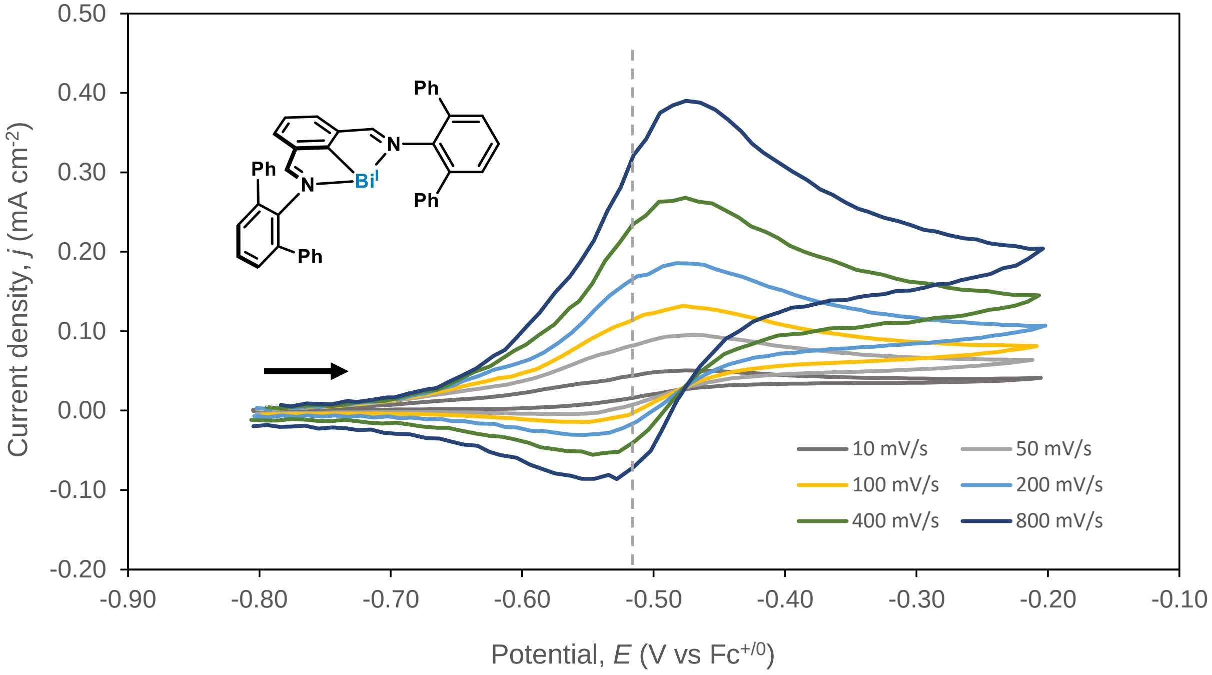

Figure S38: Cyclic voltammograms of a 1 mM solution of **4** in a 100 mM solution of [^{n}Bu$_4$N][PF$_6$] in anhydrous degassed MeCN. The traces shown are before (blue) and after (orange) the addition of 40 equivalents of [^{n}Bu$_4$N][Cl]. The loss of reversibility upon addition of the chloride salt indicates disproportionation or subsequent oxidation of the nascent Bi(II) formed during the anodic scan. Black arrow indicates the direction of the potential sweep.

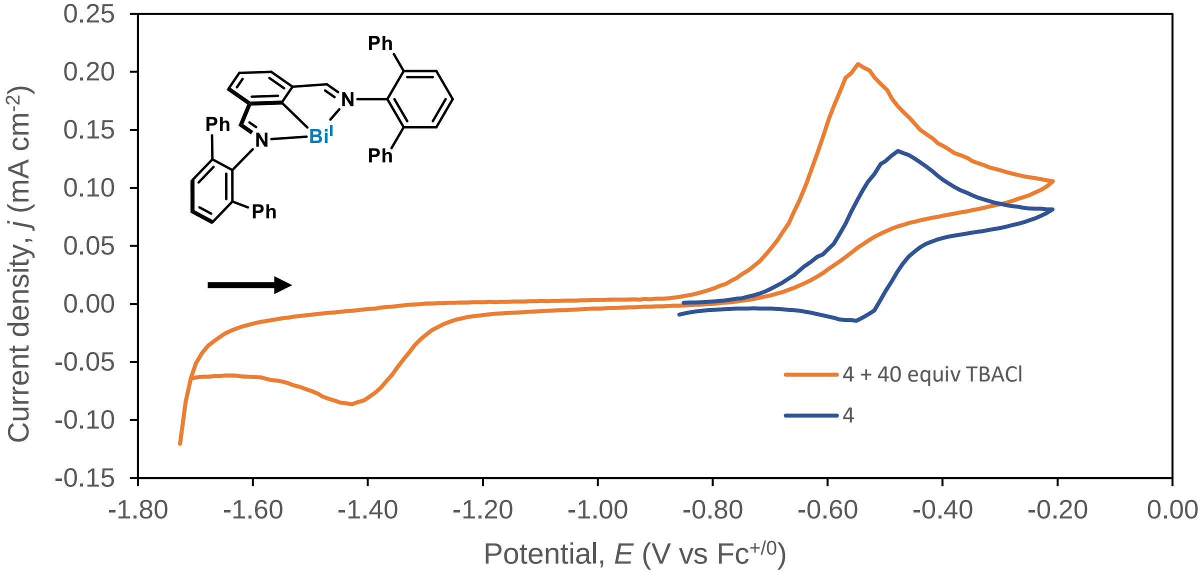

12. α Calculations

The coordinating ability index, α^{TM} used in Figure 3 of the main text are mainly taken from the literature.[14,15] The only α^{TM} value missing for our work was phthalimide. We also calculated the coordinating ability index for tetrafluoroborate specifically to bismuth (α^{Bi}). The search parameters are outlined below.

11.1 Calculation for phthalimide α^{TM}

The following structures were searched using ConQuest 2021.2.0 from the Cambridge Structural Database (version CSD 5.42) with the following constraints to obtain the c and u values for phthalimide.

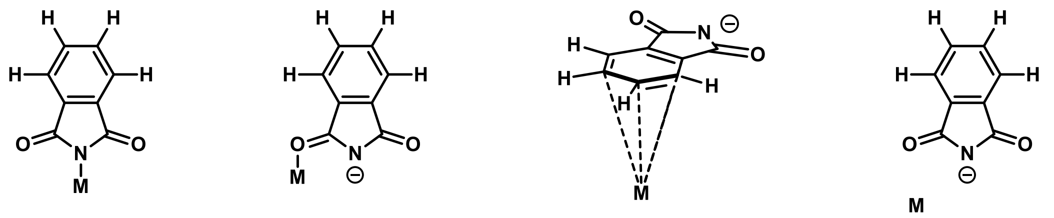

	Coordinated			Uncoordinated
	N-bound	O-bound	π-bound	
Search constraints:		Set N atom connectivity = 2	Set C-N bond type = π bond<br>Set N atom connectivity = 2	Set N atom connectivity = 2
Found Structures:	49	0	0	0

As described in the literature, α^{TM} can be calculated using the following equation:

$$\alpha^{TM} = \log(c/u)$$

Where c = number of structures where the ligand is found coordinated to the metal, and u = the number of structures where the ligand is found uncoordinated to the metal.

As no uncoordinated structures could be found, α^{TM} for phthalimide was calculated using the simplified formula:

$$\alpha^{TM} = \log(c) = 1.7$$

11.2 Calculation for tetrafluoroborate α^{Bi}

The following structures were searched using ConQuest 2021.2.0 from the Cambridge Structural Database (version CSD 5.42) with the following constraints to obtain the c and u values for tetrafluoroborate with bismuth.

	Coordinated	Any Structure
	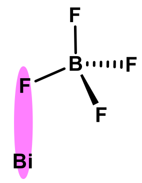	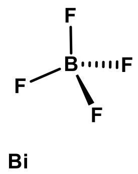
Search constraints:	Set Bi–F to intermolecular<br>Distance range: 0–3.54 Å<br>Set F atom connectivity = unspecified	Set F atom connectivity = unspecified
Found Structures:	13	24<br>(28 with 4 duplicates)

The search for coordinated structures resulted in 13 hits.[16]
This search for any structure resulted in 28 hits, 4 of which were duplicates,[17] to give 24 unique structures. Of these, 13 hits overlapped with the coordinated search.[16] Of the remaining, one hit[18] showed a polymeric structure where the Bi-F bond was shorter than the sum of the van der Waals radii, and was added to the count for coordinated structures. In total, 14 coordinated structures were found and 10 uncoordinated.[19] With this, the coordinating ability index for tetrafluoroborate to bismuth (α^{Bi}) could be calculated as follows:

$$\alpha^{Bi} = \log(c/u) = 0.1$$

From the above search, the structures found with $[BF_4]^-$ coordinated to a Bi center included complexes with Bi in the +III and +V oxidation states. Examples of Bi(III) compounds found in the search include bismuthino cations,[20–23] and complexes featuring bismuth in the backbone of chelating ligands coordinated to charged transition metals.[24,25] Examples of Bi(V) compounds found in the search include bismuthonium salts[26–31] and aryl bismuth oxide.[32] While no examples of Bi(II) were found, we would like to emphasize that α is a statistical parameter that does not account for oxidation state.

13. NMR Spectra

Figure S39: Stacked ^{1}H NMR spectra (300 MHz, MeCN-d3) showing the aromatic region for **1**, **2OTf** and an aliquot of the reaction mixture of the disproportionation of **2OTf** using $[NBu_4][Cl]$.

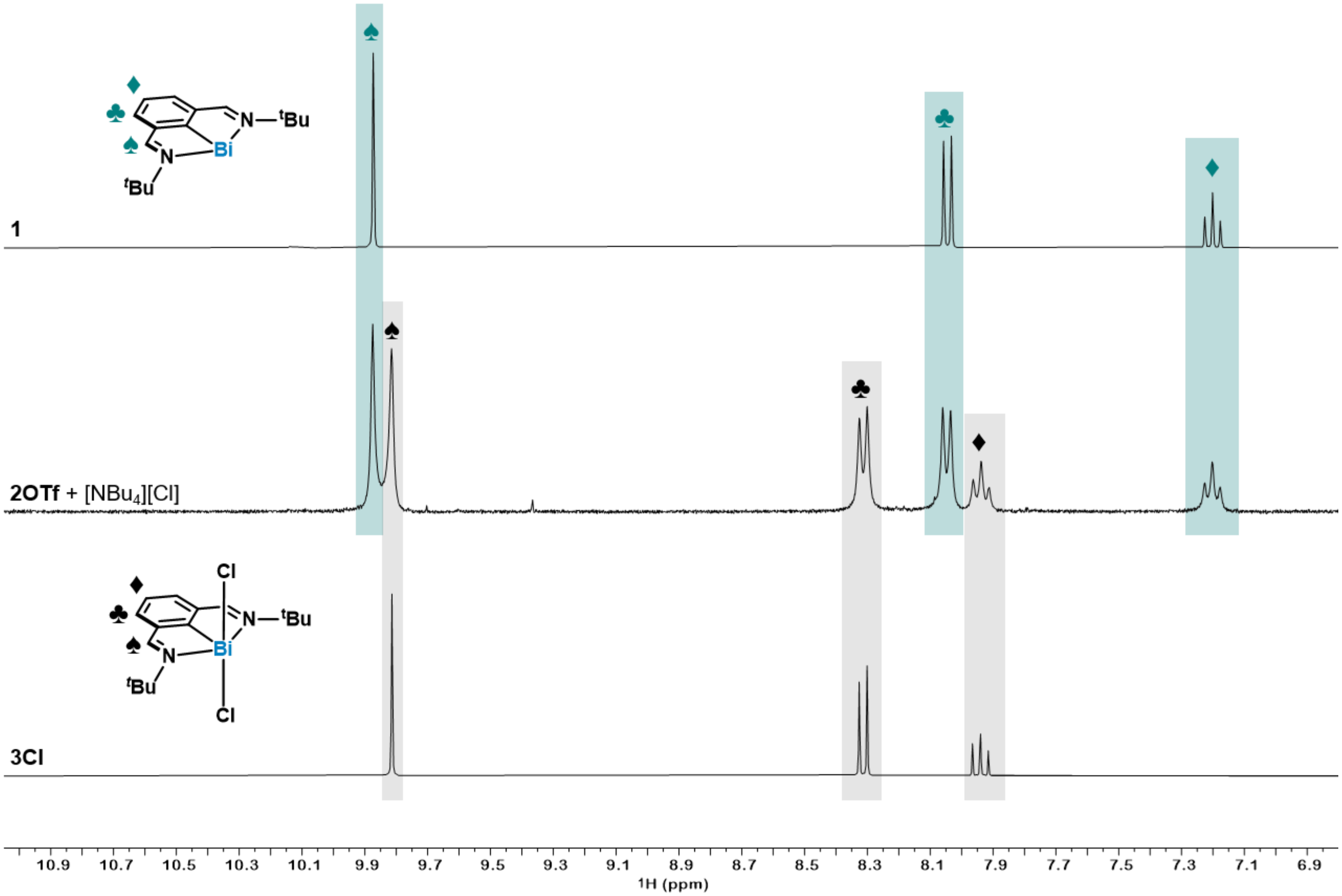

Figure S40: Stacked ^{1}H NMR spectra (MeCN-d3) showing the aromatic region for **1** (300 MHz, 25°C), **3F** (300 MHz, 25°C) and an aliquot of the reaction mixture of the disproportionation of **2OTf** using [Cp2Co][F] (600 MHz, -40°C).

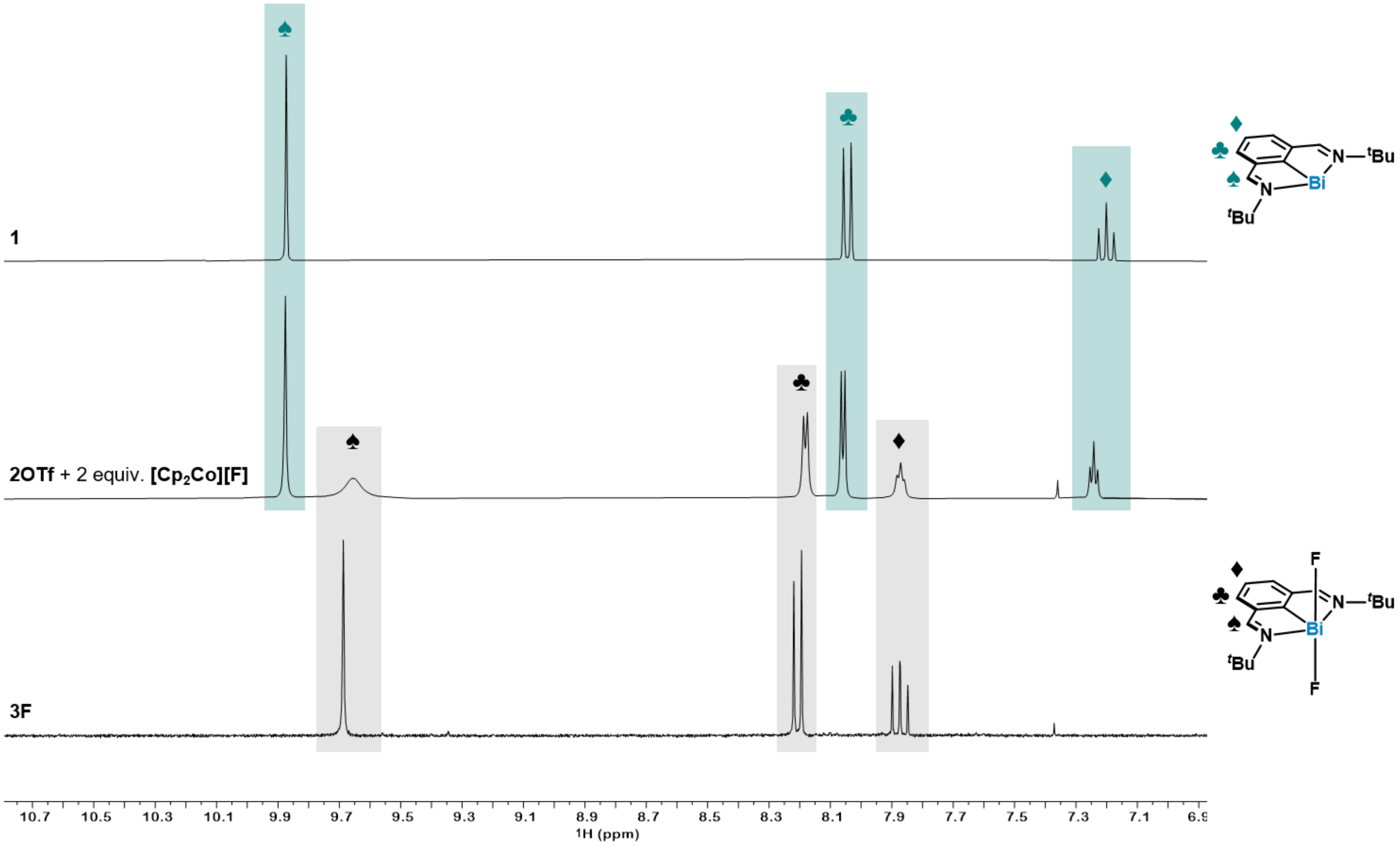

Figure S41: ^{1}H NMR spectrum (600 MHz, MeCN-d$_3$) of **3Br**.

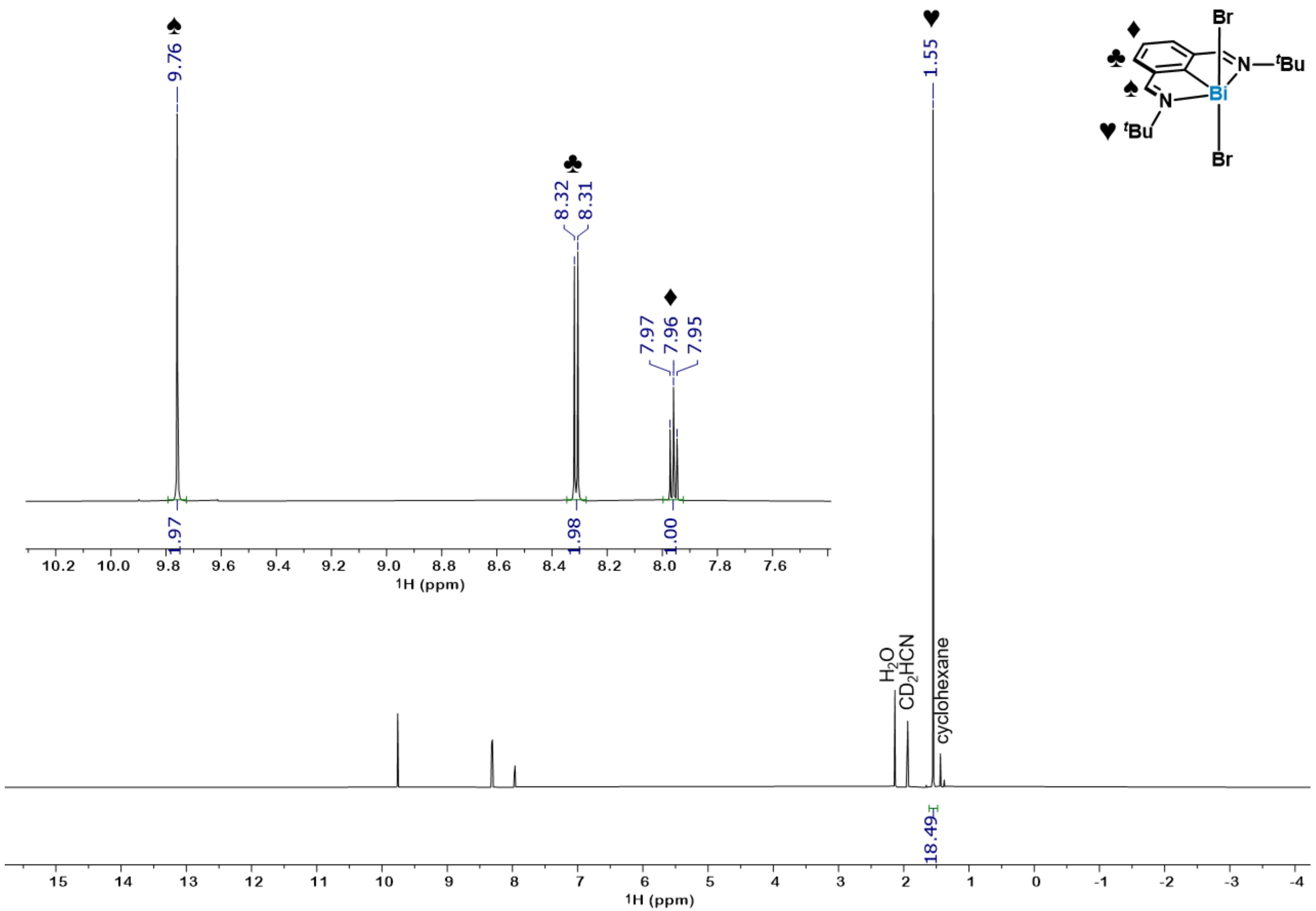

Figure S42: Stacked ^{1}H NMR spectra (MeCN-d_3) showing the aromatic region for **1** (300 MHz, 25°C), **3Br** (600 MHz, -40°C) and an aliquot of the reaction mixture of the disproportionation of **2OTf** using $[NBu_4][Br]$ (600 MHz, 25°C).

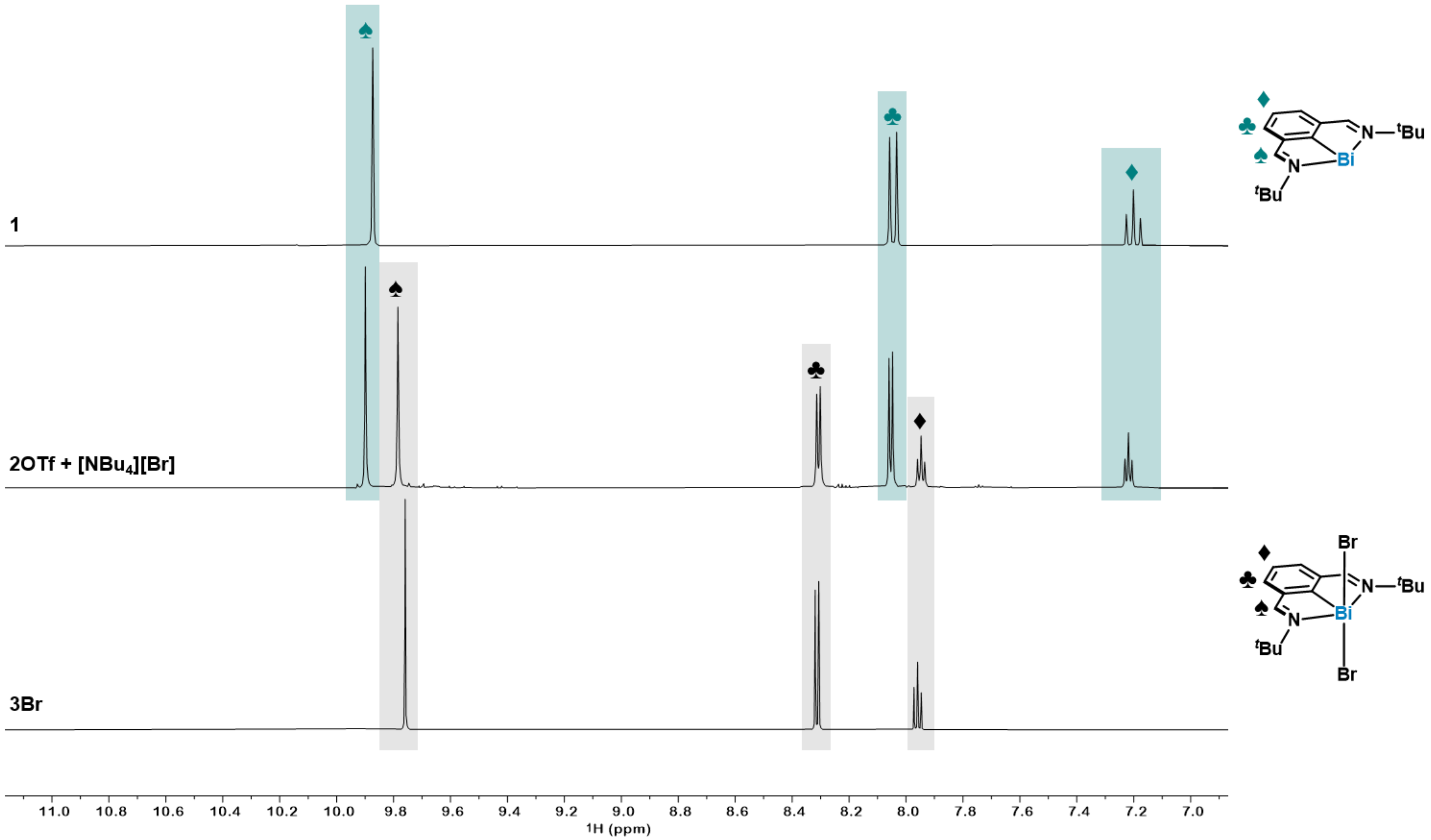

Figure S43: $^{13}C\{^{1}H\}$ NMR spectrum (150.94 MHz, MeCN-d_3) of **3Br**.

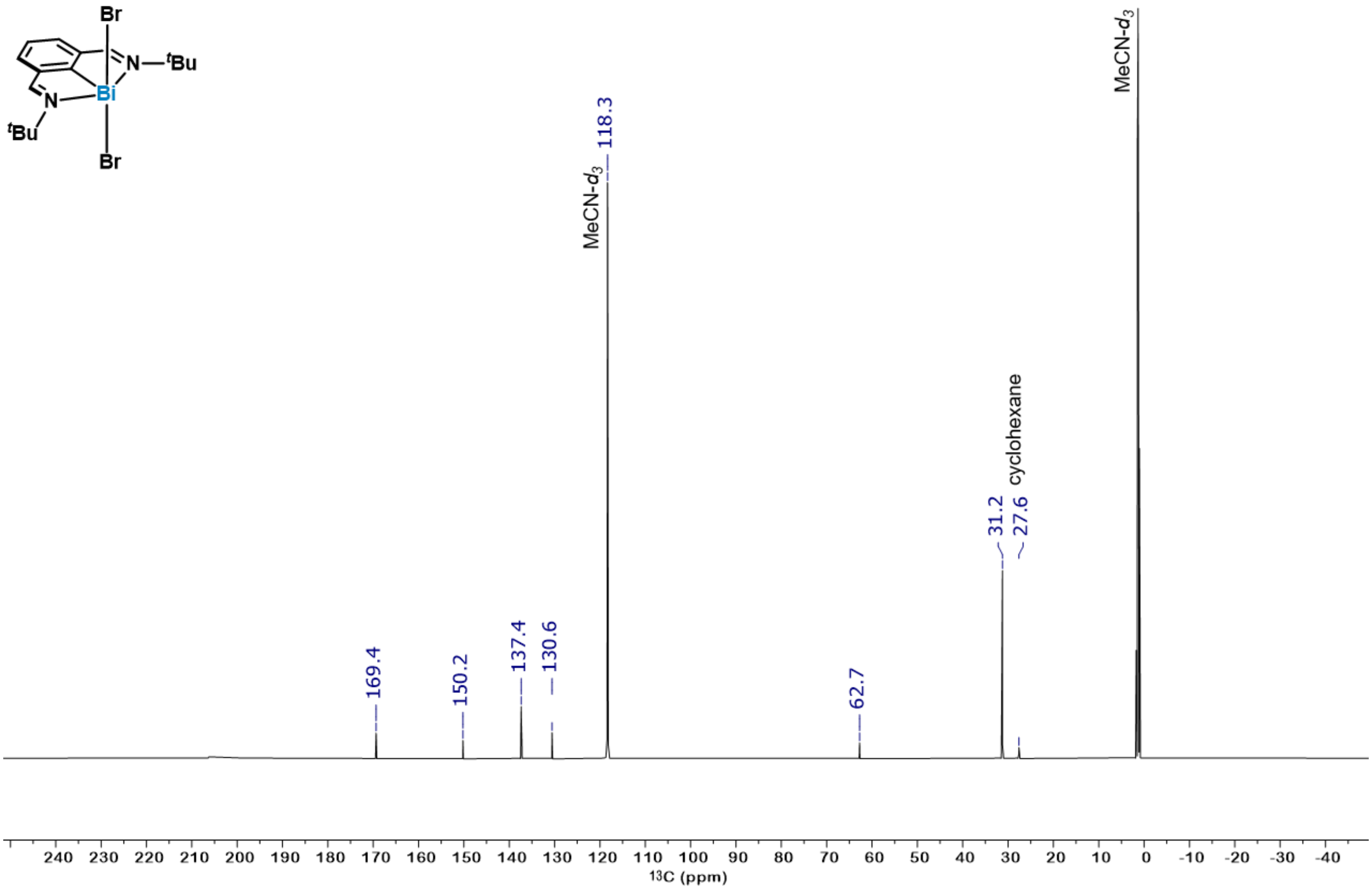

Figure S44: Stacked ^{1}H NMR spectra (DMF-d$_7$) showing the aromatic region for **1** (300 MHz, 25°C), **3I** (600 MHz, -40°C) and an aliquot of the reaction mixture of the disproportionation of **2OTf** using [NBu$_4$][I] (600 MHz, 25°C and -40°C).

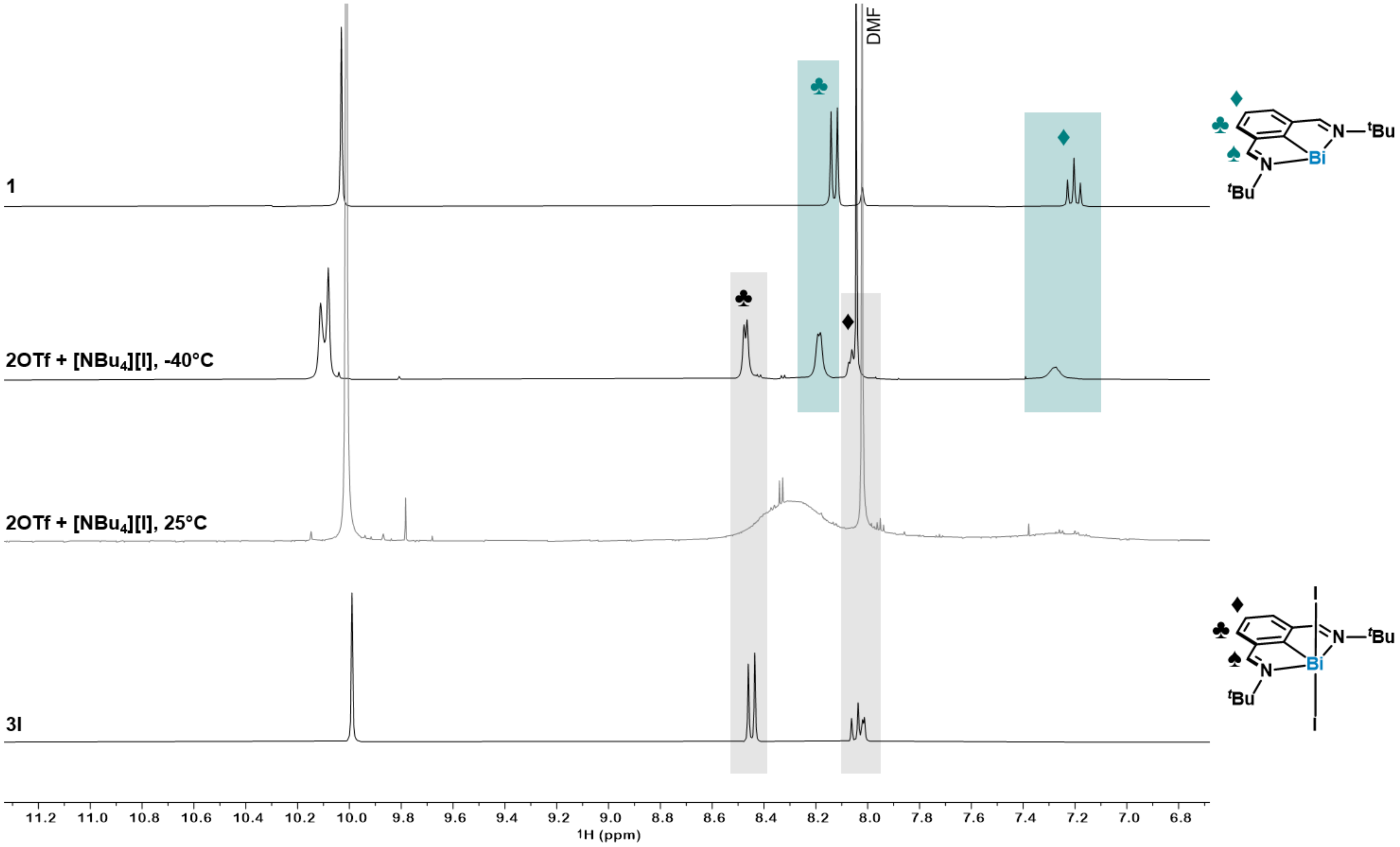

Figure S45: Stacked ^{1}H NMR spectra (MeCN-d$_3$) showing the aromatic region for **1** (300 MHz, 25°C), **3OAc** (600 MHz, -40°C) and an aliquot of the reaction mixture of the disproportionation of **2OTf** using [NBu$_4$][OAc] (600 MHz, -40°C).

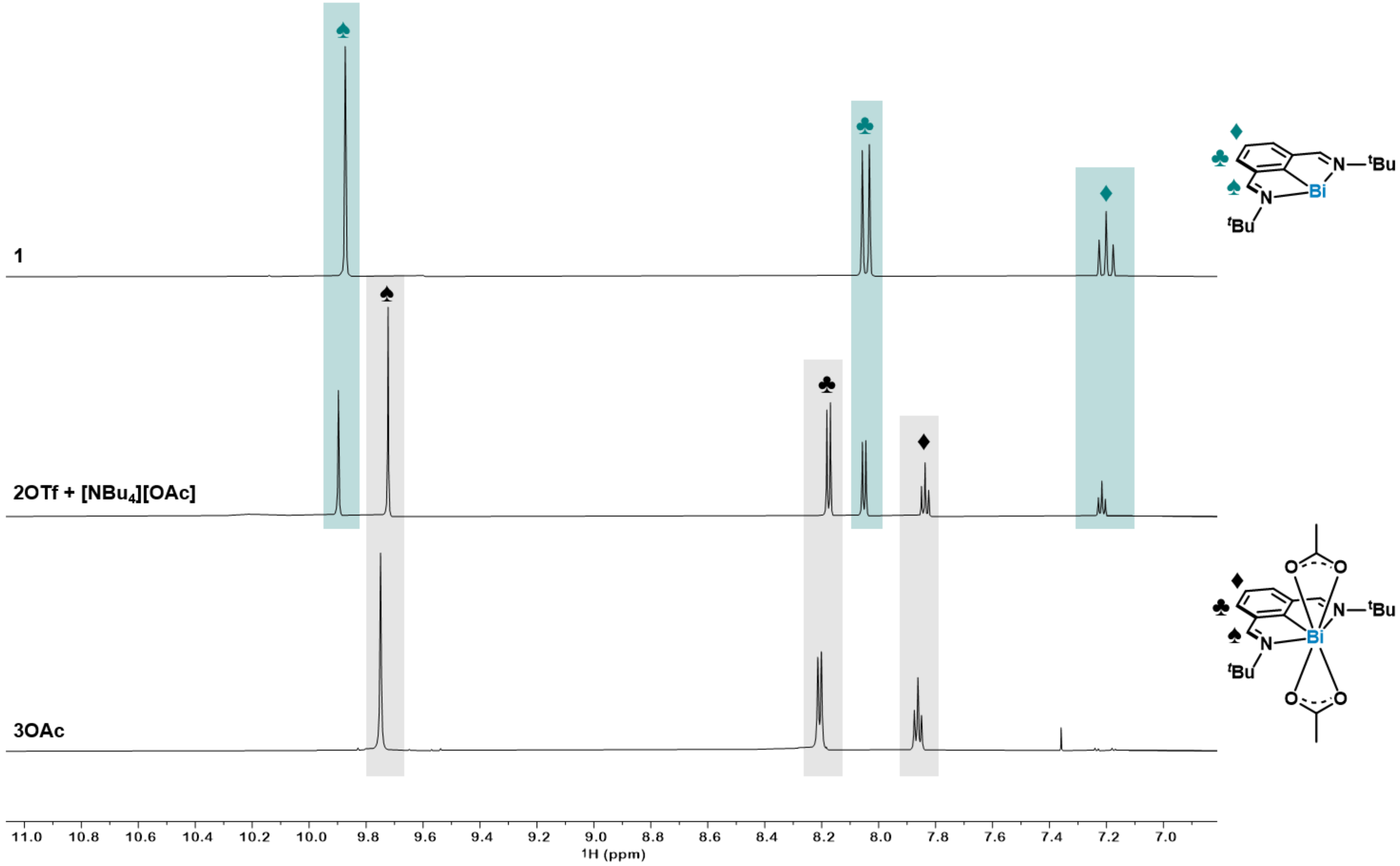

Figure S46: ^{1}H NMR spectrum (600.20 MHz, MeCN-d_3) of **3OBz**.

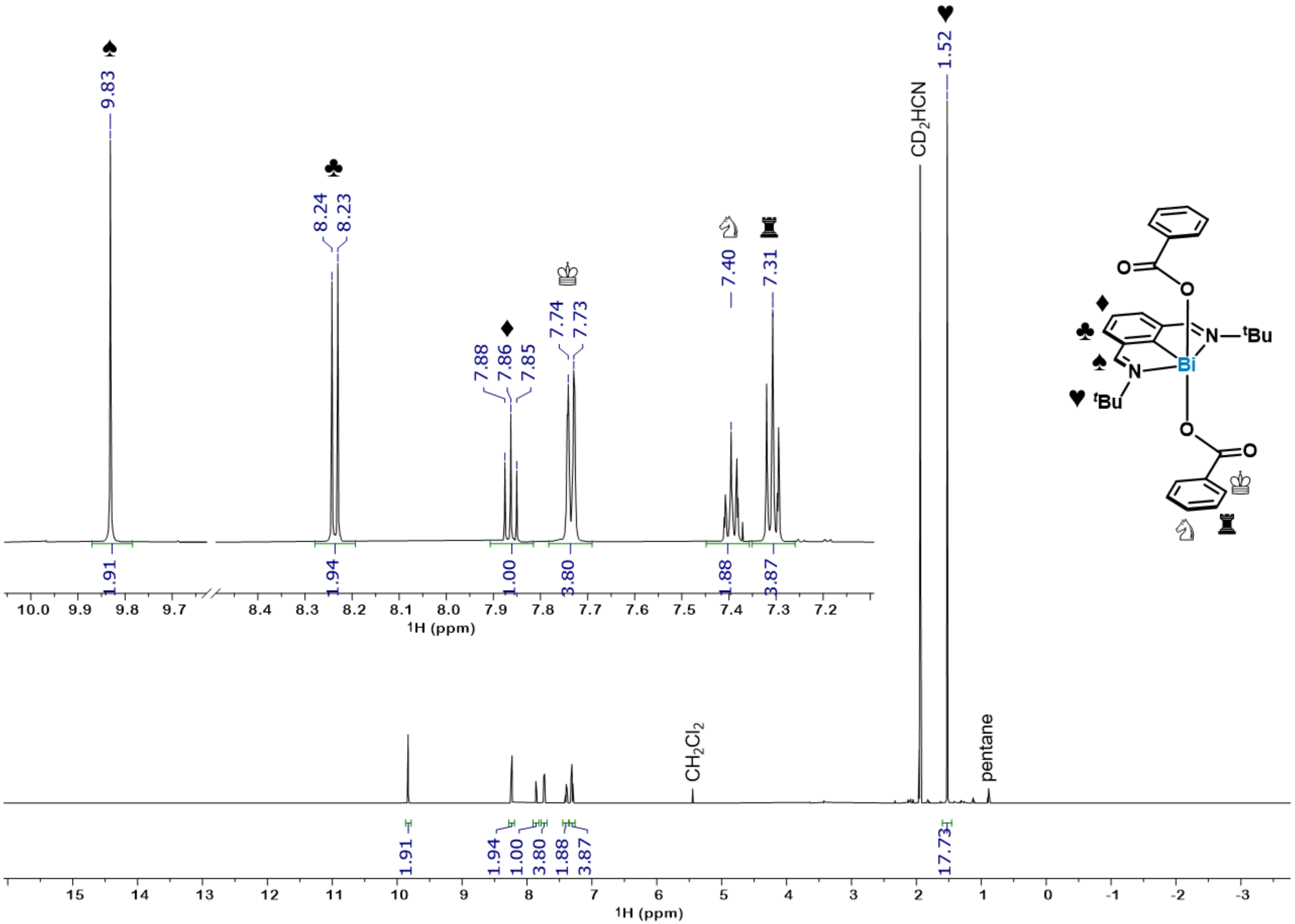

Figure S47: Stacked ^{1}H NMR spectra (300 MHz, MeCN-d$_3$) showing the aromatic region for **1**, **3OBz** and an aliquot of the reaction mixture of the disproportionation of **2OTf** using [NBu$_4$][OBz].

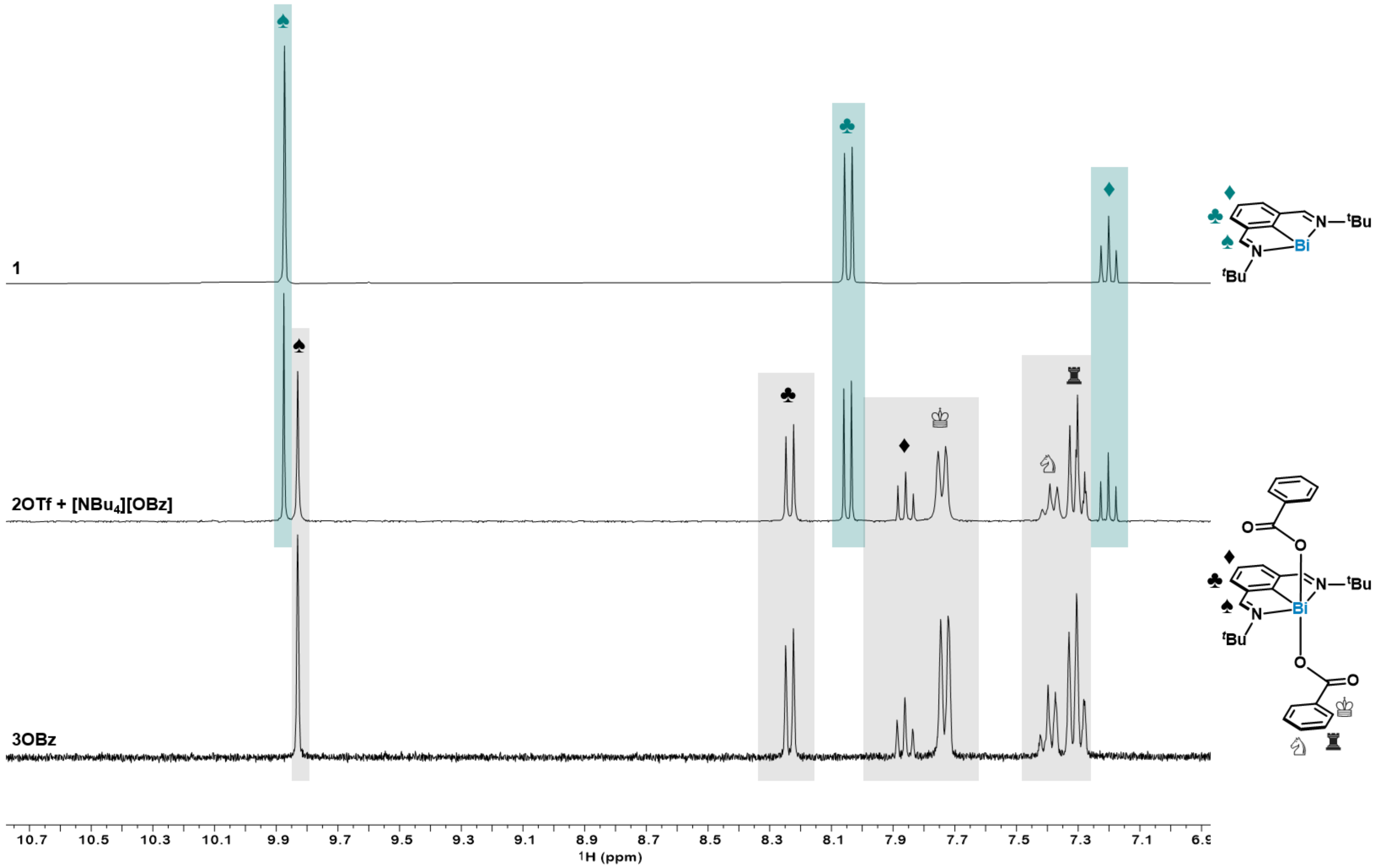

Figure S48: $^{13}C\{^1H\}$ NMR spectrum (150.94 MHz, MeCN-d_3) of **3OBz**.

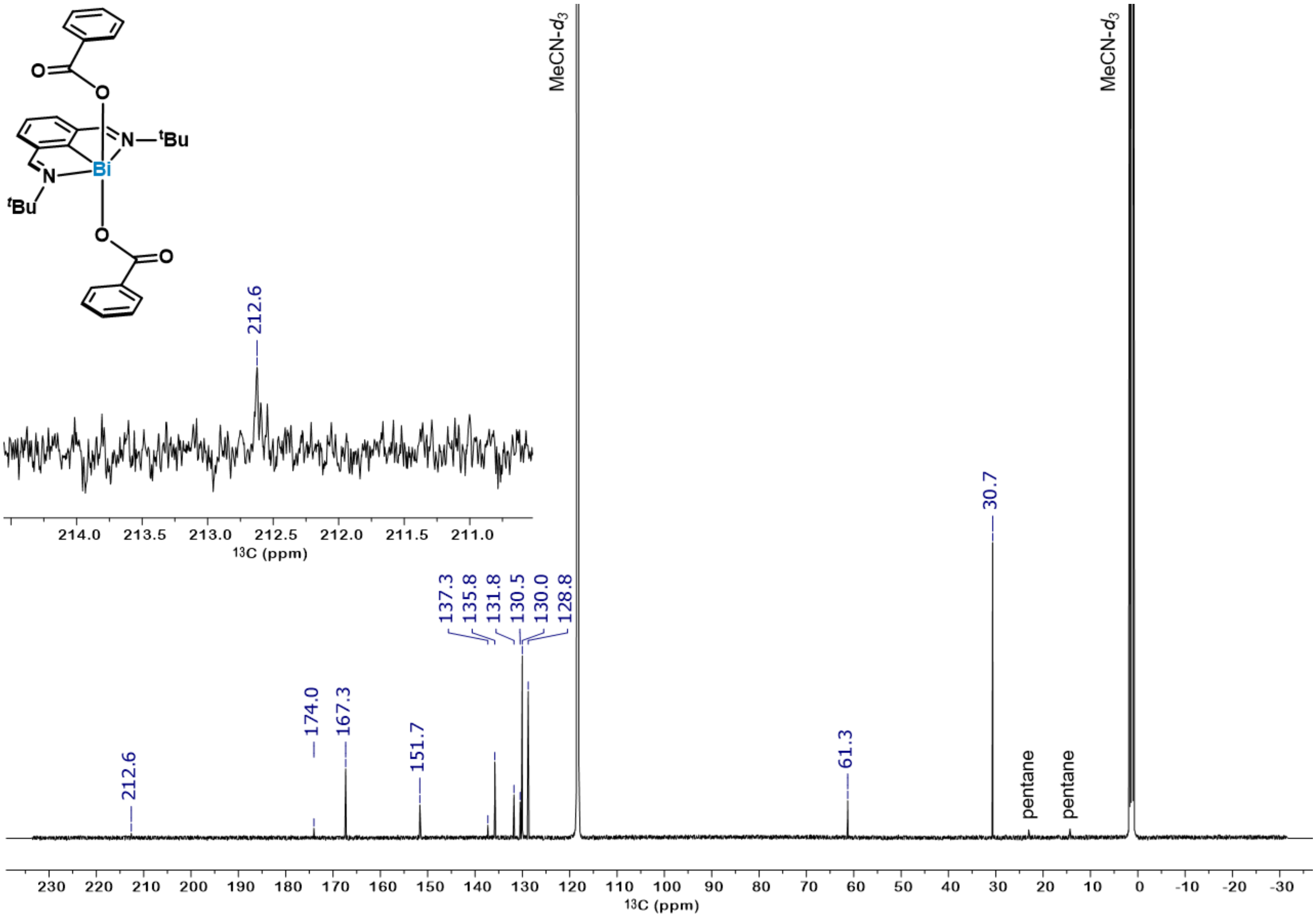

Figure S49: ^{1}H NMR spectrum (600.20 MHz, MeCN-d$_3$) of **3OPh**.

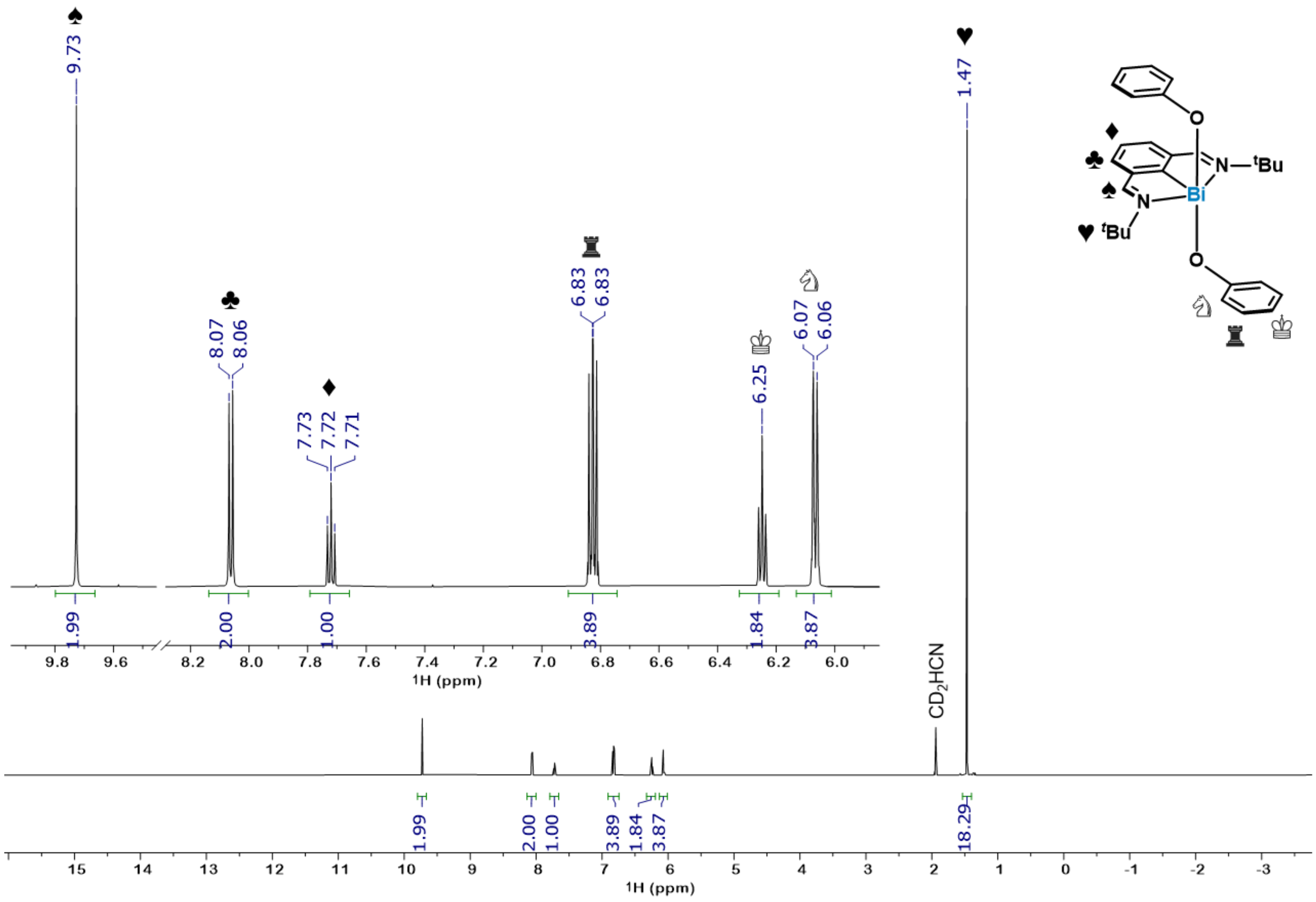

Figure S50: Stacked ^{1}H NMR spectra (300 MHz, MeCN-d_3) showing the aromatic region for **1**, **3OPh** and an aliquot of the reaction mixture of the disproportionation of **2OTf** using NaOPh. TMB = 1,3,5-trimethoxybenzene, used as internal standard.

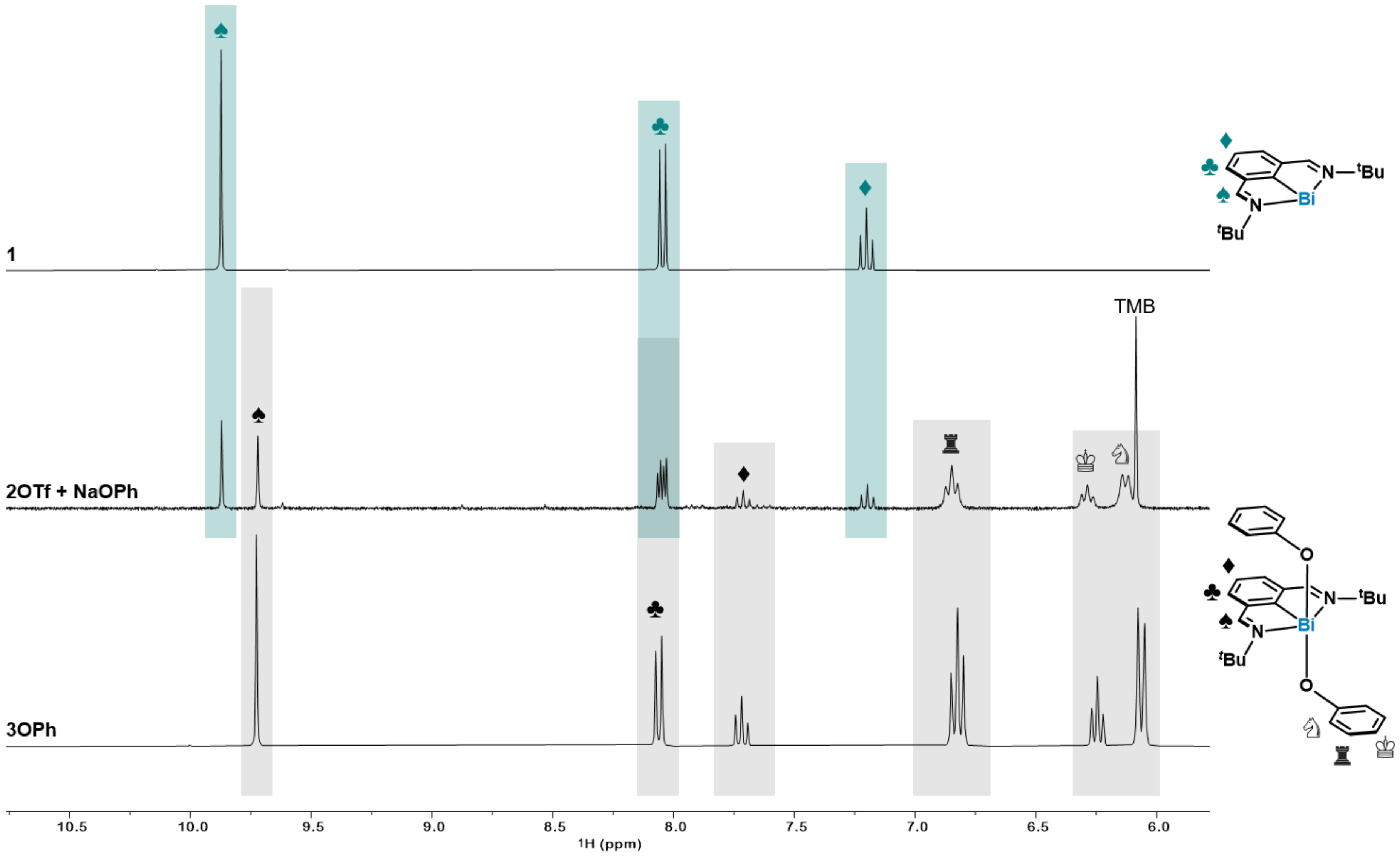

Figure S51: $^{13}C\{^{1}H\}$ NMR spectrum (150.94 MHz, MeCN-d_3) of **3OPh**.

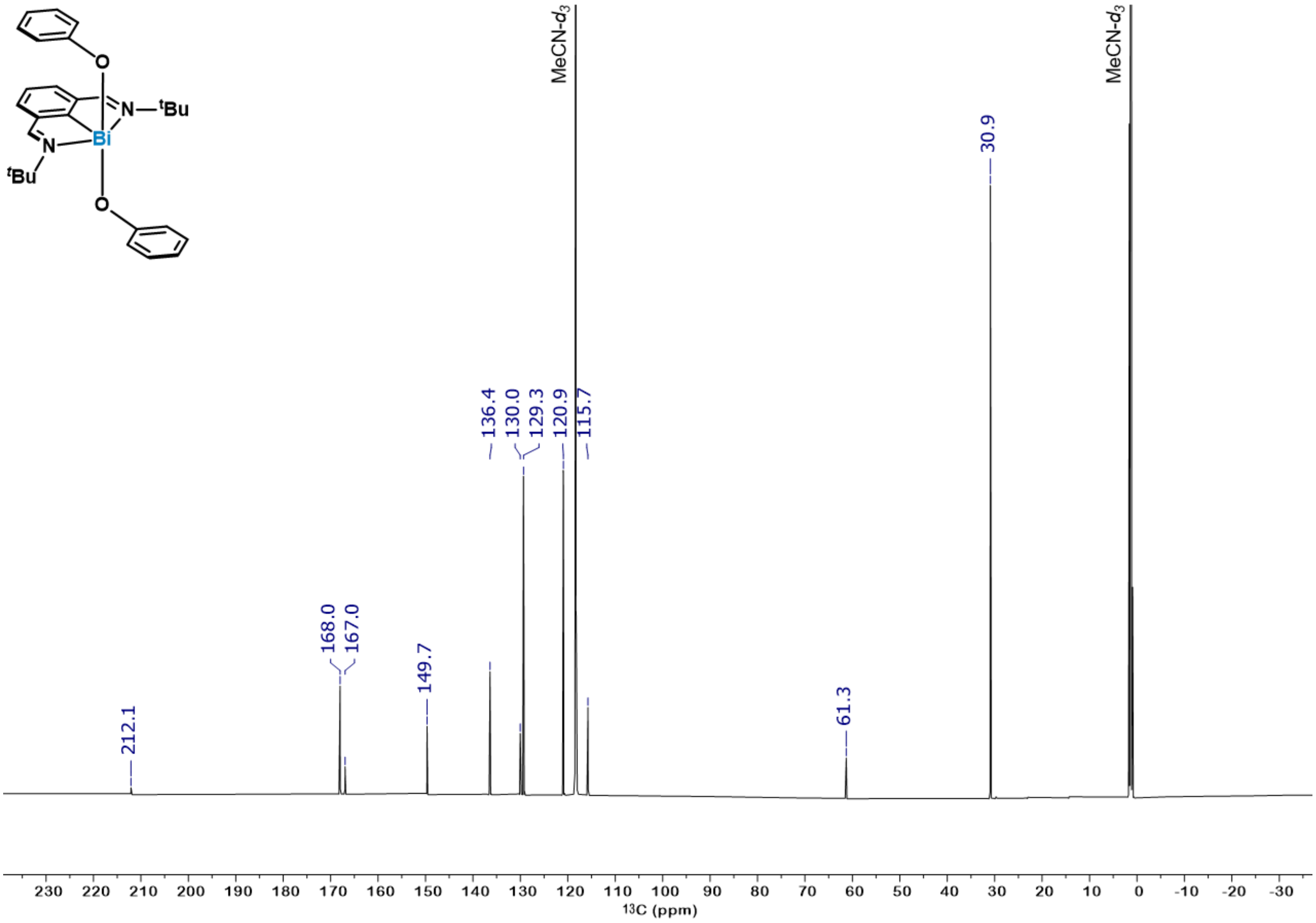

Figure S52: ^{1}H NMR spectrum (600.20 MHz, MeCN-d$_3$) of **3SPh**.

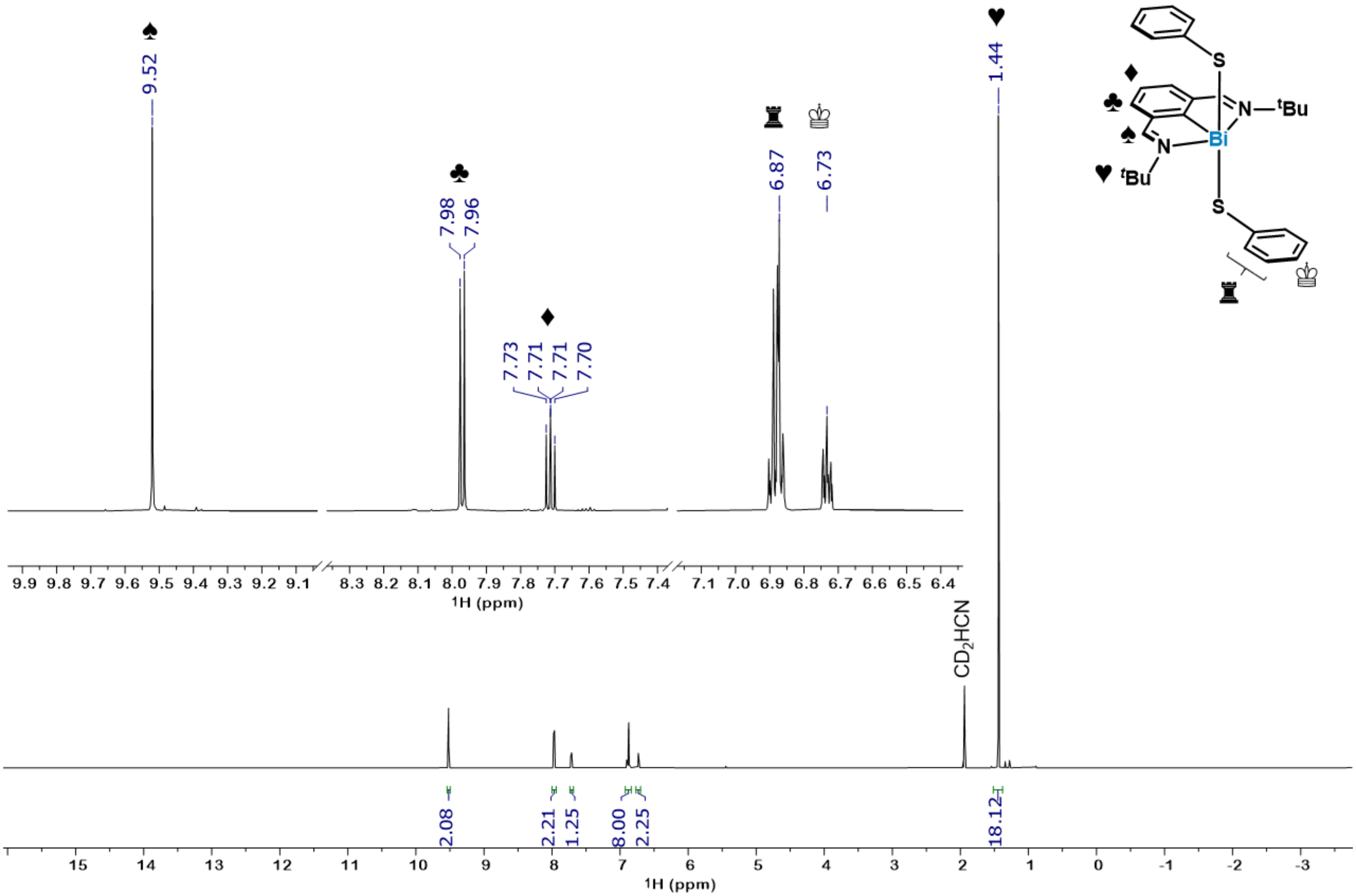

Figure S53: Stacked ^{1}H NMR spectra (300 MHz, MeCN-d_3) showing the aromatic region for **1**, **3SPh** and an aliquot of the reaction mixture of the disproportionation of **2OTf** using NaSPh.

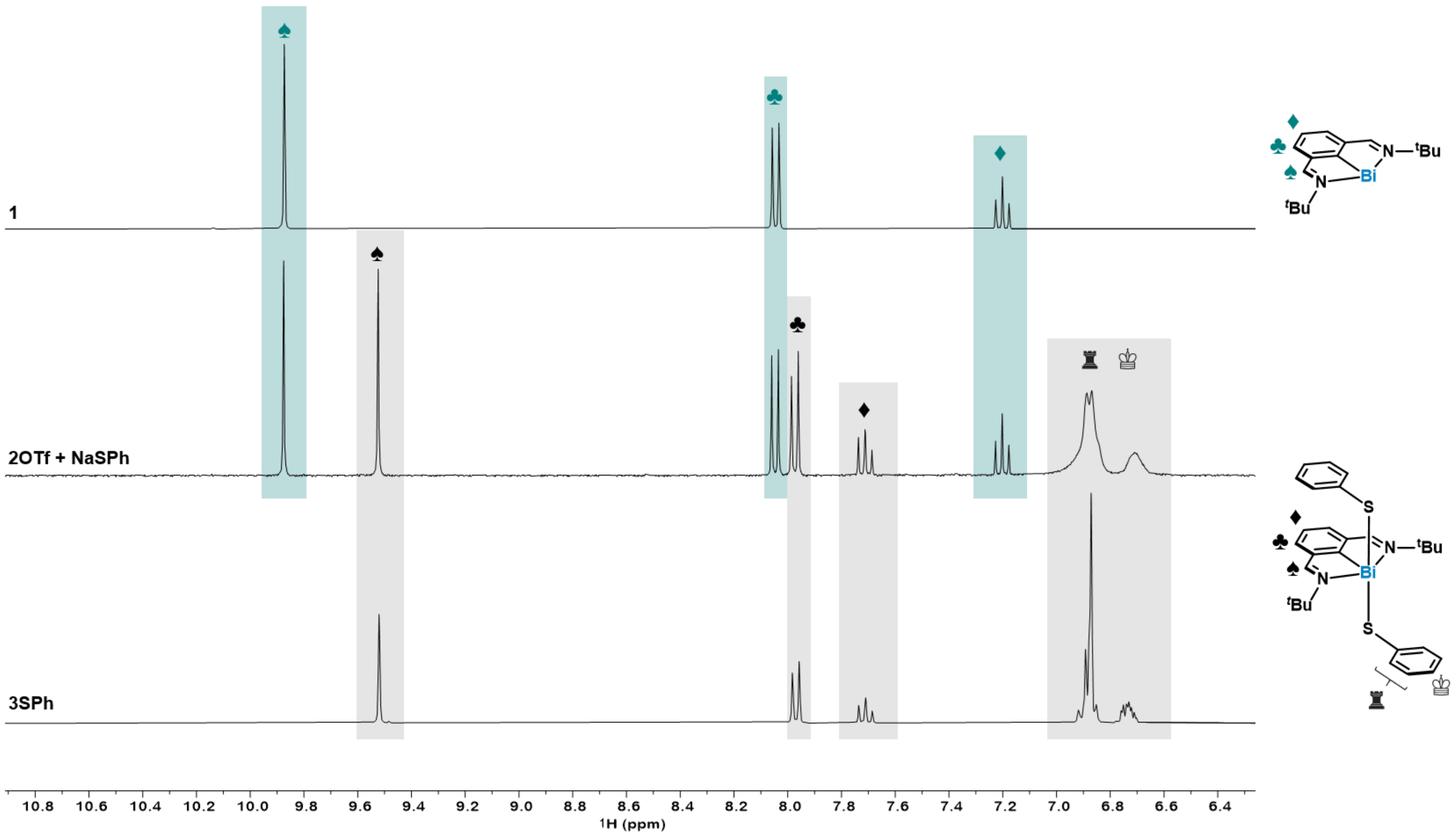

Figure S54: $^{13}C\{^{1}H\}$ NMR spectrum (150.94 MHz, MeCN-d_3) of **3SPh**.

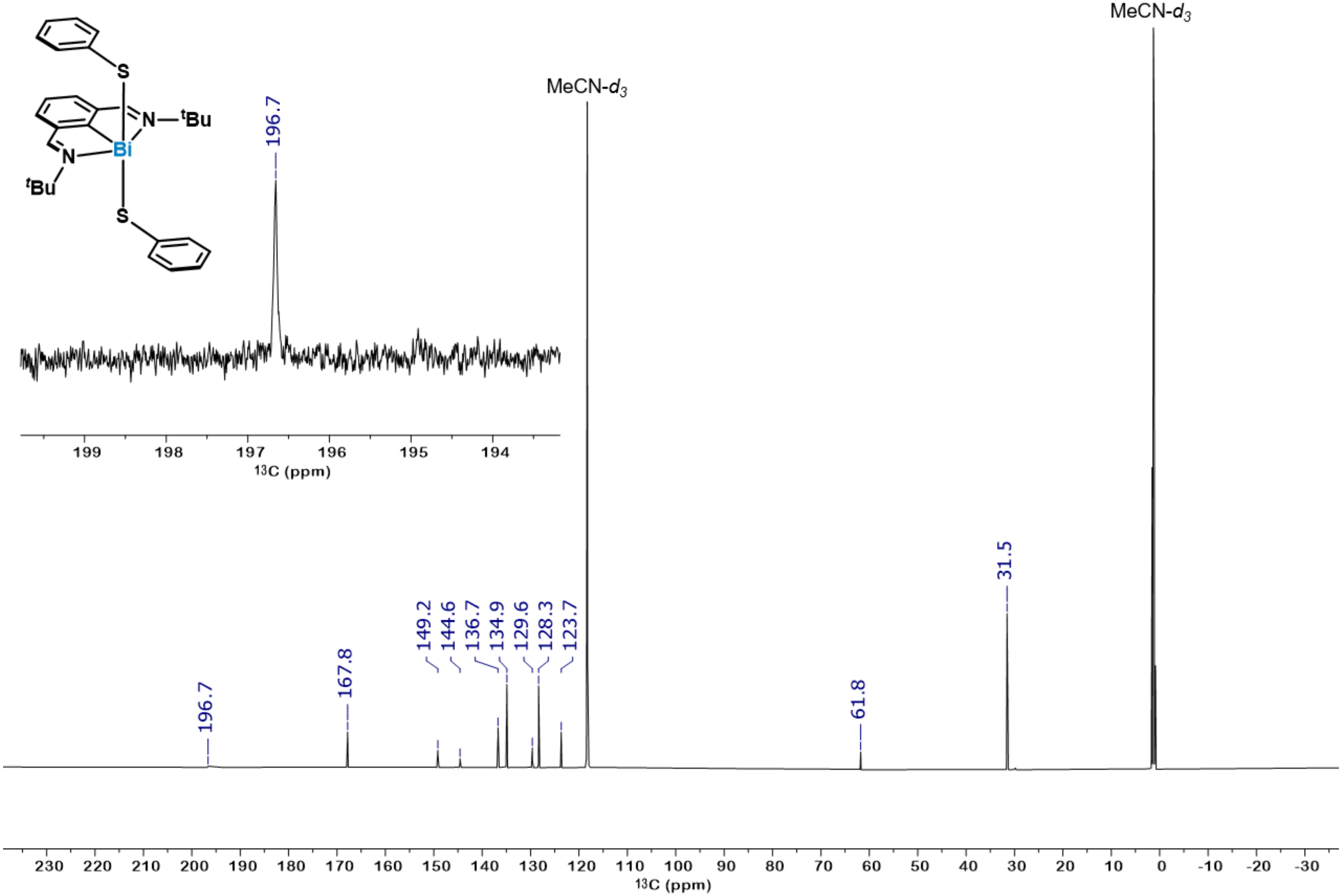

Figure S55: ^{1}H NMR spectrum (600.22 MHz, DMF-d_7) of **3Phth**.

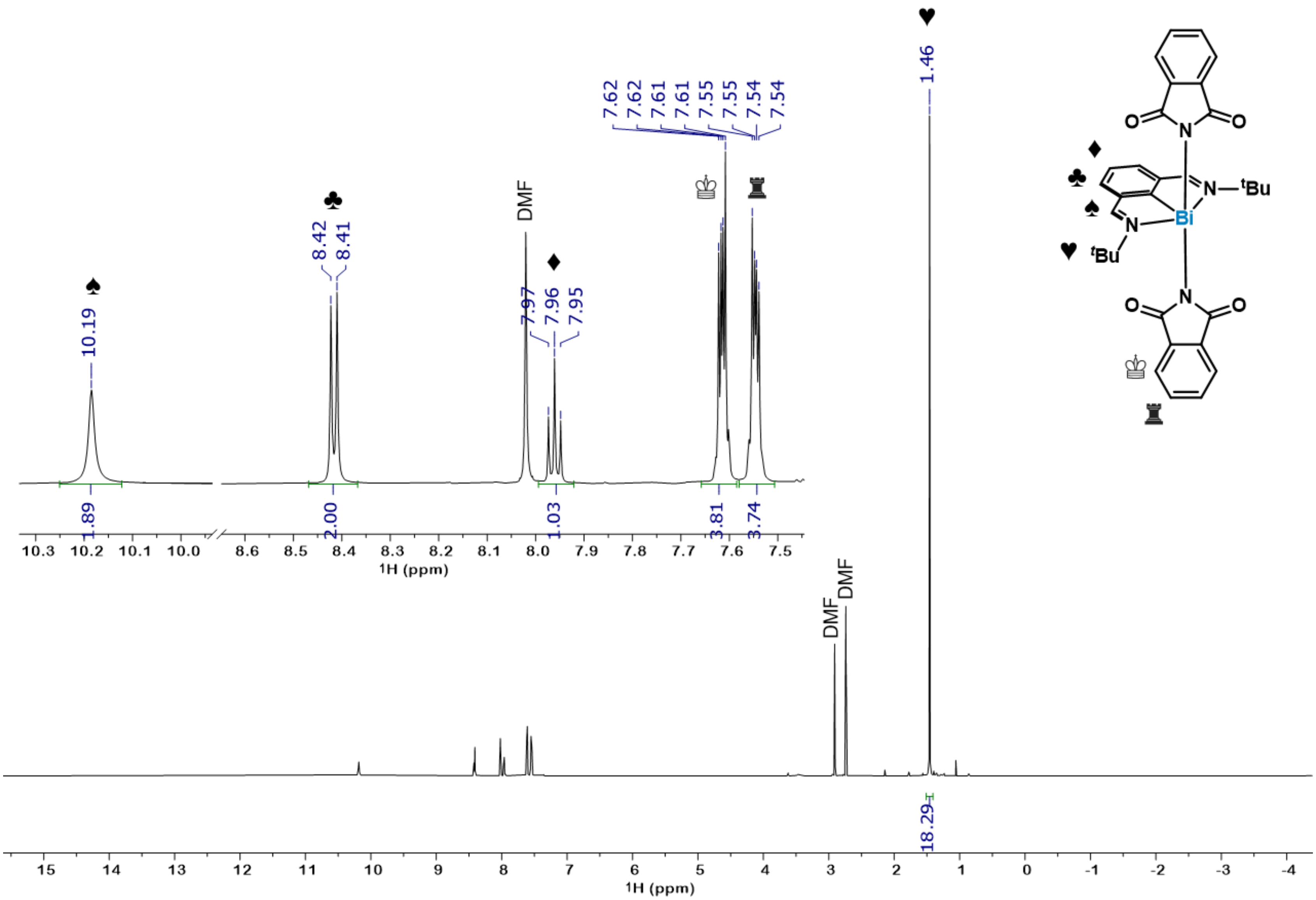

Figure S56: Stacked ^{1}H NMR spectra (300 MHz, DMF-d_7) showing the aromatic region for **1**, **3Phth** and an aliquot of the reaction mixture of the disproportionation of **2OTf** using potassium phthalimide.

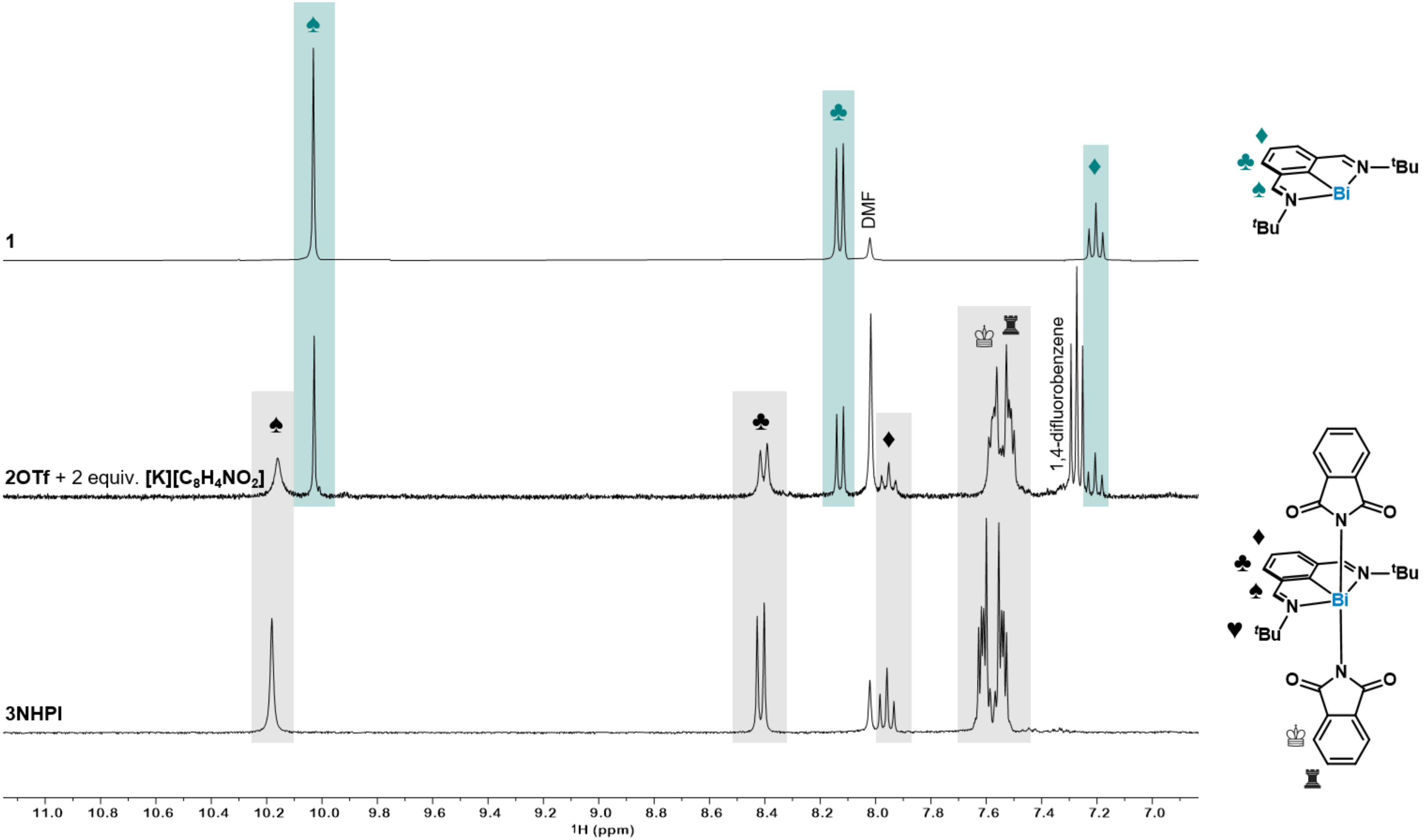

Figure S57: $^{13}C\{^{1}H\}$ NMR spectrum (300.15 MHz, DMF-d_7) of **3Phth**.

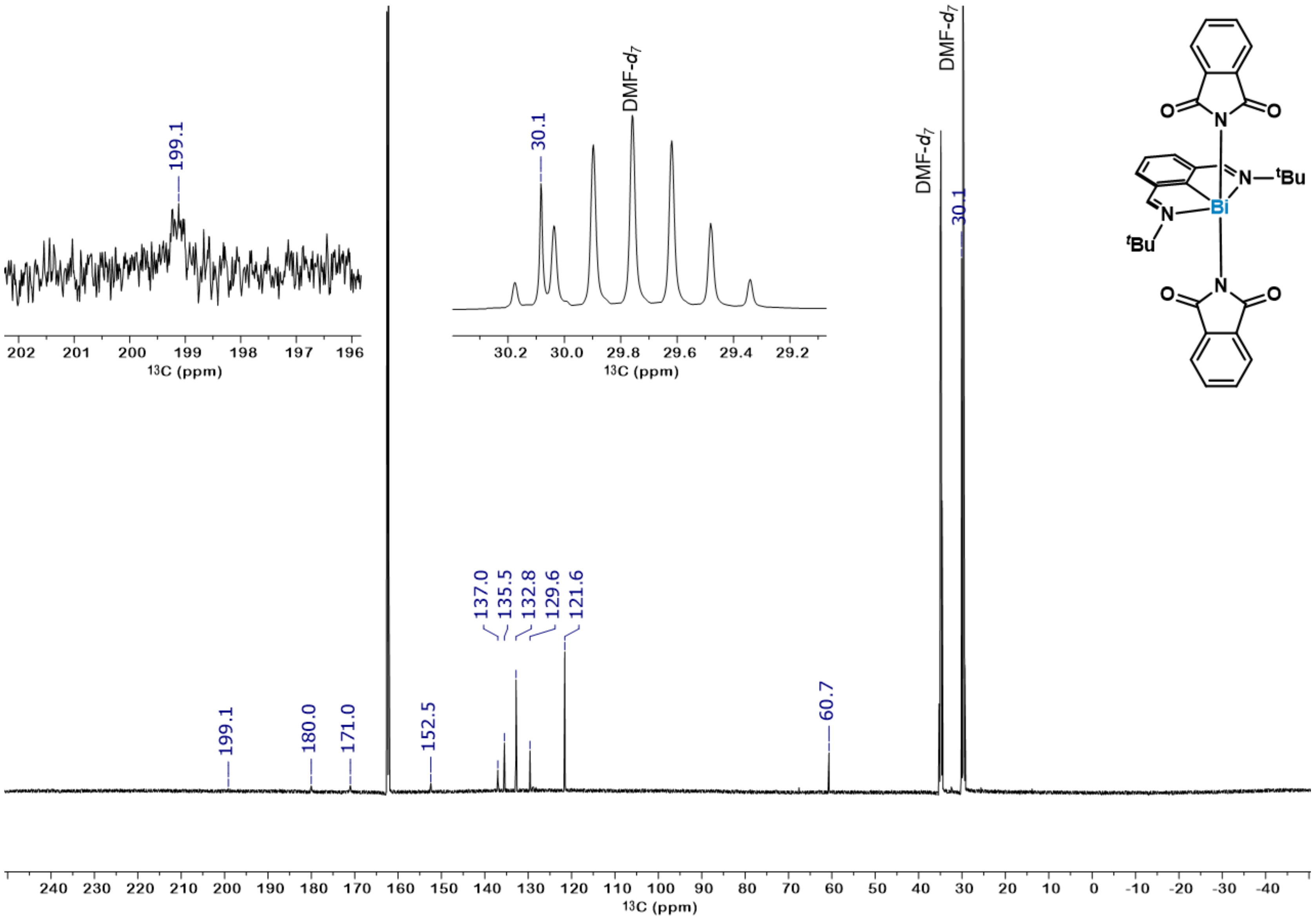

Figure S58: ^{1}H NMR spectrum (600.20 MHz, MeCN-d$_3$) of **3-Phth-OTf**.

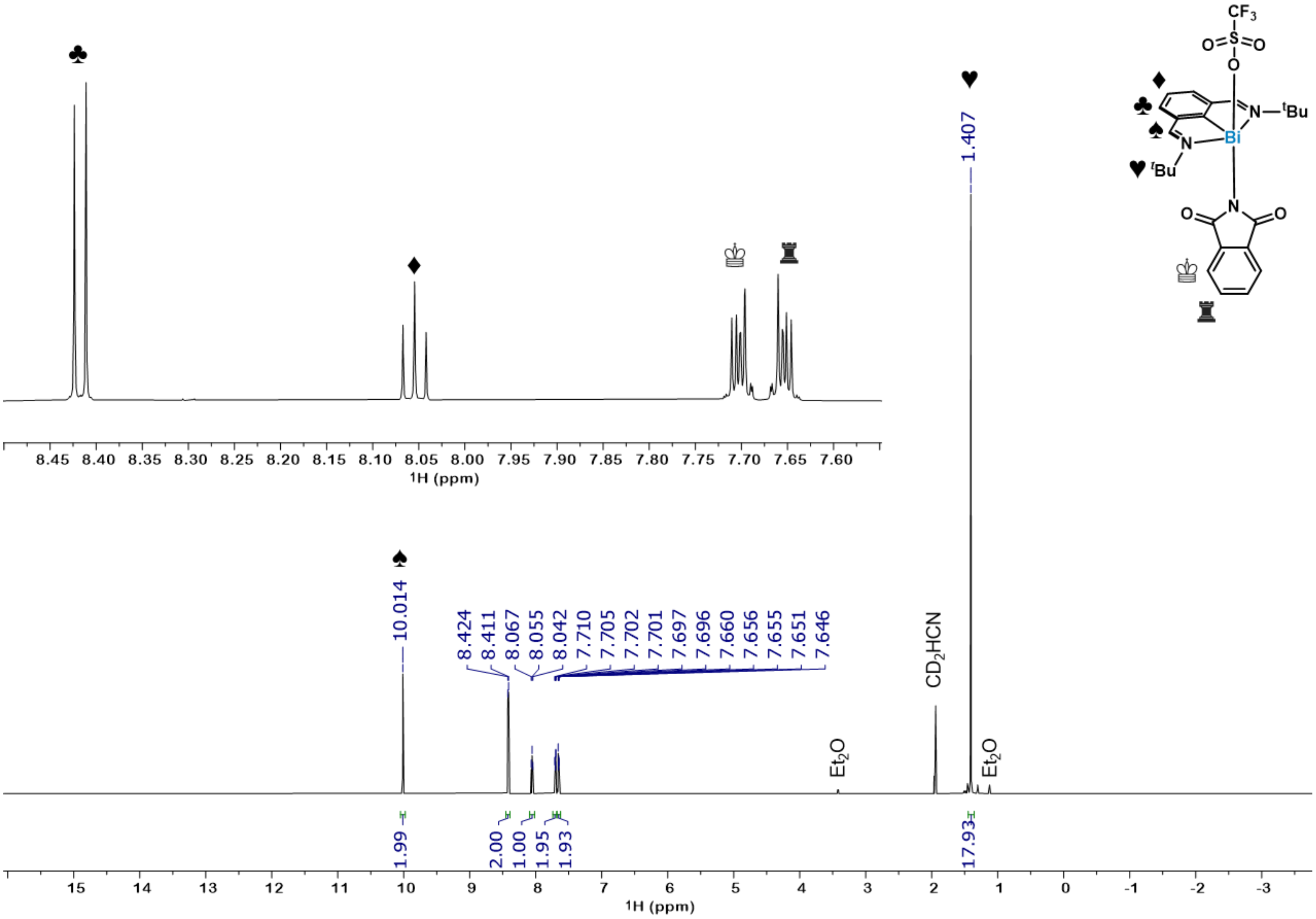

Figure S59: $^{13}C\{^{1}H\}$ NMR spectrum (150.94 MHz, MeCN-d$_3$) of **3-Phth-OTf**.

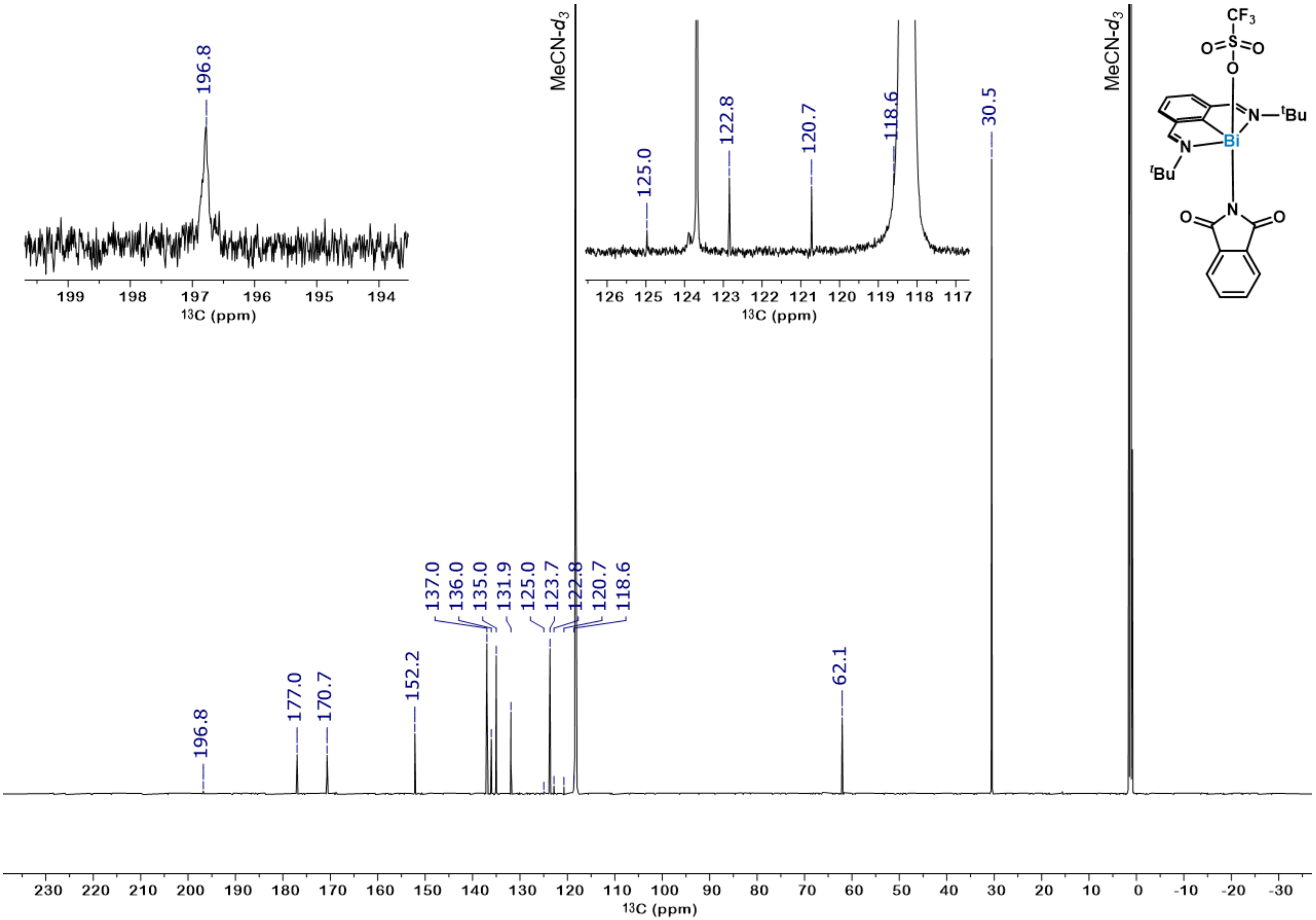

Figure S60: ^{19}F NMR spectrum (564.72 MHz, MeCN-d$_3$) of **3-Phth-OTf**.

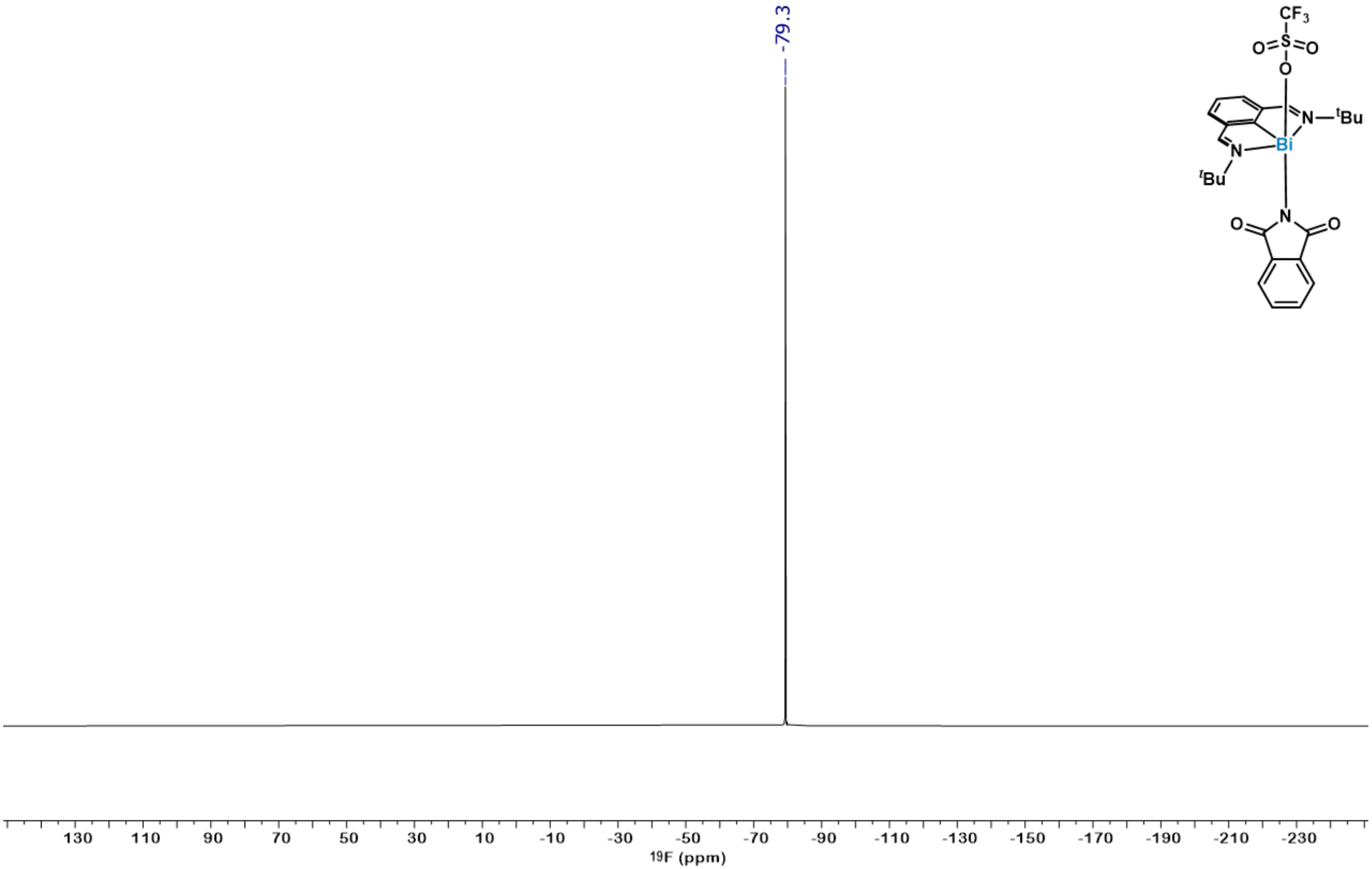

Figure S61: ^{1}H NMR spectrum (600.20 MHz, MeCN-d_3) of **3OMs**.

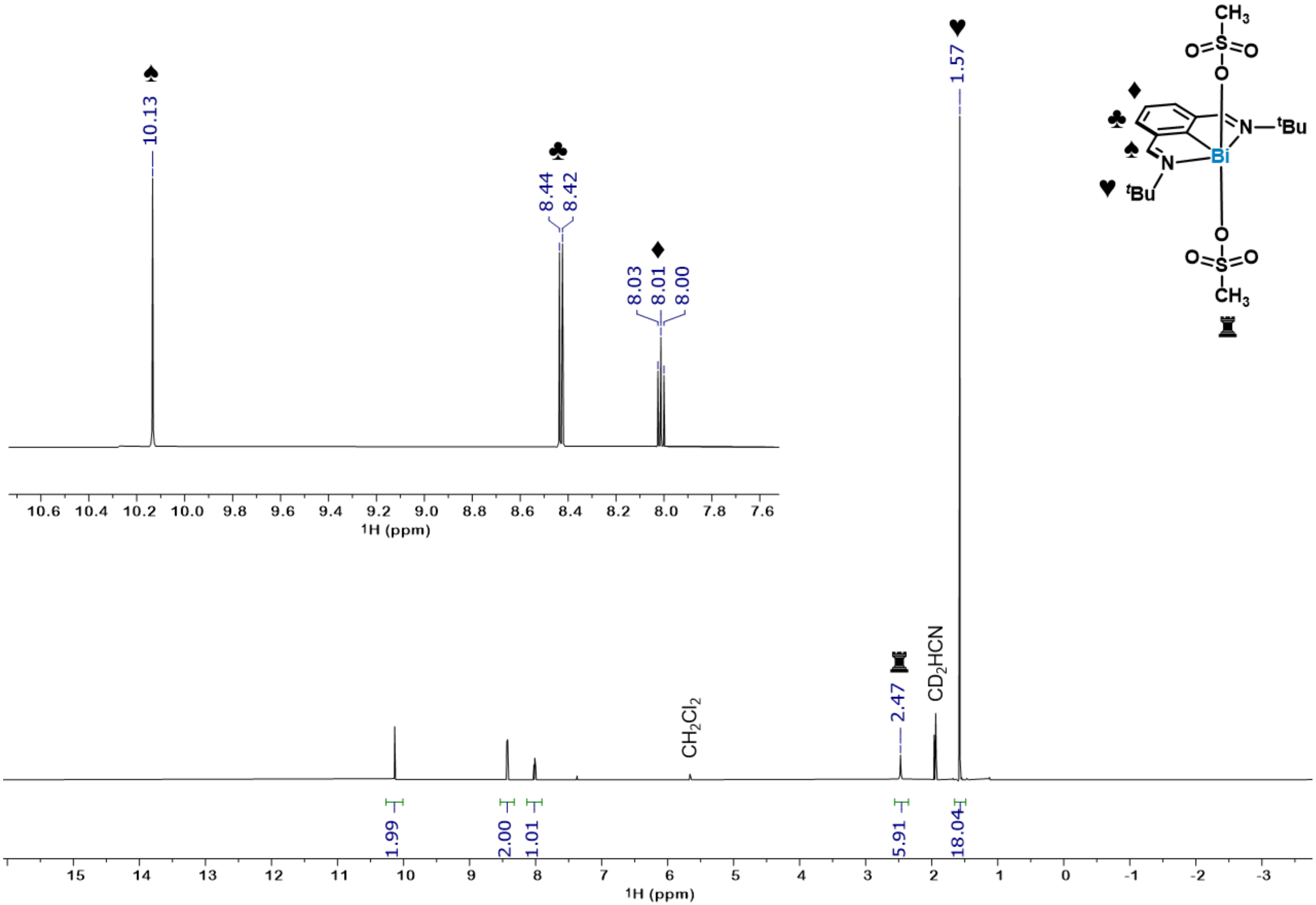

Figure S62: $^{13}C\{^{1}H\}$ NMR spectrum (150.94 MHz, MeCN-d3) of **3OMs**.

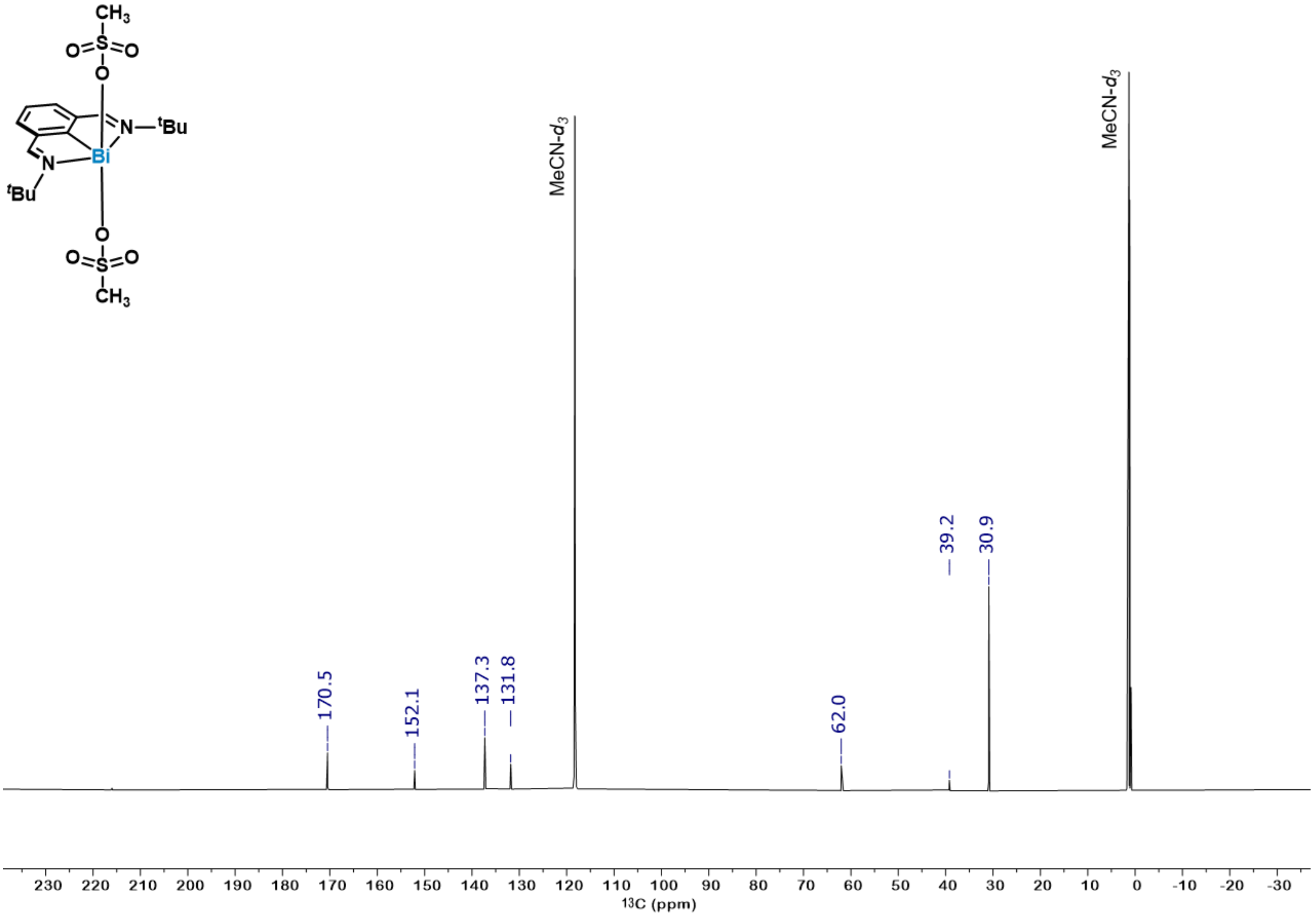

Figure S63: ^{1}H NMR spectrum (600.20 MHz, MeCN-d$_3$) of **3OTf**.

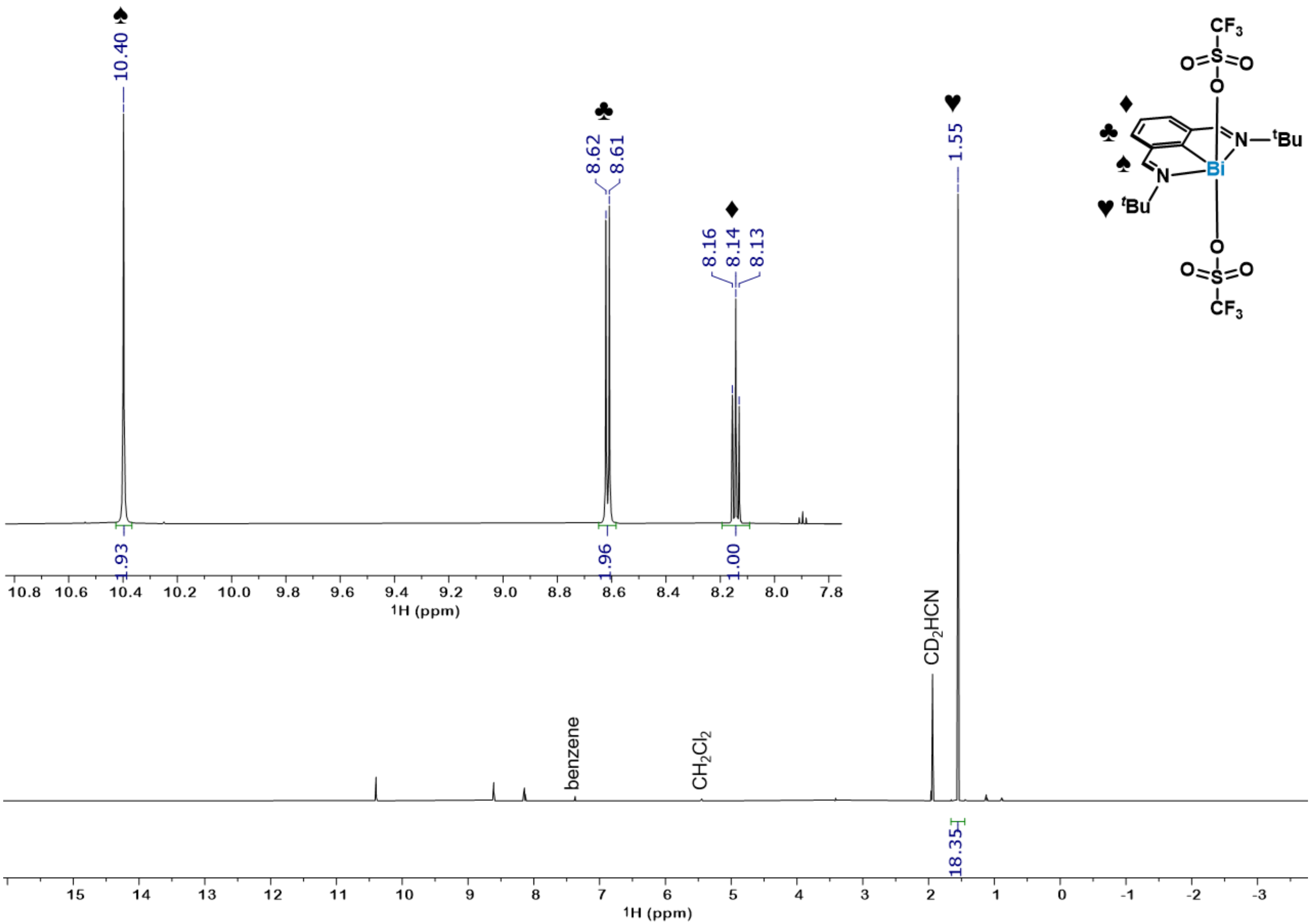

Figure S64: $^{13}C\{^{1}H\}$ NMR spectrum (150.94 MHz, MeCN-d3) of **3OTf**.

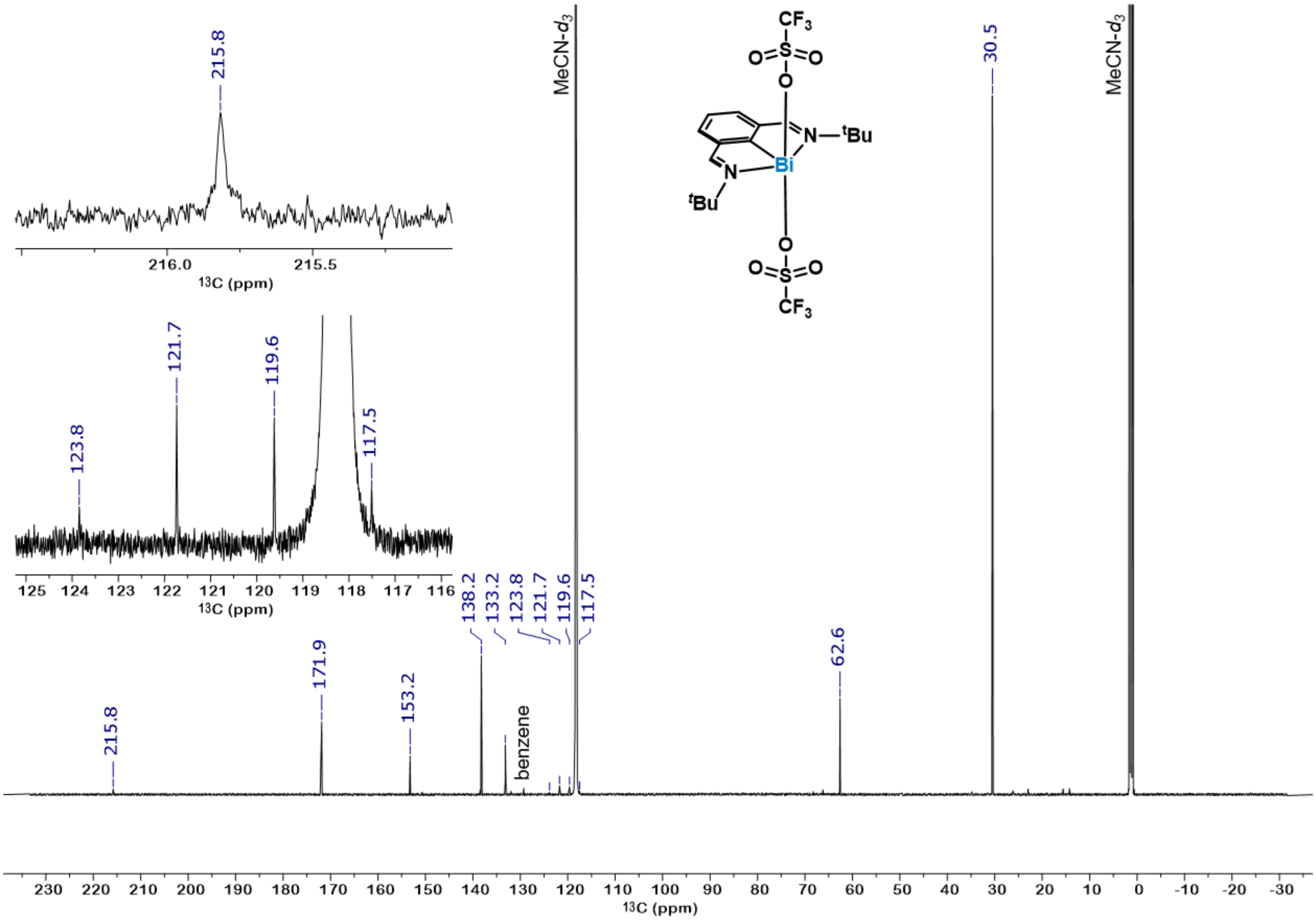

Figure S65: ^{19}F NMR spectrum (564.72 MHz, MeCN-d$_3$) of **3OTf**.

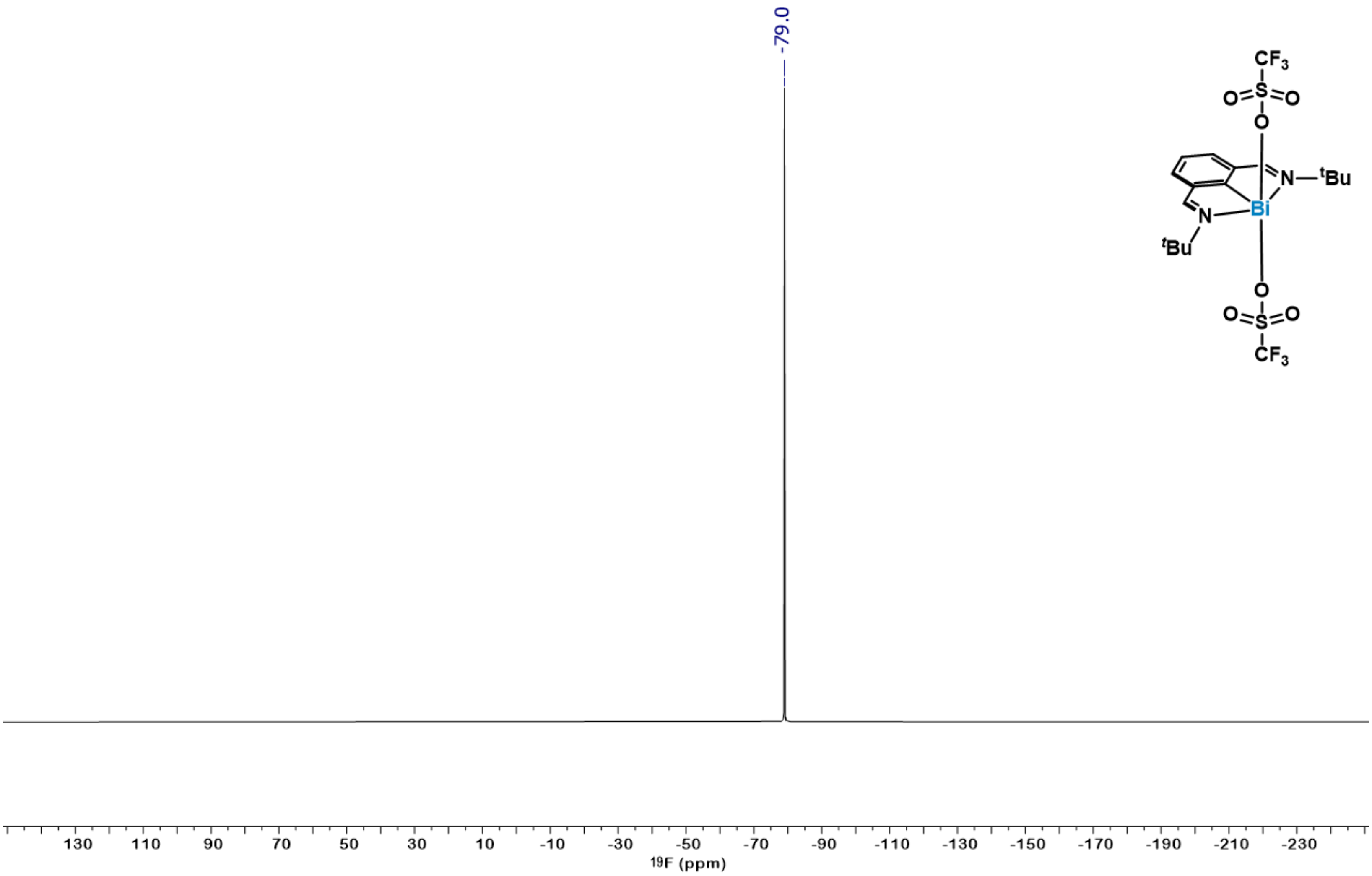

Figure S66: Stacked ^{1}H NMR spectra (MeCN-d$_3$) showing the aromatic region for **1** (300 MHz, 25°C), **3F** (300 MHz, 25°C) and an aliquot of the reaction mixture of the disproportionation of **2OTf** using 30 equiv. [NBu$_4$][BF$_4$] (600 MHz, -40°C).

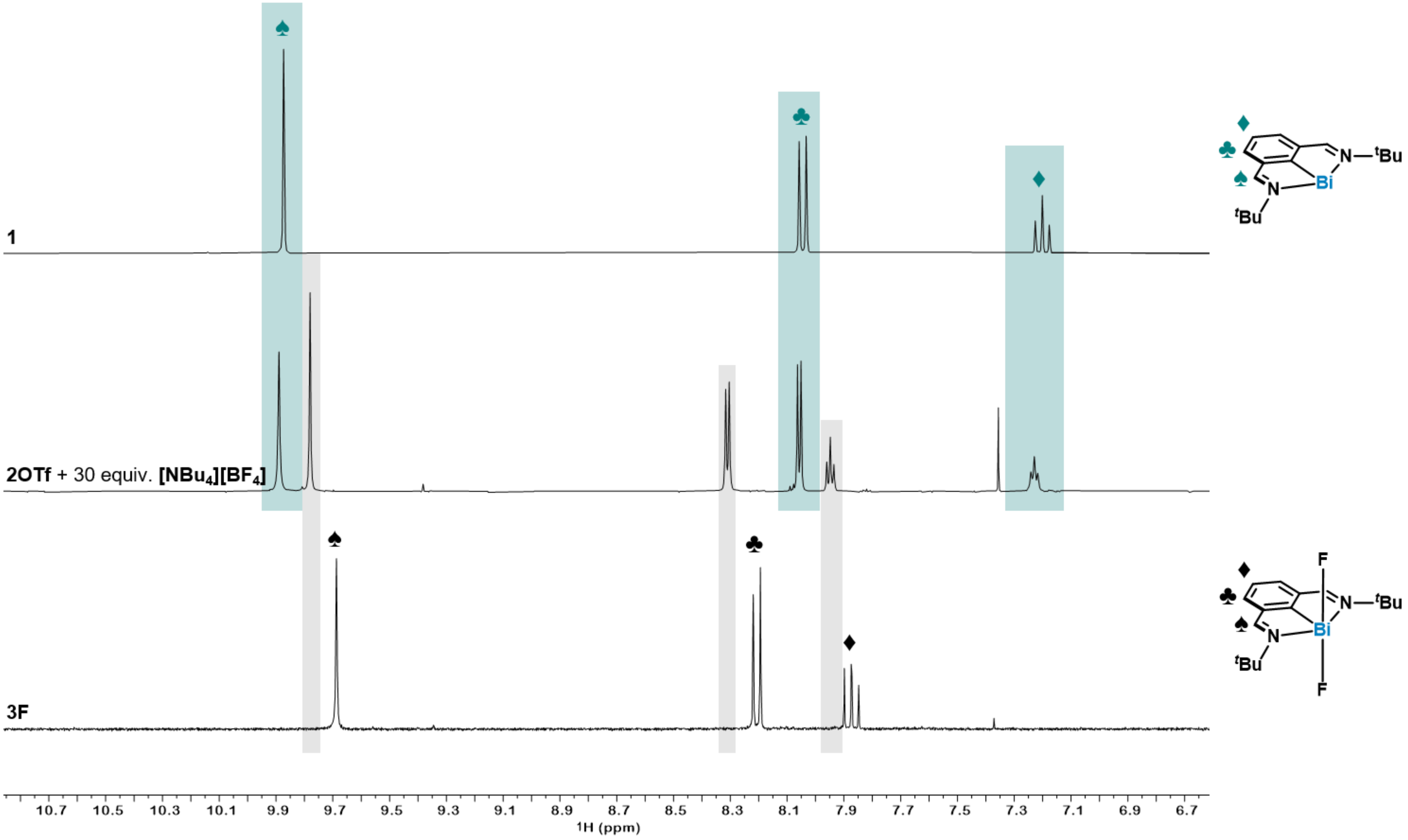

Figure S67: ^{19}F NMR spectrum (MeCN-d_3, 564.72 MHz, -40°C) of an aliquot of the reaction mixture of the disproportionation of **2** using 30 equiv. $[NBu_4][BF_4]$.

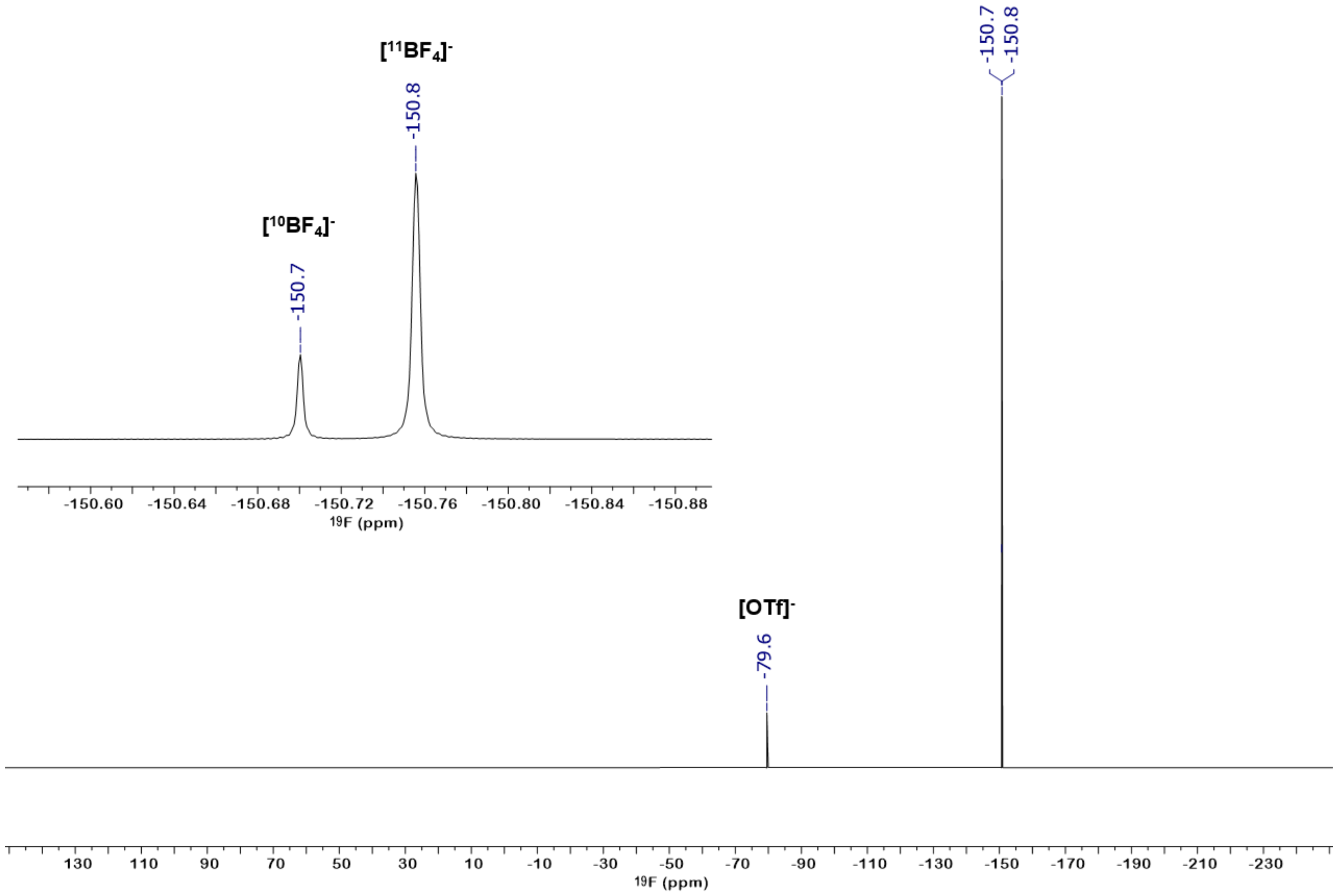

Figure S68: ^{11}B NMR spectrum (MeCN-d$_3$, 192.57 MHz, -40°C) of an aliquot of the reaction mixture of the disproportionation of **2** using 30 equiv. [NBu$_4$][BF$_4$].

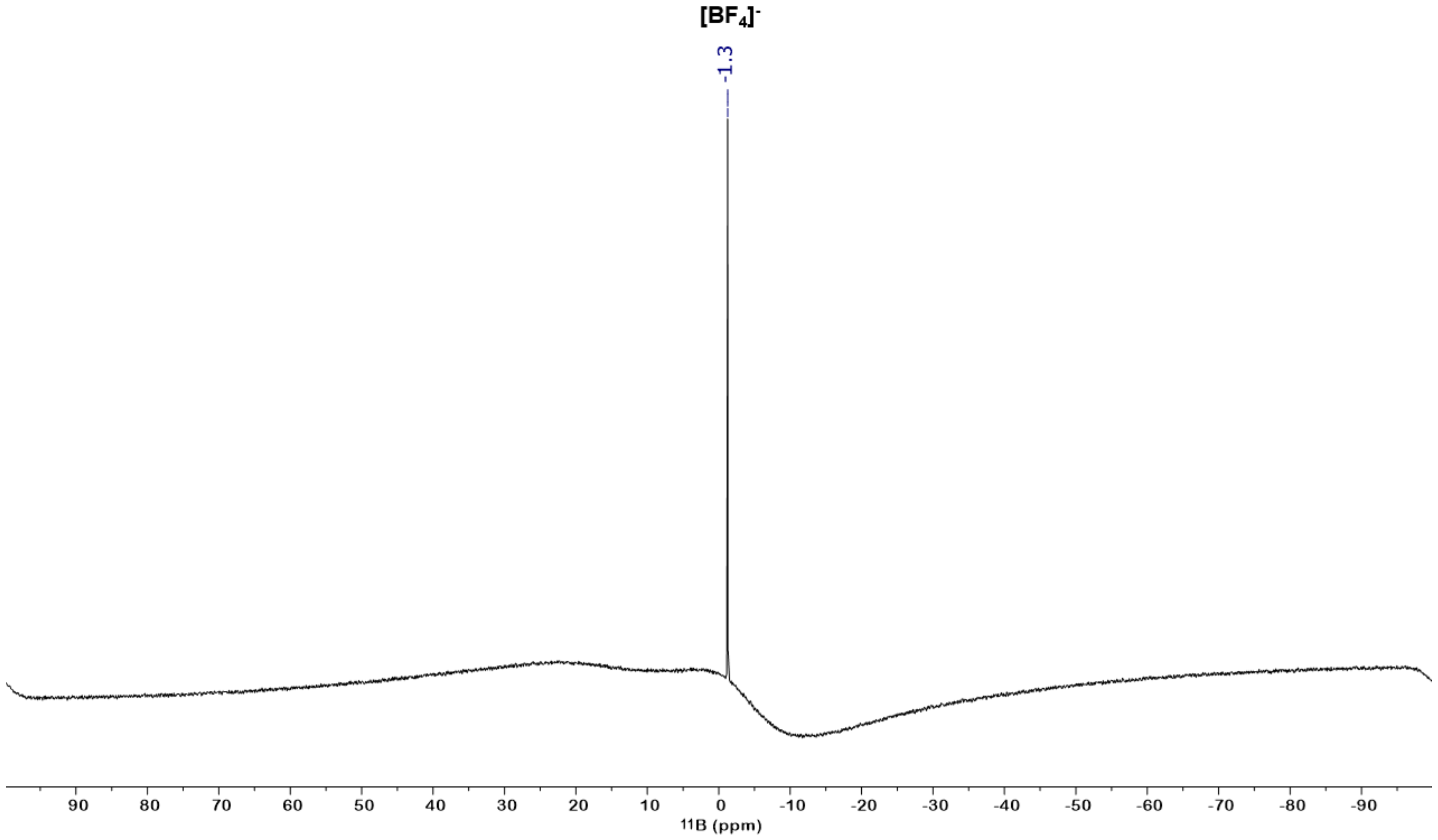

Figure S69: ^{1}H NMR spectrum (600.20 MHz, MeCN-d_3) of **2BF$_4$**.

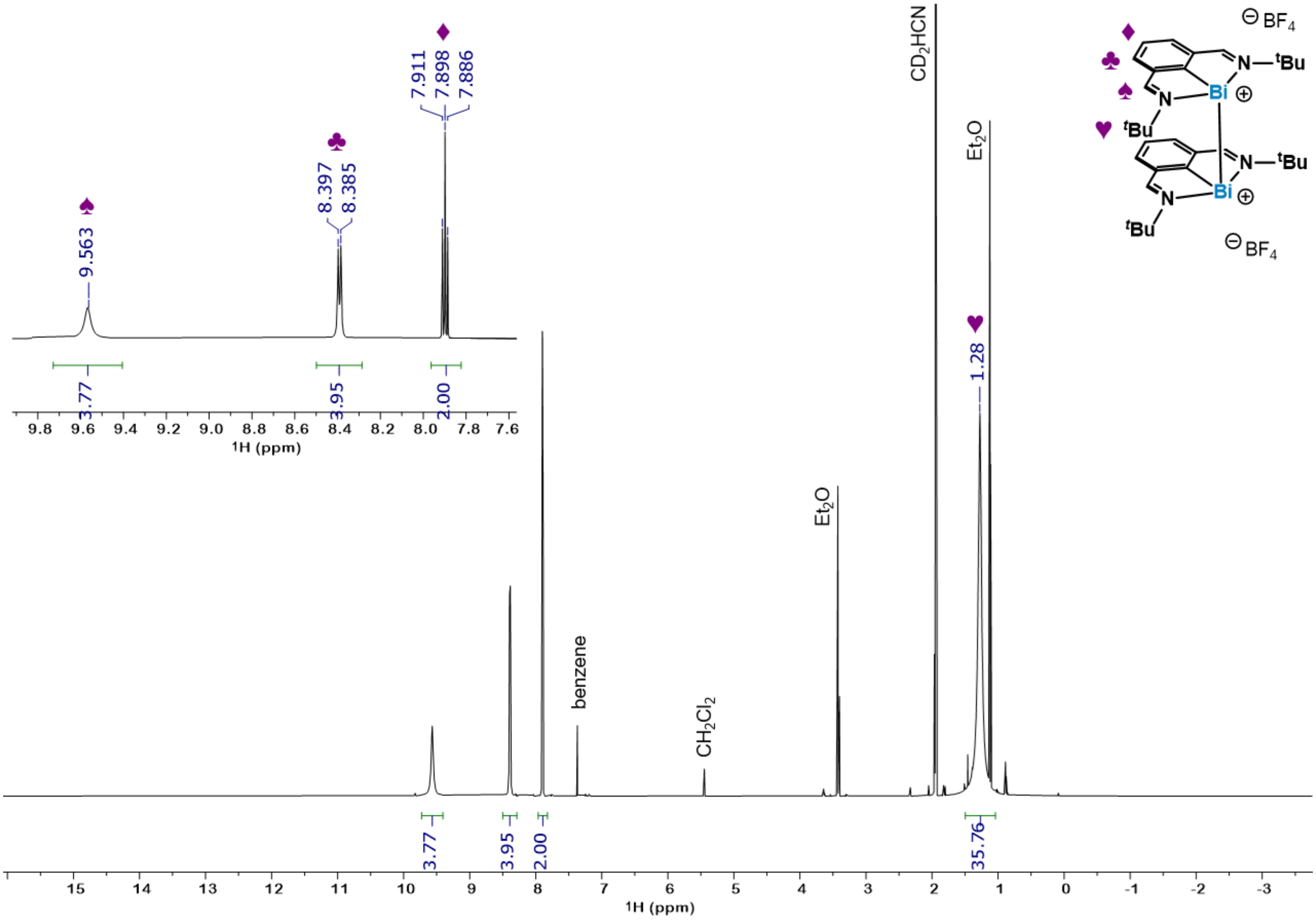

Figure S70: ${}^{11}B$ NMR spectrum (192.57 MHz, MeCN-d_3) of **2BF$_4$**.

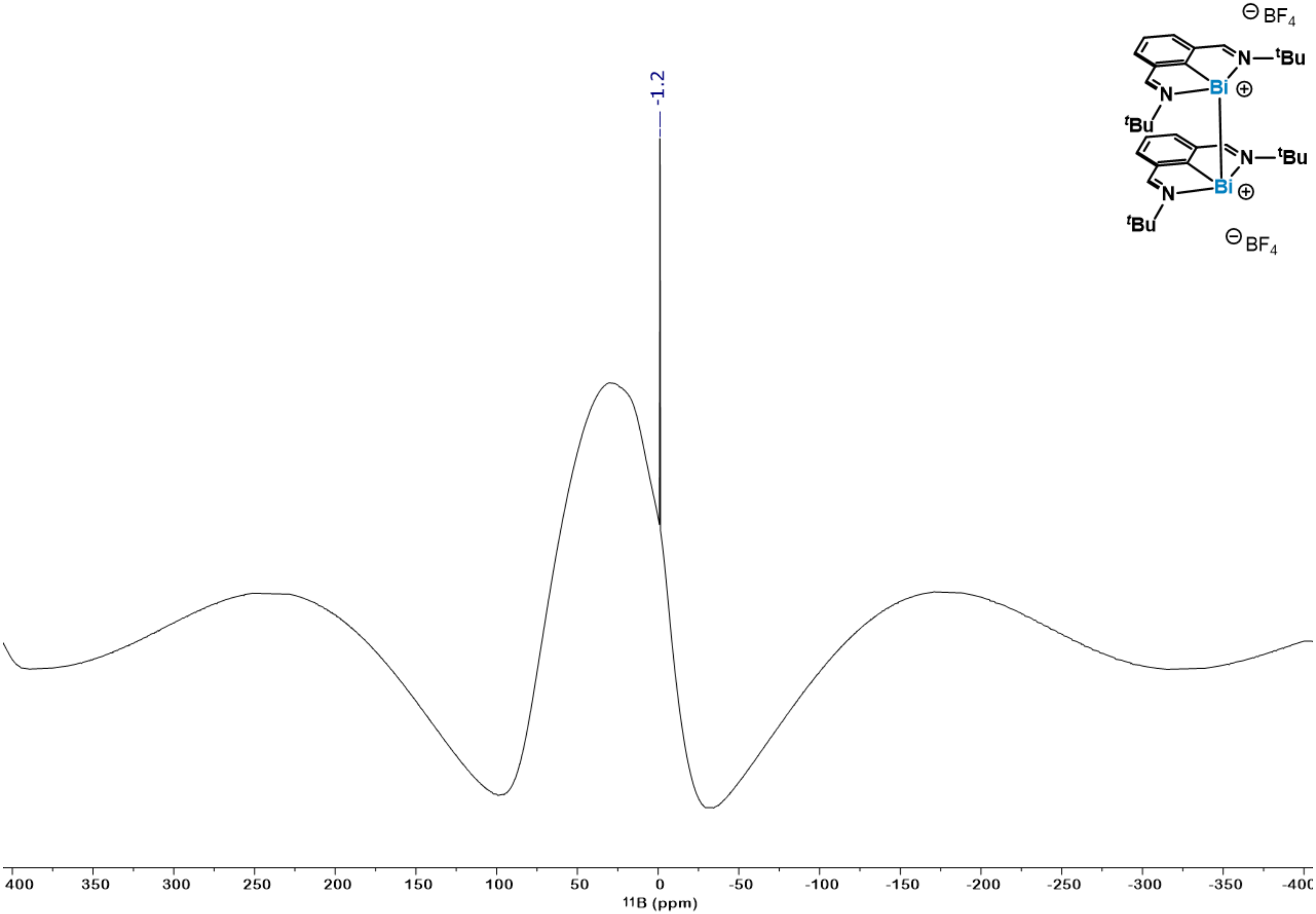

Figure S71: $^{13}C\{^1H\}$ NMR spectrum (150.94 MHz, MeCN-d$_3$) of **2BF$_4$**.

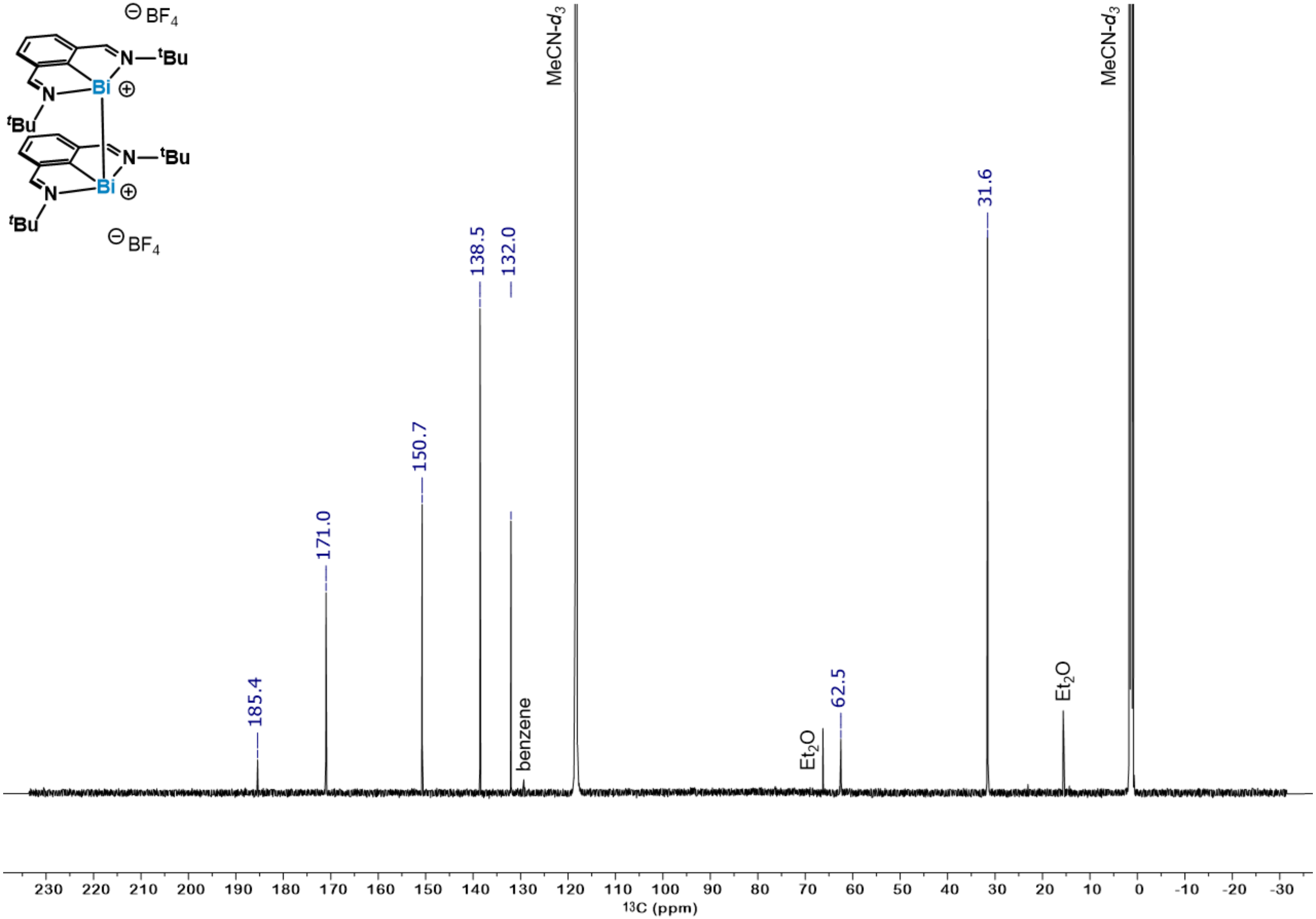

Figure S72: ^{19}F NMR spectrum (564.72 MHz, MeCN-d$_3$) of **2BF$_4$**.

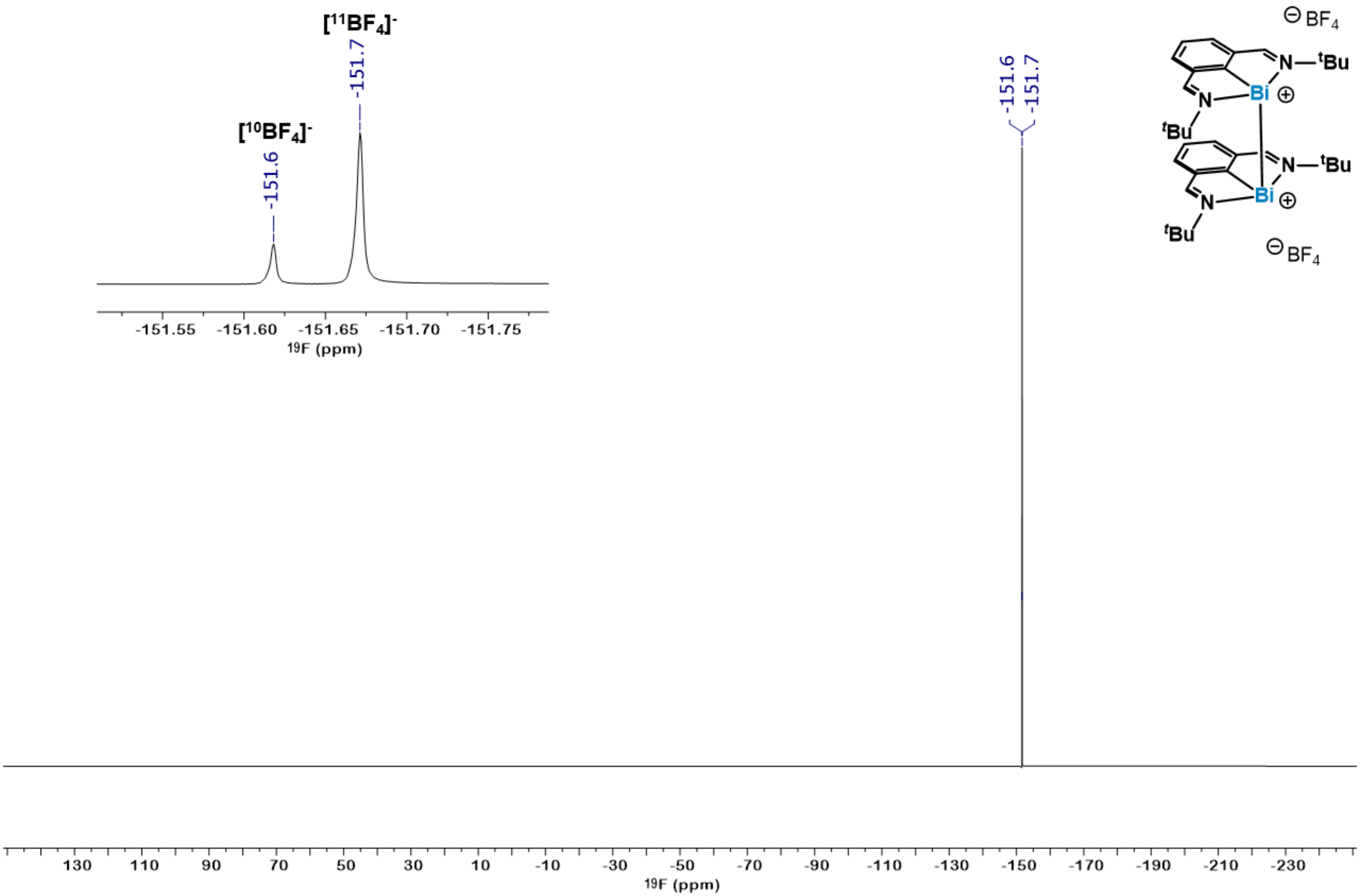

14. X-Ray Crystallography

14.1 Single crystal structure analysis of **3Br** acetonitrile solvate

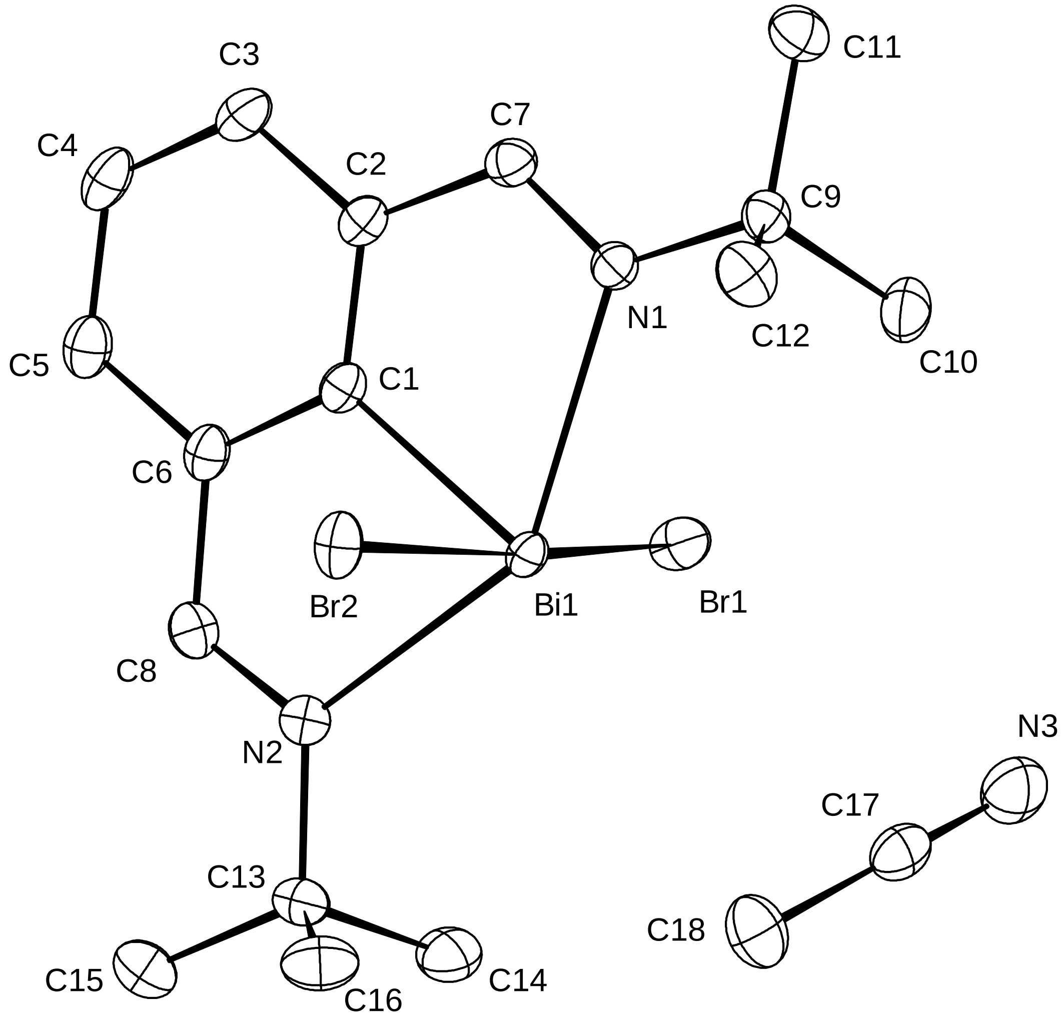

Figure S73: The molecular structure of **3Br acetonitrile solvate**. H atoms have been removed for clarity.

X-ray Crystal Structure Analysis of **3Br acetonitrile solvate**:

C_{20} H_{29} Bi Br_2 N_4, M_r = 694.27 g mol^{-1}, yellow plate, crystal size 0.209 x 0.127 x 0.04 mm^3, Monoclinic, space group $P2_1/n$ [14], a = 14.7592(4) Å, b = 9.0909(2) Å, c = 19.0499(6) Å, β= 103.3440(10)°, V = 2487.00(12) Å^3, T = 100(2) K, Z = 4, D_{calc} = 1.854 g·cm^3, λ = 0.71073 Å, $\mu(Mo\text{-}K_\alpha)$ = 10.314 mm^{-1}, Gaussian absorption correction ($T_{\min}$ = 0.35581, $T_{\max}$ = 0.71817), Bruker-AXS D8 Venture with Photon III detector and IµS Diamond microfocus Mo-anode X-ray source, 1.984 to 31.529°, 292412 measured reflections, 8292 independent reflections, 7271

reflections with $I > 2\sigma(I)$, $R_{int} = 0.0826$. The structure was solved by *SHELXT* and refined by full-matrix least-squares (*SHELXL*) against F^2 to $R_1 = 0.0154$ [$I > 2\sigma(I)$], $wR_2 = 0.0360$ [all data], 224 parameters and 0 restraints.

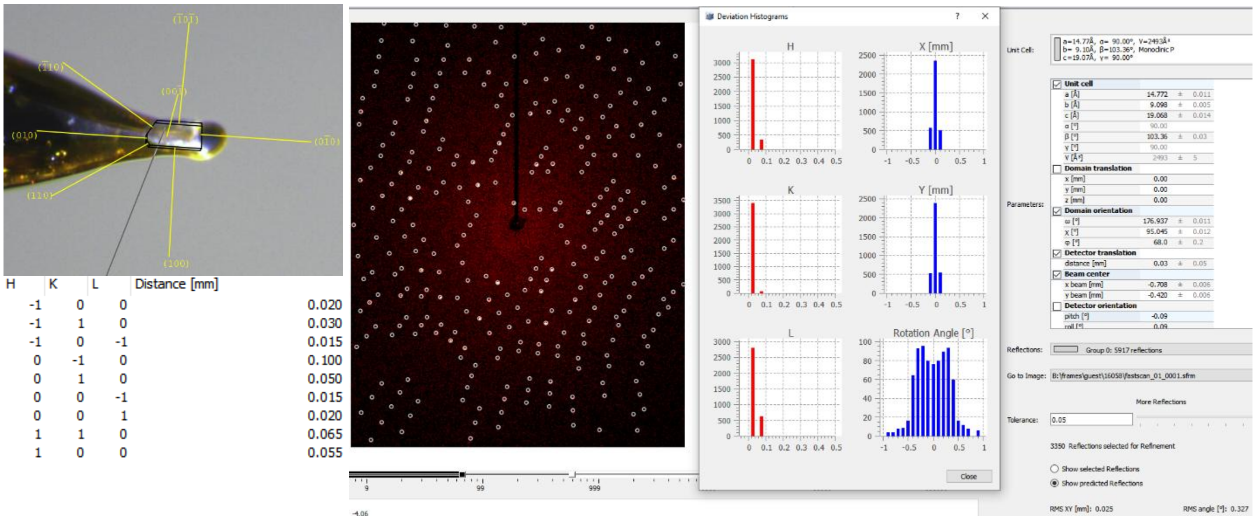

Figure S74: Crystal faces and unit cell determination/refinement of compound **3Br acetonitrile solvate**.

INTENSITY STATISTICS FOR DATASET

Resolution	#Data	#Theory	%Complete	Redundancy	Mean I	Mean I/s	Rmerge	Rsigma
Inf - 2.87	133	136	97.8	30.96	95.76	52.73	0.0352	0.0220
2.87 - 1.89	307	308	99.7	39.67	89.34	65.48	0.0418	0.0174
1.89 - 1.49	439	439	100.0	45.90	60.92	62.62	0.0462	0.0148
1.49 - 1.29	457	457	100.0	47.49	46.38	63.96	0.0502	0.0109
1.29 - 1.17	446	446	100.0	47.35	42.40	59.51	0.0516	0.0108
1.17 - 1.09	417	417	100.0	47.10	33.72	54.01	0.0610	0.0119
1.09 - 1.02	452	452	100.0	44.96	28.16	48.69	0.0711	0.0135
1.02 - 0.97	432	432	100.0	40.14	24.05	41.31	0.0794	0.0153
0.97 - 0.92	503	503	100.0	39.10	24.22	39.63	0.0865	0.0163
0.92 - 0.89	387	387	100.0	36.13	19.18	32.72	0.1026	0.0193
0.89 - 0.86	420	420	100.0	35.07	20.78	35.32	0.1013	0.0192
0.86 - 0.83	467	467	100.0	34.76	17.19	29.94	0.1142	0.0215
0.83 - 0.80	573	573	100.0	32.90	14.17	26.67	0.1360	0.0254
0.80 - 0.78	411	411	100.0	30.84	14.07	25.13	0.1345	0.0262
0.78 - 0.76	447	447	100.0	29.53	11.96	22.03	0.1504	0.0301
0.76 - 0.74	536	536	100.0	27.48	9.93	19.04	0.1753	0.0356
0.74 - 0.73	283	283	100.0	24.75	10.64	18.24	0.1820	0.0374
0.73 - 0.71	601	601	100.0	23.52	9.74	16.82	0.1924	0.0410
0.71 - 0.70	312	312	100.0	20.21	8.50	13.89	0.2115	0.0524
0.70 - 0.69	351	351	100.0	20.31	8.86	14.78	0.1934	0.0480
0.69 - 0.68	388	392	99.0	17.77	8.34	12.81	0.1879	0.0573
0.78 - 0.68	2918	2922	99.9	23.77	9.78	17.07	0.1792	0.0412
Inf - 0.68	8762	8770	99.9	34.47	25.45	35.33	0.0795	0.0193

Complete .cif-data of the compound are available under the CCDC number CCDC-**2490752**. The structure contained a disordered solute molecule, which could not properly refined.

A solvent mask was calculated and 102 electrons were found in a volume of 510 Å³ in 1 void per unit cell. This is consistent with the presence of 1[C_2H_3N] per asymmetric unit which account for 88 electrons per unit cell. The solvent mask (SQUEEZE routine in Olex2) was applied leading into a structure with a void volume of 475.74 Å³ which belongs to 19.1% of the unit cell volume (probe radius of 1.2 Å and an approx. grid spacing of 0.7 Å).

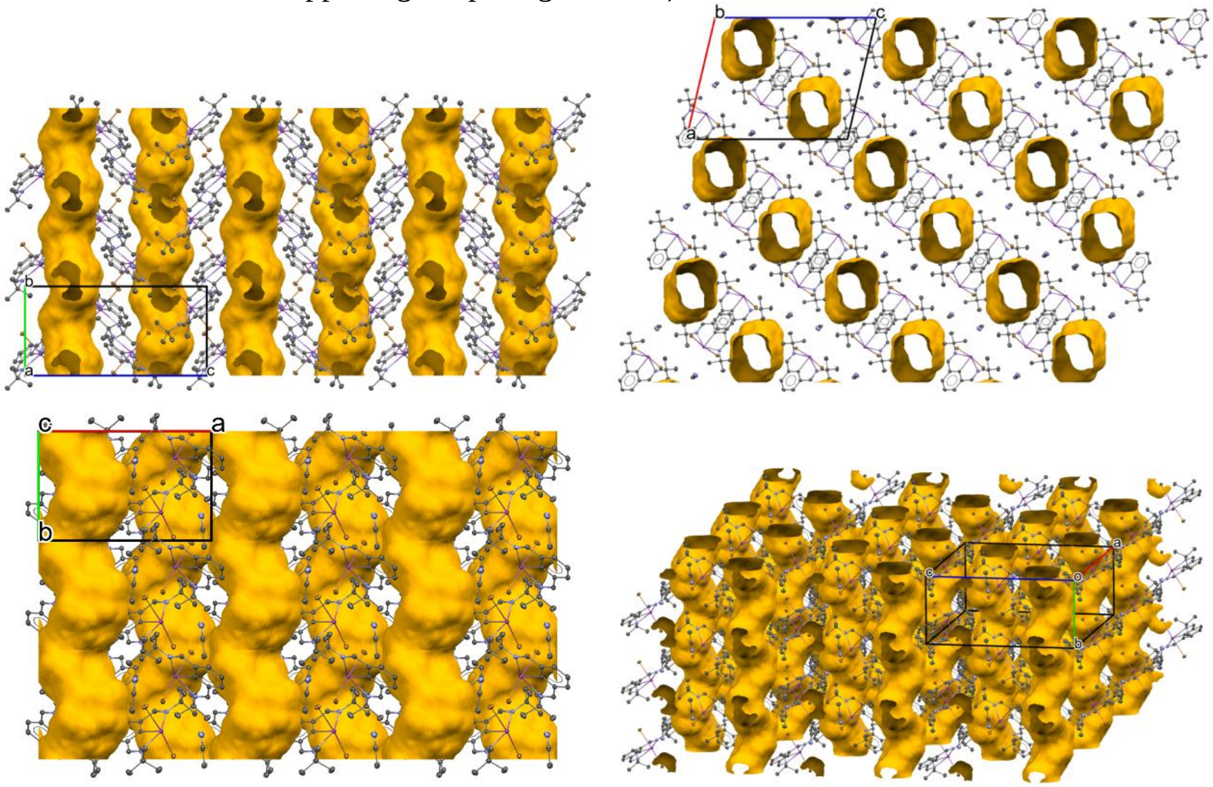

Figure S75: 3 x 3 x 3 packing motive of the unit cell and **s**olvent accessible voids viewed along crystallographic a- (top left), b- (top right), c-axis (bottom left) and in a random orientation (bottom right)

Table S4: Bond lengths [Å] and angles [°] of compound **3Br acetonitrile solvate**.

Identification code	16058	
Empirical formula	C_{20} H_{29} Bi Br_2 N_4	
Color	yellow	
Formula weight	694.27 g·mol^{-1}	
Temperature	100(2) K	
Wavelength	0.71073 Å	
Crystal system	Monoclinic	
Space group	$P2_1/n$, (no. 14)	
Unit cell dimensions	a = 14.7592(4) Å	α= 90°.
	b = 9.0909(2) Å	β= 103.3440(10)°.
	c = 19.0499(6) Å	γ = 90°.
Volume	2487.00(12) Å^3	
Z	4	
Density (calculated)	1.854 Mg·m^{-3}	
Absorption coefficient	10.314 mm^{-1}	
F(000)	1320 e	
Crystal size	0.209 x 0.127 x 0.04 mm^3	
θ range for data collection	1.984 to 31.529°.	
Index ranges	$-21 \leq h \leq 21$, $-13\leq k \leq 13$, $-28 \leq l \leq 28$	
Reflections collected	292412	
Independent reflections	8292 [R_{int} = 0.0826]	
Reflections with I>2σ(I)	7271	
Completeness to θ = 25.242°	100.0 %	
Absorption correction	Gaussian	
Max. and min. transmission	0.71817 and 0.35581	
Refinement method	Full-matrix least-squares on F^2	
Data / restraints / parameters	8292 / 0 / 224	
Goodness-of-fit on F^2	1.058	
Final R indices [I>2σ(I)]	R_1 = 0.0154	wR^2 = 0.0350
R indices (all data)	R_1 = 0.0203	wR^2 = 0.0360
Extinction coefficient	n/a	
Largest diff. peak and hole	0.881 and -0.926 e·Å^{-3}	

Table S5: Bond lengths [Å] and angles [°] of compound **3Br acetonitrile solvate**.

Bi(1)-Br(1)	2.82495(17)	Bi(1)-Br(2)	
2.83243(17)	Bi(1)-N(1)	2.5092(13)	Bi(1)-N(2)
2.4909(13)	Bi(1)-C(1)	2.1982(16)	N(1)-C(7)
1.276(2)	N(1)-C(9)	1.485(2)	N(2)-C(8)
1.278(2)	N(2)-C(13)	1.485(2)	C(1)-C(2)
1.380(2)	C(1)-C(6)	1.386(2)	C(2)-C(3)
1.402(2)	C(2)-C(7)	1.471(2)	C(3)-H(3)
0.9500	C(3)-C(4)	1.389(2)	C(4)-H(4)
0.9500	C(4)-C(5)	1.389(2)	C(5)-H(5)
0.9500	C(5)-C(6)	1.401(2)	C(6)-C(8)
1.463(2)	C(7)-H(7)	0.9500	C(8)-H(8)
0.9500	C(9)-C(10)	1.526(2)	C(9)-C(11)
1.523(2)	C(9)-C(12)	1.534(2)	C(10)-
H(10A)	0.9800	C(10)-H(10B)	0.9800
C(10)-H(10C)	0.9800	C(11)-H(11A)	0.9800
C(11)-H(11B)	0.9800	C(11)-H(11C)	0.9800
C(12)-H(12A)	0.9800	C(12)-H(12B)	0.9800
C(12)-H(12C)	0.9800	C(13)-C(14)	1.529(2)
C(13)-C(15)	1.521(2)	C(13)-C(16)	1.530(2)
C(14)-H(14A)	0.9800	C(14)-H(14B)	0.9800
C(14)-H(14C)	0.9800	C(15)-H(15A)	0.9800
C(15)-H(15B)	0.9800	C(15)-H(15C)	0.9800
C(16)-H(16A)	0.9800	C(16)-H(16B)	0.9800
C(16)-H(16C)	0.9800	N(3)-C(17)	1.142(2)
C(17)-C(18)	1.452(2)	C(18)-H(18A)	0.9800
C(18)-H(18B)	0.9800	C(18)-H(18C)	0.9800
Br(1)-Bi(1)-Br(2)	172.450(5)	N(1)-Bi(1)-Br(1)	90.03(3)
N(1)-Bi(1)-Br(2)	89.09(3)	N(2)-Bi(1)-Br(1)	88.63(3)
N(2)-Bi(1)-Br(2)	87.52(3)	N(2)-Bi(1)-N(1)	142.57(4)
C(1)-Bi(1)-Br(1)	87.24(4)	C(1)-Bi(1)-Br(2)	85.38(4)
C(1)-Bi(1)-N(1)	71.09(5)	C(1)-Bi(1)-N(2)	71.48(5)
C(7)-N(1)-Bi(1)	111.81(10)	C(7)-N(1)-C(9)	122.91(13)

C(9)-N(1)-Bi(1)	125.28(10)	C(8)-N(2)-Bi(1)	111.84(10)
C(8)-N(2)-C(13)	123.05(14)	C(13)-N(2)-Bi(1)	125.09(10)
C(2)-C(1)-Bi(1)	119.63(11)	C(2)-C(1)-C(6)	121.37(15)
C(6)-C(1)-Bi(1)	118.99(11)	C(1)-C(2)-C(3)	119.46(15)
C(1)-C(2)-C(7)	117.71(14)	C(3)-C(2)-C(7)	122.83(14)
C(2)-C(3)-H(3)	120.2	C(4)-C(3)-C(2)	119.51(15)
C(4)-C(3)-H(3)	120.2	C(3)-C(4)-H(4)	119.6
C(5)-C(4)-C(3)	120.76(15)	C(5)-C(4)-H(4)	119.6
C(4)-C(5)-H(5)	120.2	C(4)-C(5)-C(6)	119.58(15)
C(6)-C(5)-H(5)	120.2	C(1)-C(6)-C(5)	119.31(15)
C(1)-C(6)-C(8)	117.76(14)	C(5)-C(6)-C(8)	122.92(15)
N(1)-C(7)-C(2)	119.75(14)	N(1)-C(7)-H(7)	120.1
C(2)-C(7)-H(7)	120.1	N(2)-C(8)-C(6)	119.93(14)
N(2)-C(8)-H(8)	120.0	C(6)-C(8)-H(8)	120.0
N(1)-C(9)-C(10)	106.64(13)	N(1)-C(9)-C(11)	113.55(13)
N(1)-C(9)-C(12)	106.30(12)	C(10)-C(9)-C(12)	109.48(14)
C(11)-C(9)-C(10)	110.26(13)	C(11)-C(9)-C(12)	110.44(14)
C(9)-C(10)-H(10A)	109.5	C(9)-C(10)-H(10B)	109.5
C(9)-C(10)-H(10C)	109.5	H(10A)-C(10)-H(10B)	109.5
H(10A)-C(10)-H(10C)	109.5	H(10B)-C(10)-H(10C)	109.5
C(9)-C(11)-H(11A)	109.5	C(9)-C(11)-H(11B)	109.5
C(9)-C(11)-H(11C)	109.5	H(11A)-C(11)-H(11B)	109.5
H(11A)-C(11)-H(11C)	109.5	H(11B)-C(11)-H(11C)	109.5
C(9)-C(12)-H(12A)	109.5	C(9)-C(12)-H(12B)	109.5
C(9)-C(12)-H(12C)	109.5	H(12A)-C(12)-H(12B)	109.5
H(12A)-C(12)-H(12C)	109.5	H(12B)-C(12)-H(12C)	109.5
N(2)-C(13)-C(14)	106.57(13)	N(2)-C(13)-C(15)	113.23(14)
N(2)-C(13)-C(16)	106.97(14)	C(14)-C(13)-C(16)	109.62(15)
C(15)-C(13)-C(14)	110.30(15)	C(15)-C(13)-C(16)	110.02(14)
C(13)-C(14)-H(14A)	109.5	C(13)-C(14)-H(14B)	109.5
C(13)-C(14)-H(14C)	109.5	H(14A)-C(14)-H(14B)	109.5
H(14A)-C(14)-H(14C)	109.5	H(14B)-C(14)-H(14C)	109.5
C(13)-C(15)-H(15A)	109.5	C(13)-C(15)-H(15B)	109.5
C(13)-C(15)-H(15C)	109.5	H(15A)-C(15)-H(15B)	109.5
H(15A)-C(15)-H(15C)	109.5	H(15B)-C(15)-H(15C)	109.5
C(13)-C(16)-H(16A)	109.5	C(13)-C(16)-H(16B)	109.5

C(13)-C(16)-H(16C)	109.5	H(16A)-C(16)-H(16B)	109.5
H(16A)-C(16)-H(16C)	109.5	H(16B)-C(16)-H(16C)	109.5
N(3)-C(17)-C(18)	179.6(2)	C(17)-C(18)-H(18A)	109.5
C(17)-C(18)-H(18B)	109.5	C(17)-C(18)-H(18C)	109.5
H(18A)-C(18)-H(18B)	109.5	H(18A)-C(18)-H(18C)	109.5
H(18B)-C(18)-H(18C)	109.5		

14.2 Single crystal structure analysis of **3OPh** diethyl ether solvate

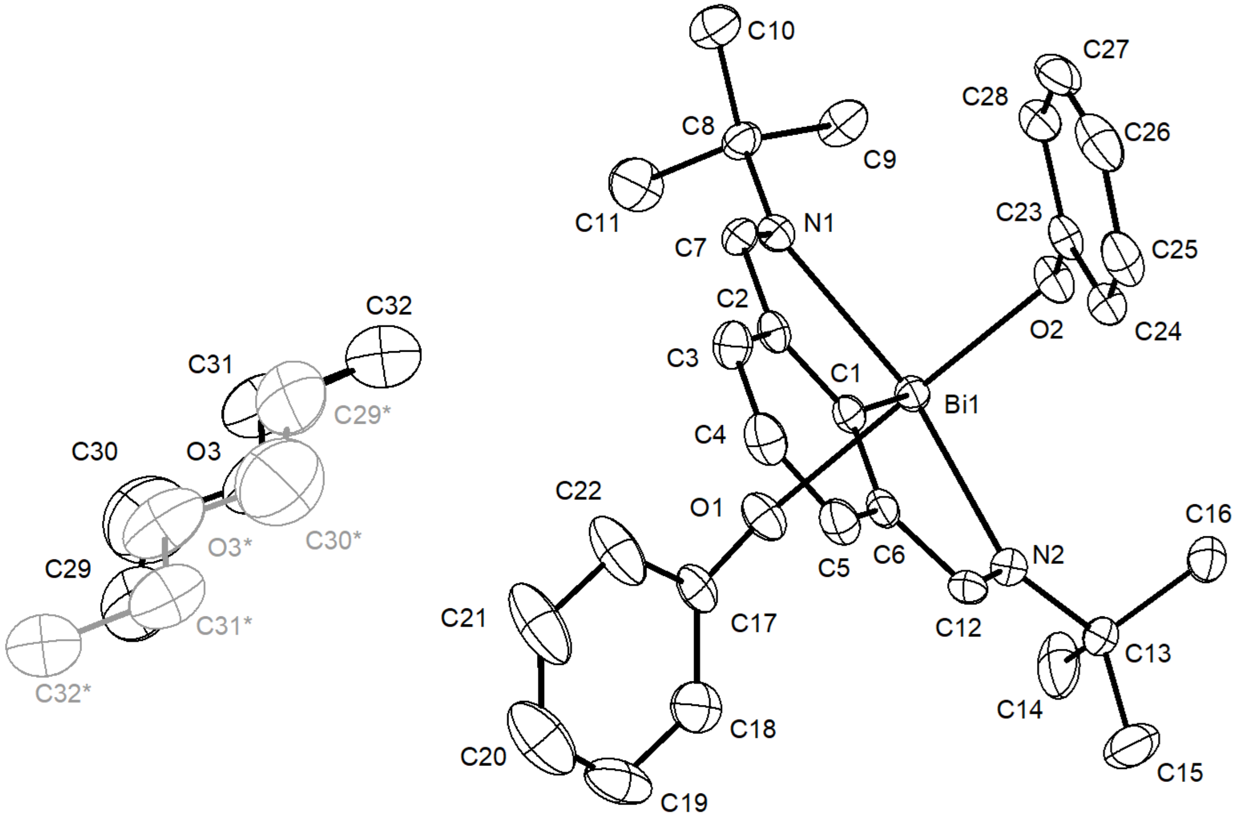

Figure S76: The molecular structure of **3OPh diethyl ether solvate**. H atoms have been removed for clarity.

X-ray Crystal Structure Analysis of **3OPh diethyl ether solvate**:

$C_{60}H_{76}Bi_2N_4O_5$, M_r = 1351.20 g mol^{-1}, yellow prism, crystal size 0.10 x 0.081 x 0.051 mm^3, Orthorhombic, space group *Pbca* [61], a = 16.1056(14) Å, b = 12.6037(11) Å, c = 28.202(2) Å, V = 5724.7(8) Å^3, T = 100(2) K, Z = 4, D_{calc} = 1.568 g·cm^3, λ = 0.71073 Å, $\mu(Mo\text{-}K_\alpha)$ = 6.189 mm^{-1}, Gaussian absorption correction (T_{min} = 0.63610, T_{max} = 0.84582), Bruker-AXS Kappa Mach3 with APEX-II detector and IµS microfocus Mo-anode X-ray source, 1.444 < θ < 32.573°, 196089 measured reflections, 10428 independent reflections, 5266 reflections with $I > 2\sigma(I)$, R_{int} = 0.1420. The structure was solved by *SHELXT* and refined by full-matrix least-squares (*SHELXL*) against F^2 to R_1 = 0.0395 [$I > 2\sigma(I)$], wR_2 = 0.0797 [all data], 351 parameters and 84 restraints.

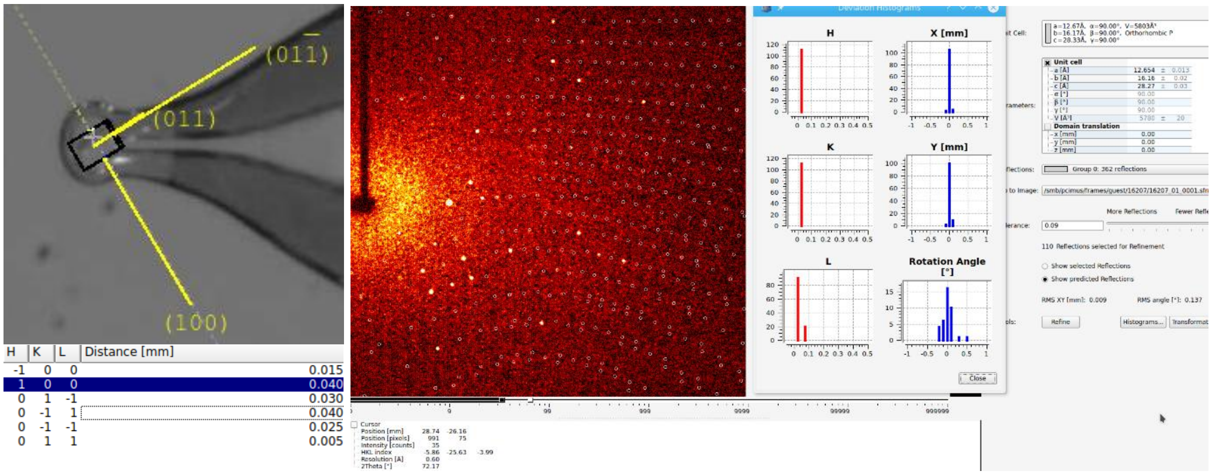

Figure S77: Crystal faces and unit cell determination/refinement of compound **3OPh diethyl ether solvate**.

INTENSITY STATISTICS FOR DATASET

Resolution	#Data	#Theory	%Complete	Redundancy	Mean I	Mean I/s	Rmerge	Rsigma
Inf - 2.75	201	202	99.5	27.42	81.58	40.58	0.0395	0.0133
2.75 - 1.77	472	472	100.0	32.45	60.86	35.78	0.0512	0.0140
1.77 - 1.39	658	658	100.0	33.75	41.21	28.03	0.0696	0.0172
1.39 - 1.20	691	691	100.0	33.37	31.99	23.92	0.0896	0.0214
1.20 - 1.09	637	637	100.0	30.26	25.55	19.37	0.1136	0.0283
1.09 - 1.01	663	663	100.0	22.16	20.65	13.64	0.1402	0.0422
1.01 - 0.94	754	754	100.0	18.25	17.66	10.83	0.1599	0.0554
0.94 - 0.89	682	682	100.0	15.69	13.93	8.24	0.1947	0.0761
0.89 - 0.85	687	687	100.0	14.68	13.26	7.40	0.2117	0.0860
0.85 - 0.82	614	614	100.0	13.99	9.87	5.84	0.2667	0.1178
0.82 - 0.79	668	668	100.0	13.66	10.24	5.87	0.2621	0.1197
0.79 - 0.76	812	812	100.0	12.87	8.58	4.73	0.3206	0.1529
0.76 - 0.74	602	602	100.0	12.48	7.30	4.03	0.3716	0.1853
0.74 - 0.72	688	688	100.0	11.96	6.86	3.71	0.3962	0.2078
0.72 - 0.70	736	736	100.0	11.73	5.40	2.97	0.4728	0.2702
0.70 - 0.69	410	410	100.0	11.29	5.91	3.15	0.4496	0.2618
0.69 - 0.67	904	904	100.0	11.01	4.72	2.46	0.5282	0.3373
0.67 - 0.66	481	481	100.0	10.71	4.07	2.11	0.6022	0.4070
0.66 - 0.64	1068	1068	100.0	10.29	3.65	1.87	0.6296	0.4770
0.64 - 0.63	807	848	95.2	8.63	3.25	1.43	0.6981	0.6549
0.73 - 0.63	4772	4813	99.1	10.60	4.56	2.36	0.5397	0.3700
Inf - 0.63	13235	13277	99.7	16.96	15.44	9.55	0.1329	0.0820

Complete .cif-data of the compound are available under the CCDC number CCDC-**2490749**.

The structure contains a disorderd diethyl ether solute molecule. The 'Ether' fragment in the DSR tool (implemented in Olex2) was used to describe the diethyl ether.[33,34]

Table S6: Crystal data and structure refinement of compound **3OPh diethyl ether solvate**.

Identification code	16207	
Empirical formula	$C_{60} H_{76} Bi_2 N_4 O_5$	
Color	yellow	
Formula weight	1351.20 g·mol^{-1}	
Temperature	100(2) K	
Wavelength	0.71073 Å	
Crystal system	Orthorhombic	
Space group	*Pbca*, (no. 61)	
Unit cell dimensions	a = 16.1056(14) Å	α= 90°.
	b = 12.6037(11) Å	β= 90°.
	c = 28.202(2) Å	γ = 90°.
Volume	5724.7(8) Å^3	
Z	4	
Density (calculated)	1.568 Mg·m^{-3}	
Absorption coefficient	6.189 mm^{-1}	
F(000)	2680 e	
Crystal size	0.10 x 0.081 x 0.051 mm^3	
θ range for data collection	1.444 to 32.573°.	
Index ranges	$-24 \le h \le 24$, $-19 \le k \le 19$, $-42 \le l \le 42$	
Reflections collected	196089	
Independent reflections	10428 [R_{int} = 0.1420]	
Reflections with I>2σ(I)	5266	
Completeness to θ = 25.242°	100.0 %	
Absorption correction	Gaussian	
Max. and min. transmission	0.84582 and 0.63610	
Refinement method	Full-matrix least-squares on F^2	
Data / restraints / parameters	10428 / 84 / 351	
Goodness-of-fit on F^2	1.007	
Final R indices [I>2σ(I)]	R_1 = 0.0395	wR^2 = 0.0620
R indices (all data)	R_1 = 0.1100	wR^2 = 0.0797
Extinction coefficient	n/a	
Largest diff. peak and hole	1.092 and -1.586 e·Å^{-3}	

Table S7: Bond lengths [Å] and angles [°] of compound **3OPh diethyl ether solvate**.

Bi(1)-O(1)	2.318(3)	Bi(1)-O(2)	2.243(3)
Bi(1)-N(1)	2.487(4)	Bi(1)-N(2)	2.538(4)
Bi(1)-C(1)	2.193(5)	O(1)-C(17)	1.310(6)
O(2)-C(23)	1.329(6)	N(1)-C(7)	1.270(6)
N(1)-C(8)	1.490(6)	N(2)-C(12)	1.262(7)
N(2)-C(13)	1.481(6)	C(1)-C(2)	1.385(6)
C(1)-C(6)	1.392(6)	C(2)-C(3)	1.394(7)
C(2)-C(7)	1.460(6)	C(3)-H(3)	0.9500
C(3)-C(4)	1.384(7)	C(4)-H(4)	0.9500
C(4)-C(5)	1.392(8)	C(5)-H(5)	0.9500
C(5)-C(6)	1.387(7)	C(6)-C(12)	1.468(7)
C(7)-H(7)	0.9500	C(8)-C(9)	1.527(7)
C(8)-C(10)	1.529(7)	C(8)-C(11)	1.531(7)
C(9)-H(9A)	0.9800	C(9)-H(9B)	0.9800
C(9)-H(9C)	0.9800	C(10)-H(10A)	0.9800
C(10)-H(10B)	0.9800	C(10)-H(10C)	0.9800
C(11)-H(11A)	0.9800	C(11)-H(11B)	0.9800
C(11)-H(11C)	0.9800	C(12)-H(12)	0.9500
C(13)-C(14)	1.526(9)	C(13)-C(15)	1.524(8)
C(13)-C(16)	1.520(7)	C(14)-H(14A)	0.9800
C(14)-H(14B)	0.9800	C(14)-H(14C)	0.9800
C(15)-H(15A)	0.9800	C(15)-H(15B)	0.9800
C(15)-H(15C)	0.9800	C(16)-H(16A)	0.9800
C(16)-H(16B)	0.9800	C(16)-H(16C)	0.9800
C(17)-C(18)	1.403(8)	C(17)-C(22)	1.392(8)
C(18)-H(18)	0.9500	C(18)-C(19)	1.414(9)
C(19)-H(19)	0.9500	C(19)-C(20)	1.361(10)
C(20)-H(20)	0.9500	C(20)-C(21)	1.352(10)
C(21)-H(21)	0.9500	C(21)-C(22)	1.385(9)
C(22)-H(22)	0.9500	C(23)-C(24)	1.402(7)
C(23)-C(28)	1.411(7)	C(24)-H(24)	0.9500
C(24)-C(25)	1.368(7)	C(25)-H(25)	0.9500
C(25)-C(26)	1.392(8)	C(26)-H(26)	0.9500
C(26)-C(27)	1.381(8)	C(27)-H(27)	0.9500

C(27)-C(28)	1.382(8)	C(28)-H(28)	0.9500
O(3)-C(30)	1.4307(10)	O(3)-C(31)	1.4299(10)
C(29)-H(29A)	0.9800	C(29)-H(29B)	0.9800
C(29)-H(29C)	0.9800	C(29)-C(30)	1.526(9)
C(30)-H(30A)	0.9900	C(30)-H(30B)	0.9900
C(31)-H(31A)	0.9900	C(31)-H(31B)	0.9900
C(31)-C(32)	1.535(8)	C(32)-H(32A)	0.9800
C(32)-H(32B)	0.9800	C(32)-H(32C)	0.9800

O(1)-Bi(1)-N(1)	92.44(13)	O(1)-Bi(1)-N(2)	87.27(13)
O(2)-Bi(1)-O(1)	175.46(12)	O(2)-Bi(1)-N(1)	87.55(13)
O(2)-Bi(1)-N(2)	89.93(14)	N(1)-Bi(1)-N(2)	141.85(14)
C(1)-Bi(1)-O(1)	86.55(14)	C(1)-Bi(1)-O(2)	89.14(14)
C(1)-Bi(1)-N(1)	71.39(15)	C(1)-Bi(1)-N(2)	70.51(16)
C(17)-O(1)-Bi(1)	134.3(3)	C(23)-O(2)-Bi(1)	127.3(3)
C(7)-N(1)-Bi(1)	111.8(3)	C(7)-N(1)-C(8)	123.0(4)
C(8)-N(1)-Bi(1)	125.2(3)	C(12)-N(2)-Bi(1)	111.6(3)
C(12)-N(2)-C(13)	124.2(5)	C(13)-N(2)-Bi(1)	124.2(3)
C(2)-C(1)-Bi(1)	119.0(3)	C(2)-C(1)-C(6)	120.9(4)
C(6)-C(1)-Bi(1)	120.0(3)	C(1)-C(2)-C(3)	119.3(4)
C(1)-C(2)-C(7)	117.5(4)	C(3)-C(2)-C(7)	123.2(4)
C(2)-C(3)-H(3)	120.0	C(4)-C(3)-C(2)	120.1(5)
C(4)-C(3)-H(3)	120.0	C(3)-C(4)-H(4)	119.8
C(3)-C(4)-C(5)	120.4(5)	C(5)-C(4)-H(4)	119.8
C(4)-C(5)-H(5)	120.1	C(6)-C(5)-C(4)	119.9(5)
C(6)-C(5)-H(5)	120.1	C(1)-C(6)-C(12)	117.4(4)
C(5)-C(6)-C(1)	119.5(5)	C(5)-C(6)-C(12)	123.1(5)
N(1)-C(7)-C(2)	120.1(4)	N(1)-C(7)-H(7)	119.9
C(2)-C(7)-H(7)	119.9	N(1)-C(8)-C(9)	107.1(4)
N(1)-C(8)-C(10)	111.9(4)	N(1)-C(8)-C(11)	107.2(4)
C(9)-C(8)-C(10)	109.5(4)	C(9)-C(8)-C(11)	110.3(4)
C(10)-C(8)-C(11)	110.7(4)	C(8)-C(9)-H(9A)	109.5
C(8)-C(9)-H(9B)	109.5	C(8)-C(9)-H(9C)	109.5
H(9A)-C(9)-H(9B)	109.5	H(9A)-C(9)-H(9C)	109.5
H(9B)-C(9)-H(9C)	109.5	C(8)-C(10)-H(10A)	109.5
C(8)-C(10)-H(10B)	109.5	C(8)-C(10)-H(10C)	109.5

H(10A)-C(10)-H(10B)	109.5	H(10A)-C(10)-H(10C)	109.5
H(10B)-C(10)-H(10C)	109.5	C(8)-C(11)-H(11A)	109.5
C(8)-C(11)-H(11B)	109.5	C(8)-C(11)-H(11C)	109.5
H(11A)-C(11)-H(11B)	109.5	H(11A)-C(11)-H(11C)	109.5
H(11B)-C(11)-H(11C)	109.5	N(2)-C(12)-C(6)	120.1(4)
N(2)-C(12)-H(12)	120.0	C(6)-C(12)-H(12)	120.0
N(2)-C(13)-C(14)	106.9(4)	N(2)-C(13)-C(15)	113.7(5)
N(2)-C(13)-C(16)	106.4(4)	C(15)-C(13)-C(14)	110.8(6)
C(16)-C(13)-C(14)	110.1(5)	C(16)-C(13)-C(15)	108.8(5)
C(13)-C(14)-H(14A)	109.5	C(13)-C(14)-H(14B)	109.5
C(13)-C(14)-H(14C)	109.5	H(14A)-C(14)-H(14B)	109.5
H(14A)-C(14)-H(14C)	109.5	H(14B)-C(14)-H(14C)	109.5
C(13)-C(15)-H(15A)	109.5	C(13)-C(15)-H(15B)	109.5
C(13)-C(15)-H(15C)	109.5	H(15A)-C(15)-H(15B)	109.5
H(15A)-C(15)-H(15C)	109.5	H(15B)-C(15)-H(15C)	109.5
C(13)-C(16)-H(16A)	109.5	C(13)-C(16)-H(16B)	109.5
C(13)-C(16)-H(16C)	109.5	H(16A)-C(16)-H(16B)	109.5
H(16A)-C(16)-H(16C)	109.5	H(16B)-C(16)-H(16C)	109.5
O(1)-C(17)-C(18)	121.8(5)	O(1)-C(17)-C(22)	122.0(5)
C(22)-C(17)-C(18)	116.1(5)	C(17)-C(18)-H(18)	119.9
C(17)-C(18)-C(19)	120.2(6)	C(19)-C(18)-H(18)	119.9
C(18)-C(19)-H(19)	119.7	C(20)-C(19)-C(18)	120.5(6)
C(20)-C(19)-H(19)	119.7	C(19)-C(20)-H(20)	119.7
C(21)-C(20)-C(19)	120.6(6)	C(21)-C(20)-H(20)	119.7
C(20)-C(21)-H(21)	120.3	C(20)-C(21)-C(22)	119.4(7)
C(22)-C(21)-H(21)	120.3	C(17)-C(22)-H(22)	118.4
C(21)-C(22)-C(17)	123.1(7)	C(21)-C(22)-H(22)	118.4
O(2)-C(23)-C(24)	122.5(5)	O(2)-C(23)-C(28)	121.1(4)
C(24)-C(23)-C(28)	116.3(5)	C(23)-C(24)-H(24)	119.1
C(25)-C(24)-C(23)	121.9(5)	C(25)-C(24)-H(24)	119.1
C(24)-C(25)-H(25)	119.5	C(24)-C(25)-C(26)	121.0(5)
C(26)-C(25)-H(25)	119.5	C(25)-C(26)-H(26)	120.8
C(27)-C(26)-C(25)	118.4(5)	C(27)-C(26)-H(26)	120.8
C(26)-C(27)-H(27)	119.5	C(26)-C(27)-C(28)	120.9(5)
C(28)-C(27)-H(27)	119.5	C(23)-C(28)-H(28)	119.3
C(27)-C(28)-C(23)	121.4(5)	C(27)-C(28)-H(28)	119.3

C(31)-O(3)-C(30)	95.1(10)	H(29A)-C(29)-H(29B)	109.5
H(29A)-C(29)-H(29C)	109.5	H(29B)-C(29)-H(29C)	109.5
C(30)-C(29)-H(29A)	109.5	C(30)-C(29)-H(29B)	109.5
C(30)-C(29)-H(29C)	109.5	O(3)-C(30)-C(29)	102.3(7)
O(3)-C(30)-H(30A)	111.3	O(3)-C(30)-H(30B)	111.3
C(29)-C(30)-H(30A)	111.3	C(29)-C(30)-H(30B)	111.3
H(30A)-C(30)-H(30B)	109.2	O(3)-C(31)-H(31A)	111.7
O(3)-C(31)-H(31B)	111.7	O(3)-C(31)-C(32)	100.2(6)
H(31A)-C(31)-H(31B)	109.5	C(32)-C(31)-H(31A)	111.7
C(32)-C(31)-H(31B)	111.7	C(31)-C(32)-H(32A)	109.5
C(31)-C(32)-H(32B)	109.5	C(31)-C(32)-H(32C)	109.5
H(32A)-C(32)-H(32B)	109.5	H(32A)-C(32)-H(32C)	109.5
H(32B)-C(32)-H(32C)	109.5		

14.3 Single crystal structure analysis of **3SPh**

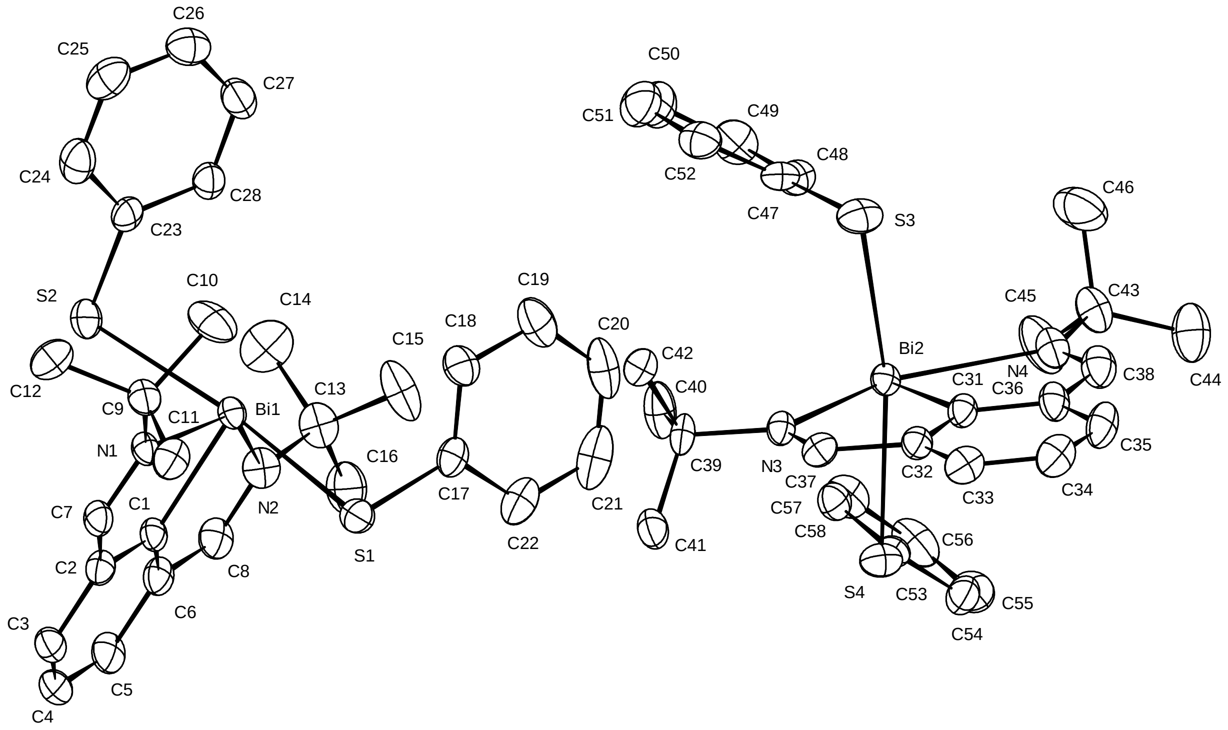

Figure S78: The molecular structure of **3SPh**. H atoms have been removed for clarity.

X-ray Crystal Structure Analysis of **3SPh**:

C_{28} H_{33} Bi N_2 S_2, M_r = 670.66 g mol^{-1}, yellow plate, crystal size 0.142 x 0.123 x 0.021 mm^3, Monoclinic, space group $P2_1$ [4], a = 16.1742(9) Å, b = 10.9502(6) Å, c = 16.5543(10) Å, β = 104.188(4)°, V = 2842.5(3) $Å^3$, T = 100(2) K, Z = 4, D_{calc} = 1.567 g·cm^3, λ = 0.71073 Å, μ(Mo-K_α) = 6.367 mm^{-1}, Gaussian absorption correction ($T_{\min}$ = 0.47024, $T_{\max}$ = 0.88275), Bruker-AXS D8 Venture with Photon III detector and IµS Diamond microfocus Mo-anode X-ray source, 2.026 < θ < 31.168°, 267502 measured reflections, 18204 independent reflections, 14720 reflections with $I > 2\sigma(I)$, R_{int} = 0.1153. The structure was solved by *SHELXT* and refined by full-matrix least-squares (*SHELXL*) against F^2 to R_1 = 0.0327 [$I > 2\sigma(I)$], wR_2 = 0.0672 [all data], 607 parameters, 1 restraints and an absolute structure parameter x = -0.024(3).

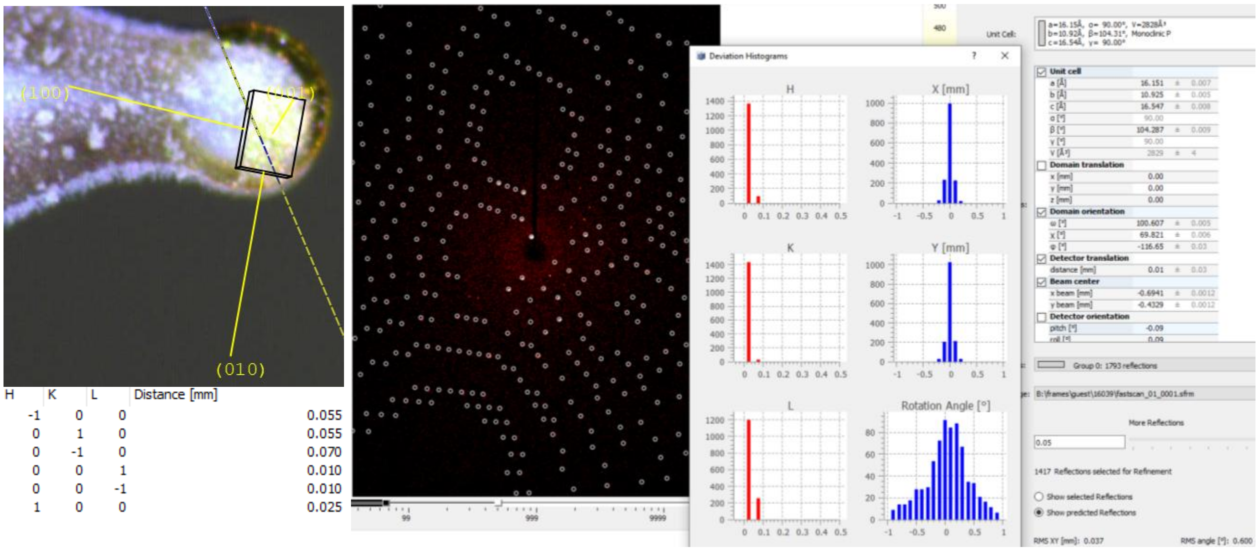

Figure S79: Crystal faces and unit cell determination/refinement of **3SPh**.

INTENSITY STATISTICS FOR DATASET

Resolution	#Data	#Theory	%Complete	Redundancy	Mean I	Mean I/s	Rmerge	Rsigma
Inf - 2.89	144	147	98.0	31.20	144.94	42.40	0.0586	0.0289
2.89 - 1.91	342	342	100.0	37.43	115.35	44.46	0.0610	0.0192
1.91 - 1.50	488	488	100.0	37.45	75.06	39.55	0.0738	0.0206
1.50 - 1.31	472	472	100.0	37.71	58.44	34.50	0.0835	0.0222
1.31 - 1.18	509	509	100.0	37.19	45.40	30.96	0.0980	0.0242
1.18 - 1.10	437	437	100.0	35.70	40.43	28.38	0.1116	0.0261
1.10 - 1.03	528	528	100.0	35.54	31.80	25.02	0.1328	0.0299
1.03 - 0.98	460	460	100.0	31.40	27.61	21.98	0.1425	0.0340
0.98 - 0.94	441	441	100.0	29.04	23.97	20.21	0.1622	0.0382
0.94 - 0.90	513	513	100.0	26.84	20.97	17.56	0.1751	0.0432
0.90 - 0.87	459	459	100.0	25.99	18.98	16.35	0.1956	0.0479
0.87 - 0.84	523	523	100.0	25.71	16.26	14.75	0.2151	0.0537
0.84 - 0.81	594	594	100.0	25.12	14.52	13.28	0.2342	0.0596
0.81 - 0.79	450	450	100.0	23.94	13.20	11.95	0.2512	0.0668
0.79 - 0.77	513	513	100.0	24.16	11.04	10.43	0.2887	0.0770
0.77 - 0.75	562	562	100.0	23.43	9.60	9.42	0.3174	0.0881
0.75 - 0.74	281	281	100.0	22.78	10.36	9.65	0.3043	0.0857
0.74 - 0.72	648	648	100.0	21.11	8.29	7.61	0.3530	0.1084
0.72 - 0.71	367	367	100.0	19.58	7.40	6.79	0.3889	0.1256
0.71 - 0.70	364	364	100.0	20.59	6.72	6.34	0.4183	0.1348
0.70 - 0.69	453	526	86.1	14.80	6.47	5.24	0.4197	0.1901
0.79 - 0.69	3188	3261	97.8	20.89	8.61	7.99	0.3404	0.1083
Inf - 0.69	9548	9624	99.2	27.74	28.84	18.88	0.1217	0.0374

Complete .cif-data of the compound are available under the CCDC number CCDC-**2490750**.

Table S8: Crystal data and structure refinement of compound **3SPh**.

Identification code	16039	
Empirical formula	$C_{28} H_{33} Bi N_2 S_2$	
Color	yellow	
Formula weight	670.66 g·mol^{-1}	
Temperature	100(2) K	
Wavelength	0.71073 Å	
Crystal system	Monoclinic	
Space group	$P2_1$, (no. 4)	
Unit cell dimensions	a = 16.1742(9) Å	α= 90°.
	b = 10.9502(6) Å	β= 104.188(4)°.
	c = 16.5543(10) Å	γ = 90°.
Volume	2842.5(3) Å^3	
Z	4	
Density (calculated)	1.567 Mg·m^{-3}	
Absorption coefficient	6.367 mm^{-1}	
F(000)	1320 e	
Crystal size	0.142 x 0.123 x 0.021 mm^3	
θ range for data collection	2.026 to 31.168°.	
Index ranges	$-23 \le h \le 23, -15\le k \le 15, -24 \le l \le 24$	
Reflections collected	267502	
Independent reflections	18204 [R_{int} = 0.1153]	
Reflections with I>2σ(I)	14720	
Completeness to θ = 25.242°	99.9 %	
Absorption correction	Gaussian	
Max. and min. transmission	0.88275 and 0.47024	
Refinement method	Full-matrix least-squares on F^2	
Data / restraints / parameters	18204 / 1 / 607	
Goodness-of-fit on F^2	1.028	
Final R indices [I>2σ(I)]	R_1 = 0.0327	wR^2 = 0.0610
R indices (all data)	R_1 = 0.0512	wR^2 = 0.0672
Absolute structure parameter	-0.024(3)	
Extinction coefficient	n/a	
Largest diff. peak and hole	1.261 and -1.269 e·Å^{-3}	

Table S9: Bond lengths [Å] and angles [°] of compound **3SPh**

Bi(1)-S(1)	2.7876(19)	Bi(1)-S(2)	2.714(2)
Bi(1)-N(1)	2.461(5)	Bi(1)-N(2)	2.576(8)
Bi(1)-C(1)	2.211(6)	S(1)-C(17)	1.772(8)
S(2)-C(23)	1.774(9)	N(1)-C(7)	1.270(8)
N(1)-C(9)	1.508(8)	N(2)-C(8)	1.286(11)
N(2)-C(13)	1.487(11)	C(1)-C(2)	1.374(9)
C(1)-C(6)	1.383(9)	C(2)-C(3)	1.400(9)
C(2)-C(7)	1.476(9)	C(3)-H(3)	0.9500
C(3)-C(4)	1.383(10)	C(4)-H(4)	0.9500
C(4)-C(5)	1.384(11)	C(5)-H(5)	0.9500
C(5)-C(6)	1.399(10)	C(6)-C(8)	1.459(11)
C(7)-H(7)	0.9500	C(8)-H(8)	0.9500
C(9)-C(10)	1.539(11)	C(9)-C(11)	1.517(11)
C(9)-C(12)	1.521(10)	C(10)-H(10A)	0.9800
C(10)-H(10B)	0.9800	C(10)-H(10C)	0.9800
C(11)-H(11A)	0.9800	C(11)-H(11B)	0.9800
C(11)-H(11C)	0.9800	C(12)-H(12A)	0.9800
C(12)-H(12B)	0.9800	C(12)-H(12C)	0.9800
C(13)-C(14)	1.532(12)	C(13)-C(15)	1.524(12)
C(13)-C(16)	1.524(12)	C(14)-H(14A)	0.9800
C(14)-H(14B)	0.9800	C(14)-H(14C)	0.9800
C(15)-H(15A)	0.9800	C(15)-H(15B)	0.9800
C(15)-H(15C)	0.9800	C(16)-H(16A)	0.9800
C(16)-H(16B)	0.9800	C(16)-H(16C)	0.9800
C(17)-C(18)	1.399(11)	C(17)-C(22)	1.383(10)
C(18)-H(18)	0.9500	C(18)-C(19)	1.398(11)
C(19)-H(19)	0.9500	C(19)-C(20)	1.385(13)
C(20)-H(20)	0.9500	C(20)-C(21)	1.361(15)
C(21)-H(21)	0.9500	C(21)-C(22)	1.373(13)
C(22)-H(22)	0.9500	C(23)-C(24)	1.404(12)
C(23)-C(28)	1.399(11)	C(24)-H(24)	0.9500
C(24)-C(25)	1.383(12)	C(25)-H(25)	0.9500
C(25)-C(26)	1.368(12)	C(26)-H(26)	0.9500

C(26)-C(27)	1.387(11)	C(27)-H(27)	0.9500
C(27)-C(28)	1.381(11)	C(28)-H(28)	0.9500
Bi(2)-S(3)	2.716(2)	Bi(2)-S(4)	2.7741(19)
Bi(2)-N(3)	2.454(6)	Bi(2)-N(4)	2.593(7)
Bi(2)-C(31)	2.204(6)	S(3)-C(47)	1.777(9)
S(4)-C(53)	1.760(8)	N(3)-C(37)	1.264(9)
N(3)-C(39)	1.494(8)	N(4)-C(38)	1.275(11)
N(4)-C(43)	1.482(10)	C(31)-C(32)	1.387(9)
C(31)-C(36)	1.387(9)	C(32)-C(33)	1.403(9)
C(32)-C(37)	1.472(9)	C(33)-H(33)	0.9500
C(33)-C(34)	1.381(10)	C(34)-H(34)	0.9500
C(34)-C(35)	1.390(12)	C(35)-H(35)	0.9500
C(35)-C(36)	1.413(10)	C(36)-C(38)	1.476(11)
C(37)-H(37)	0.9500	C(38)-H(38)	0.9500
C(39)-C(40)	1.517(11)	C(39)-C(41)	1.520(10)
C(39)-C(42)	1.540(10)	C(40)-H(40A)	0.9800
C(40)-H(40B)	0.9800	C(40)-H(40C)	0.9800
C(41)-H(41A)	0.9800	C(41)-H(41B)	0.9800
C(41)-H(41C)	0.9800	C(42)-H(42A)	0.9800
C(42)-H(42B)	0.9800	C(42)-H(42C)	0.9800
C(43)-C(44)	1.515(12)	C(43)-C(45)	1.518(13)
C(43)-C(46)	1.532(13)	C(44)-H(44A)	0.9800
C(44)-H(44B)	0.9800	C(44)-H(44C)	0.9800
C(45)-H(45A)	0.9800	C(45)-H(45B)	0.9800
C(45)-H(45C)	0.9800	C(46)-H(46A)	0.9800
C(46)-H(46B)	0.9800	C(46)-H(46C)	0.9800
C(47)-C(48)	1.393(11)	C(47)-C(52)	1.387(11)
C(48)-H(48)	0.9500	C(48)-C(49)	1.385(11)
C(49)-H(49)	0.9500	C(49)-C(50)	1.378(13)
C(50)-H(50)	0.9500	C(50)-C(51)	1.355(13)
C(51)-H(51)	0.9500	C(51)-C(52)	1.377(12)
C(52)-H(52)	0.9500	C(53)-C(54)	1.392(10)
C(53)-C(58)	1.395(11)	C(54)-H(54)	0.9500
C(54)-C(55)	1.368(12)	C(55)-H(55)	0.9500
C(55)-C(56)	1.367(13)	C(56)-H(56)	0.9500
C(56)-C(57)	1.384(12)	C(57)-H(57)	0.9500

C(57)-C(58)	1.401(10)	C(58)-H(58)	0.9500
S(2)-Bi(1)-S(1)	165.43(8)	N(1)-Bi(1)-S(1)	80.83(14)
N(1)-Bi(1)-S(2)	84.66(15)	N(1)-Bi(1)-N(2)	141.6(2)
N(2)-Bi(1)-S(1)	99.07(18)	N(2)-Bi(1)-S(2)	93.09(16)
C(1)-Bi(1)-S(1)	89.29(17)	C(1)-Bi(1)-S(2)	87.29(16)
C(1)-Bi(1)-N(1)	71.3(2)	C(1)-Bi(1)-N(2)	70.3(2)
C(17)-S(1)-Bi(1)	97.0(3)	C(23)-S(2)-Bi(1)	95.7(3)
C(7)-N(1)-Bi(1)	113.2(4)	C(7)-N(1)-C(9)	120.2(6)
C(9)-N(1)-Bi(1)	126.6(4)	C(8)-N(2)-Bi(1)	110.9(6)
C(8)-N(2)-C(13)	121.9(7)	C(13)-N(2)-Bi(1)	127.2(5)
C(2)-C(1)-Bi(1)	118.8(5)	C(2)-C(1)-C(6)	121.2(6)
C(6)-C(1)-Bi(1)	120.0(5)	C(1)-C(2)-C(3)	119.5(6)
C(1)-C(2)-C(7)	117.6(6)	C(3)-C(2)-C(7)	122.9(6)
C(2)-C(3)-H(3)	120.2	C(4)-C(3)-C(2)	119.6(7)
C(4)-C(3)-H(3)	120.2	C(3)-C(4)-H(4)	119.7
C(3)-C(4)-C(5)	120.7(7)	C(5)-C(4)-H(4)	119.7
C(4)-C(5)-H(5)	120.2	C(4)-C(5)-C(6)	119.6(7)
C(6)-C(5)-H(5)	120.2	C(1)-C(6)-C(5)	119.3(7)
C(1)-C(6)-C(8)	119.1(6)	C(5)-C(6)-C(8)	121.5(7)
N(1)-C(7)-C(2)	119.1(6)	N(1)-C(7)-H(7)	120.5
C(2)-C(7)-H(7)	120.5	N(2)-C(8)-C(6)	119.7(7)
N(2)-C(8)-H(8)	120.2	C(6)-C(8)-H(8)	120.2
N(1)-C(9)-C(10)	106.8(6)	N(1)-C(9)-C(11)	108.9(6)
N(1)-C(9)-C(12)	108.5(6)	C(11)-C(9)-C(10)	110.3(7)
C(11)-C(9)-C(12)	112.1(6)	C(12)-C(9)-C(10)	110.1(7)
C(9)-C(10)-H(10A)	109.5	C(9)-C(10)-H(10B)	109.5
C(9)-C(10)-H(10C)	109.5	H(10A)-C(10)-H(10B)	109.5
H(10A)-C(10)-H(10C)	109.5	H(10B)-C(10)-H(10C)	109.5
C(9)-C(11)-H(11A)	109.5	C(9)-C(11)-H(11B)	109.5
C(9)-C(11)-H(11C)	109.5	H(11A)-C(11)-H(11B)	109.5
H(11A)-C(11)-H(11C)	109.5	H(11B)-C(11)-H(11C)	109.5
C(9)-C(12)-H(12A)	109.5	C(9)-C(12)-H(12B)	109.5
C(9)-C(12)-H(12C)	109.5	H(12A)-C(12)-H(12B)	109.5
H(12A)-C(12)-H(12C)	109.5	H(12B)-C(12)-H(12C)	109.5
N(2)-C(13)-C(14)	106.6(7)	N(2)-C(13)-C(15)	106.7(7)

N(2)-C(13)-C(16)	113.8(7)	C(15)-C(13)-C(14)	108.7(8)
C(15)-C(13)-C(16)	110.2(8)	C(16)-C(13)-C(14)	110.6(7)
C(13)-C(14)-H(14A)	109.5	C(13)-C(14)-H(14B)	109.5
C(13)-C(14)-H(14C)	109.5	H(14A)-C(14)-H(14B)	109.5
H(14A)-C(14)-H(14C)	109.5	H(14B)-C(14)-H(14C)	109.5
C(13)-C(15)-H(15A)	109.5	C(13)-C(15)-H(15B)	109.5
C(13)-C(15)-H(15C)	109.5	H(15A)-C(15)-H(15B)	109.5
H(15A)-C(15)-H(15C)	109.5	H(15B)-C(15)-H(15C)	109.5
C(13)-C(16)-H(16A)	109.5	C(13)-C(16)-H(16B)	109.5
C(13)-C(16)-H(16C)	109.5	H(16A)-C(16)-H(16B)	109.5
H(16A)-C(16)-H(16C)	109.5	H(16B)-C(16)-H(16C)	109.5
C(18)-C(17)-S(1)	121.2(6)	C(22)-C(17)-S(1)	120.4(7)
C(22)-C(17)-C(18)	118.4(8)	C(17)-C(18)-H(18)	120.5
C(19)-C(18)-C(17)	119.1(8)	C(19)-C(18)-H(18)	120.5
C(18)-C(19)-H(19)	119.4	C(20)-C(19)-C(18)	121.2(9)
C(20)-C(19)-H(19)	119.4	C(19)-C(20)-H(20)	120.6
C(21)-C(20)-C(19)	118.9(8)	C(21)-C(20)-H(20)	120.6
C(20)-C(21)-H(21)	119.5	C(20)-C(21)-C(22)	120.9(9)
C(22)-C(21)-H(21)	119.5	C(17)-C(22)-H(22)	119.3
C(21)-C(22)-C(17)	121.5(9)	C(21)-C(22)-H(22)	119.3
C(24)-C(23)-S(2)	120.7(6)	C(28)-C(23)-S(2)	121.7(6)
C(28)-C(23)-C(24)	117.6(8)	C(23)-C(24)-H(24)	119.7
C(25)-C(24)-C(23)	120.7(7)	C(25)-C(24)-H(24)	119.7
C(24)-C(25)-H(25)	119.5	C(26)-C(25)-C(24)	121.1(7)
C(26)-C(25)-H(25)	119.5	C(25)-C(26)-H(26)	120.4
C(25)-C(26)-C(27)	119.1(8)	C(27)-C(26)-H(26)	120.4
C(26)-C(27)-H(27)	119.6	C(28)-C(27)-C(26)	120.7(7)
C(28)-C(27)-H(27)	119.6	C(23)-C(28)-H(28)	119.6
C(27)-C(28)-C(23)	120.8(7)	C(27)-C(28)-H(28)	119.6
S(3)-Bi(2)-S(4)	164.39(7)	N(3)-Bi(2)-S(3)	84.02(14)
N(3)-Bi(2)-S(4)	80.38(14)	N(3)-Bi(2)-N(4)	142.0(2)
N(4)-Bi(2)-S(3)	92.03(16)	N(4)-Bi(2)-S(4)	99.91(17)
C(31)-Bi(2)-S(3)	86.40(17)	C(31)-Bi(2)-S(4)	88.27(17)
C(31)-Bi(2)-N(3)	72.0(2)	C(31)-Bi(2)-N(4)	70.0(2)
C(47)-S(3)-Bi(2)	97.9(3)	C(53)-S(4)-Bi(2)	97.0(3)
C(37)-N(3)-Bi(2)	112.5(5)	C(37)-N(3)-C(39)	121.7(6)

C(39)-N(3)-Bi(2)	125.8(4)	C(38)-N(4)-Bi(2)	111.1(5)
C(38)-N(4)-C(43)	121.5(7)	C(43)-N(4)-Bi(2)	127.4(5)
C(32)-C(31)-Bi(2)	118.0(4)	C(36)-C(31)-Bi(2)	120.8(5)
C(36)-C(31)-C(32)	121.2(6)	C(31)-C(32)-C(33)	119.2(6)
C(31)-C(32)-C(37)	117.3(6)	C(33)-C(32)-C(37)	123.5(6)
C(32)-C(33)-H(33)	120.1	C(34)-C(33)-C(32)	119.8(7)
C(34)-C(33)-H(33)	120.1	C(33)-C(34)-H(34)	119.3
C(33)-C(34)-C(35)	121.3(7)	C(35)-C(34)-H(34)	119.3
C(34)-C(35)-H(35)	120.6	C(34)-C(35)-C(36)	118.9(7)
C(36)-C(35)-H(35)	120.6	C(31)-C(36)-C(35)	119.5(7)
C(31)-C(36)-C(38)	118.5(7)	C(35)-C(36)-C(38)	122.0(7)
N(3)-C(37)-C(32)	120.2(6)	N(3)-C(37)-H(37)	119.9
C(32)-C(37)-H(37)	119.9	N(4)-C(38)-C(36)	119.6(7)
N(4)-C(38)-H(38)	120.2	C(36)-C(38)-H(38)	120.2
N(3)-C(39)-C(40)	107.5(6)	N(3)-C(39)-C(41)	109.2(6)
N(3)-C(39)-C(42)	107.8(6)	C(40)-C(39)-C(41)	110.8(7)
C(40)-C(39)-C(42)	110.1(7)	C(41)-C(39)-C(42)	111.4(6)
C(39)-C(40)-H(40A)	109.5	C(39)-C(40)-H(40B)	109.5
C(39)-C(40)-H(40C)	109.5	H(40A)-C(40)-H(40B)	109.5
H(40A)-C(40)-H(40C)	109.5	H(40B)-C(40)-H(40C)	109.5
C(39)-C(41)-H(41A)	109.5	C(39)-C(41)-H(41B)	109.5
C(39)-C(41)-H(41C)	109.5	H(41A)-C(41)-H(41B)	109.5
H(41A)-C(41)-H(41C)	109.5	H(41B)-C(41)-H(41C)	109.5
C(39)-C(42)-H(42A)	109.5	C(39)-C(42)-H(42B)	109.5
C(39)-C(42)-H(42C)	109.5	H(42A)-C(42)-H(42B)	109.5
H(42A)-C(42)-H(42C)	109.5	H(42B)-C(42)-H(42C)	109.5
N(4)-C(43)-C(44)	114.6(7)	N(4)-C(43)-C(45)	106.8(7)
N(4)-C(43)-C(46)	105.7(7)	C(44)-C(43)-C(45)	109.3(8)
C(44)-C(43)-C(46)	111.2(8)	C(45)-C(43)-C(46)	109.1(9)
C(43)-C(44)-H(44A)	109.5	C(43)-C(44)-H(44B)	109.5
C(43)-C(44)-H(44C)	109.5	H(44A)-C(44)-H(44B)	109.5
H(44A)-C(44)-H(44C)	109.5	H(44B)-C(44)-H(44C)	109.5
C(43)-C(45)-H(45A)	109.5	C(43)-C(45)-H(45B)	109.5
C(43)-C(45)-H(45C)	109.5	H(45A)-C(45)-H(45B)	109.5
H(45A)-C(45)-H(45C)	109.5	H(45B)-C(45)-H(45C)	109.5
C(43)-C(46)-H(46A)	109.5	C(43)-C(46)-H(46B)	109.5

C(43)-C(46)-H(46C)	109.5	H(46A)-C(46)-H(46B)	109.5
H(46A)-C(46)-H(46C)	109.5	H(46B)-C(46)-H(46C)	109.5
C(48)-C(47)-S(3)	121.5(6)	C(52)-C(47)-S(3)	120.8(7)
C(52)-C(47)-C(48)	117.7(8)	C(47)-C(48)-H(48)	119.7
C(49)-C(48)-C(47)	120.5(8)	C(49)-C(48)-H(48)	119.7
C(48)-C(49)-H(49)	119.9	C(50)-C(49)-C(48)	120.2(8)
C(50)-C(49)-H(49)	119.9	C(49)-C(50)-H(50)	120.1
C(51)-C(50)-C(49)	119.7(8)	C(51)-C(50)-H(50)	120.1
C(50)-C(51)-H(51)	119.7	C(50)-C(51)-C(52)	120.7(8)
C(52)-C(51)-H(51)	119.7	C(47)-C(52)-H(52)	119.4
C(51)-C(52)-C(47)	121.1(8)	C(51)-C(52)-H(52)	119.4
C(54)-C(53)-S(4)	120.4(6)	C(54)-C(53)-C(58)	118.2(7)
C(58)-C(53)-S(4)	121.4(6)	C(53)-C(54)-H(54)	119.2
C(55)-C(54)-C(53)	121.6(8)	C(55)-C(54)-H(54)	119.2
C(54)-C(55)-H(55)	119.6	C(56)-C(55)-C(54)	120.8(8)
C(56)-C(55)-H(55)	119.6	C(55)-C(56)-H(56)	120.5
C(55)-C(56)-C(57)	119.0(8)	C(57)-C(56)-H(56)	120.5
C(56)-C(57)-H(57)	119.5	C(56)-C(57)-C(58)	121.0(8)
C(58)-C(57)-H(57)	119.5	C(53)-C(58)-C(57)	119.3(7)
C(53)-C(58)-H(58)	120.3	C(57)-C(58)-H(58)	120.3

14.4 Single crystal structure analysis of **3Phth** tetrahydrofuran solvate

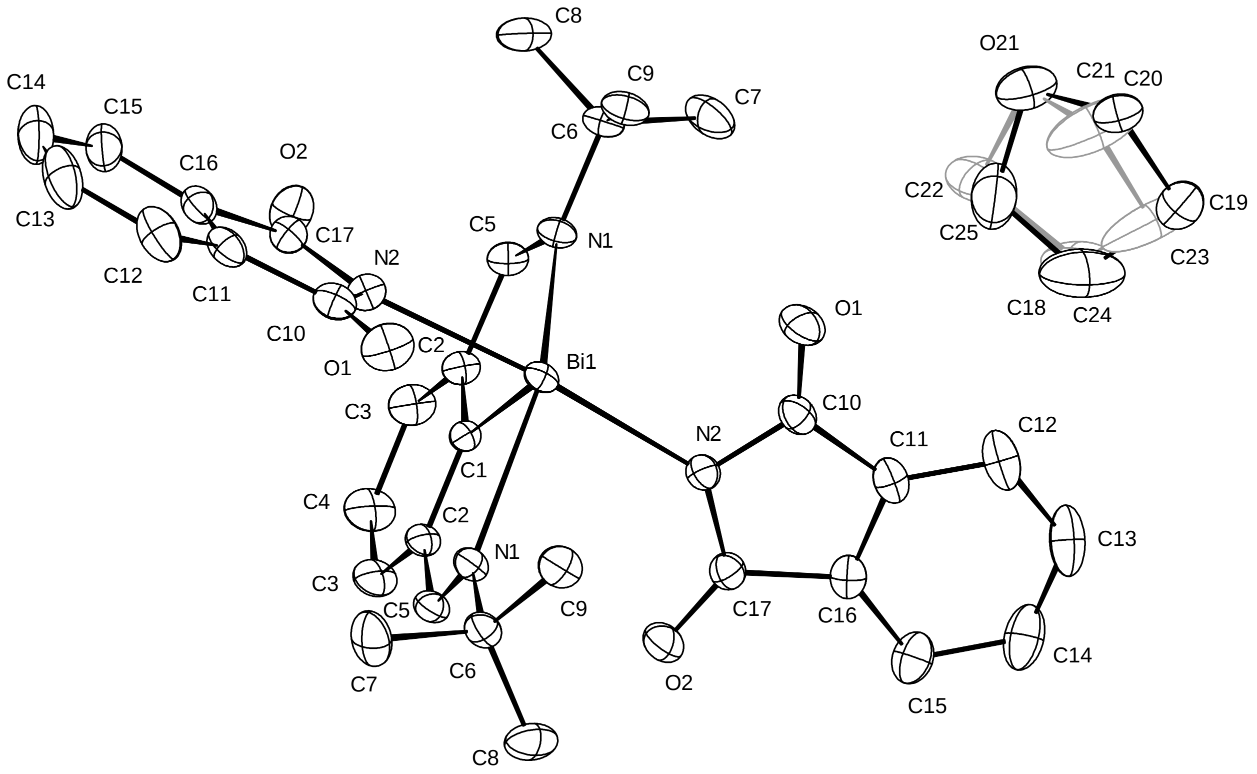

Figure S80: The molecular structure of **3Phth tetrahydrofuran solvate**. H atoms have been removed for clarity. Main structure shown in black and disordered parts shown in grey.

X-ray Crystal Structure Analysis of **3Phth tetrahydrofuran solvate**:

C_{40} H_{47} Bi N_4 O_6, M_r = 888.823 g mol^{-1}, colorless prism, crystal size 0.30 x 0.183 x 0.15 mm^3, Monoclinic, space group *C*2/*c* [15], a = 29.7162(19) Å, b = 9.0823(5) Å, c = 16.0182(10) Å, β= 120.518(2)°, *V* = 3724.3(4) Å^3, *T* = 100(2) K, *Z* = 4, D_{calc} = 1.585 g·cm^3, λ = 0.71073 Å, μ(*Mo-K*$_\alpha$) = 4.778 mm^{-1}, Gaussian absorption correction ($T_{\min}$ = 0.19625, $T_{\max}$ = 0.40719), Bruker-AXS Kappa Mach3 with APEX-II detector and IµS microfocus Mo-anode X-ray source, 1.59 < θ < 34.54°, 125002 measured reflections, 7815 independent reflections, 7444 reflections with $I > 2\sigma(I)$, R_{int} = 0.0360. The structure was solved by *SHELXT* and refined by full-matrix least-squares (*olex2.refine*) against F^2 to R_1 = 0.0126 [$I > 2\sigma(I)$], wR_2 = 0.0311 [all data], 285 parameters and 102 restraints.

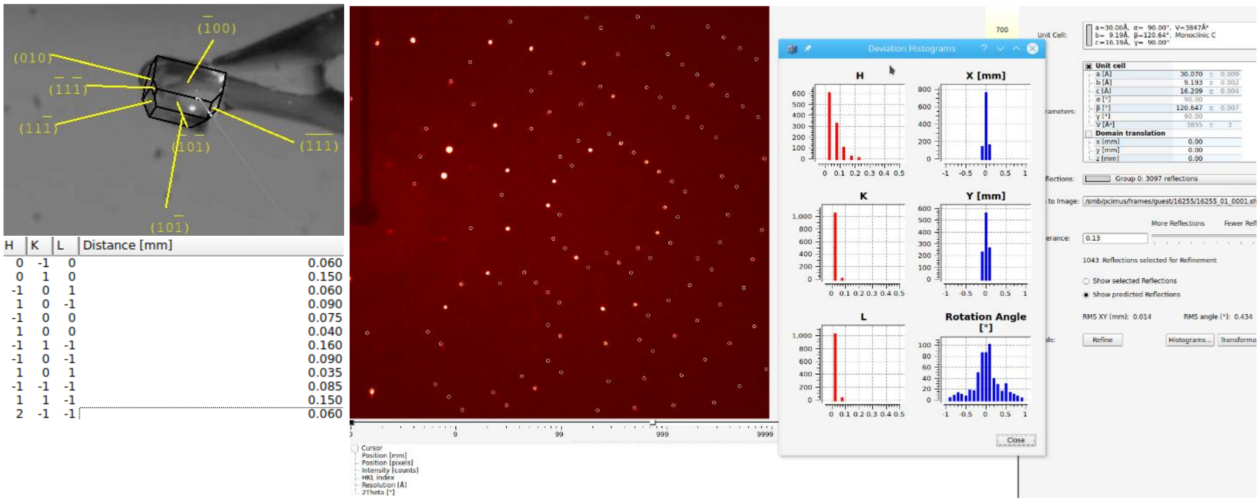

Figure S81: Crystal faces and unit cell determination/refinement of compound **3Phth tetrahydrofuran solvate**.

INTENSITY STATISTICS FOR DATASET

Resolution	#Data	#Theory	%Complete	Redundancy	Mean I	Mean I/s	Rmerge	Rsigma
Inf - 2.67	249	249	100.0	9.65	140.81	68.72	0.0314	0.0127
2.67 - 1.76	569	569	100.0	12.62	99.58	80.13	0.0304	0.0109
1.76 - 1.38	843	843	100.0	13.37	65.51	78.78	0.0287	0.0108
1.38 - 1.20	824	824	100.0	13.27	48.38	75.73	0.0287	0.0113
1.20 - 1.09	793	793	100.0	12.30	41.81	66.83	0.0305	0.0123
1.09 - 1.00	932	932	100.0	9.84	34.84	55.70	0.0316	0.0145
1.00 - 0.95	700	700	100.0	8.86	30.35	50.86	0.0337	0.0162
0.95 - 0.90	837	837	100.0	8.06	24.11	44.40	0.0366	0.0184
0.90 - 0.86	824	824	100.0	7.51	22.39	41.83	0.0379	0.0202
0.86 - 0.82	983	984	99.9	7.23	18.95	38.28	0.0415	0.0222
0.82 - 0.79	882	882	100.0	6.89	17.06	34.21	0.0438	0.0248
0.79 - 0.77	644	644	100.0	6.80	16.26	32.34	0.0454	0.0260
0.77 - 0.75	735	735	100.0	6.55	14.91	30.33	0.0488	0.0282
0.75 - 0.73	820	820	100.0	6.31	13.95	28.20	0.0508	0.0301
0.73 - 0.71	904	904	100.0	6.20	12.06	24.91	0.0564	0.0342
0.71 - 0.69	1004	1004	100.0	5.95	11.41	23.41	0.0602	0.0367
0.69 - 0.68	570	570	100.0	5.79	9.71	20.75	0.0671	0.0416
0.68 - 0.66	1190	1190	100.0	5.60	9.32	19.86	0.0722	0.0448
0.66 - 0.65	638	638	100.0	5.57	8.61	18.64	0.0757	0.0482
0.65 - 0.64	687	687	100.0	5.37	8.52	18.63	0.0789	0.0495
0.64 - 0.63	731	1050	69.6	2.86	7.27	14.00	0.0882	0.0692
0.73 - 0.63	5724	6043	94.7	5.26	9.72	20.34	0.0678	0.0439
Inf - 0.63	16359	16679	98.1	7.74	26.56	39.98	0.0350	0.0189

Complete .cif-data of the compound are available under the CCDC number CCDC-**2490747**.

For final refinement, the *anis -a* (anharmonic motion refinement) instruction in Olex2 refinement was applied to the Bi central atom. This applies higher Gram-Charlier coefficients to the refinement, taking the anharmonic motion of the central atom into account. It appears as a shashlik-like distribution, which indicates that anharmonic motion could presumably cause this observation.

To take this into account, higher order Gram-Charlier coefficients have been applied before final refinement cycles. This does not influence the reflection-to-parameter ratio significantly.
The SIMU C21 C20 C19 C23 C18 C24 C22 C25 were used to treat the anisotropic displacement parameters of the disordered parts of the structure.

Table S10: Crystal data and structure refinement of compound **3Phth tetrahydrofuran solvate**.

Identification code	16255	
Empirical formula	C_{40} H_{47} Bi N_4 O_6	
Color	colorless	
Formula weight	888.823 g·mol^{-1}	
Temperature	100(2) K	
Wavelength	0.71073 Å	
Crystal system	Monoclinic	
Space group	*C*2/*c*, (no. 15)	
Unit cell dimensions	a = 29.7162(19) Å	α= 90°.
	b = 9.0823(5) Å	β= 120.518(2)°.
	c = 16.0182(10) Å	γ = 90°.
Volume	3724.3(4) Å^3	
Z	4	
Density (calculated)	1.585 Mg·m^{-3}	
Absorption coefficient	4.778 mm^{-1}	
F(000)	1771.823 e	
Crystal size	0.30 x 0.183 x 0.15 mm^3	
θ range for data collection	1.59 to 34.54°.	
Index ranges	$-46 \le h \le 46$, $-14 \le k \le 14$, $-25 \le l \le 25$	
Reflections collected	125002	
Independent reflections	7815 [R_{int} = 0.0360]	
Reflections with I>2σ(I)	7444	
Completeness to θ = 25.2417°	100.00 %	
Absorption correction	Gaussian	
Max. and min. transmission	0.40719 and 0.19625	
Refinement method	Full-matrix least-squares on F^2	
Data / restraints / parameters	7815 / 102 / 285	
Goodness-of-fit on F^2	1.0277	
Final R indices [I>2σ(I)]	R_1 = 0.0126	wR^2 = 0.0303
R indices (all data)	R_1 = 0.0144	wR^2 = 0.0311
Largest diff. peak and hole	0.5931 and -0.4225 e·Å^{-3}	

Table S11: Bond lengths [Å] and angles [°] of compound **3Phth tetrahydrofuran solvate**.

Bi(1)-N(1)	2.4928(8)	Bi(1)-N(1)#1	2.4928(8)
Bi(1)-N(2)	2.4408(9)	Bi(1)-N(2)#1	2.4408(9)
Bi(1)-C(1)	2.1952(12)	O(1)-C(10)	1.2259(14)
O(2)-C(17)	1.2203(13)	N(1)-C(5)	1.2807(13)
N(1)-C(6)	1.4907(13)	N(2)-C(10)	1.3772(13)
N(2)-C(17)	1.3830(14)	C(1)-C(2)#1	1.3828(11)
C(1)-C(2)	1.3828(11)	C(2)-C(3)	1.3966(14)
C(2)-C(5)	1.4680(14)	C(3)-H(3)	0.9500
C(3)-C(4)	1.3927(14)	C(4)-H(4)	0.9500
C(5)-H(5)	0.9500	C(6)-C(7)	1.5259(17)
C(6)-C(8)	1.5282(17)	C(6)-C(9)	1.5250(15)
C(7)-H(7a)	0.9800	C(7)-H(7b)	0.9800
C(7)-H(7c)	0.9800	C(8)-H(8a)	0.9800
C(8)-H(8b)	0.9800	C(8)-H(8c)	0.9800
C(9)-H(9a)	0.9800	C(9)-H(9b)	0.9800
C(9)-H(9c)	0.9800	C(10)-C(11)	1.4976(16)
C(11)-C(12)	1.3845(15)	C(11)-C(16)	1.3831(16)
C(12)-H(12)	0.9500	C(12)-C(13)	1.393(2)
C(13)-H(13)	0.9500	C(13)-C(14)	1.381(3)
C(14)-H(14)	0.9500	C(14)-C(15)	1.3963(19)
C(15)-H(15)	0.9500	C(15)-C(16)	1.3812(17)
C(16)-C(17)	1.4992(15)	O(21)-C(20)	1.512(6)
O(21)-C(22)	1.480(7)	O(21)-C(21)	1.302(9)
O(21)-C(25)	1.417(9)	C(20)-H(20a)	0.9900
C(20)-H(20b)	0.9900	C(20)-C(19)	1.517(8)
C(19)-H(19a)	0.9900	C(19)-H(19b)	0.9900
C(19)-C(24)	1.560(10)	C(22)-H(22b)	0.9900
C(22)-H(22a)	0.9900	C(22)-C(18)	1.580(9)
C(18)-H(18b)	0.9900	C(18)-H(18a)	0.9900
C(18)-C(23)	1.440(10)	C(21)-C(23)	1.517(10)
C(21)-H(21a)	0.9900	C(21)-H(21b)	0.9900
C(25)-H(25b)	0.9900	C(25)-H(25a)	0.9900
C(25)-C(24)	1.440(11)	C(23)-H(23a)	0.9900

C(23)-H(23b)	0.9900	C(24)-H(24a)	0.9900
C(24)-H(24b)	0.9900		

N(1)-Bi(1)-N(1)#1	143.02(4)	N(2)#1-Bi(1)-N(1)	83.84(3)
N(2)-Bi(1)-N(1)	93.35(3)	N(2)#1-Bi(1)-N(1)#1	93.35(3)
N(2)-Bi(1)-N(1)#1	83.84(3)	N(2)-Bi(1)-N(2)#1	171.18(4)
C(1)-Bi(1)-N(1)#1	71.51(2)	C(1)-Bi(1)-N(1)	71.51(2)
C(1)-Bi(1)-N(2)#1	85.59(2)	C(1)-Bi(1)-N(2)	85.59(2)
C(5)-N(1)-Bi(1)#1	111.85(6)	C(6)-N(1)-Bi(1)#1	126.88(6)
C(6)-N(1)-C(5)	120.86(8)	C(10)-N(2)-Bi(1)#1	113.34(7)
C(17)-N(2)-Bi(1)#1	137.42(7)	C(17)-N(2)-C(10)	109.23(9)
C(2)-C(1)-Bi(1)	118.96(6)	C(2)#1-C(1)-Bi(1)	118.96(6)
C(2)#1-C(1)-C(2)	122.08(12)	C(3)-C(2)-C(1)	119.04(10)
C(5)-C(2)-C(1)	118.08(8)	C(5)-C(2)-C(3)	122.87(9)
H(3)-C(3)-C(2)	120.29(6)	C(4)-C(3)-C(2)	119.42(10)
C(4)-C(3)-H(3)	120.29(7)	C(3)#1-C(4)-C(3)	120.98(14)
H(4)-C(4)-C(3)	119.51(7)	H(4)-C(4)-C(3)#1	119.51(7)
C(2)-C(5)-N(1)	119.33(8)	H(5)-C(5)-N(1)	120.33(6)
H(5)-C(5)-C(2)	120.33(5)	C(7)-C(6)-N(1)	108.02(9)
C(8)-C(6)-N(1)	110.96(9)	C(8)-C(6)-C(7)	111.84(10)
C(9)-C(6)-N(1)	106.42(8)	C(9)-C(6)-C(7)	109.94(10)
C(9)-C(6)-C(8)	109.51(9)	H(7a)-C(7)-C(6)	109.5
H(7b)-C(7)-C(6)	109.5	H(7b)-C(7)-H(7a)	109.5
H(7c)-C(7)-C(6)	109.5	H(7c)-C(7)-H(7a)	109.5
H(7c)-C(7)-H(7b)	109.5	H(8a)-C(8)-C(6)	109.5
H(8b)-C(8)-C(6)	109.5	H(8b)-C(8)-H(8a)	109.5
H(8c)-C(8)-C(6)	109.5	H(8c)-C(8)-H(8a)	109.5
H(8c)-C(8)-H(8b)	109.5	H(9a)-C(9)-C(6)	109.5
H(9b)-C(9)-C(6)	109.5	H(9b)-C(9)-H(9a)	109.5
H(9c)-C(9)-C(6)	109.5	H(9c)-C(9)-H(9a)	109.5
H(9c)-C(9)-H(9b)	109.5	N(2)-C(10)-O(1)	124.59(11)
C(11)-C(10)-O(1)	126.67(10)	C(11)-C(10)-N(2)	108.75(9)
C(12)-C(11)-C(10)	132.34(11)	C(16)-C(11)-C(10)	106.72(9)
C(16)-C(11)-C(12)	120.95(12)	H(12)-C(12)-C(11)	121.28(8)
C(13)-C(12)-C(11)	117.44(13)	C(13)-C(12)-H(12)	121.28(8)
H(13)-C(13)-C(12)	119.36(8)	C(14)-C(13)-C(12)	121.29(12)

C(14)-C(13)-H(13)	119.36(8)	H(14)-C(14)-C(13)	119.32(8)
C(15)-C(14)-C(13)	121.35(13)	C(15)-C(14)-H(14)	119.32(9)
H(15)-C(15)-C(14)	121.60(9)	C(16)-C(15)-C(14)	116.80(13)
C(16)-C(15)-H(15)	121.60(8)	C(15)-C(16)-C(11)	122.17(11)
C(17)-C(16)-C(11)	106.92(9)	C(17)-C(16)-C(15)	130.91(11)
N(2)-C(17)-O(2)	126.41(10)	C(16)-C(17)-O(2)	125.22(10)
C(16)-C(17)-N(2)	108.37(9)	C(22)-O(21)-C(20)	118.2(4)
C(21)-O(21)-C(20)	11.7(6)	C(21)-O(21)-C(22)	108.8(4)
C(25)-O(21)-C(20)	98.8(4)	C(25)-O(21)-C(22)	21.5(4)
C(25)-O(21)-C(21)	88.5(5)	H(20a)-C(20)-O(21)	110.81(19)
H(20b)-C(20)-O(21)	110.81(18)	H(20b)-C(20)-H(20a)	108.9
C(19)-C(20)-O(21)	104.7(4)	C(19)-C(20)-H(20a)	110.8(2)
C(19)-C(20)-H(20b)	110.8(3)	H(19a)-C(19)-C(20)	112.0(2)
H(19b)-C(19)-C(20)	112.0(3)	H(19b)-C(19)-H(19a)	109.7
C(24)-C(19)-C(20)	98.9(5)	C(24)-C(19)-H(19a)	112.0(5)
C(24)-C(19)-H(19b)	112.0(4)	H(22b)-C(22)-O(21)	111.7(3)
H(22a)-C(22)-O(21)	111.7(3)	H(22a)-C(22)-H(22b)	109.5
C(18)-C(22)-O(21)	100.2(5)	C(18)-C(22)-H(22b)	111.7(3)
C(18)-C(22)-H(22a)	111.7(3)	H(18b)-C(18)-C(22)	110.6(3)
H(18a)-C(18)-C(22)	110.6(3)	H(18a)-C(18)-H(18b)	108.7
C(23)-C(18)-C(22)	105.7(4)	C(23)-C(18)-H(18b)	110.6(5)
C(23)-C(18)-H(18a)	110.6(5)	C(23)-C(21)-O(21)	106.6(6)
H(21a)-C(21)-O(21)	110.4(5)	H(21a)-C(21)-C(23)	110.4(5)
H(21b)-C(21)-O(21)	110.4(5)	H(21b)-C(21)-C(23)	110.4(5)
H(21b)-C(21)-H(21a)	108.6	H(25b)-C(25)-O(21)	108.9(4)
H(25a)-C(25)-O(21)	108.9(5)	H(25a)-C(25)-H(25b)	107.7
C(24)-C(25)-O(21)	113.3(6)	C(24)-C(25)-H(25b)	108.9(5)
C(24)-C(25)-H(25a)	108.9(4)	C(21)-C(23)-C(18)	105.0(6)
H(23a)-C(23)-C(18)	110.7(5)	H(23a)-C(23)-C(21)	110.7(4)
H(23b)-C(23)-C(18)	110.7(5)	H(23b)-C(23)-C(21)	110.7(5)
H(23b)-C(23)-H(23a)	108.8	C(25)-C(24)-C(19)	104.3(5)
H(24a)-C(24)-C(19)	110.9(4)	H(24a)-C(24)-C(25)	110.9(5)
H(24b)-C(24)-C(19)	110.9(4)	H(24b)-C(24)-C(25)	110.9(4)
H(24b)-C(24)-H(24a)	108.9		

Symmetry transformations used to generate equivalent atoms: #1 x+1,y,z+1

14.5 Single crystal structure analysis of **3Phth-OTf**

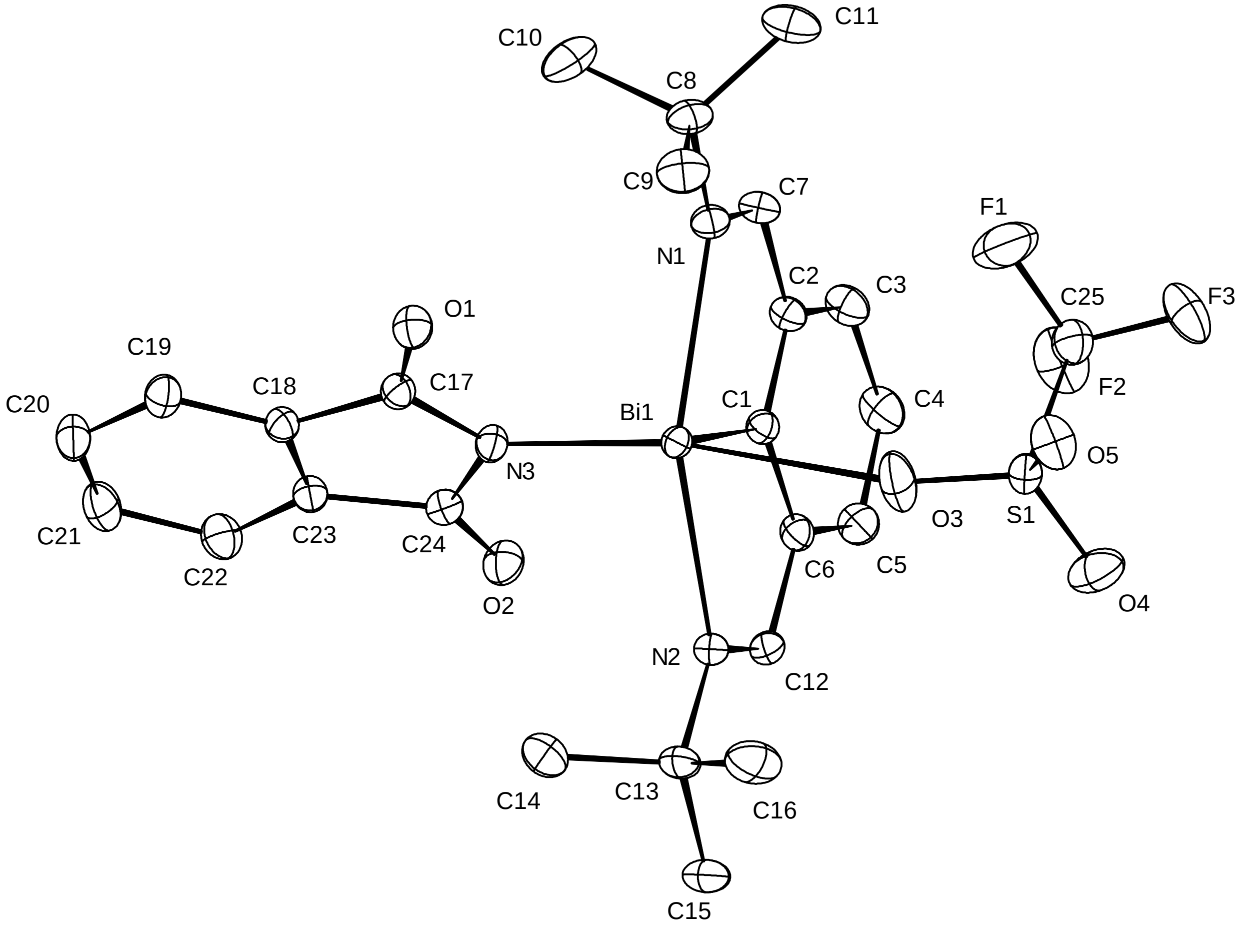

Figure S82: The molecular structure of **3Phth-OTf**. The H atoms have been removed for clarity, with the exception of the H atoms at the Sb central atom.

X-ray Crystal Structure Analysis of **3Phth-OTf**:

$C_{25} H_{27} Bi F_3 N_3 O_5 S$, M_r = 747.53 g mol^{-1}, colorless prism, crystal size 0.140 x 0.056 x 0.052 mm^3, Monoclinic, space group $P2_1/n$ [14], a = 14.6122(5) Å, b = 13.3465(4) Å, c = 14.8301(5) Å, β= 108.221(2)°, V = 2747.17(16) Å^3, T = 100(2) K, Z = 4, D_{calc} = 1.807 g·cm^3, λ = 0.71073 Å, $\mu(Mo\text{-}K_\alpha)$ = 6.554 mm^{-1}, Gaussian absorption correction ($T_{\min}$ = 0.42852, $T_{\max}$ = 0.78934), Bruker-AXS D8 Venture with Photon III detector and IµS Diamond microfocus Mo-anode X-ray source, 1.708 < θ < 33.844°, 623344 measured reflections, 11028 independent reflections, 9404 reflections with $I > 2\sigma(I)$, R_{int} = 0.1286. The structure was solved by *SHELXT* and refined by full-matrix least-squares (*SHELXL*) against F^2 to R_1 = 0.0196 [$I > 2\sigma(I)$], wR_2 = 0.0413 [all data], 349 parameters and 0 restraints.

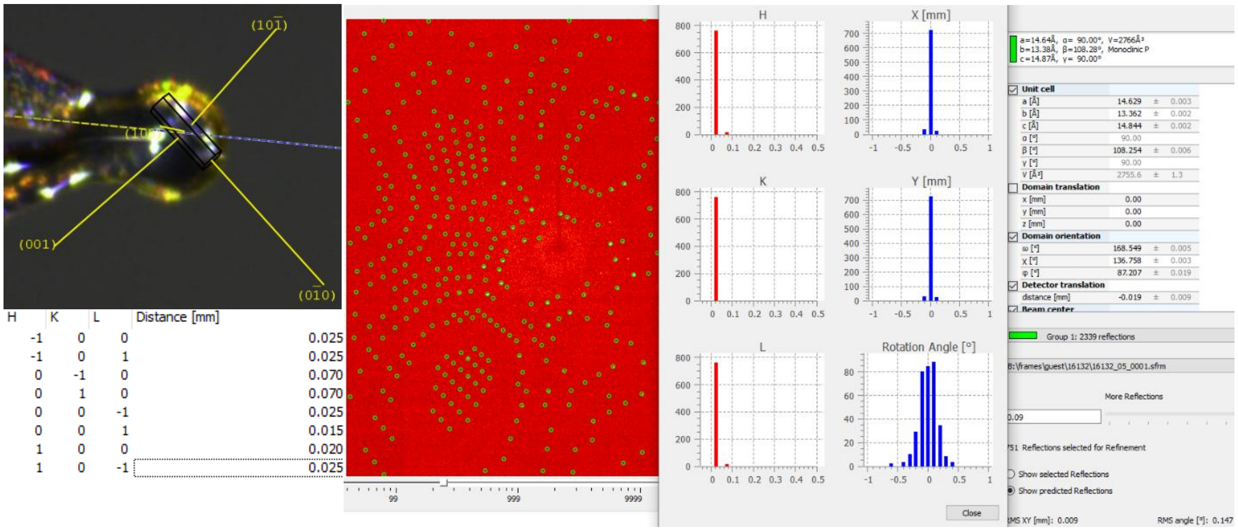

Figure S83: Crystal faces and unit cell determination/refinement of **3Phth-OTf**.

INTENSITY STATISTICS FOR DATASET

Resolution	#Data	#Theory	%Complete	Redundancy	Mean I	Mean I/s	Rmerge	Rsigma
Inf - 2.69	173	176	98.3	60.55	92.28	79.12	0.0341	0.0096
2.69 - 1.76	412	412	100.0	73.54	63.50	79.87	0.0405	0.0089
1.76 - 1.39	579	579	100.0	75.75	45.75	74.18	0.0542	0.0098
1.39 - 1.21	574	574	100.0	73.99	34.10	62.13	0.0714	0.0111
1.21 - 1.10	560	560	100.0	69.56	28.76	55.04	0.0856	0.0126
1.10 - 1.02	574	574	100.0	65.20	26.49	49.67	0.0962	0.0139
1.02 - 0.96	565	565	100.0	63.34	21.31	43.79	0.1143	0.0159
0.96 - 0.91	567	567	100.0	60.86	19.46	40.41	0.1295	0.0176
0.91 - 0.87	571	571	100.0	59.09	15.85	34.21	0.1529	0.0201
0.87 - 0.83	688	688	100.0	57.70	14.33	31.90	0.1777	0.0226
0.83 - 0.80	614	614	100.0	55.63	13.27	29.04	0.1961	0.0249
0.80 - 0.78	453	453	100.0	54.27	12.50	27.88	0.2099	0.0268
0.78 - 0.75	783	783	100.0	52.23	10.77	23.99	0.2411	0.0309
0.75 - 0.73	597	597	100.0	50.15	9.70	21.63	0.2650	0.0346
0.73 - 0.72	318	318	100.0	49.81	8.81	20.09	0.2927	0.0380
0.72 - 0.70	693	693	100.0	47.08	9.09	19.61	0.2845	0.0389
0.70 - 0.68	787	787	100.0	44.32	7.22	16.32	0.3246	0.0483
0.68 - 0.67	431	431	100.0	43.08	7.14	15.56	0.3314	0.0511
0.67 - 0.66	443	443	100.0	40.66	6.56	14.03	0.3467	0.0568
0.66 - 0.65	488	488	100.0	40.83	6.13	13.47	0.3676	0.0604
0.65 - 0.64	561	596	94.1	32.60	5.67	11.24	0.3848	0.0798
0.74 - 0.64	4040	4075	99.1	43.00	7.39	16.14	0.3190	0.0499
Inf - 0.64	11431	11469	99.7	55.48	18.94	34.51	0.1175	0.0197

Complete .cif-data of the compound are available under the CCDC number CCDC-**2490753**.

Table S12: Crystal data and structure refinement of **3Phth-OTf**.

Identification code	16132	
Empirical formula	C_{25} H_{27} Bi F_3 N_3 O_5 S	
Color	colorless	
Formula weight	747.53 g·mol^{-1}	
Temperature	100(2) K	
Wavelength	0.71073 Å	
Crystal system	Monoclinic	
Space group	$P2_1/n$, (no. 14)	
Unit cell dimensions	a = 14.6122(5) Å	α= 90°.
	b = 13.3465(4) Å	β= 108.221(2)°.
	c = 14.8301(5) Å	γ = 90°.
Volume	2747.17(16) Å^3	
Z	4	
Density (calculated)	1.807 Mg·m^{-3}	
Absorption coefficient	6.554 mm^{-1}	
F(000)	1456 e	
Crystal size	0.140 x 0.056 x 0.052 mm^3	
θ range for data collection	1.708 to 33.844°.	
Index ranges	$-22 \le h \le 22$, $-20 \le k \le 20$, $-23 \le l \le 23$	
Reflections collected	623344	
Independent reflections	11028 [R_{int} = 0.1286]	
Reflections with I>2σ(I)	9404	
Completeness to θ = 25.242°	100.0 %	
Absorption correction	Gaussian	
Max. and min. transmission	0.78934 and 0.42852	
Refinement method	Full-matrix least-squares on F^2	
Data / restraints / parameters	11028 / 0 / 349	
Goodness-of-fit on F^2	1.109	
Final R indices [I>2σ(I)]	R_1 = 0.0196	wR^2 = 0.0401
R indices (all data)	R_1 = 0.0274	wR^2 = 0.0413
Extinction coefficient	n/a	
Largest diff. peak and hole	0.743 and -1.183 e·Å^{-3}	

Table S13: Bond lengths [Å] and angles [°] of **3Phth-OTf**.

Bi(1)-O(3)	2.6208(15)	Bi(1)-N(1)	2.5194(16)
Bi(1)-N(2)	2.4778(16)	Bi(1)-N(3)	2.2596(15)
Bi(1)-C(1)	2.1940(18)	S(1)-O(3)	1.4422(16)
S(1)-O(4)	1.4349(16)	S(1)-O(5)	1.4375(16)
S(1)-C(25)	1.821(2)	F(1)-C(25)	1.338(3)
F(2)-C(25)	1.326(3)	F(3)-C(25)	1.328(2)
O(1)-C(17)	1.223(2)	O(2)-C(24)	1.217(2)
N(1)-C(7)	1.274(2)	N(1)-C(8)	1.495(2)
N(2)-C(12)	1.278(2)	N(2)-C(13)	1.485(2)
N(3)-C(17)	1.391(2)	N(3)-C(24)	1.398(2)
C(1)-C(2)	1.386(3)	C(1)-C(6)	1.394(2)
C(2)-C(3)	1.400(3)	C(2)-C(7)	1.467(3)
C(3)-H(3)	0.9500	C(3)-C(4)	1.387(3)
C(4)-H(4)	0.9500	C(4)-C(5)	1.403(3)
C(5)-H(5)	0.9500	C(5)-C(6)	1.394(3)
C(6)-C(12)	1.463(3)	C(7)-H(7)	0.9500
C(8)-C(9)	1.526(3)	C(8)-C(10)	1.527(3)
C(8)-C(11)	1.527(3)	C(9)-H(9A)	0.9800
C(9)-H(9B)	0.9800	C(9)-H(9C)	0.9800
C(10)-H(10A)	0.9800	C(10)-H(10B)	0.9800
C(10)-H(10C)	0.9800	C(11)-H(11A)	0.9800
C(11)-H(11B)	0.9800	C(11)-H(11C)	0.9800
C(12)-H(12)	0.9500	C(13)-C(14)	1.527(3)
C(13)-C(15)	1.529(3)	C(13)-C(16)	1.526(3)
C(14)-H(14A)	0.9800	C(14)-H(14B)	0.9800
C(14)-H(14C)	0.9800	C(15)-H(15A)	0.9800
C(15)-H(15B)	0.9800	C(15)-H(15C)	0.9800
C(16)-H(16A)	0.9800	C(16)-H(16B)	0.9800
C(16)-H(16C)	0.9800	C(17)-C(18)	1.489(3)
C(18)-C(19)	1.392(3)	C(18)-C(23)	1.392(3)
C(19)-H(19)	0.9500	C(19)-C(20)	1.394(3)
C(20)-H(20)	0.9500	C(20)-C(21)	1.397(3)
C(21)-H(21)	0.9500	C(21)-C(22)	1.393(3)

C(22)-H(22)	0.9500	C(22)-C(23)	1.379(3)
C(23)-C(24)	1.494(3)		

N(1)-Bi(1)-O(3)	95.15(5)	N(2)-Bi(1)-O(3)	79.24(5)
N(2)-Bi(1)-N(1)	142.51(5)	N(3)-Bi(1)-O(3)	168.28(6)
N(3)-Bi(1)-N(1)	93.00(5)	N(3)-Bi(1)-N(2)	89.17(5)
C(1)-Bi(1)-O(3)	83.71(6)	C(1)-Bi(1)-N(1)	70.82(6)
C(1)-Bi(1)-N(2)	71.73(6)	C(1)-Bi(1)-N(3)	91.06(6)
O(3)-S(1)-C(25)	102.89(10)	O(4)-S(1)-O(3)	114.63(11)
O(4)-S(1)-O(5)	115.13(10)	O(4)-S(1)-C(25)	103.73(10)
O(5)-S(1)-O(3)	114.27(10)	O(5)-S(1)-C(25)	104.06(10)
S(1)-O(3)-Bi(1)	165.12(11)	C(7)-N(1)-Bi(1)	111.85(12)
C(7)-N(1)-C(8)	121.43(16)	C(8)-N(1)-Bi(1)	126.42(12)
C(12)-N(2)-Bi(1)	111.90(12)	C(12)-N(2)-C(13)	123.30(17)
C(13)-N(2)-Bi(1)	124.64(12)	C(17)-N(3)-Bi(1)	116.97(12)
C(17)-N(3)-C(24)	109.86(15)	C(24)-N(3)-Bi(1)	131.98(12)
C(2)-C(1)-Bi(1)	119.99(13)	C(2)-C(1)-C(6)	121.13(16)
C(6)-C(1)-Bi(1)	118.77(13)	C(1)-C(2)-C(3)	119.48(17)
C(1)-C(2)-C(7)	117.58(16)	C(3)-C(2)-C(7)	122.77(17)
C(2)-C(3)-H(3)	120.1	C(4)-C(3)-C(2)	119.87(18)
C(4)-C(3)-H(3)	120.1	C(3)-C(4)-H(4)	119.8
C(3)-C(4)-C(5)	120.39(18)	C(5)-C(4)-H(4)	119.8
C(4)-C(5)-H(5)	120.1	C(6)-C(5)-C(4)	119.70(17)
C(6)-C(5)-H(5)	120.1	C(1)-C(6)-C(5)	119.38(17)
C(1)-C(6)-C(12)	117.32(16)	C(5)-C(6)-C(12)	123.30(17)
N(1)-C(7)-C(2)	119.73(17)	N(1)-C(7)-H(7)	120.1
C(2)-C(7)-H(7)	120.1	N(1)-C(8)-C(9)	105.66(15)
N(1)-C(8)-C(10)	107.73(16)	N(1)-C(8)-C(11)	111.87(17)
C(9)-C(8)-C(10)	110.23(18)	C(9)-C(8)-C(11)	110.48(17)
C(11)-C(8)-C(10)	110.72(18)	C(8)-C(9)-H(9A)	109.5
C(8)-C(9)-H(9B)	109.5	C(8)-C(9)-H(9C)	109.5
H(9A)-C(9)-H(9B)	109.5	H(9A)-C(9)-H(9C)	109.5
H(9B)-C(9)-H(9C)	109.5	C(8)-C(10)-H(10A)	109.5
C(8)-C(10)-H(10B)	109.5	C(8)-C(10)-H(10C)	109.5
H(10A)-C(10)-H(10B)	109.5	H(10A)-C(10)-H(10C)	109.5
H(10B)-C(10)-H(10C)	109.5	C(8)-C(11)-H(11A)	109.5

C(8)-C(11)-H(11B)	109.5	C(8)-C(11)-H(11C)	109.5
H(11A)-C(11)-H(11B)	109.5	H(11A)-C(11)-H(11C)	109.5
H(11B)-C(11)-H(11C)	109.5	N(2)-C(12)-C(6)	120.11(16)
N(2)-C(12)-H(12)	119.9	C(6)-C(12)-H(12)	119.9
N(2)-C(13)-C(14)	106.60(16)	N(2)-C(13)-C(15)	112.34(17)
N(2)-C(13)-C(16)	106.51(16)	C(14)-C(13)-C(15)	110.03(18)
C(16)-C(13)-C(14)	110.48(19)	C(16)-C(13)-C(15)	110.77(18)
C(13)-C(14)-H(14A)	109.5	C(13)-C(14)-H(14B)	109.5
C(13)-C(14)-H(14C)	109.5	H(14A)-C(14)-H(14B)	109.5
H(14A)-C(14)-H(14C)	109.5	H(14B)-C(14)-H(14C)	109.5
C(13)-C(15)-H(15A)	109.5	C(13)-C(15)-H(15B)	109.5
C(13)-C(15)-H(15C)	109.5	H(15A)-C(15)-H(15B)	109.5
H(15A)-C(15)-H(15C)	109.5	H(15B)-C(15)-H(15C)	109.5
C(13)-C(16)-H(16A)	109.5	C(13)-C(16)-H(16B)	109.5
C(13)-C(16)-H(16C)	109.5	H(16A)-C(16)-H(16B)	109.5
H(16A)-C(16)-H(16C)	109.5	H(16B)-C(16)-H(16C)	109.5
O(1)-C(17)-N(3)	124.26(17)	O(1)-C(17)-C(18)	127.78(17)
N(3)-C(17)-C(18)	107.94(15)	C(19)-C(18)-C(17)	131.16(18)
C(23)-C(18)-C(17)	107.33(16)	C(23)-C(18)-C(19)	121.50(18)
C(18)-C(19)-H(19)	121.4	C(18)-C(19)-C(20)	117.12(19)
C(20)-C(19)-H(19)	121.4	C(19)-C(20)-H(20)	119.4
C(19)-C(20)-C(21)	121.16(19)	C(21)-C(20)-H(20)	119.4
C(20)-C(21)-H(21)	119.5	C(22)-C(21)-C(20)	121.08(19)
C(22)-C(21)-H(21)	119.5	C(21)-C(22)-H(22)	121.2
C(23)-C(22)-C(21)	117.69(19)	C(23)-C(22)-H(22)	121.2
C(18)-C(23)-C(24)	107.42(16)	C(22)-C(23)-C(18)	121.43(18)
C(22)-C(23)-C(24)	131.15(18)	O(2)-C(24)-N(3)	125.65(17)
O(2)-C(24)-C(23)	126.91(18)	N(3)-C(24)-C(23)	107.44(15)
F(1)-C(25)-S(1)	110.56(15)	F(2)-C(25)-S(1)	111.91(16)
F(2)-C(25)-F(1)	107.8(2)	F(2)-C(25)-F(3)	108.06(18)
F(3)-C(25)-S(1)	111.31(16)	F(3)-C(25)-F(1)	107.00(19)

14.6 Single crystal structure analysis of **3OMs**

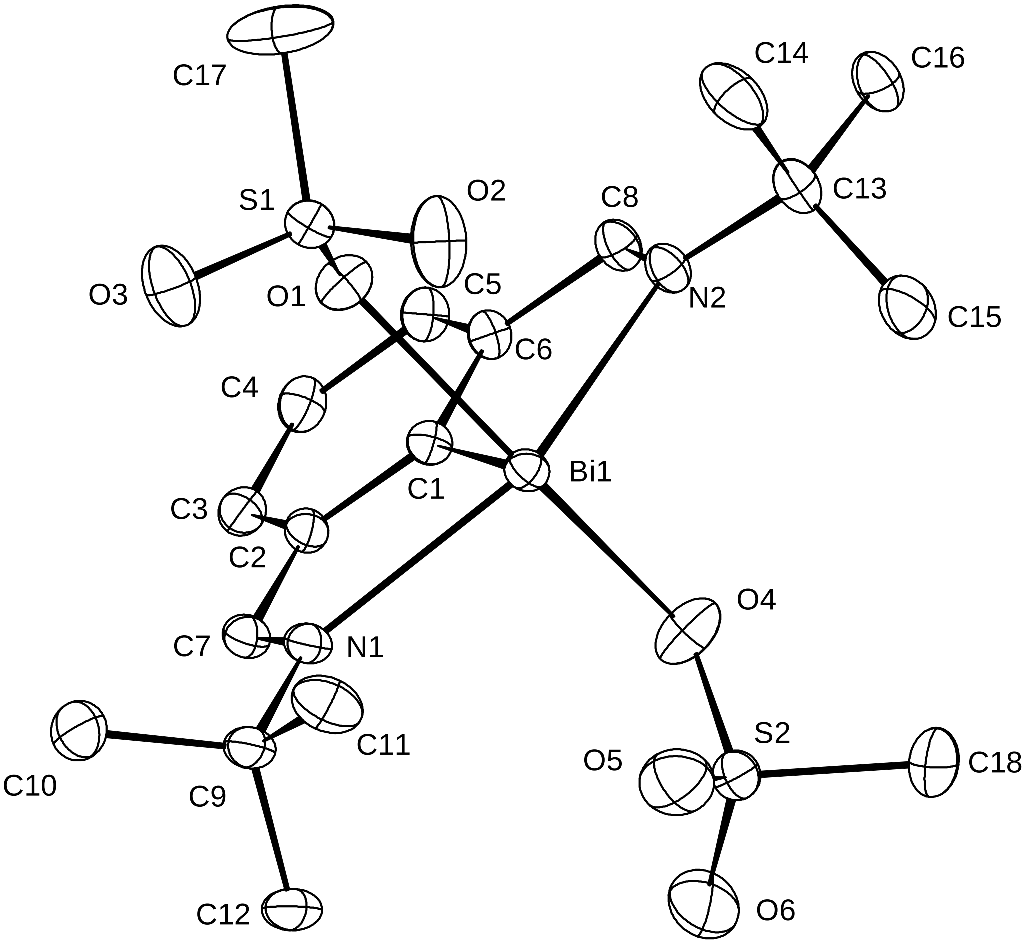

Figure S84: The molecular structure of **3OMs**. The H atoms have been removed for clarity.

X-ray Crystal Structure Analysis of 3OMs:
C_{18} H_{29} Bi N_2 O_6 S_2, M_r = 642.553 g mol^{-1}, colorless plate, crystal size 0.100 x 0.076 x 0.012 mm^3, Triclinic, space group *P*-1 [2], a = 9.0274(13) Å, b = 9.6842(16) Å, c = 14.312(2) Å, α = 106.854(4)°, β = 90.403(4)°, γ = 96.298(4)°, *V* = 1189.2(3) Å^3, *T* = 100(2) K, *Z* = 2, D_{calc} = 1.794 g·cm^3, λ = 0.71073 Å, $\mu(Mo\text{-}K_\alpha)$ = 7.610 mm^{-1}, Gaussian absorption correction ($T_{\min}$ = 0.60566, $T_{\max}$ = 0.92799), Bruker-AXS D8 Venture with Photon III detector and IµS Diamond microfocus Mo-anode X-ray source, 2.21 < θ < 31.00°, 254736 measured reflections, 7578 independent reflections, 7209 reflections with $I > 2\sigma(I)$, R_{int} = 0.0659. The structure was solved by SHELXT and refined by full-matrix least-squares (SHELXL). The final structure refinement was performed by olex2.refine 1.5 (L-M) together with NoSpherA2 (atomic form factors) against F^2 to R_1 = 0.0148 [$I > 2\sigma(I)$], wR_2 = 0.0359 [all data] with 523 parameters and 42 restraints.

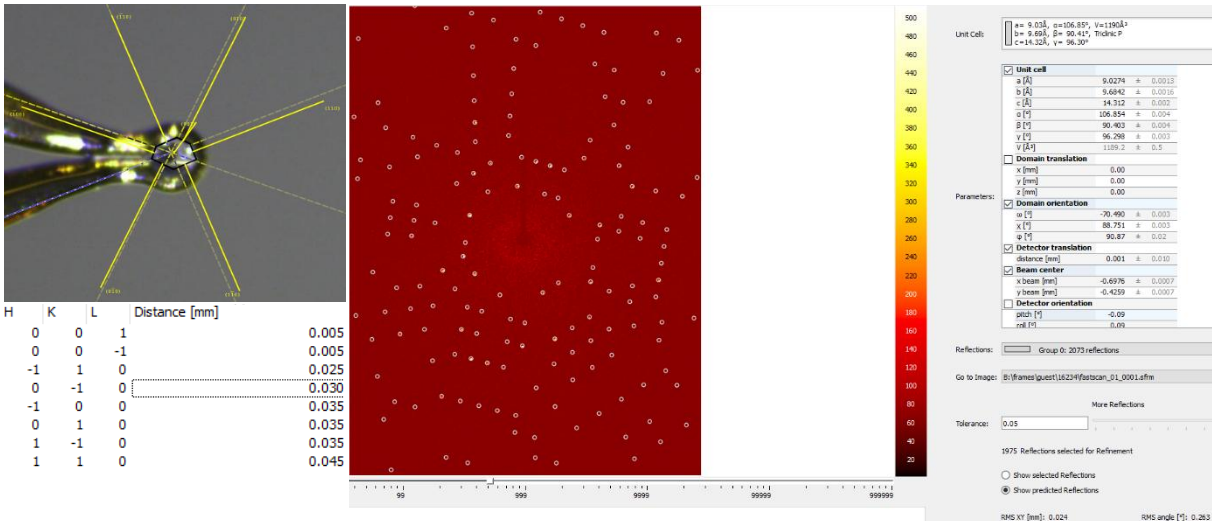

Figure S85: Crystal faces and unit cell determination/refinement of **3OMs**.

```
INTENSITY STATISTICS FOR DATASET
```

Resolution	#Data	#Theory	%Complete	Redundancy	Mean I	Mean I/s	Rmerge	Rsigma
Inf - 2.73	241	250	96.4	17.15	159.58	78.30	0.0377	0.0182
2.73 - 1.83	564	564	100.0	22.22	123.18	87.58	0.0338	0.0106
1.83 - 1.46	794	794	100.0	23.76	86.48	80.62	0.0348	0.0101
1.46 - 1.27	812	812	100.0	22.60	65.34	66.89	0.0422	0.0119
1.27 - 1.16	776	776	100.0	21.00	53.47	57.82	0.0507	0.0138
1.16 - 1.07	864	864	100.0	18.78	45.50	49.35	0.0564	0.0160
1.07 - 1.01	764	764	100.0	17.71	44.20	46.86	0.0607	0.0175
1.01 - 0.96	800	800	100.0	17.25	34.73	39.87	0.0704	0.0203
0.96 - 0.92	766	766	100.0	16.10	31.43	35.86	0.0786	0.0230
0.92 - 0.88	874	874	100.0	15.79	25.99	31.16	0.0889	0.0264
0.88 - 0.85	836	836	100.0	15.26	24.51	29.54	0.0989	0.0292
0.85 - 0.82	894	894	100.0	14.76	22.77	26.61	0.1058	0.0315
0.82 - 0.80	702	702	100.0	14.63	20.02	24.12	0.1137	0.0346
0.80 - 0.78	788	788	100.0	14.32	19.41	22.94	0.1223	0.0368
0.78 - 0.76	836	836	100.0	13.57	18.64	20.95	0.1290	0.0399
0.76 - 0.74	928	928	100.0	13.09	16.14	18.35	0.1410	0.0457
0.74 - 0.73	514	514	100.0	12.86	14.08	16.48	0.1514	0.0508
0.73 - 0.71	1182	1182	100.0	12.52	13.95	15.85	0.1555	0.0537
0.71 - 0.70	508	508	100.0	12.29	13.18	15.18	0.1627	0.0574
0.70 - 0.69	656	656	100.0	12.19	12.10	13.74	0.1740	0.0620
0.69 - 0.68	749	798	93.9	10.12	11.03	12.25	0.1791	0.0772
0.78 - 0.68	5373	5422	99.1	12.40	14.36	16.31	0.1511	0.0528
Inf - 0.68	15848	15906	99.6	16.02	35.73	35.82	0.0643	0.0223

Complete .cif-data of the compound are available under the CCDC number CCDC-**2491196**.

The final structure refinement was carried out with using aspherical scattering factors with NoSpherA2. DFT-calculated with ORCA using a B3LYP functional and def2-TZVPP basis set, whereby the H atom positions were refined using anisotropic atomic displacement parameters. NoSpherA2 implementation of HAR makes use of tailor-made aspherical atomic form factors calculated on-the-fly from a Hirshfeld-partitioned electron density (ED). The ED was calculated from a gaussian basis set single determinant SCF wave function by DFT using selected functional for the asymmetric unit.[35] The following options were used:

SOFTWARE: ORCA 6.0
PARTITIONING: NoSpherA2
INT ACCURACY: High
METHOD: B3LYP
BASIS SET: x2c-TZVPP
CHARGE: 0
MULTIPLICITY: 1
RELATIVISTIC: ZORA
DATE: 2025-09-22_12-51-16

Table S14: Crystal data and structure refinement of **3OMs**.

Identification code	16234	
Empirical formula	C_{18} H_{29} Bi N_2 O_6 S_2	
Color	colorless	
Formula weight	642.553 g·mol^{-1}	
Temperature	100(2) K	
Wavelength	0.71073 Å	
Crystal system	Triclinic	
Space group	*P*-1, (no. 2)	
Unit cell dimensions	a = 9.0274(13) Å	α= 106.854(4)°.
	b = 9.6842(16) Å	β= 90.403(4)°.
	c = 14.312(2) Å	γ = 96.298(4)°.
Volume	1189.2(3) Å^3	
Z	2	
Density (calculated)	1.794 Mg·m^{-3}	
Absorption coefficient	7.610 mm^{-1}	
F(000)	622.233 e	
Crystal size	0.100 x 0.076 x 0.012 mm^3	
θ range for data collection	2.21 to 31.00°.	
Index ranges	$-13 \leq h \leq 13$, $-14\leq k \leq 14$, $-21 \leq l \leq 21$	
Reflections collected	254736	
Independent reflections	7578 [R_{int} = 0.0659]	
Reflections with I>2σ(I)	7209	
Completeness to θ = 25.2417°	99.98 %	
Absorption correction	Gaussian	
Max. and min. transmission	0.92799 and 0.60566	
Refinement method	Full-matrix least-squares on F^2	
Data / restraints / parameters	7578 / 42 / 523	
Goodness-of-fit on F^2	1.0417	
Final R indices [I>2σ(I)]	R_1 = 0.0148	wR^2 = 0.0352
R indices (all data)	R_1 = 0.0165	wR^2 = 0.0359
Largest diff. peak and hole	1.5962 and -0.4513 e·Å^{-3}	

Table S15: Bond lengths [Å] and angles [°] of **3OMs**.

Bi(1)-O(1)	2.3534(13)	Bi(1)-O(4)	2.3740(14)
Bi(1)-N(1)	2.4787(15)	Bi(1)-N(2)	2.4782(14)
Bi(1)-C(1)	2.1800(16)	S(1)-O(1)	1.4925(13)
S(1)-O(2)	1.4400(15)	S(1)-O(3)	1.4352(15)
S(1)-C(17)	1.756(3)	S(2)-O(4)	1.4865(15)
S(2)-O(5)	1.4470(18)	S(2)-O(6)	1.4340(17)
S(2)-C(18)	1.754(2)	N(1)-C(7)	1.285(2)
N(1)-C(9)	1.490(2)	N(2)-C(8)	1.279(2)
N(2)-C(13)	1.480(2)	C(1)-C(2)	1.386(2)
C(1)-C(6)	1.391(2)	C(2)-C(3)	1.405(2)
C(2)-C(7)	1.469(2)	C(3)-H(3)	1.02(2)
C(3)-C(4)	1.396(3)	C(4)-H(4)	1.07(2)
C(4)-C(5)	1.397(2)	C(5)-H(5)	1.08(2)
C(5)-C(6)	1.400(2)	C(6)-C(8)	1.471(2)
C(7)-H(7)	1.09(2)	C(8)-H(8)	1.09(2)
C(9)-C(10)	1.525(3)	C(9)-C(11)	1.534(3)
C(9)-C(12)	1.532(3)	C(10)-H(10A)	1.11(3)
C(10)-H(10B)	1.07(3)	C(10)-H(10C)	1.08(3)
C(11)-H(11A)	1.07(3)	C(11)-H(11B)	1.14(3)
C(11)-H(11C)	1.04(3)	C(12)-H(12A)	1.10(3)
C(12)-H(12B)	1.10(3)	C(12)-H(12C)	1.08(3)
C(13)-C(14)	1.531(3)	C(13)-C(15)	1.534(3)
C(13)-C(16)	1.519(3)	C(14)-H(14A)	1.07(3)
C(14)-H(14B)	1.12(3)	C(14)-H(14C)	1.09(3)
C(15)-H(15A)	1.11(3)	C(15)-H(15B)	1.11(3)
C(15)-H(15C)	1.09(3)	C(16)-H(16A)	1.11(3)
C(16)-H(16B)	1.12(3)	C(16)-H(16C)	1.08(3)
C(17)-H(17A)	0.97(5)	C(17)-H(17B)	1.10(4)
C(17)-H(17C)	1.12(4)	C(18)-H(18A)	1.16(4)
C(18)-H(18B)	1.11(4)	C(18)-H(18C)	1.06(4)
O(4)-Bi(1)-O(1)	157.66(5)	N(1)-Bi(1)-O(1)	84.80(5)
N(1)-Bi(1)-O(4)	93.48(5)	N(2)-Bi(1)-O(1)	85.26(5)

N(2)-Bi(1)-O(4)	83.03(5)	N(2)-Bi(1)-N(1)	143.68(5)
C(1)-Bi(1)-O(1)	78.50(5)	C(1)-Bi(1)-O(4)	79.78(5)
C(1)-Bi(1)-N(1)	72.08(6)	C(1)-Bi(1)-N(2)	71.72(5)
O(2)-S(1)-O(1)	110.51(9)	O(3)-S(1)-O(1)	111.42(9)
O(3)-S(1)-O(2)	115.17(9)	C(17)-S(1)-O(1)	103.26(11)
C(17)-S(1)-O(2)	108.24(16)	C(17)-S(1)-O(3)	107.45(15)
O(5)-S(2)-O(4)	109.08(9)	O(6)-S(2)-O(4)	112.19(12)
O(6)-S(2)-O(5)	115.36(12)	C(18)-S(2)-O(4)	104.95(12)
C(18)-S(2)-O(5)	107.85(14)	C(18)-S(2)-O(6)	106.77(13)
S(1)-O(1)-Bi(1)	122.53(7)	S(2)-O(4)-Bi(1)	117.51(8)
C(7)-N(1)-Bi(1)	111.57(11)	C(9)-N(1)-Bi(1)	127.01(11)
C(9)-N(1)-C(7)	121.41(15)	C(8)-N(2)-Bi(1)	112.39(11)
C(13)-N(2)-Bi(1)	123.52(10)	C(13)-N(2)-C(8)	124.08(15)
C(2)-C(1)-Bi(1)	118.94(12)	C(6)-C(1)-Bi(1)	119.21(12)
C(6)-C(1)-C(2)	121.83(15)	C(3)-C(2)-C(1)	119.00(16)
C(7)-C(2)-C(1)	117.77(15)	C(7)-C(2)-C(3)	123.19(15)
H(3)-C(3)-C(2)	118.6(16)	C(4)-C(3)-C(2)	119.60(16)
C(4)-C(3)-H(3)	121.7(16)	H(4)-C(4)-C(3)	119.1(13)
C(5)-C(4)-C(3)	120.88(16)	C(5)-C(4)-H(4)	120.0(13)
H(5)-C(5)-C(4)	122.4(15)	C(6)-C(5)-C(4)	119.40(16)
C(6)-C(5)-H(5)	118.2(15)	C(5)-C(6)-C(1)	119.28(15)
C(8)-C(6)-C(1)	117.55(14)	C(8)-C(6)-C(5)	123.17(15)
C(2)-C(7)-N(1)	119.60(15)	H(7)-C(7)-N(1)	120.9(15)
H(7)-C(7)-C(2)	119.5(15)	C(6)-C(8)-N(2)	119.12(15)
H(8)-C(8)-N(2)	122.9(14)	H(8)-C(8)-C(6)	117.9(14)
C(10)-C(9)-N(1)	110.52(16)	C(11)-C(9)-N(1)	107.23(15)
C(11)-C(9)-C(10)	109.55(18)	C(12)-C(9)-N(1)	108.08(15)
C(12)-C(9)-C(10)	111.08(16)	C(12)-C(9)-C(11)	110.30(17)
H(10A)-C(10)-C(9)	110.7(17)	H(10B)-C(10)-C(9)	113.1(16)
H(10B)-C(10)-H(10A)	106(3)	H(10C)-C(10)-C(9)	109.8(15)
H(10C)-C(10)-H(10A)	108(2)	H(10C)-C(10)-H(10B)	109(2)
H(11A)-C(11)-C(9)	113.5(17)	H(11B)-C(11)-C(9)	110.3(14)
H(11B)-C(11)-H(11A)	107(2)	H(11C)-C(11)-C(9)	110.9(18)
H(11C)-C(11)-H(11A)	108(3)	H(11C)-C(11)-H(11B)	106(2)
H(12A)-C(12)-C(9)	108.3(17)	H(12B)-C(12)-C(9)	110.8(13)
H(12B)-C(12)-H(12A)	112(2)	H(12C)-C(12)-C(9)	110.5(16)

H(12C)-C(12)-H(12A)	109(2)	H(12C)-C(12)-H(12B)	106(2)
C(14)-C(13)-N(2)	106.33(15)	C(15)-C(13)-N(2)	106.33(15)
C(15)-C(13)-C(14)	109.82(19)	C(16)-C(13)-N(2)	113.71(15)
C(16)-C(13)-C(14)	110.74(16)	C(16)-C(13)-C(15)	109.75(17)
H(14A)-C(14)-C(13)	111.9(16)	H(14B)-C(14)-C(13)	112.3(17)
H(14B)-C(14)-H(14A)	101(2)	H(14C)-C(14)-C(13)	109(2)
H(14C)-C(14)-H(14A)	113(3)	H(14C)-C(14)-H(14B)	109(2)
H(15A)-C(15)-C(13)	113(2)	H(15B)-C(15)-C(13)	108.3(18)
H(15B)-C(15)-H(15A)	109(3)	H(15C)-C(15)-C(13)	110.5(18)
H(15C)-C(15)-H(15A)	105(2)	H(15C)-C(15)-H(15B)	112(3)
H(16A)-C(16)-C(13)	113.2(16)	H(16B)-C(16)-C(13)	112.3(16)
H(16B)-C(16)-H(16A)	108(3)	H(16C)-C(16)-C(13)	109.2(17)
H(16C)-C(16)-H(16A)	109(2)	H(16C)-C(16)-H(16B)	104(2)
H(17A)-C(17)-S(1)	108(3)	H(17B)-C(17)-S(1)	108(2)
H(17B)-C(17)-H(17A)	113(4)	H(17C)-C(17)-S(1)	106.3(19)
H(17C)-C(17)-H(17A)	116(4)	H(17C)-C(17)-H(17B)	106(3)
H(18A)-C(18)-S(2)	98(2)	H(18B)-C(18)-S(2)	111(2)
H(18B)-C(18)-H(18A)	117(3)	H(18C)-C(18)-S(2)	104(2)
H(18C)-C(18)-H(18A)	116(3)	H(18C)-C(18)-H(18B)	109(3)

14.7 Single crystal structure analysis of **3OTf**

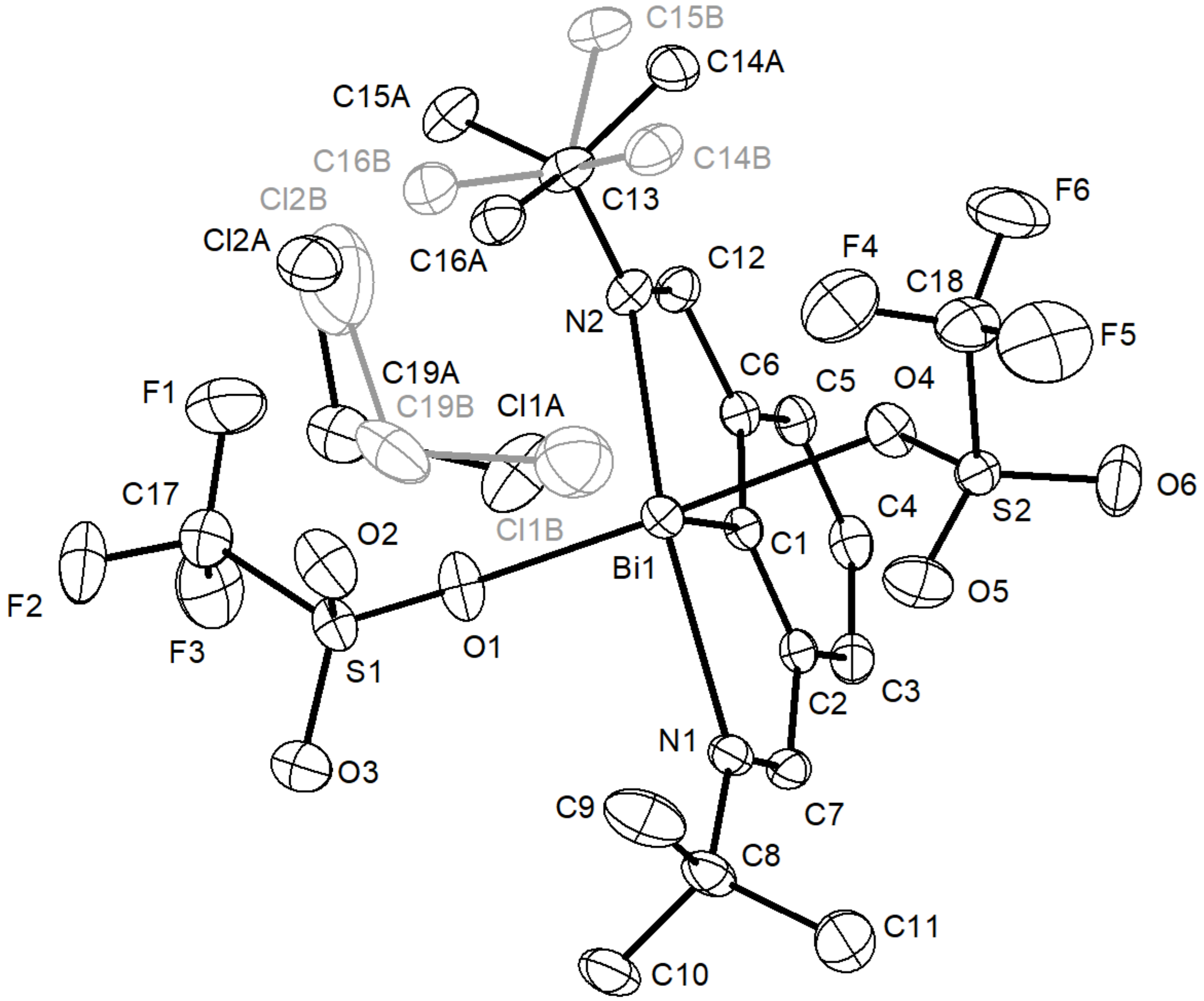

Figure S86: The molecular structure of **3OTf**. The H atoms have been removed for clarity. Main structure shown in black and disordered parts shown in grey.

X-ray Crystal Structure Analysis of **3OTf**:

$C_{19} H_{25} Bi Cl_2 F_6 N_2 O_6 S_2$, M_r = 835.41 g mol^{-1}, colorless prism, crystal size 0.192 x 0.175 x 0.140 mm^3, Triclinic, space group *P*-1 [2], a = 9.1000(12) Å, b = 12.7117(15) Å, c = 13.5571(17) Å, α = 81.089(3)°, β = 75.126(3)°, γ = 70.007(3)°, V = 1420.5(3) Å^3, T = 100(2) K, Z = 2, D_{calc} = 1.953 g·cm^3, λ = 0.71073 Å, $\mu(Mo\text{-}K_\alpha)$ = 6.617 mm^{-1}, Gaussian absorption correction ($T_{\min}$ = 0.39178, $T_{\max}$ = 0.60396), Bruker-AXS D8 Venture with Photon III detector and IµS Diamond microfocus Mo-anode X-ray source, 2.397 < θ < 31.524°, 125086 measured reflections, 9453 independent reflections, 8799 reflections with $I > 2\sigma(I)$, R_{int} = 0.0885. The structure was solved by *SHELXT* and refined by full-matrix least-squares (*SHELXL*) against F^2 to R_1 = 0.0211 [$I > 2\sigma(I)$], wR_2 = 0.0486 [all data], 399 parameters and 0 restraints.

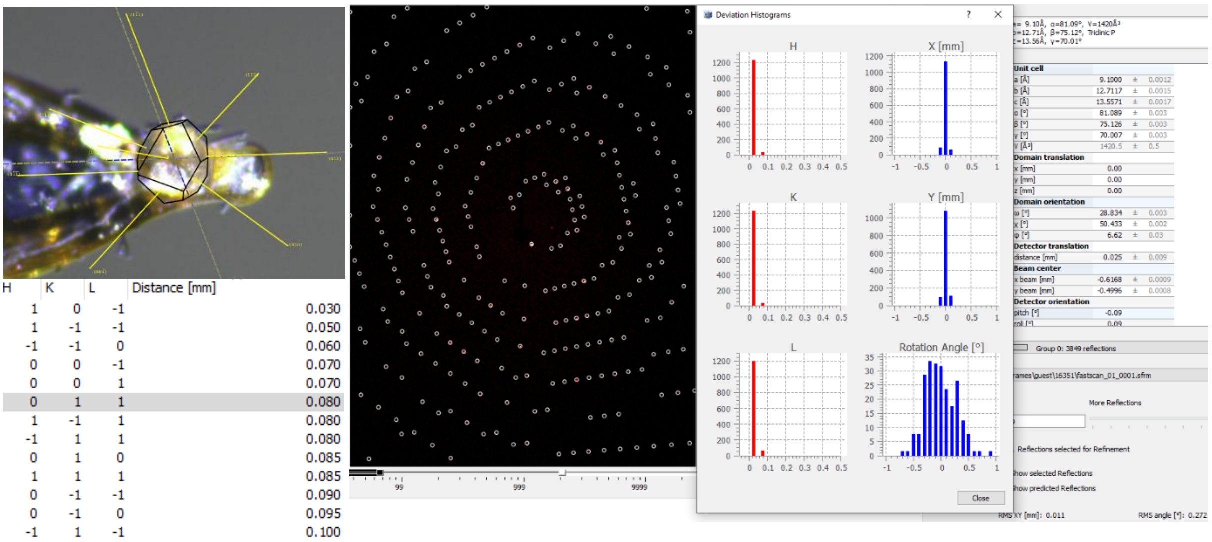

Figure S87: Crystal faces and unit cell determination/refinement of **3OTf**.

INTENSITY STATISTICS FOR DATASET

Resolution	#Data	#Theory	%Complete	Redundancy	Mean I	Mean I/s	Rmerge	Rsigma
Inf - 2.71	284	300	94.7	6.27	142.70	28.96	0.0511	0.0463
2.71 - 1.81	670	684	98.0	8.13	104.04	31.45	0.0514	0.0383
1.81 - 1.46	941	944	99.7	9.60	75.41	33.00	0.0568	0.0269
1.46 - 1.27	960	960	100.0	9.72	55.30	30.00	0.0664	0.0279
1.27 - 1.15	1006	1006	100.0	9.73	45.40	27.03	0.0735	0.0300
1.15 - 1.07	946	946	100.0	8.89	39.92	23.57	0.0788	0.0334
1.07 - 1.01	902	902	100.0	7.74	35.85	21.25	0.0826	0.0380
1.01 - 0.96	986	986	100.0	7.22	27.62	18.27	0.0965	0.0448
0.96 - 0.92	890	890	100.0	6.88	25.82	17.27	0.0999	0.0489
0.92 - 0.88	1126	1126	100.0	6.40	20.66	14.53	0.1127	0.0575
0.88 - 0.85	914	914	100.0	6.31	19.60	13.87	0.1206	0.0616
0.85 - 0.82	1104	1104	100.0	6.13	17.94	12.65	0.1254	0.0662
0.82 - 0.80	820	820	100.0	6.05	16.05	11.58	0.1399	0.0719
0.80 - 0.78	950	950	100.0	5.95	15.41	11.15	0.1410	0.0755
0.78 - 0.76	950	950	100.0	5.77	14.39	10.68	0.1478	0.0813
0.76 - 0.74	1178	1178	100.0	5.11	11.38	8.30	0.1577	0.0993
0.74 - 0.73	622	622	100.0	4.93	11.92	8.40	0.1542	0.1029
0.73 - 0.71	1288	1288	100.0	4.77	10.93	7.82	0.1629	0.1114
0.71 - 0.70	729	730	99.9	4.21	9.45	6.54	0.1793	0.1331
0.70 - 0.69	804	804	100.0	4.39	9.17	6.57	0.1775	0.1341
0.69 - 0.68	792	846	93.6	3.72	9.00	6.03	0.1756	0.1473
0.78 - 0.68	6363	6418	99.1	4.75	10.99	7.87	0.1611	0.1105
Inf - 0.68	18862	18950	99.5	6.60	29.64	16.13	0.0845	0.0495

Complete .cif-data of the compound are available under the CCDC number CCDC-**2490748**.

Table S16: Crystal data and structure refinement of **3OTf**.

Identification code	16351	
Empirical formula	$C_{19} H_{25} Bi Cl_2 F_6 N_2 O_6 S_2$	
Color	colorless	
Formula weight	835.41 g·mol^{-1}	
Temperature	100(2) K	
Wavelength	0.71073 Å	
Crystal system	Triclinic	
Space group	*P*-1, (no. 2)	
Unit cell dimensions	a = 9.1000(12) Å	α= 81.089(3)°.
	b = 12.7117(15) Å	β= 75.126(3)°.
	c = 13.5571(17) Å	γ = 70.007(3)°.
Volume	1420.5(3) Å^3	
Z	2	
Density (calculated)	1.953 Mg·m^{-3}	
Absorption coefficient	6.617 mm^{-1}	
F(000)	808 e	
Crystal size	0.192 x 0.175 x 0.140 mm^3	
θ range for data collection	2.397 to 31.524°.	
Index ranges	$-13 \le h \le 13$, $-18\le k \le 18$, $-19 \le l \le 19$	
Reflections collected	125086	
Independent reflections	9453 [R_{int} = 0.0885]	
Reflections with $I>2\sigma(I)$	8799	
Completeness to θ = 25.242°	99.9 %	
Absorption correction	Gaussian	
Max. and min. transmission	0.60396 and 0.39178	
Refinement method	Full-matrix least-squares on F^2	
Data / restraints / parameters	9453 / 0 / 399	
Goodness-of-fit on F^2	1.045	
Final R indices [$I>2\sigma(I)$]	R_1 = 0.0211	wR^2 = 0.0476
R indices (all data)	R_1 = 0.0238	wR^2 = 0.0486
Extinction coefficient	n/a	
Largest diff. peak and hole	1.009 and -0.734 e·Å^{-3}	

Table S17: Bond lengths [Å] and angles [°] of **3OTf**.

Bi(1)-O(1)	2.3820(15)	Bi(1)-O(4)	2.4158(15)
Bi(1)-N(1)	2.4616(17)	Bi(1)-N(2)	2.4736(17)
Bi(1)-C(1)	2.1818(19)	S(1)-O(1)	1.4767(17)
S(1)-O(2)	1.4332(17)	S(1)-O(3)	1.4311(18)
S(1)-C(17)	1.826(3)	S(2)-O(4)	1.4747(15)
S(2)-O(5)	1.4324(17)	S(2)-O(6)	1.4235(18)
S(2)-C(18)	1.822(2)	F(1)-C(17)	1.316(3)
F(2)-C(17)	1.329(3)	F(3)-C(17)	1.329(3)
F(4)-C(18)	1.325(3)	F(5)-C(18)	1.324(3)
F(6)-C(18)	1.321(3)	N(1)-C(7)	1.273(3)
N(1)-C(8)	1.499(3)	N(2)-C(12)	1.277(3)
N(2)-C(13)	1.494(3)	C(1)-C(2)	1.388(3)
C(1)-C(6)	1.382(3)	C(2)-C(3)	1.395(3)
C(2)-C(7)	1.475(3)	C(3)-H(3)	0.9500
C(3)-C(4)	1.395(3)	C(4)-H(4)	0.9500
C(4)-C(5)	1.391(3)	C(5)-H(5)	0.9500
C(5)-C(6)	1.400(3)	C(6)-C(12)	1.474(3)
C(7)-H(7)	0.9500	C(8)-C(9)	1.526(4)
C(8)-C(10)	1.527(3)	C(8)-C(11)	1.518(3)
C(9)-H(9A)	0.9800	C(9)-H(9B)	0.9800
C(9)-H(9C)	0.9800	C(10)-H(10A)	0.9800
C(10)-H(10B)	0.9800	C(10)-H(10C)	0.9800
C(11)-H(11A)	0.9800	C(11)-H(11B)	0.9800
C(11)-H(11C)	0.9800	C(12)-H(12)	0.9500
C(13)-C(14A)	1.461(4)	C(13)-C(15A)	1.567(4)
C(13)-C(16A)	1.546(4)	C(13)-C(14B)	1.633(8)
C(13)-C(15B)	1.610(8)	C(13)-C(16B)	1.370(8)
C(14A)-H(14A)	0.9800	C(14A)-H(14B)	0.9800
C(14A)-H(14C)	0.9800	C(15A)-H(15A)	0.9800
C(15A)-H(15B)	0.9800	C(15A)-H(15C)	0.9800
C(16A)-H(16A)	0.9800	C(16A)-H(16B)	0.9800
C(16A)-H(16C)	0.9800	C(14B)-H(14D)	0.9800
C(14B)-H(14E)	0.9800	C(14B)-H(14F)	0.9800

C(15B)-H(15D)	0.9800	C(15B)-H(15E)	0.9800
C(15B)-H(15F)	0.9800	C(16B)-H(16D)	0.9800
C(16B)-H(16E)	0.9800	C(16B)-H(16F)	0.9800
Cl(1A)-C(19A)	1.777(5)	Cl(2A)-C(19A)	1.727(6)
C(19A)-H(19A)	0.9900	C(19A)-H(19B)	0.9900
Cl(1B)-C(19B)	1.778(14)	Cl(2B)-C(19B)	1.775(16)
C(19B)-H(19C)	0.9900	C(19B)-H(19D)	0.9900
O(1)-Bi(1)-O(4)	160.03(5)	O(1)-Bi(1)-N(1)	82.65(6)
O(1)-Bi(1)-N(2)	86.26(6)	O(4)-Bi(1)-N(1)	96.30(5)
O(4)-Bi(1)-N(2)	82.84(5)	N(1)-Bi(1)-N(2)	143.51(6)
C(1)-Bi(1)-O(1)	80.74(6)	C(1)-Bi(1)-O(4)	79.98(6)
C(1)-Bi(1)-N(1)	72.16(6)	C(1)-Bi(1)-N(2)	71.79(6)
O(1)-S(1)-C(17)	101.37(12)	O(2)-S(1)-O(1)	112.38(10)
O(2)-S(1)-C(17)	105.81(13)	O(3)-S(1)-O(1)	113.65(11)
O(3)-S(1)-O(2)	117.28(11)	O(3)-S(1)-C(17)	104.31(12)
O(4)-S(2)-C(18)	101.60(11)	O(5)-S(2)-O(4)	112.18(10)
O(5)-S(2)-C(18)	104.80(12)	O(6)-S(2)-O(4)	113.77(11)
O(6)-S(2)-O(5)	118.38(12)	O(6)-S(2)-C(18)	103.73(12)
S(1)-O(1)-Bi(1)	122.63(9)	S(2)-O(4)-Bi(1)	123.55(9)
C(7)-N(1)-Bi(1)	111.90(13)	C(7)-N(1)-C(8)	121.85(18)
C(8)-N(1)-Bi(1)	126.24(14)	C(12)-N(2)-Bi(1)	112.17(13)
C(12)-N(2)-C(13)	122.24(17)	C(13)-N(2)-Bi(1)	125.53(12)
C(2)-C(1)-Bi(1)	118.68(14)	C(6)-C(1)-Bi(1)	119.19(13)
C(6)-C(1)-C(2)	122.13(17)	C(1)-C(2)-C(3)	118.92(18)
C(1)-C(2)-C(7)	117.01(18)	C(3)-C(2)-C(7)	123.96(18)
C(2)-C(3)-H(3)	120.3	C(2)-C(3)-C(4)	119.34(19)
C(4)-C(3)-H(3)	120.3	C(3)-C(4)-H(4)	119.3
C(5)-C(4)-C(3)	121.35(19)	C(5)-C(4)-H(4)	119.3
C(4)-C(5)-H(5)	120.5	C(4)-C(5)-C(6)	119.10(19)
C(6)-C(5)-H(5)	120.5	C(1)-C(6)-C(5)	119.15(18)
C(1)-C(6)-C(12)	117.52(17)	C(5)-C(6)-C(12)	123.33(18)
N(1)-C(7)-C(2)	119.92(18)	N(1)-C(7)-H(7)	120.0
C(2)-C(7)-H(7)	120.0	N(1)-C(8)-C(9)	107.23(18)
N(1)-C(8)-C(10)	108.01(18)	N(1)-C(8)-C(11)	109.60(19)
C(9)-C(8)-C(10)	109.4(2)	C(11)-C(8)-C(9)	110.3(2)

C(11)-C(8)-C(10)	112.1(2)	C(8)-C(9)-H(9A)	109.5
C(8)-C(9)-H(9B)	109.5	C(8)-C(9)-H(9C)	109.5
H(9A)-C(9)-H(9B)	109.5	H(9A)-C(9)-H(9C)	109.5
H(9B)-C(9)-H(9C)	109.5	C(8)-C(10)-H(10A)	109.5
C(8)-C(10)-H(10B)	109.5	C(8)-C(10)-H(10C)	109.5
H(10A)-C(10)-H(10B)	109.5	H(10A)-C(10)-H(10C)	109.5
H(10B)-C(10)-H(10C)	109.5	C(8)-C(11)-H(11A)	109.5
C(8)-C(11)-H(11B)	109.5	C(8)-C(11)-H(11C)	109.5
H(11A)-C(11)-H(11B)	109.5	H(11A)-C(11)-H(11C)	109.5
H(11B)-C(11)-H(11C)	109.5	N(2)-C(12)-C(6)	119.28(18)
N(2)-C(12)-H(12)	120.4	C(6)-C(12)-H(12)	120.4
N(2)-C(13)-C(15A)	108.78(19)	N(2)-C(13)-C(16A)	106.49(18)
N(2)-C(13)-C(14B)	104.3(3)	N(2)-C(13)-C(15B)	113.3(3)
C(14A)-C(13)-N(2)	109.4(2)	C(14A)-C(13)-C(15A)	113.0(2)
C(14A)-C(13)-C(16A)	112.3(2)	C(16A)-C(13)-C(15A)	106.6(2)
C(15B)-C(13)-C(14B)	101.7(4)	C(16B)-C(13)-N(2)	111.3(4)
C(16B)-C(13)-C(14B)	112.8(5)	C(16B)-C(13)-C(15B)	112.8(5)
F(1)-C(17)-S(1)	111.59(19)	F(1)-C(17)-F(2)	108.9(2)
F(1)-C(17)-F(3)	108.5(2)	F(2)-C(17)-S(1)	109.37(19)
F(3)-C(17)-S(1)	109.85(19)	F(3)-C(17)-F(2)	108.6(2)
F(4)-C(18)-S(2)	110.64(18)	F(5)-C(18)-S(2)	110.85(19)
F(5)-C(18)-F(4)	107.5(2)	F(6)-C(18)-S(2)	111.23(18)
F(6)-C(18)-F(4)	108.3(2)	F(6)-C(18)-F(5)	108.2(2)
C(13)-C(14A)-H(14A)	109.5	C(13)-C(14A)-H(14B)	109.5
C(13)-C(14A)-H(14C)	109.5	H(14A)-C(14A)-H(14B)	109.5
H(14A)-C(14A)-H(14C)	109.5	H(14B)-C(14A)-H(14C)	109.5
C(13)-C(15A)-H(15A)	109.5	C(13)-C(15A)-H(15B)	109.5
C(13)-C(15A)-H(15C)	109.5	H(15A)-C(15A)-H(15B)	109.5
H(15A)-C(15A)-H(15C)	109.5	H(15B)-C(15A)-H(15C)	109.5
C(13)-C(16A)-H(16A)	109.5	C(13)-C(16A)-H(16B)	109.5
C(13)-C(16A)-H(16C)	109.5	H(16A)-C(16A)-H(16B)	109.5
H(16A)-C(16A)-H(16C)	109.5	H(16B)-C(16A)-H(16C)	109.5
C(13)-C(14B)-H(14D)	109.5	C(13)-C(14B)-H(14E)	109.5
C(13)-C(14B)-H(14F)	109.5	H(14D)-C(14B)-H(14E)	109.5
H(14D)-C(14B)-H(14F)	109.5	H(14E)-C(14B)-H(14F)	109.5
C(13)-C(15B)-H(15D)	109.5	C(13)-C(15B)-H(15E)	109.5

C(13)-C(15B)-H(15F)	109.5	H(15D)-C(15B)-H(15E)	109.5
H(15D)-C(15B)-H(15F)	109.5	H(15E)-C(15B)-H(15F)	109.5
C(13)-C(16B)-H(16D)	109.5	C(13)-C(16B)-H(16E)	109.5
C(13)-C(16B)-H(16F)	109.5	H(16D)-C(16B)-H(16E)	109.5
H(16D)-C(16B)-H(16F)	109.5	H(16E)-C(16B)-H(16F)	109.5
Cl(1A)-C(19A)-H(19A)	109.2	Cl(1A)-C(19A)-H(19B)	109.2
Cl(2A)-C(19A)-Cl(1A)	112.2(3)	Cl(2A)-C(19A)-H(19A)	109.2
Cl(2A)-C(19A)-H(19B)	109.2	H(19A)-C(19A)-H(19B)	107.9
Cl(1B)-C(19B)-H(19C)	109.3	Cl(1B)-C(19B)-H(19D)	109.3
Cl(2B)-C(19B)-Cl(1B)	111.5(10)	Cl(2B)-C(19B)-H(19C)	109.3
Cl(2B)-C(19B)-H(19D)	109.3	H(19C)-C(19B)-H(19D)	108.0

Table S18: Hydrogen coordinates and isotropic displacement parameters (Å^2) in the solid state structure of **3OTf**.

	x	y	z	Ueq
H(3)	0.3057	0.2732	-0.1736	0.027
H(4)	0.0322	0.3654	-0.1643	0.027
H(5)	-0.1440	0.4154	-0.0075	0.023
H(7)	0.5409	0.1882	-0.0789	0.025
H(9A)	0.6671	0.1894	0.2053	0.066
H(9B)	0.7881	0.0639	0.1972	0.066
H(9C)	0.5989	0.0854	0.2321	0.066
H(10A)	0.6189	-0.0296	0.0914	0.052
H(10B)	0.8076	-0.0487	0.0585	

0.052				
H(10C)	0.6985	0.0026	-0.0240	0.052
H(11A)	0.7915	0.1689	-0.0648	0.071
H(11B)	0.9000	0.1317	0.0185	0.071
H(11C)	0.7619	0.2507	0.0219	0.071
H(12)	-0.1866	0.4194	0.1890	0.024
H(14A)	-0.2305	0.5428	0.3221	0.046
H(14B)	-0.0682	0.5346	0.3528	0.046
H(14C)	-0.2203	0.5249	0.4400	0.046
H(15A)	-0.3161	0.3674	0.3424	0.045
H(15B)	-0.3062	0.3492	0.4603	0.045
H(15C)	-0.2011	0.2524	0.3846	0.045
H(16A)	0.0463	0.2425	0.4278	0.040
H(16B)	-0.0512	0.3357	0.5081	0.040
H(16C)	0.0990	0.3516	0.4224	0.040
H(14D)	0.0930	0.4133	0.4050	0.044
H(14E)	-0.0763	0.4849	0.4694	0.044
H(14F)	-0.0144	0.5248	0.3529	0.044
H(15D)	-0.2738	0.5365	0.3150	

0.042			
H(15E)	-0.3274	0.5021	0.4341
0.042			
H(15F)	-0.3496	0.4365	0.3501
0.042			
H(16D)	0.0027	0.2384	0.4207
0.048			
H(16E)	-0.1721	0.2535	0.4053
0.048			
H(16F)	-0.1530	0.3095	0.4972
0.048			
H(19A)	0.3152	0.0869	0.5285
0.044			
H(19B)	0.4422	0.0759	0.5953
0.044			
H(19C)	0.3913	0.0987	0.5047
0.059			
H(19D)	0.4853	0.1186	0.5814
0.059			

14.8 Single crystal structure analysis of **2BF4**

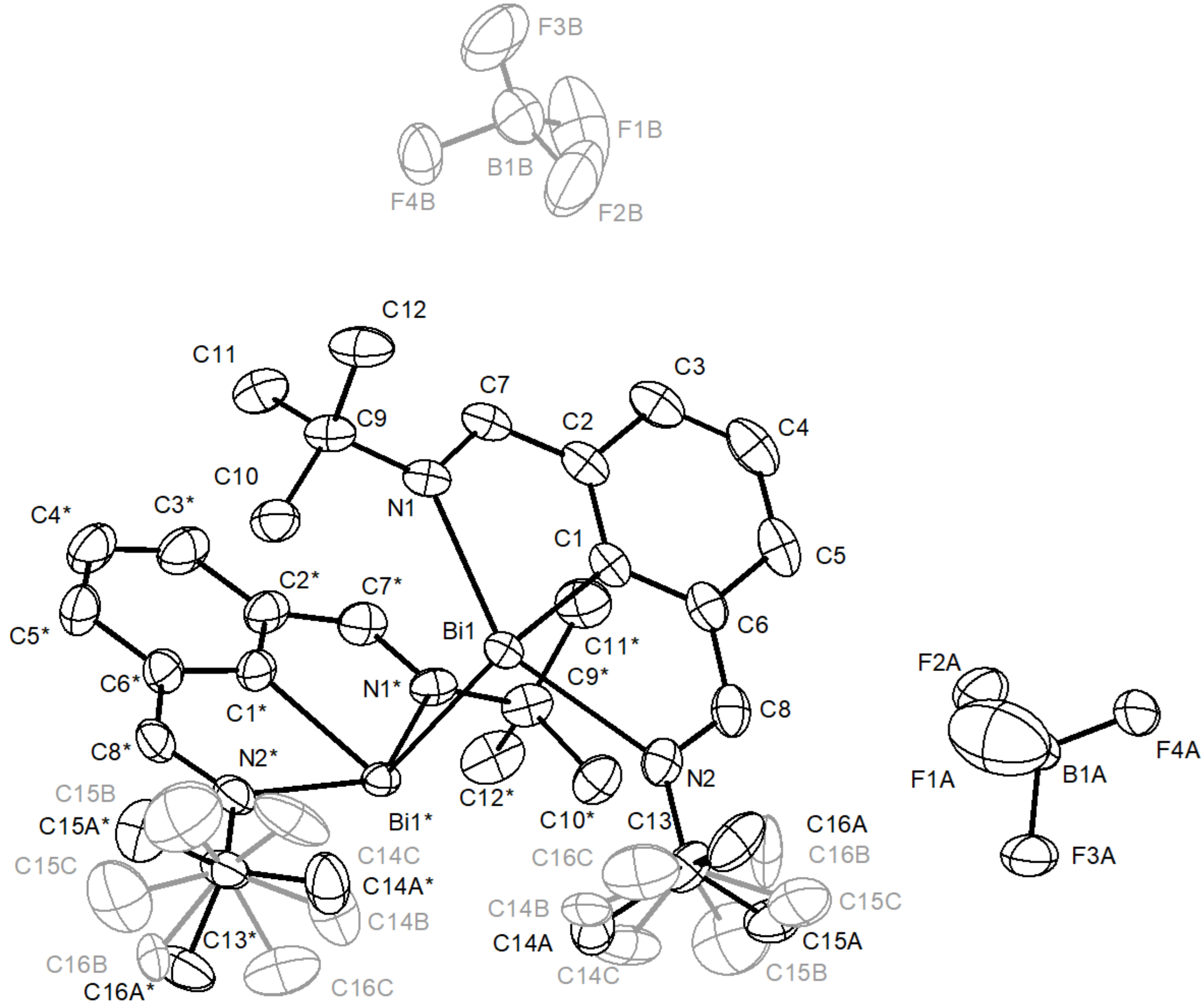

Figure S88: The molecular structure of **2BF4**. The H atoms have been removed for clarity. Main structure shown in black and disordered parts shown in grey.

X-ray Crystal Structure Analysis of **2BF4**:

C_{18} H_{27} B Bi F_4 N_2 $O_{0.50}$, M_r = 575.20 g mol^{-1}, yellow prism, crystal size 0.26 x 0.26 x 0.20 mm^3, Tetragonal, space group *P*4/*ncc* [130], a = 20.6282(6) Å, b = 20.0238(10) Å, V = 8520.6(7) Å^3, T = 100(2) K, Z = 16, D_{calc} = 1.794 g·cm^3, λ = 0.71073 Å, μ(*Mo-K*$_{\alpha}$) = 8.317 mm^{-1}, Gaussian absorption correction ($T_{\min}$ = 0.17303, $T_{\max}$ = 0.27986), Bruker-AXS Kappa Mach3 with APEX-II detector and IµS microfocus Mo-anode X-ray source, 1.396 < θ < 33.369°, 296380 measured reflections, 8272 independent reflections, 6552 reflections with $I > 2\sigma(I)$, R_{int} = 0.0747. The structure was solved by *SHELXT* and refined by full-matrix least-squares (*SHELXL*) against F^2 to R_1 = 0.0319 [$I > 2\sigma(I)$], wR_2 = 0.0796 [all data], 332 parameters and 166 restraints.

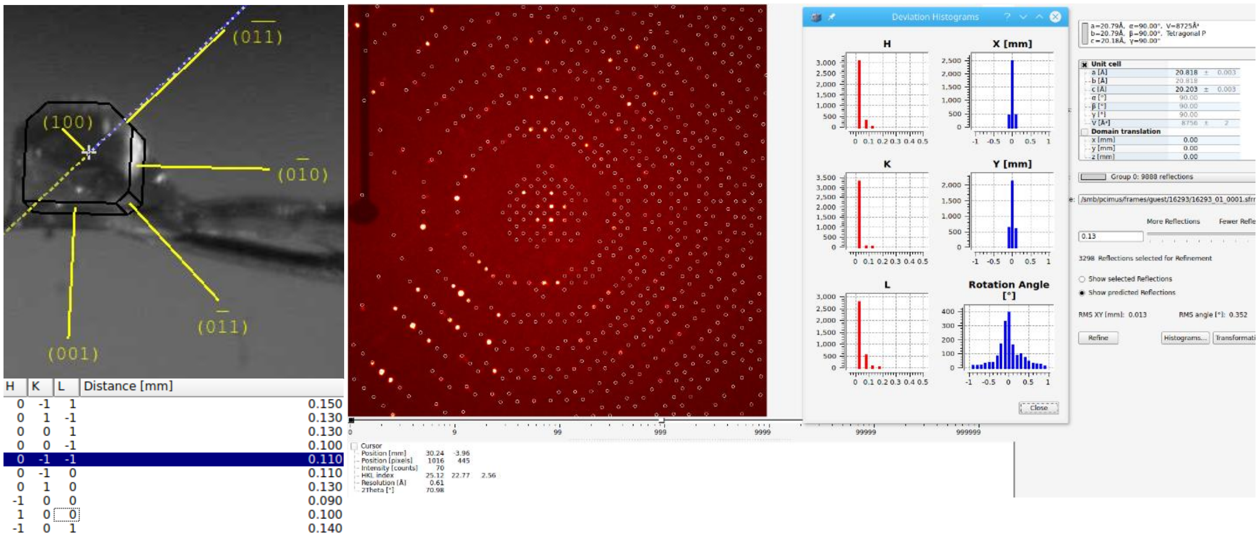

Figure S89: Crystal faces and unit cell determination/refinement of **2BF4**.

INTENSITY STATISTICS FOR DATASET

Resolution	#Data	#Theory	%Complete	Redundancy	Mean I	Mean I/s	Rmerge	Rsigma
Inf - 2.88	139	141	98.6	48.93	43.05	59.65	0.0479	0.0144
2.88 - 1.85	323	323	100.0	60.92	34.47	65.78	0.0550	0.0129
1.85 - 1.44	468	468	100.0	64.21	21.46	61.54	0.0637	0.0120
1.44 - 1.25	449	449	100.0	65.02	16.67	57.85	0.0714	0.0128
1.25 - 1.13	465	465	100.0	62.72	11.92	53.64	0.0776	0.0127
1.13 - 1.04	487	487	100.0	48.12	9.31	42.47	0.0776	0.0150
1.04 - 0.98	431	431	100.0	39.20	7.77	36.28	0.0761	0.0172
0.98 - 0.93	439	439	100.0	34.21	7.41	31.11	0.0771	0.0194
0.93 - 0.88	558	558	100.0	30.21	5.93	28.59	0.0830	0.0224
0.88 - 0.85	395	395	100.0	28.75	4.37	24.87	0.0939	0.0254
0.85 - 0.82	441	441	100.0	27.69	3.96	22.23	0.1023	0.0286
0.82 - 0.79	525	525	100.0	26.76	3.81	20.65	0.1081	0.0304
0.79 - 0.77	390	390	100.0	26.10	3.08	18.33	0.1253	0.0349
0.77 - 0.75	435	435	100.0	24.88	3.05	17.12	0.1295	0.0365
0.75 - 0.73	487	487	100.0	24.50	2.69	16.23	0.1389	0.0404
0.73 - 0.71	527	527	100.0	23.50	2.13	13.74	0.1595	0.0498
0.71 - 0.69	611	611	100.0	22.86	2.13	13.32	0.1727	0.0524
0.69 - 0.68	331	331	100.0	21.52	1.64	10.43	0.1994	0.0678
0.68 - 0.67	325	325	100.0	22.14	1.76	11.76	0.1981	0.0627
0.67 - 0.65	906	930	97.4	18.39	1.39	8.76	0.2423	0.0963
0.75 - 0.65	3187	3211	99.3	21.71	1.92	12.08	0.1788	0.0607
Inf - 0.65	9132	9158	99.7	34.45	7.53	28.33	0.0735	0.0203

Complete .cif-data of the compound are available under the CCDC number CCDC-**2490751**.

The structure contained a disordered solute molecule, which could not be properly refined.

A solvent mask was calculated and 290 electrons were found in a volume of 1370 Å³ in 2 void per unit cell. This is consistent with the presence of 1[C_4H_8O] per asymmetric unit which account for 320 electrons per unit cell. The solvent mask (SQUEEZE routine in Olex2) was applied leading

into a structure with a void volume of 1150.99 Å³ which belongs to 13.5% of the unit cell volume (probe radius of 1.2 Å and an approx. grid spacing of 0.7 Å).

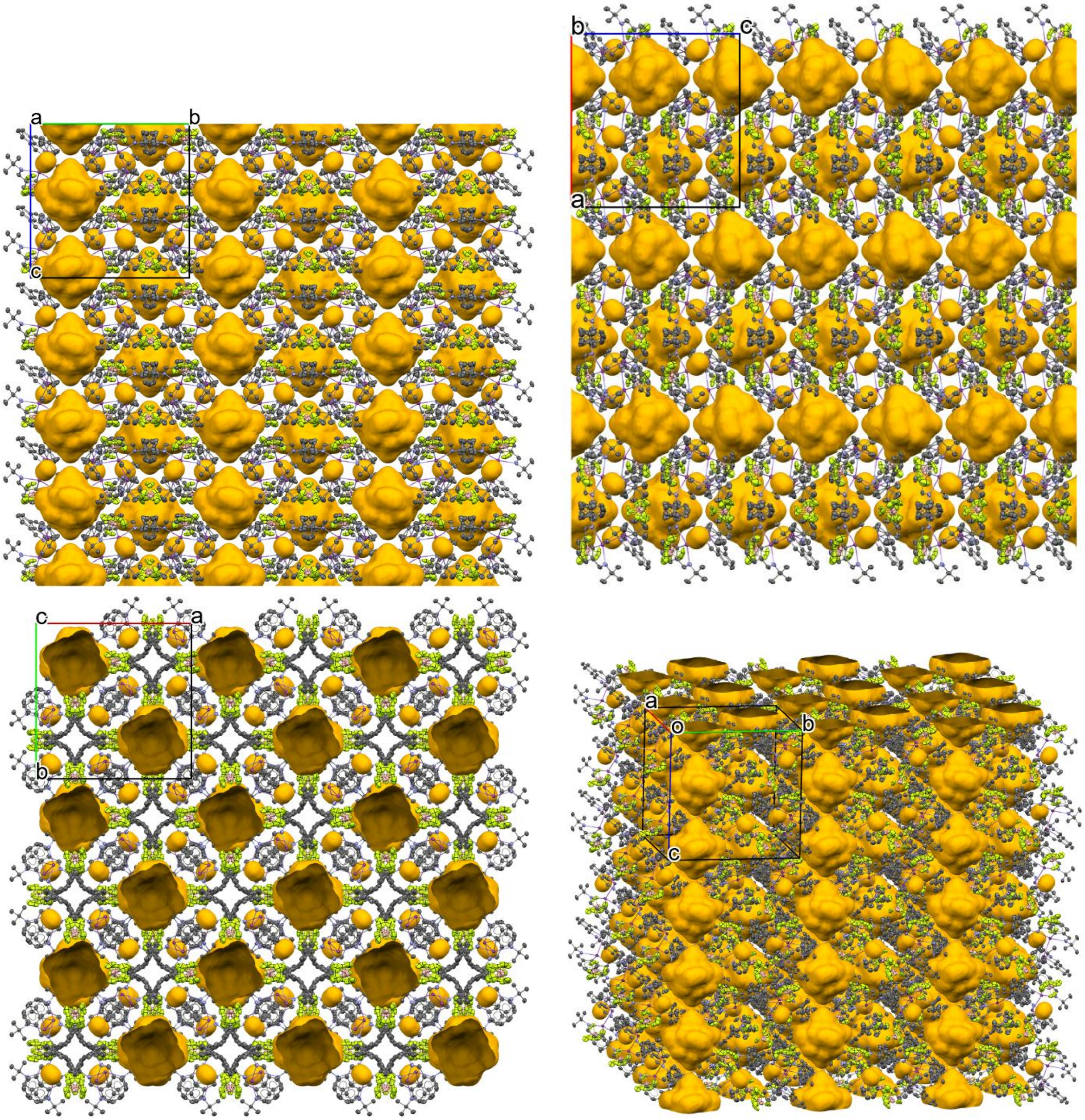

Figure S90: 3 x 3 x 3 packing motive of the unit cell and solvent accessible voids viewed along crystallographic a- (top left), b- (top right), c-axis (bottom left) and in a random orientation (bottom right)

Table S19: Crystal data and structure refinement of **$2BF_4$**.

Identification code	16293	
Empirical formula	C_{18} H_{27} B Bi F_4 N_2 $O_{0.50}$	
Color	yellow	
Formula weight	575.20 g·mol^{-1}	
Temperature	100(2) K	
Wavelength	0.71073 Å	
Crystal system	Tetragonal	
Space group	*P*4/*ncc*, (no. 130)	
Unit cell dimensions	a = 20.6282(6) Å	α= 90°.
	b = 20.6282(6) Å	β= 90°.
	c = 20.0238(10) Å	γ = 90°.
Volume	8520.6(7) Å^3	
Z	16	
Density (calculated)	1.794 Mg·m^{-3}	
Absorption coefficient	8.317 mm^{-1}	
F(000)	4432 e	
Crystal size	0.26 x 0.26 x 0.20 mm^3	
θ range for data collection	1.396 to 33.369°.	
Index ranges	-31 ≤ h ≤ 31, -31≤ k ≤ 31, -30 ≤ l ≤ 30	
Reflections collected	296380	
Independent reflections	8272 [R_{int} = 0.0747]	
Reflections with I>2σ(I)	6552	
Completeness to θ = 25.242°	100.0 %	
Absorption correction	Gaussian	
Max. and min. transmission	0.27986 and 0.17303	
Refinement method	Full-matrix least-squares on F^2	
Data / restraints / parameters	8272 / 166 / 332	
Goodness-of-fit on F^2	1.126	
Final R indices [I>2σ(I)]	R_1 = 0.0319	wR^2 = 0.0704
R indices (all data)	R_1 = 0.0462	wR^2 = 0.0796
Extinction coefficient	n/a	
Largest diff. peak and hole	1.317 and -1.813 e·Å^{-3}	

Table S20: Bond lengths [Å] and angles [°] of **2BF$_4$**.

Bi(1)-Bi(1)#1	3.0794(2)	Bi(1)-N(1)	2.461(3)
Bi(1)-N(2)	2.542(3)	Bi(1)-C(1)	2.193(4)
N(1)-C(7)	1.285(5)	N(1)-C(9)	1.489(5)
N(2)-C(8)	1.279(5)	N(2)-C(13)	1.493(5)
C(1)-C(2)	1.386(5)	C(1)-C(6)	1.390(6)
C(2)-C(3)	1.396(5)	C(2)-C(7)	1.457(6)
C(3)-H(3)	0.9500	C(3)-C(4)	1.390(7)
C(4)-H(4)	0.9500	C(4)-C(5)	1.385(7)
C(5)-H(5)	0.9500	C(5)-C(6)	1.400(6)
C(6)-C(8)	1.463(6)	C(7)-H(7)	0.9500
C(8)-H(8)	0.9500	C(9)-C(10)	1.525(6)
C(9)-C(11)	1.522(6)	C(9)-C(12)	1.540(6)
C(10)-H(10A)	0.9800	C(10)-H(10B)	0.9800
C(10)-H(10C)	0.9800	C(11)-H(11A)	0.9800
C(11)-H(11B)	0.9800	C(11)-H(11C)	0.9800
C(12)-H(12A)	0.9800	C(12)-H(12B)	0.9800
C(12)-H(12C)	0.9800	C(13)-C(14A)	1.456(7)
C(13)-C(15A)	1.547(6)	C(13)-C(16A)	1.527(6)
C(13)-C(14B)	1.510(9)	C(13)-C(15B)	1.516(9)
C(13)-C(16B)	1.521(8)	C(13)-C(14C)	1.560(8)
C(13)-C(15C)	1.508(8)	C(13)-C(16C)	1.546(8)
C(14A)-H(14A)	0.9800	C(14A)-H(14B)	0.9800
C(14A)-H(14C)	0.9800	C(15A)-H(15A)	0.9800
C(15A)-H(15B)	0.9800	C(15A)-H(15C)	0.9800
C(16A)-H(16A)	0.9800	C(16A)-H(16B)	0.9800
C(16A)-H(16C)	0.9800	C(14B)-H(14D)	0.9800
C(14B)-H(14E)	0.9800	C(14B)-H(14F)	0.9800
C(15B)-H(15D)	0.9800	C(15B)-H(15E)	0.9800
C(15B)-H(15F)	0.9800	C(16B)-H(16D)	0.9800
C(16B)-H(16E)	0.9800	C(16B)-H(16F)	0.9800
C(14C)-H(14G)	0.9800	C(14C)-H(14H)	0.9800
C(14C)-H(14I)	0.9800	C(15C)-H(15G)	0.9800
C(15C)-H(15H)	0.9800	C(15C)-H(15I)	0.9800

C(16C)-H(16G)	0.9800	C(16C)-H(16H)	0.9800
C(16C)-H(16I)	0.9800	F(1A)-B(1A)	1.370(14)
F(2A)-B(1A)	1.373(12)	F(3A)-B(1A)	1.384(9)
F(4A)-B(1A)	1.339(10)	F(1B)-B(1B)	1.44(4)
F(2B)-B(1B)	1.32(5)	F(3B)-B(1B)	1.12(4)
F(4B)-B(1B)	1.36(4)		

N(1)-Bi(1)-Bi(1)#1	98.89(7)	N(1)-Bi(1)-N(2)	142.68(11)
N(2)-Bi(1)-Bi(1)#1	88.65(7)	C(1)-Bi(1)-Bi(1)#1	100.88(10)
C(1)-Bi(1)-N(1)	71.93(13)	C(1)-Bi(1)-N(2)	70.75(13)
C(7)-N(1)-Bi(1)	111.9(3)	C(7)-N(1)-C(9)	120.3(3)
C(9)-N(1)-Bi(1)	127.5(2)	C(8)-N(2)-Bi(1)	110.9(3)
C(8)-N(2)-C(13)	120.1(3)	C(13)-N(2)-Bi(1)	128.2(2)
C(2)-C(1)-Bi(1)	118.6(3)	C(2)-C(1)-C(6)	121.0(4)
C(6)-C(1)-Bi(1)	119.9(3)	C(1)-C(2)-C(3)	119.3(4)
C(1)-C(2)-C(7)	117.5(3)	C(3)-C(2)-C(7)	123.0(4)
C(2)-C(3)-H(3)	120.1	C(4)-C(3)-C(2)	119.9(4)
C(4)-C(3)-H(3)	120.1	C(3)-C(4)-H(4)	119.6
C(5)-C(4)-C(3)	120.8(4)	C(5)-C(4)-H(4)	119.6
C(4)-C(5)-H(5)	120.3	C(4)-C(5)-C(6)	119.5(4)
C(6)-C(5)-H(5)	120.3	C(1)-C(6)-C(5)	119.6(4)
C(1)-C(6)-C(8)	118.0(4)	C(5)-C(6)-C(8)	122.4(4)
N(1)-C(7)-C(2)	120.0(4)	N(1)-C(7)-H(7)	120.0
C(2)-C(7)-H(7)	120.0	N(2)-C(8)-C(6)	119.9(4)
N(2)-C(8)-H(8)	120.0	C(6)-C(8)-H(8)	120.0
N(1)-C(9)-C(10)	107.1(3)	N(1)-C(9)-C(11)	110.0(3)
N(1)-C(9)-C(12)	108.0(3)	C(10)-C(9)-C(12)	109.6(3)
C(11)-C(9)-C(10)	110.6(4)	C(11)-C(9)-C(12)	111.4(4)
C(9)-C(10)-H(10A)	109.5	C(9)-C(10)-H(10B)	109.5
C(9)-C(10)-H(10C)	109.5	H(10A)-C(10)-H(10B)	109.5
H(10A)-C(10)-H(10C)	109.5	H(10B)-C(10)-H(10C)	109.5
C(9)-C(11)-H(11A)	109.5	C(9)-C(11)-H(11B)	109.5
C(9)-C(11)-H(11C)	109.5	H(11A)-C(11)-H(11B)	109.5
H(11A)-C(11)-H(11C)	109.5	H(11B)-C(11)-H(11C)	109.5
C(9)-C(12)-H(12A)	109.5	C(9)-C(12)-H(12B)	109.5
C(9)-C(12)-H(12C)	109.5	H(12A)-C(12)-H(12B)	109.5

H(12A)-C(12)-H(12C)	109.5	H(12B)-C(12)-H(12C)	109.5
N(2)-C(13)-C(15A)	110.4(5)	N(2)-C(13)-C(16A)	103.5(5)
N(2)-C(13)-C(14B)	106.0(11)	N(2)-C(13)-C(15B)	111.5(11)
N(2)-C(13)-C(16B)	107.8(11)	N(2)-C(13)-C(14C)	104.7(6)
N(2)-C(13)-C(15C)	119.6(7)	N(2)-C(13)-C(16C)	107.4(7)
C(14A)-C(13)-N(2)	109.2(6)	C(14A)-C(13)-C(15A)	112.0(4)
C(14A)-C(13)-C(16A)	113.2(5)	C(16A)-C(13)-C(15A)	108.2(4)
C(14B)-C(13)-C(15B)	110.8(6)	C(14B)-C(13)-C(16B)	110.5(6)
C(15B)-C(13)-C(16B)	110.2(6)	C(15C)-C(13)-C(14C)	108.5(5)
C(15C)-C(13)-C(16C)	109.2(5)	C(16C)-C(13)-C(14C)	106.6(5)
C(13)-C(14A)-H(14A)	109.5	C(13)-C(14A)-H(14B)	109.5
C(13)-C(14A)-H(14C)	109.5	H(14A)-C(14A)-H(14B)	109.5
H(14A)-C(14A)-H(14C)	109.5	H(14B)-C(14A)-H(14C)	109.5
C(13)-C(15A)-H(15A)	109.5	C(13)-C(15A)-H(15B)	109.5
C(13)-C(15A)-H(15C)	109.5	H(15A)-C(15A)-H(15B)	109.5
H(15A)-C(15A)-H(15C)	109.5	H(15B)-C(15A)-H(15C)	109.5
C(13)-C(16A)-H(16A)	109.5	C(13)-C(16A)-H(16B)	109.5
C(13)-C(16A)-H(16C)	109.5	H(16A)-C(16A)-H(16B)	109.5
H(16A)-C(16A)-H(16C)	109.5	H(16B)-C(16A)-H(16C)	109.5
C(13)-C(14B)-H(14D)	109.5	C(13)-C(14B)-H(14E)	109.5
C(13)-C(14B)-H(14F)	109.5	H(14D)-C(14B)-H(14E)	109.5
H(14D)-C(14B)-H(14F)	109.5	H(14E)-C(14B)-H(14F)	109.5
C(13)-C(15B)-H(15D)	109.5	C(13)-C(15B)-H(15E)	109.5
C(13)-C(15B)-H(15F)	109.5	H(15D)-C(15B)-H(15E)	109.5
H(15D)-C(15B)-H(15F)	109.5	H(15E)-C(15B)-H(15F)	109.5
C(13)-C(16B)-H(16D)	109.5	C(13)-C(16B)-H(16E)	109.5
C(13)-C(16B)-H(16F)	109.5	H(16D)-C(16B)-H(16E)	109.5
H(16D)-C(16B)-H(16F)	109.5	H(16E)-C(16B)-H(16F)	109.5
C(13)-C(14C)-H(14G)	109.5	C(13)-C(14C)-H(14H)	109.5
C(13)-C(14C)-H(14I)	109.5	H(14G)-C(14C)-H(14H)	109.5
H(14G)-C(14C)-H(14I)	109.5	H(14H)-C(14C)-H(14I)	109.5
C(13)-C(15C)-H(15G)	109.5	C(13)-C(15C)-H(15H)	109.5
C(13)-C(15C)-H(15I)	109.5	H(15G)-C(15C)-H(15H)	109.5
H(15G)-C(15C)-H(15I)	109.5	H(15H)-C(15C)-H(15I)	109.5
C(13)-C(16C)-H(16G)	109.5	C(13)-C(16C)-H(16H)	109.5
C(13)-C(16C)-H(16I)	109.5	H(16G)-C(16C)-H(16H)	109.5

H(16G)-C(16C)-H(16I)	109.5	H(16H)-C(16C)-H(16I)	109.5
F(1A)-B(1A)-F(2A)	107.4(9)	F(1A)-B(1A)-F(3A)	104.7(8)
F(2A)-B(1A)-F(3A)	111.5(8)	F(4A)-B(1A)-F(1A)	103.3(10)
F(4A)-B(1A)-F(2A)	111.8(8)	F(4A)-B(1A)-F(3A)	117.1(7)
F(2B)-B(1B)-F(1B)	97(3)	F(2B)-B(1B)-F(4B)	111(3)
F(3B)-B(1B)-F(1B)	121(4)	F(3B)-B(1B)-F(2B)	120(4)
F(3B)-B(1B)-F(4B)	96(3)	F(4B)-B(1B)-F(1B)	112(3)

Symmetry transformations used to generate equivalent atoms:

#1 -y+1,-x+1,-z+3/2

15. References

[1] Wang, F.; Planas, O.; Cornella, J. Bi(I)-Catalyzed Transfer-Hydrogenation with Ammonia-Borane. *J. Am. Chem. Soc.* **2019**, *141*, 4235–4240.

[2] Vránová, I.; Jambor, R.; Růžička, A.; Jirásko, R.; Dostál, L. Reactivity of N,C,N-Chelated Antimony(III) and Bismuth(III) Chlorides with Lithium Reagents: Addition vs Substitution. *Organometallics,* **2015**, *34*, 534–541.

[3] Mato, M.; Cleto Bruzzese, P.; Takahashi, F.; Leutzsch, M.; Reijerse, E. J.; Schnegg, A.; Cornella, J. Oxidative Addition of Aryl Electrophiles into a Red-Light-Active Bismuthinidene. *J. Am. Chem. Soc.* **2023**, *145*, 18742–18747.

[4] Moon, H. W.; Wang, F.; Bhattacharyya, K.; Planas, O.; Leutzsch, M.; Nöthling, N.; Auer, A. A.; Cornella, J. Mechanistic Studies on the Bismuth-Catalyzed Transfer Hydrogenation of Azoarenes. *Angew. Chem. Int. Ed.* **2023**, *62*, e202313578.

[5] Ganesamoorthy, C.; Wölper, C.; Dostál, L.; Schulz, S. Syntheses and Structures of N,C,N-Stabilized Antimony Chalcogenides. *J. Organomet. Chem.* **2017**, *845*, 38–43.

[6] Béland, V. A.; Nöthling, N.; Leutzsch, M.; Cornella, J. Activation and Catalytic Degradation of SF_6 and $PhSF_5$ at a Bismuth Center. *J. Am. Chem. Soc.* **2024**, *146*, 25409–25415.

[7] Bennett, B. K.; Harrison, R. G.; Richmond, T. G. Cobaltocenium Fluoride: A Novel Source of "Naked" Fluoride Formed by Carbon-Fluorine Bond Activation in a Saturated Perfluorocarbon. *J. Am. Chem. Soc.* **1994**, *116*, 11165–11166.

[8] Dunaj, T.; Schwarzmann, J.; Ramler, J.; Stoy, S.; Reith, S.; Nitzsche, J.; Völlinger, L.; von Hänisch, C.; Lichtenberg, C. Bismuth Cations: Fluoride Ion Abstraction, Isocyanide Coordination, and Impact of Steric Bulk on Lewis Acidity. *Chem. Eur. J.* **2023**, *29*, e202204012.

[9] Yang, X.; Kuziola, J.; Béland, V. A.; Busch, J.; Leutzsch, M.; Burés, J.; Cornella, J. Bismuth-Catalyzed Amide Reduction. *Angew. Chem. Int. Ed.* **2023**, *62*, e202306447.

[10] Stamoulis, A.; Mato, M.; Bruzzese, P. C.; Leutzsch, M.; Cadranel, A.; Gil-Sepulcre, M.; Neese, F.; Cornella, J. Red-Light-Active *N,C,N*-Pincer Bismuthinidene: Excited State Dynamics and Mechanism of Oxidative Addition into Aryl Iodides. *J. Am. Chem. Soc.* **2025**, *147*, 6037–6048.

[11] Mayer, U.; Gutmann, V.; Gerger, W. The Acceptor Number – A Quantitative Empirical Parameter for the Electrophilic Properties of Solvents. *Monatsh. Chem.* **1975**, *106*, 1235–1257.

[12] Beckett, M. A.; Strickland, G. C.; Holland, J. R.; Sukumar Varma, K. A Convenient n.m.r. Method for the Measurement of Lewis Acidity at Boron Centres: Correlation of Reaction Rates of Lewis Acid Initiated Epoxide Polymerizations with Lewis Acidity. *Polymer* **1996**, *37*, 4629–4631.

[13] Ramler, J.; Lichtenberg, C. Molecular Bismuth Cations: Assessment of Soft Lewis Acidity. *Chem. Eur. J.* **2020**, *26*, 10250–10258.

[14] Díaz-Torres, R.; Alvarez, S. Coordinating Ability of Anions and Solvents towards Transition Metals and Lanthanides. *Dalton Trans.* **2011**, *40*, 10742.

[15] Alvarez, S. Coordinating Ability of Anions, Solvents, Amino Acids, and Gases towards Alkaline and Alkaline-Earth Elements, Transition Metals, and Lanthanides. *Chem. Eur. J.* **2020**, *26*, 4350–4377.

[16] CCDC Refcodes: COMMAV, EYABUC, FOQPAC, FUBLUM, KOFDEO, OJAKOC, PIHFAN01, QAKCAG, QIBGOY, TITLEN, VUGTEZ, VUGVIF, ZUCJIQ.

[17] CCDC Refcodes: EYABUC01, PIHFAN01, QIBGOY01, TAGPUM01.

[18] CCDC Refcode: POZXEK.

[19] CCDC Refcodes: ADUTAX, AVAQAQ, AVAQEU, BUFSAX, HAWCOX, LIHQIC, ROCCOC, TAGPUM, VUGSIC, XUKTII.

[20] Carmalt, C. J.; Farrugia, L. J.; Norman, N. C. Cationic, Arylbismuth(III) Complexes of the Form $[BiR_2L_2]^+$ and $[BiRL_4]^{2+}$ Where L Is a Neutral Two-Electron Donor Ligand. *J. Chem. Soc. Dalton Trans.* **1996**, 443–454.

[21] Planas, O.; Wang, F.; Leutzsch, M.; Cornella, J. Fluorination of Arylboronic Esters Enabled by Bismuth Redox Catalysis. *Science* **2020**, *367*, 313–317.

[22] Nejman, P. S.; Curzon, T. E.; Bühl, M.; McKay, D.; Woollins, J. D.; Ashbrook, S. E.; Cordes, D. B.; Slawin, A. M. Z.; Kilian, P. Phosphorus–Bismuth *Peri*-Substituted Acenaphthenes: A Synthetic, Structural, and Computational Study. *Inorg. Chem.* **2020**, *59*, 5616–5625.

[23] Ramler, J.; Hofmann, K.; Lichtenberg, C. Neutral and Cationic Bismuth Compounds: Structure, Heteroaromaticity, and Lewis Acidity of Bismepines. *Inorg. Chem.* **2020**, *59*, 3367–3376.

[24] Hunger, M.; Limberg, C.; Kircher, P. Syntheses of Heteronuclear Molybdenum/Bismuth Alkoxides Stabilized by Organic Ligands. *Organometallics* **2000**, *19*, 1044–1050.

[25] García-Romero, Á.; Plajer, A. J.; Miguel, D.; Wright, D. S.; Bond, A. D.; Álvarez, C. M.; García-Rodríguez, R. *Tris* (2-Pyridyl) Bismuthines: Coordination Chemistry, Reactivity, and Anion-Triggered Pyridyl Coupling. *Inorg. Chem.* **2020**, *59*, 7103–7116.

[26] Matano, Y.; Azuma, N.; Suzuki, H. Synthesis, X-Ray Structure and Reactions of (2-Oxoalkyl)Triarylbismuthonium Salts. *J. Chem. Soc. Perkin Trans.* **1994**, 1739.

[27] Matano, Y.; Yoshimune, M.; Azuma, N.; Suzuki, H. First Synthesis, X-Ray Structure Analysis and Reactions of Alkenyltriphenylbismuthonium Salts. *J. Chem. Soc. Perkin Trans.* **1996**, 1971.

[28] Matano, Y. Synthesis, Structure, and Reactions of Triaryl(Methyl)Bismuthonium Salts. *Organometallics* **2000**, *19*, 2258–2263.

[29] Matano, Y. First Synthesis of Alkynyltriphenylbismuthonium Salts and Their Dual Reaction Modes in Sulfonylation. *Chem. Commun.* **2000**, 2233–2234.

[30] Matano, Y.; Suzuki, T.; Shinokura, T.; Imahori, H. Mesityltriphenylbismuthonium Tetrafluoroborate as an Efficient Bismuth(V) Oxidant: Remarkable Steric Effects on Reaction Rates and Chemoselectivities in Alcohol Oxidation. *Tetrahedron Lett.* **2007**, *48*, 2885–2888.

[31] Park, G.; Brock, D. J.; Pellois, J. P.; Gabbaï, F. P. Heavy Pnictogenium Cations as Transmembrane Anion Transporters in Vesicles and Erythrocytes. *Chem* **2019**, *5*, 2215–2227.

[32] Qiu, R.; Yin, S.; Song, X.; Meng, Z.; Qiu, Y.; Tan, N.; Xu, X.; Luo, S.; Dai, F. R.; Au, C. T.; Wong, W. Y. Effect of Butterfly-Shaped Sulfur-Bridged Ligand and Counter Anions on the Catalytic Activity and Diastereoselectivity of Organobismuth Complexes. *Dalton Trans.* **2011**, *40*, 9482.

[33] Kratzert, D.; Holstein, J. J.; Krossing, I. DSR: enhanced modelling and refinement of disordered structures with SHELXL. *J. Appl. Crystallogr.* **2015**, *48*, 933–938.

[34] Kratzert, D.; Krossing, I. Recent improvements in DSR. *J. Appl. Crystallogr.* **2018**, *51*, 928–934.

[35] Kleemiss, F.; Dolomanov, O. V.; Bodensteiner, M.; Peyerimhoff, N.; Midgley, L.; Bourhis, L. J.; Genoni, A.; Malaspina, L. A.; Jayatilaka, D.; Spencer, J. L.; White, F.; Grundkötter-Stock, B.;

Steinhauer, S.; Lentz, D.; Puschmann, H.; Grabowsky, S. Accurate crystal structures and chemical properties from NoSpherA2. *Chem. Sci.* **2021**, *12*, 1675–1692.
